# Site-Selective C–H Functionalization on Coumarins Directed by Manganese: Mechanistic Insights from Time-Resolved Spectroscopy and Catalytic Development

**DOI:** 10.1021/acsorginorgau.6c00003

**Published:** 2026-04-13

**Authors:** Thomas J. Burden, Jonathan B. Eastwood, Emily A. Thompson, Matteo Albino, Ian P. Clark, Adrian C. Whitwood, Jean-Philippe Krieger, Matthew J. Harper, Huw T. Jenkins, Ian J. S. Fairlamb, Jason M. Lynam

**Affiliations:** a Department of Chemistry, 8748 University of York, Heslington, York YO10 5DD, U.K.; b Central Laser Facility, Research Complex at Harwell, STFC Rutherford Appleton Laboratory, Harwell Campus, Didcot, Oxfordshire OX11 0QX, U.K.; c Syngenta Crop Protection AG, Breitenloh 5, Münchwilen 4333, Switzerland; ∥ 89387 Syngenta Crop Protection, Jealotts Hill, Bracknell RG42 6EY, U.K.

**Keywords:** coumarins, C−H activation, *in operando* IR, site selectivity, manganacycles, microED

## Abstract

Mn-catalyzed C–H bond functionalization offers great potential in the synthesis of highly functionalized aromatic and heteroaromatic compounds, involving the intermediacy of manganacycles and their interception through reactions with appropriate acceptor compounds. The coumarin motif is widely embedded in an eclectic array of compounds possessing interesting biological and photophysical properties. In this study, we examined the effect of changing the position of a manganese 2-pyridyl-directing group from C3 to C4 on a coumarin moiety, directing C4–H functionalization or C3–H functionalization, respectively. The reactions of cyclomanganated intermediates with phenylacetylene have been studied using time-resolved infrared spectroscopic methods on a pico- to microsecond time scale, unraveling the bond-forming processes that underpin the differing site C–H bond functionalizations. The findings facilitated the development of a catalytic methodology for the C3-alkenylation of a 4-(2-pyridyl)­coumarin using phenylacetylene.

## Graphical abstract

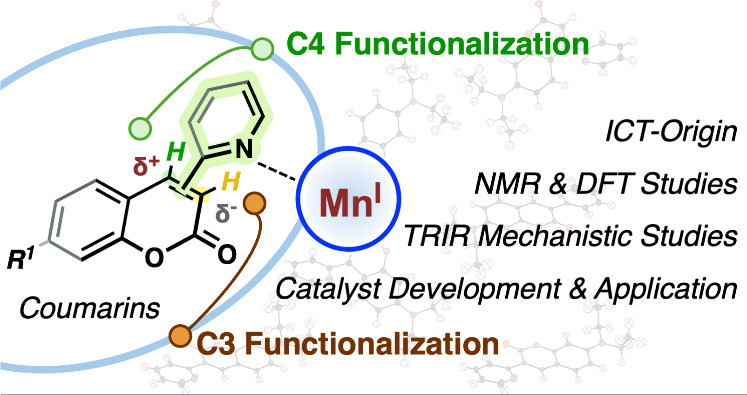

## Introduction

Site-selective C–H bond functionalizations play a key role in modern chemical synthesis.^1^ The highest priority in C–H functionalization chemistry is the direct activation and functionalization of specific C–H bonds on substrates containing many different C–H bonds. This is often difficult when multiple sites on the substrate possess similar bond dissociation energies. Attempts to control this regioselectivity issue have readily been achieved on homocyclic substrates through the use of judicial positioning of metal-directing groups including steric and/or electronically deactivating functionalities. As is the case with the dubbed “*ortho-*fluorine effect” through location of a fluoro-handle on the fragment of the molecule for C–H functionalization under modified conditions, an *ortho* or *para* C–H functionalized product could be formed.^2,3^ Coumarins are important heterocycles with a wide span of applications across pharmaceutical agents, functional materials, photo-optical dyes, and natural products.^4–6^ The regioselective C–H functionalization of coumarins carries advantages in terms of the direct transformation of more complex synthetic targets.^5^ Various privileged heteroaromatic systems have been functionalized through exploitation of the differing intrinsic electronic structure at various C–H sites.^7^ What makes the coumarin unique is the two potential sites for metal-directed C–H functionalization at C3–H and C4–H. The former site is nucleophilic, and the latter site is electrophilic, which in principle enables selective functionalization processes accessible through exploiting this intrinsic reactivity. The functionalization of coumarin derivatives to afford extended alkenylated products has garnered recent attention, including compounds with a distinct chromo-fluorogenic behavior in cyanide sensing and live-cell bioimaging.^8,9^ Furthermore, other applications are seen with powerful fluorescent dyes and bioactive monoamine oxidase inhibitors.^10,11^

Previously, we synthesized a library of 3-(2-pyridyl)­coumarin compounds (**1**), which underwent stoichiometric C–H functionalization with alkynes to afford annulated hybrid coumarin-pyridinium salts in a highly regioselective manner.^12^ It was found that the functional group (R^1^) on the cyclomanganated coumarin (**2**) influenced the propensity to undergo a reductive elimination pathway to afford (**3**) (see “Previous C4 Work”, Scheme 1). Some functional group deactivation was noted, which could be circumvented with the aid of a CO-extrusion agent (namely, trimethylamine *N*-oxide (TMNO)).^13^ With the effect of the (R^1^) substituent being so pronounced when functionalization was directed to the C4 position, we envisioned exploiting the coumarin’s inherent internal charge transfer (ICT) to enhance the nucleophilicity at the C3 position. This may in turn modulate the relative driving force for reductive elimination coupling when compared to a catalytic alkenylation pathway (see “This C3 Work”, Scheme 1). In this study, we have therefore examined 4-(2-pyridyl)­coumarin compounds (**5**), which can undergo both reductive elimination to give (**7**) stoichiometrically and alkenylation to give (**7**′****) catalytically. The latter finding could not be accessed for the 3-(2-pyridyl)­coumarin compounds (**1**). Time-resolved infrared spectroscopy (TRIR)^14^ has been used to examine the key steps involved in either reductive elimination or alkenylation. The results illustrate the delicate interplay between the electronic perturbations and the position of 2-pyridyl directing groups (for manganese) through comparisons between the structural isomers of 3- and 4-(2-pyridyl)­coumarin compounds (**1** and **5**, respectively).

**Scheme 1:**
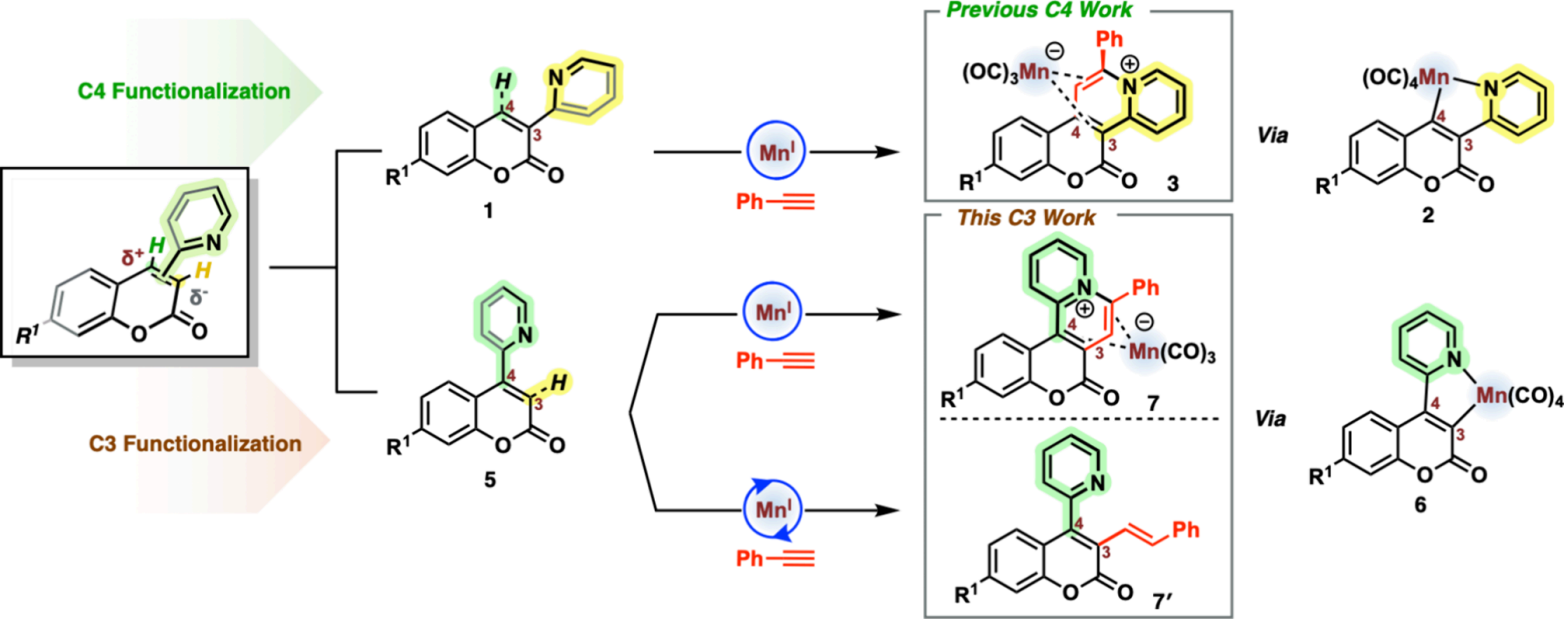
Previous C4 Stoichiometric Mn­(I) Functionalization of 3-(2-Pyridyl)­coumarins (Top) and Current C3 Stoichiometric Mn­(I) Functionalization of 4-(2-Pyridyl)­coumarins including Catalytic Hydroarylation Work

A property intrinsic to coumarins and their related derivatives is their photoemissive properties,^15^ e.g., high quantum yields and large Stokes shifts associated with the observed electronic transitions.^5^ This behavior arises as a result of inherent ICT that is often referred to as “push-pull” systems within such heteroaromatic systems. The magnitude of this effect can be readily tuned through the positioning of the “donor” and “acceptor” groups.^16–18^ The charge transfer process occurs from the donor to the acceptor in the photoexcited state of the molecule due to light absorption.^19^ Many studies have demonstrated ICT behavior and its macroscopic effect in coumarin systems, including changes in absorption and emission, through structural perturbation and distortion of the atomic bond lengths.^20^ This latter phenomenon is a result of higher (*para*)-quinoidal contribution through “donor” electron-donating substituents at the C7-position of coumarins, exacerbating the effect when used in conjugation with “acceptor” electron-withdrawing substituents at the C3- or C4-positions. This effect is depicted in (Figure 1).

**Figure 1:**
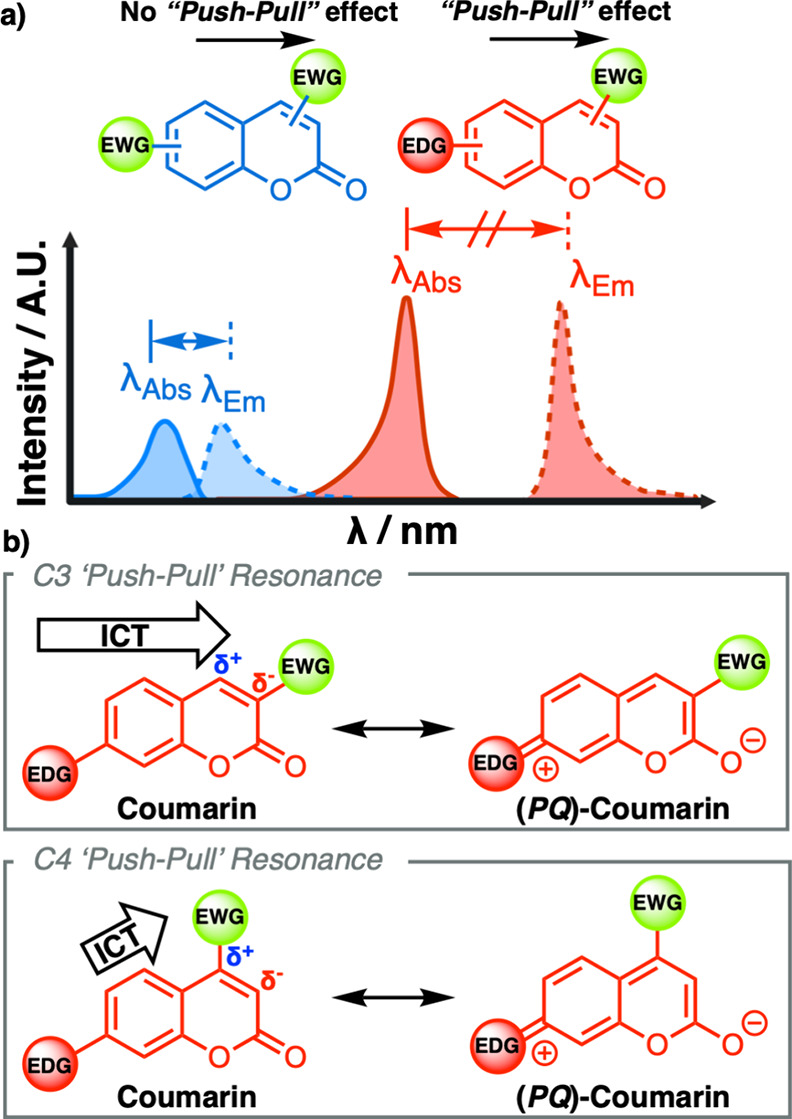
(a) Schematic UV–vis spectrum of the emission and absorbance spectrum behavior for different types of “push–pull” substituted coumarin systems demonstrating effect on peak-to-peak separation and intensity. (b) Inset, “push–pull” resonance of coumarins between neutral and (*para*)-quinoidal (*PQ*) coumarin forms. The block arrow denotes the direction of ICT.

The goals of our study are as follows:

1. Investigate the behavior of 4-(2-pyridyl)­coumarins (**5**) in both stoichiometric and catalytic C–H functionalization reactions.
2. Assess how push–pull systems influence the reactivity and properties of cyclomanganated 3- and 4-(2-pyridyl)­coumarins (**2**) and (**6**).
3. Use TRIR to identify short-lived species and track the outcomes of C–C bond formation processes in cyclomanganated 3- and 4-(2-pyridyl)­coumarins.
4. Provide evidence for the distinct reactivity observed in 4-(2-pyridyl)­coumarins (**5**) compared to 3-(2-pyridyl)­coumarins (**1**).

## Results and Discussion

We initiated our search for the origin of the observed reactivity trends of cyclomanganated 3-(2-pyridyl)­coumarin compounds (**2**) through their differing degrees of conversion into the respective reductive elimination compounds (**3**) under thermal regimes (Et_2_O, 80 °C, 18 h). Through a scope of C7-substituted analogues (Scheme 2), such compounds (**1a**–**g**) were readily synthesized via Knoevenagel condensations of 2-pyridylacetonitrile with various substituted salicylaldehydes.^12,21^ We found that in the absence of an external CO-extrusion agent (e.g., TMNO), coumarins with electron-donating substituents outperformed those without in this annulation reaction. Interestingly, 7-amino-substituted 3-(2-pyridyl)­coumarins were vastly superior even compared to 7-methyl or 7-methoxy analogues despite the latter still being considered electron-donating functionalities. With this information in hand, we studied if the photoinduced CO-loss methodology also delivered such trends. Contrarily, under the optimized photoinduced conditions (rt, *hv* = 355 nm, 2 h), all tested cyclomanganated 3-(2-pyridyl)­coumarin compounds (**2**) completely converted into the respective reductive elimination compounds (**3**) when reacted with phenylacetylene. The comparative studies are shown in (Table 1).

**Scheme 2:**
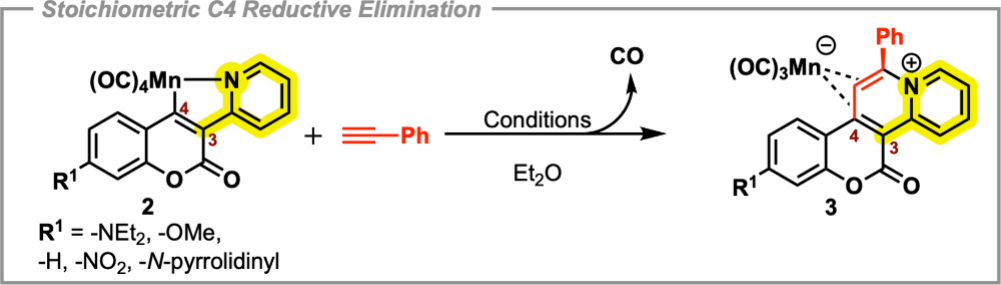
Stoichiometric Thermal or Photoinduced C4 Reductive Elimination Reaction of Derivatives of **2** with Phenylacetylene to Give Complexes of **3**

**Table 1:**
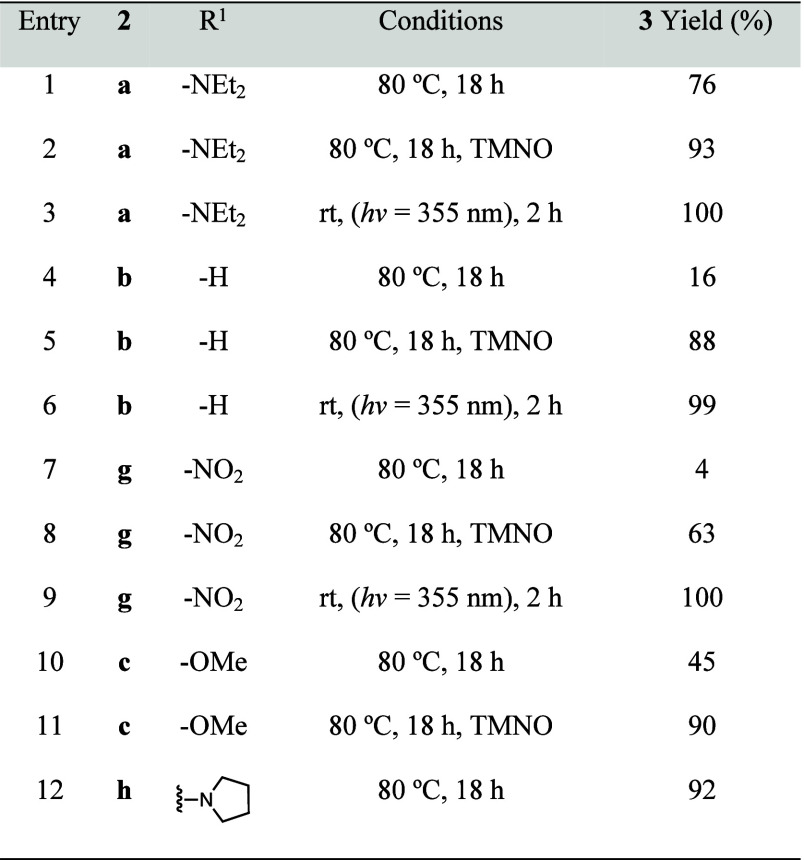
Varied Conditions to Afford Reductive Elimination Reactions under Thermal and Photochemical Methods

Conversely, the C4-(2-pyridyl)­coumarin structural isomers (**5**) exhibited no problematic reactivity. These compounds prepared through Pechmann condensation reactions of substituted phenols were then tested under similar reactions with alkynes and delivered the respective reductive elimination compound (**7c**) in excellent yields under thermal and photochemical conditions. The X-ray crystal structure of (**7c**) shown in (Figure 2) is consistent with previous structures of reductive elimination compounds (**3**) where the fused pyridinium framework exhibits a highly distorted geometry.^22^ It has long been observed in single-crystal X-ray structures of coumarin dyes that these bond shortening and modification are due to photoemissive properties.

**Figure 2:**
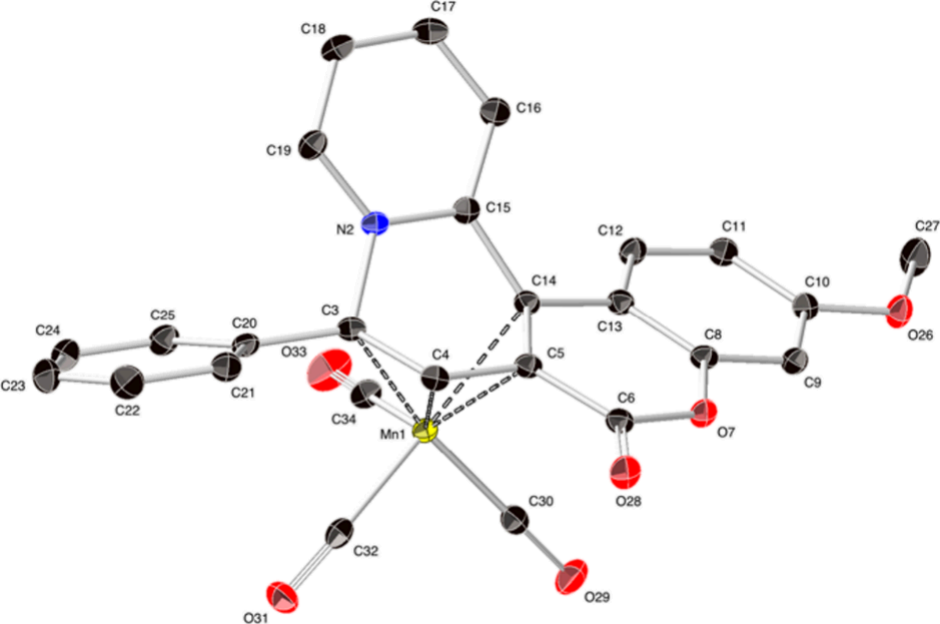
X-ray diffraction (XRD) structure of (**7c**) (ellipsoids set at 50% probability; H atoms omitted).

Herein, we demonstrate the first example of this behavior in cyclometalated manganese 3- and 4-(2-pyridyl)­coumarin compounds (Figure 3 and Table S6) by both single-crystal X-ray diffraction data and microcrystal electron diffraction (microED) data. The free ligand compound (**1a**) exhibits a greater degree of bond elongation and shortening compared to compound (**1b**). This difference again can be directly compared to the effect an electron-donating “donor” C7 substituent possesses on the “push–pull” dynamics of the coumarin system.^17^ Most noticeably, this results in the shortening of the N1–C7 bond (1.3646(32) Å) in (**1a**) due to extended π-conjugation. Instead of observing the usual sp^3^-hybridized nitrogen, the torsion angles around the 7-diethylamino-nitrogen are closer to the sp^2^-center at χ_Ν_ 7.07°. This behavior is credited to the greater (*para*)-quinoidal contribution previously presented in Figure 1. The trend also extends to cyclomanganated analogues (**2c** and **6c**).

**Figure 3:**
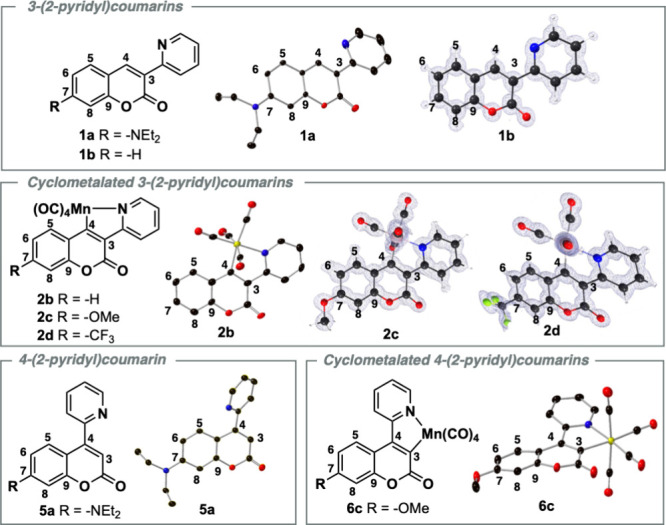
(Left) Structures of 3- and 4-(2-pyridyl)­coumarins **1a**, **1b**, and **5a**; metalated 3-(2-pyridyl)­coumarins **2b**,^12^ **2c**, and **2d**; and metalated 4-(2-pyridyl)­coumarin **6c**, along with conventional atom numbering. (Right) X-ray crystal structures (thermal ellipsoids modeled at 50% probability, hydrogen atoms not shown for clarity) and microED structures (ball and stick model, Fourier map shown in gray).

Conversely, the C5–C6 and C8–C9 bonds of the coumarin derivatives are noticeably shorter than the equivalent C6–C7 and C7–C8 bonds within the same framework (see Supporting Information, Table S6). With the aid of EDG substituents, the (*para*)-quinoidal contribution further enhances this effect (**1a**, **2c**, **5a**, and **6c**). The phenomenon is again observed throughout cyclomanganated coumarins with electron-donating substituents and reveals close to negligible elongation when electron-withdrawing substituents (−CF_3_) are appended at the C7 position (**2d**). Between the two structural isomers of cyclomanganated coumarins (**2c** and **6c**), there is a more prominent bond length change in the 3-(2-pyridyl)­coumarin (**2c**). This is thought to arise from positioning of the electron-withdrawing 2-pyridyl-group that when attached at the C3 position gives the greatest “push–pull” effect compared to when placed at C4.^16^ This effect can also be observed in the electronic spectra of these cyclomanganated compounds. When studied in dichloromethane, complexes with enhanced “push–pull” ICT possess larger molar absorption coefficients along with red-shifting of λ_max_, with (**2c** and **2d**) exhibiting λ_max_ = 349 and 338 nm, respectively. The red-shifting in absorption in a coumarin containing an electron-donating group (**2c**) arises due to reduced energy gaps in the HOMO–LUMO transition. The electron-donating group raises the energy of the HOMO, and the electron-withdrawing group lowers the LUMO energy^20^ (electronic spectra λ_max_ and molar absorption coefficients of compounds shown in SI, Table S1).

### 2-Pyridyl Positional Changes in Coumarin

The positions on the coumarin ring system, frameworks even without the aid of directing groups or ICT inducing functionalizations, are either activated or deactivated in site-selective C–H bond functionalization. The coumarin C3–H position is more electron-rich at carbon; this facilitates functionalization through electrophilic palladation.^23^ This stands in contrast to C4–H, which is electron-poor at carbon, requiring alternative electrophiles.^12^ This can be observed experimentally between the two structural regioisomers of 3- and 4-(2-pyridyl)­coumarin compounds (**1c** and **5c**). When the 2-pyridyl-group is located on the coumarin C3 position (**1c**), the resonance for the C4–H proton is observed at δ 8.79 ppm in the ^1^H NMR spectrum. In (**5c**), when the 2-pyridyl-group is located on the coumarin C4 position, the C3–H resonance appears at δ 6.38 ppm.^12^ The significance of the positioning and its relationship to electronic effects can also be observed through the changes in the resonance of the associated C4–H chemical shift with different C7 substituents. These results are presented as a Hammett plot in Figure 4, where there is a noticeable correlation across a library of C7-substituted 3-(2-pyridyl)­coumarins for the C4–H chemical shift vs the functional group’s σ^+^ or σ^–^ parameter. It can be seen that as more electron density is donated through the C7 substituent, i.e., with (−NEt_2_, −OMe, −Me), there is greater shielding; this trend has been used to explain the enhanced nucleophilicity over the electron-withdrawing group substituted analogues. To determine if alternative substitution patterns on the 3-(2-pyridyl)­coumarins give rise to such clear trends in C4–H ^1^H NMR chemical shift, a library of analogous C6-substituted coumarins was studied. In this case, there was negligible correlation between the C6 σ_meta_ parameter vs C4–H ^1^H NMR chemical shift. This was credited to the weaker mesomeric and inductive effects due to the competitive electronic communication from the intrinsic push–pull system of the coumarin (the C6-substituted 3-(2-pyridyl)­coumarin Hammett plot is shown in SI, Figure S1).

**Figure 4:**
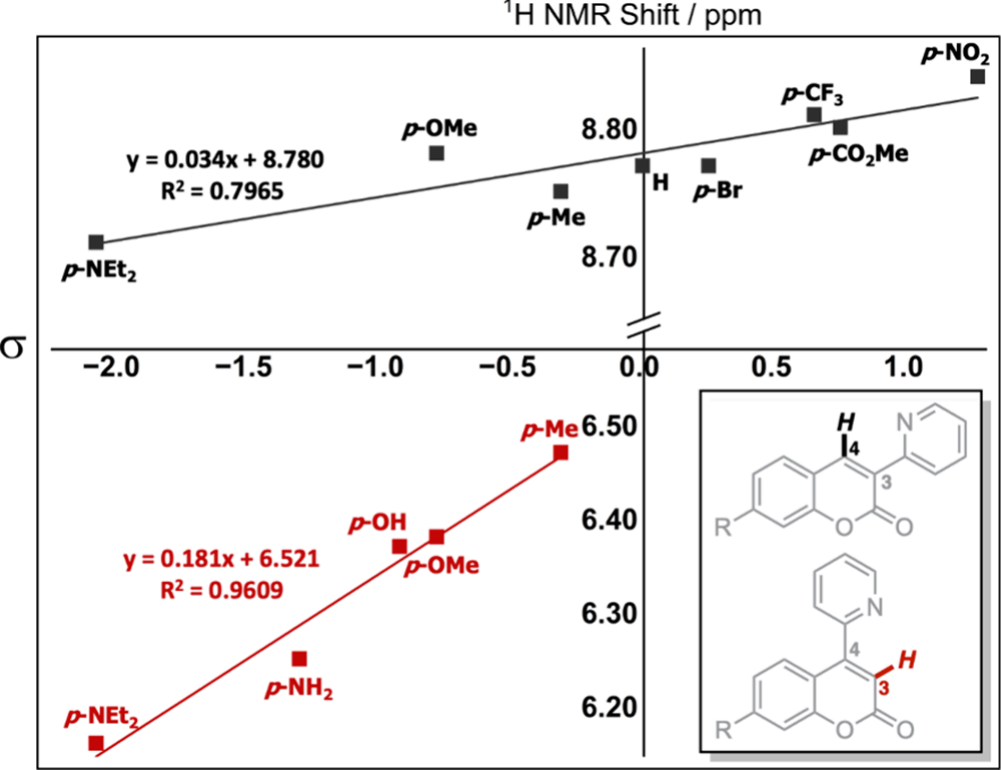
Hammett plot of σ values against coumarin C3–H and C4–H ^1^H NMR chemical shift in CDCl_3_ at 298 K (400 MHz). General structures shown in the inset, with both σ^+^ and σ^–^ plotted.

This inherent difference between the positions on representative coumarins (**1c**) and (**5c**) can also be observed through computational studies.^20,24^ DFT studies at the BP86/SVP-GD3BJ in toluene using the CPCM solvent model level of theory carried out on 7-substituted 3- and 4-(2-pyridyl)­coumarins) in the ground (S_0_) state reveal the electronic distribution of the HOMO on the framework. As shown for (**1c**) (upper-left, Figure 5) the C3 contribution (0.10) is larger than that of C4 (0.04). Even when the connectivity is changed and the 2-pyridyl group is located on the C4 position, the C3 position continues to feature a larger proportional atomic contribution (0.09). These results are also corroborated with Mulliken charges with the C3 carbon having a slight negative charge of −0.03 and −0.17, with a neutral, 0, and positive charge, 0.17, residing at the C4 position for (**1c**) and (**5c**), respectively. It is also worth noting that between both (**1c**) and (**5c**), the atomic contributions of the C7 position are equivalent, suggesting that the position contributes a similar magnitude to the ICT of the molecular structure. Isosurface maps of both coumarins (**1c**) and (**5c**) and their respective cyclomanganated derivatives again indicate the buildup of electron density around the C3 position.^25^

**Figure 5:**
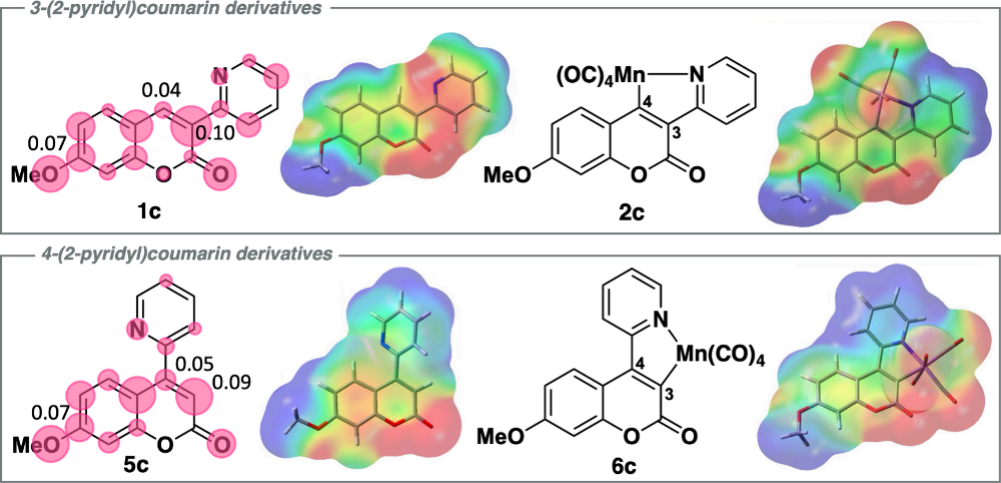
(Top) Structures of **1c** and **2c** alongside isosurface models; **1c** shown with HOMO individual atomic contributions to the electron density distributions. (Bottom) Structures of **5c** and **6c** alongside isosurface models; **6c** shown with HOMO individual atomic contributions to the electron density distributions. The sizes of the pink circles are proportional to the atomic contributions and shown only above 0.02. DFT studies at the BP86/SVP-GD3BJ in toluene using the CPCM solvent model level of theory (ESP isovalue: 0.0004).

### Substituent Effects on the Rate of Alkyne Insertion into the Mn–C Bond

Following the assessment that, across a library of synthesized 3-(2-pyridyl)­coumarins (**1a**–**g**), the varied C7-substituent resulted in changes in the effectiveness of conversion into the respective reductive elimination complex from reaction with phenylacetylene, we questioned whether the internal coumarin ICT influenced the observed trend. Previously, we assessed the conversion of a series of cyclomanganated derivatives (**2a**–**g**) to their respective reductive elimination complexes (**3a**–**g**). The study highlighted the beneficial nature of electron-donating groups on the coumarin framework in facilitating this chemistry. To assess how the substituents on the coumarin affected the kinetics of this reaction, the insertion of the alkyne into the Mn–C bond was studied for the cyclomanganated intermediates through the respective reductive elimination complexes.

### Time-Resolved Multiple Probe Spectroscopy Monitoring of Alkyne Insertion

As concluded previously, most issues with functional group deactivation on 3-(2-pyridyl)­coumarins (**2a**–**g**) were circumvented through the application of photoinduced CO-loss methodologies. With this insight, it was possible to explore the fundamental bond breaking–forming processes of the cyclomanganated 3- and 4-(2-pyridyl)­coumarins using time-resolved infrared (TRIR) spectroscopy. It was anticipated that photolysis of these compounds would result in ultrafast CO loss,^26^ creating a vacant coordination site at the metal. If the experiment was performed in neat PhC_2_H, then the alkyne would bind to the metal, and the changes in the vibrational modes of the remaining CO ligands would provide insight into the nature and dynamics of the resulting compounds. The photochemical excitation results in CO dissociation generating a single vacant coordination site at the metal, mimicking the precatalyst activation pathways that occur under thermal conditions.^27,35^

The TRIR experiments were performed using the time-resolved multiple probe spectroscopy TR^M^PS method.^14^ Initial excitation of the complexes was performed with a 355 nm pulse that was followed by a set time interval with a probe pulse in the mid-IR that enabled the evolution of species formed upon photoinduced CO loss to be observed. Through synchronization of the pump and probe lasers, TR^M^PS enables experiments utilizing pump–probe delays between 1 ps and 1 ms to be studied. IR detection over the range of 1850–2100 cm^–1^ was used so that the detection and processes associated with (**I**) and (**II**) could be observed. Resulting data are presented as difference spectra; positive peaks equate to the generated photoproducts, and negative peaks belong to consumed starting complexes.

Photolysis of cyclomanganated 3- and 4-(2-pyridyl)­coumarins (**2a**–**g**) and (**6c**) was performed in neat phenylacetylene. This was to prevent competitive stochastic solvation with excess solvent molecules.^26^ In all cases, strong negative bands were observed at all positive pump–probe delays employed, demonstrating that the complexes had been consumed. Three new transient absorptions were observed in the spectra, demonstrating that light-induced CO loss had occurred from the metal and the resulting complexes had a *fac-*[Mn­(C^N)­(CO)_3_(alkyne)] geometry.^26^ By analogy to previous data, these positive peaks were assigned to a species in which the alkyne binds to the manganese via an arene-bound intermediate (**III** **_Alkyne_**),^27^ which then isomerizes to give a π-bound form (**I**) (Scheme 3). This process was then followed by the slower insertion of the coordinated alkyne to give intermediate (**II**), a seven-membered manganacycle. Analysis of the data provided observed rate constants (*k*_1_ and *k*_2_) for both steps: the associated IR νCO peaks for the intermediates are collated (Table 2). Therefore, the light-induced CO loss had enabled alkyne coordination so that the subsequent migratory insertion reaction could be directly observed.^27,29^

**Scheme 3:**
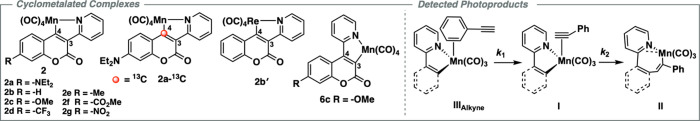
Structures of Cyclomanganated Compounds Studied with TRIR and Subsequent Detected Species and Kinetic Processes

**Table 2:** Table of TRIR Phenylacetylene Insertion Data including Ground-State Frequencies Recorded in Phenylacetylene (Complex ν_(CO)_), Frequencies of Detected Intermediates, and Associated Measured Observed Rate Constants

complex	alkyne	complex ν_(CO)_/cm^–1^	**I** ν_(CO)_/cm^–1^	**II** ν_(CO)_/cm^–1^	*k*_1_/10^10^ s^–1^	*k*_2_/10^5^ s^–1^
**2a**	PhC_2_H	1936, 1981, 2003, 2081	1904, 1950, 2016	1905, 1912, 2012	(5.83 ± 0.44)	(5.62 ± 1.91)
**2b**	PhC_2_H	1939, 1986, 2005, 2083	1909, 1951, 2020	1910, 1929, 2017	(5.92 ± 0.22)	(11.4 ± 1.7)
**2c**	PhC_2_H	1939, 1985, 2005, 2083	1909, 1951, 2020	1909, 1922, 2017	(5.01 ± 0.37)	(13.2 ± 2.9)
**2d**	PhC_2_H	1944, 1988, 2008, 2085	1914, 1955, 2021	1921, 1933, 2019	(5.83 ± 0.39)	(9.43 ± 2.51)
**2e**	PhC_2_H	1940, 1986, 2005, 2083	1911, 1951, 2026	1912, 1925, 2023	(5.15 ± 0.34)	(14.9 ± 2.5)
**2f**	PhC_2_H	1945, 1990, 2009, 2086	1915, 1956, 2021	1921, 1933, 2018	(4.56 ± 0.60)	(18.5 ± 7.7)
**2g**	PhC_2_H	1943, 1989, 2008, 2085	1918, 1959, 2022	1927, 1967, 2020	(5.71 ± 0.90)	^b^
**6c**	PhC_2_H	1949, 1998,^a^ 2081	1905, 1959, 2023	1909, 1933, 2016	^c^	(38.6 ± 11.4)
**2b**′****	PhC_2_H	1936, 1979, 2002, 2095				
**2a**	4-F_3_C-C_6_H_4_C_2_H	1940, 1985, 2005, 2083	1909, 1954, 2020	1908, 1930, 2016	^d^	(30.0 ± 7.4)
**2a**-^13^C	PhC_2_H	1935, 1981, 2002, 2081	1903, 1946, 2017	1900, 1920, 2012	(5.05 ± 0.71)	(9.99 ± 2.51)

a Likely two bands of similar frequency when recorded in phenylacetylene.

b The longer time data for **2g** were of limited signal quality to determine *k*_2_.

c **III** **_alkyne_** and **I** exhibited two bands of similar frequency when recorded in phenylacetylene, and *k*_1_ could not be determined.

d An additional intermediate species was observed to form between **III** **_alkyne_** and **I**. This was tentatively assigned to CF_3_ coordination to the manganese (see the SI for further details).

In all cyclomanganated 3-(2-pyridyl)­coumarins (**2a**–**g**), the initially formed photoproduct (**III** **_Alkyne_**) corresponding to the π-arene bound complex was detected, suggesting a common manifold of reactivity regardless of the functional group. Although short-lived (ps) bands attributable to the formation of a ^3^MLCT state of complexes **2** were observed,^12,26,28^ the rearrangements and bond-forming events corresponding to the **III** **_Alkyne_** *→* **I** *→* **II** pathway occur on the ground-state electronic surface.^26^ The rate constant of the formation of this species isomerizing to the π-bound form (**I**), *k*_1_, was consistently observed at ca. 5.6 × 10^10^ s^–1^. Generally, once in the π-bound complex (**I**), the insertion of the alkyne is observed to take place giving proposed seven-membered manganacycle species (**II**). Evidence for such species is observed across all cyclomanganated 3- and 4-(2-pyridyl)­coumarin derivatives where upon insertion, the bands belonging to (**II**) ν­(CO) are red-shifted to lower frequencies.^29^ This shift in vibrational frequency is thought to arise from the loss of a suitable π-acceptor ligand from the coordination sphere of the manganese. The observed rate constant for this process, *k*_2_, does vary with the general observation that metallocycles with electron-withdrawing functional groups exhibit slower rates of alkyne insertion. This trend is consistent with analogous observations with isoelectronic manganese systems where modifications to the alkyne (electrophile), i.e., when made more electrophilic (attached to an electron-withdrawing substituent), increased the rate of alkyne insertion.^29^ To confirm if this analogous observation was again consistent, the alkyne 4-F_3_C-C_6_H_4_C_2_H was applied, and this was accompanied by nearly a 6× increase in *k*_2_ compared to the standard PhC_2_H. When the metal at the center of the complex was exchanged for rhenium, this cyclorhenated complex (**2b′**) demonstrated no CO loss or alkyne coordination behavior, consistent with previous findings when the 5d metal was applied toward alkyne insertions.^12^

The difference in the fates of the 3-(2-pyridyl)­coumarin and 4-(2-pyridyl)­coumarin isomers was probed using TRIR spectroscopy. As mentioned previously, it was observed that compound (**6c**) underwent alkyne isomerization at a much slower observed rate than (**2c**). However, in the following reaction step, the observed rate for *k*_2_ involving alkyne insertion was ca. 4× faster. This is consistent with the observation that the C3-position on the coumarin is much more nucleophilic than the C4-position; again, this can be observed in the difference in ^1^H NMR chemical shift of the C4 position. With (**5c**), the C4–H position is largely shielded at δ = 6.38 ppm; this is supported by the DFT calculations of the HOMO of the free coumarin. To verify whether the finding for compound (**2a**) was consistent, specifically that despite having an electron-donating C7 substituent, the insertion rate *k*_2_ was slower than that of other conventional electron-donating substituents, an isotopologue in which the C4 position was enriched with ^13^C was studied. Under identical experimental conditions, this gave a near identical rate constant of *k*_2_ = (9.99 ± 2.51) × 10^5^ s^–1^ compared to (5.62 ± 1.91) × 10^5^ s^–1^ for the naturally abundant complex (**2a**). This both confirms this anomalously slow insertion rate and illustrates no clear ^13^C/^12^C PKIE during this study.^30^

**Figure 6:**
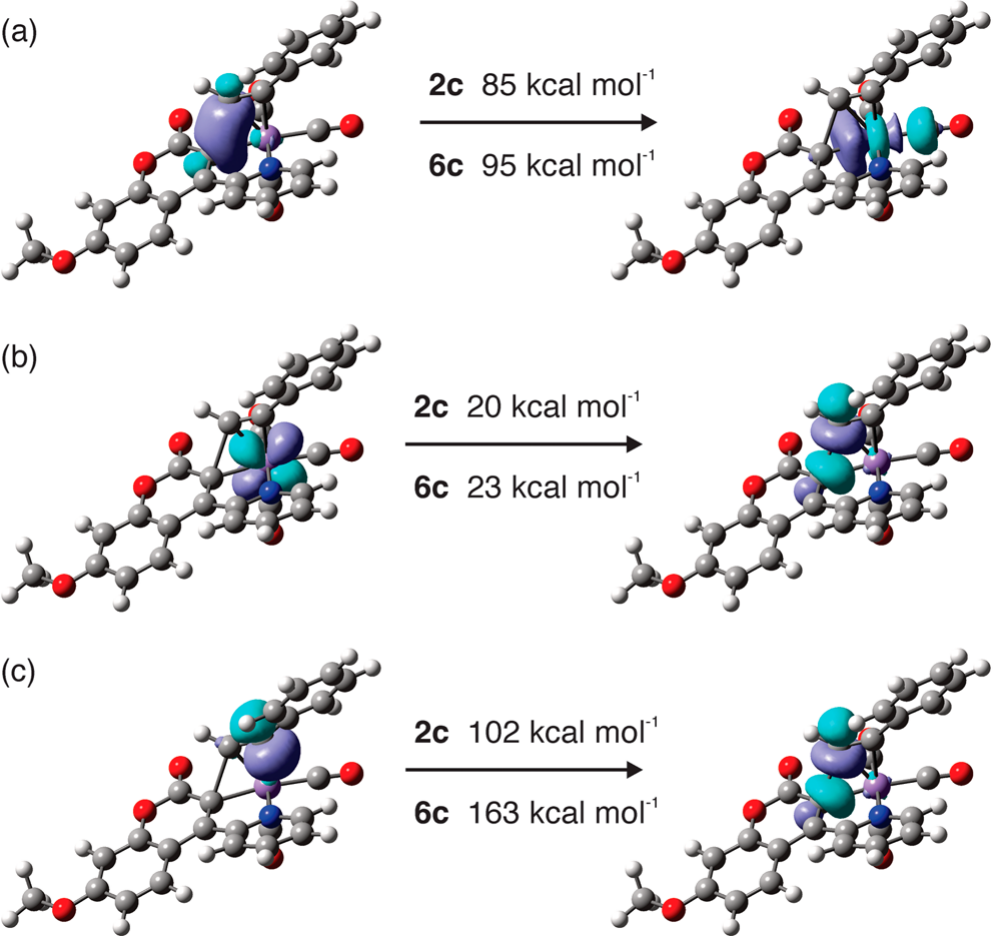
Selected donor–acceptor interactions in **TS**_6cPhCCH_. The energies given are the second-order perturbation energies associated with each interaction. Cube files for visualizing the NBOs were generated using Multiwfn.^32^

**Figure 7:**
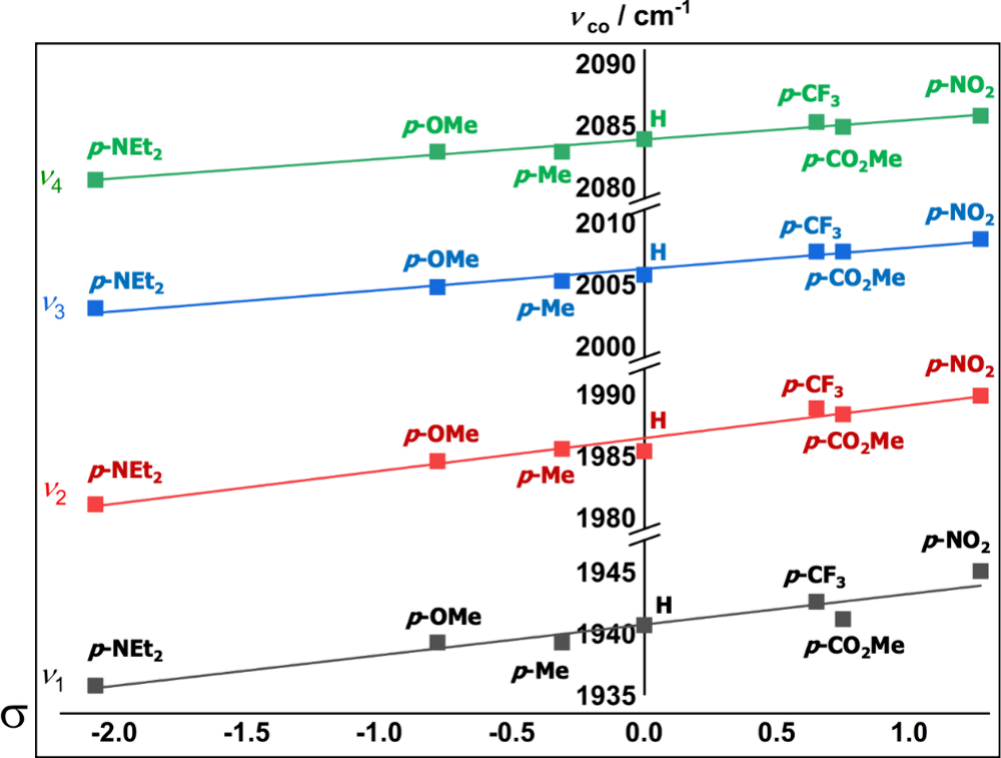
Hammett plot of Hammett “σ” values against cyclomanganated 3-(2-pyridyl)­coumarins’ (**2a**–**g**) metal carbonyl stretching frequencies in phenylacetylene; gradient showing correlation between both σ^+^ and σ^–^ plotted values and frequency of metal carbonyl stretches. Gradients for ν_1_, ν_2_, ν_3_, and ν_4_: 2.49, 2.67, 1.73, and 1.58 respectively.

Examination of the rate constants (*k*_2_) for the migratory insertion of the PhC_2_H into the Mn–C bond of the coumarin groups in complexes **2** (Table 2) demonstrates that there is only a small effect of different substituents on the coumarin framework. However, the site of metalation of coumarin has a much larger effect. Comparing the value of *k*_2_ for the coumarins metalated at the 4-position (**2a**–**g**) with that at the 3-position (**6c**) demonstrates that the latter undergoes the migratory insertion reaction ca. 4 times faster than the former. This effect is also represented in the DFT calculations (Figure 8) on this system, which demonstrate that the free energy of activation for the migratory insertion through **TS** **_2cPhCCH_** is 30 kJ mol^–1^ with respect to the η^2^-alkyne complex **2c** **_PhCCH_**, whereas for the (**6c**) system, the corresponding barrier is +19 kJ mol^–1^.

**Figure 8:**
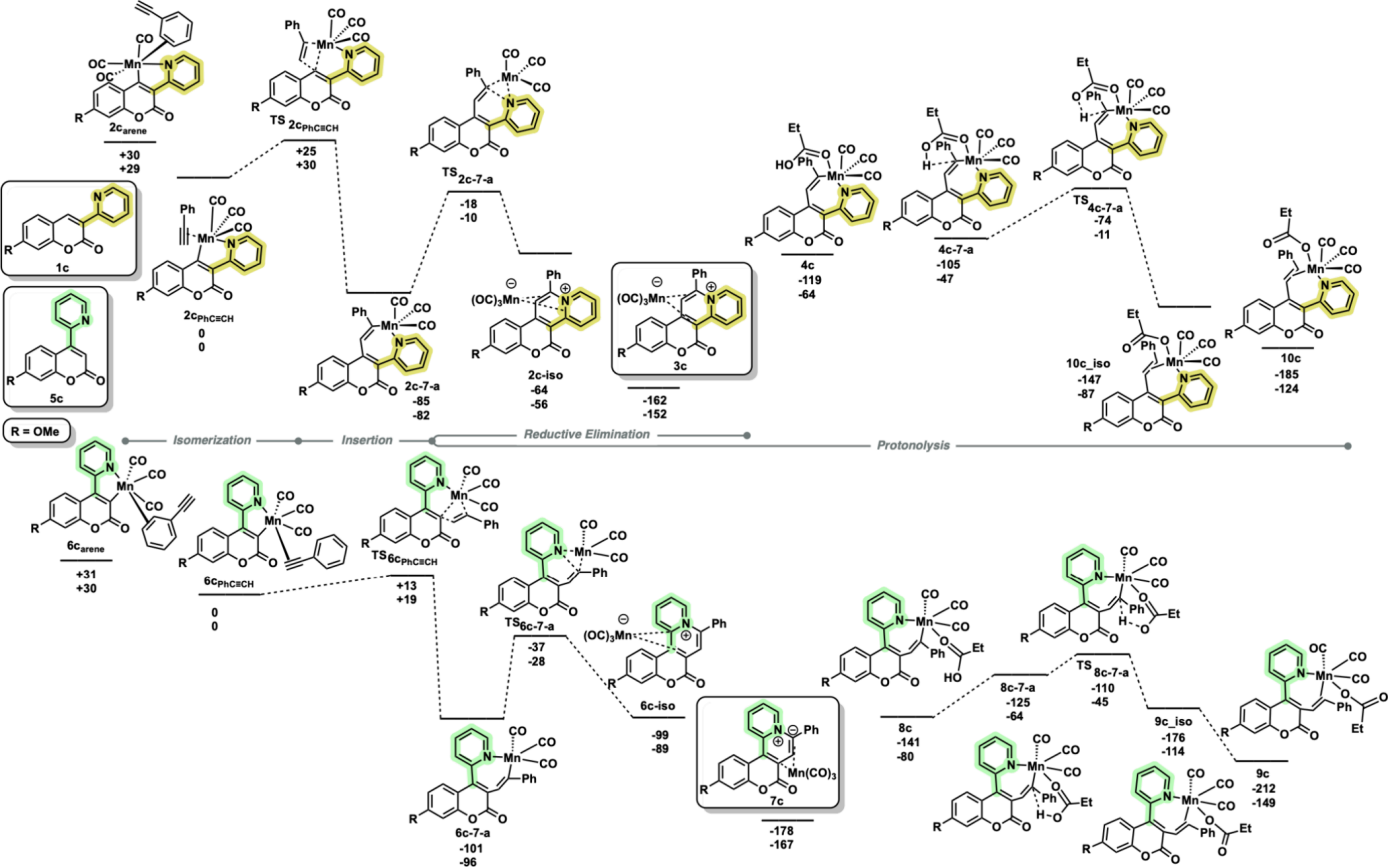
DFT calculations showing pathways of **1c** and **5c** to respective reductive elimination complexes **3c** and **7c** compared to pathways to catalytic alkenylation products **9c** and **10c** from intermediates **8c** and **4c**, respectively. Calculations were performed at the pbe0/def2-TZVPP//bp86/SV­(P) level of theory, with a COSMO implicit solvent model (toluene) and Grimme’s third empirical dispersion correction. Energies are zero-point energy-corrected electronic energies (top) and Gibbs energies at 298.15 K (bottom) in kJ mol^–1^, where R = OMe.

Our previous work on the nature of the migratory insertion of alkynes into Mn–C bonds^24^ has indicated that the major factor controlling the rate of the migratory insertion reaction is the donation and back-donation σ- and σ*-orbitals of the nascent C–C bond with orbitals based on the Mn and the alkyne. These interactions were quantified through the natural bond orbital (NBO) approach^31^ as described previously, which demonstrated that the same key interactions were present in this system as calculated previously.^24^ Specifically, the second-order perturbation analysis reveals that there is metal assistance in the formation of the new C–C bond. This involves donation from the nascent C–C σ-bonding orbital to a vacant metal-based orbital (Figure 6a) and back-donation from a filled metal orbital to the corresponding C–C σ* antibonding orbital (Figure 6b). Donation from an alkyne-based orbital to the C–C σ* orbital is also observed (Figure 6c).

The previous data had demonstrated that the greater the extent of these interactions is, the greater is the value of *k*_2_. This trend is repeated here. First, in comparison to selected values from previous studies,^29^ then the corresponding values for (**2c**) and (**6c**) fit within the global trend (Figure 6). The only substantial difference between (**2c**) and (**6c**) though is the interaction in Figure 6c. In line with the underlying hypothesis, this interaction is greater in the case of (**6c**), which undergoes the migratory insertion reaction faster than that of (**2c**). Therefore, the difference in the site of metalation in the coumarin can affect the rate of migratory insertion.

Evaluation of the ground-state metal carbonyl frequencies of complexes (**2a**–**g**) in neat phenylacetylene together highlighted the contribution and through-bond interaction of the C7-substituent on electron donation toward the metal carbonyl fragment. As shown in Figure 7, there is a relatively consistent linear trend across all four metal carbonyl frequencies, decreasing to lower wavenumbers when an electron-donating group is present on the C7 position. This relationship indicates the additional electron density as a result of the ICT that through the electrophilic C4 position is pushed onto the metal, which in turn leads to greater π-backbonding, weakening the C≡O bond.

### Reaction Order Determination of **5c**

To further understand the difference in reactivity of the cyclomanganated 4-(2-pyridyl)­coumarin and the 3-(2-pyridyl)­coumarin analogues, TRIR experiments were performed in a toluene solution of PhC_2_H. Using (**6c**) as an example, after photolysis in neat phenylacetylene to diluted conditions in toluene solutions, the changes in the TRIR spectra were studied to elucidate the molecularity and reaction order. Once in diluted toluene solutions, an additional intermediate was detected upon photoinduced CO loss. Species **III**_Toluene_ was assigned to an arene-bound toluene Mn complex; the toluene ligand is then displaced by phenylacetylene, allowing the migratory insertion reaction to take place. To confirm the identity of the structure of **III**_Toluene_ (Figure 9), an identical photolysis reaction in the absence of phenylacetylene was performed, yielding the same intermediate with positive peaks at 1909, 1936, and 2021 cm^–1^. In the presence of phenylacetylene, the initial binding of alkyne to give **III**_Toluene_ occurs under kinetic control.

**Figure 9:**
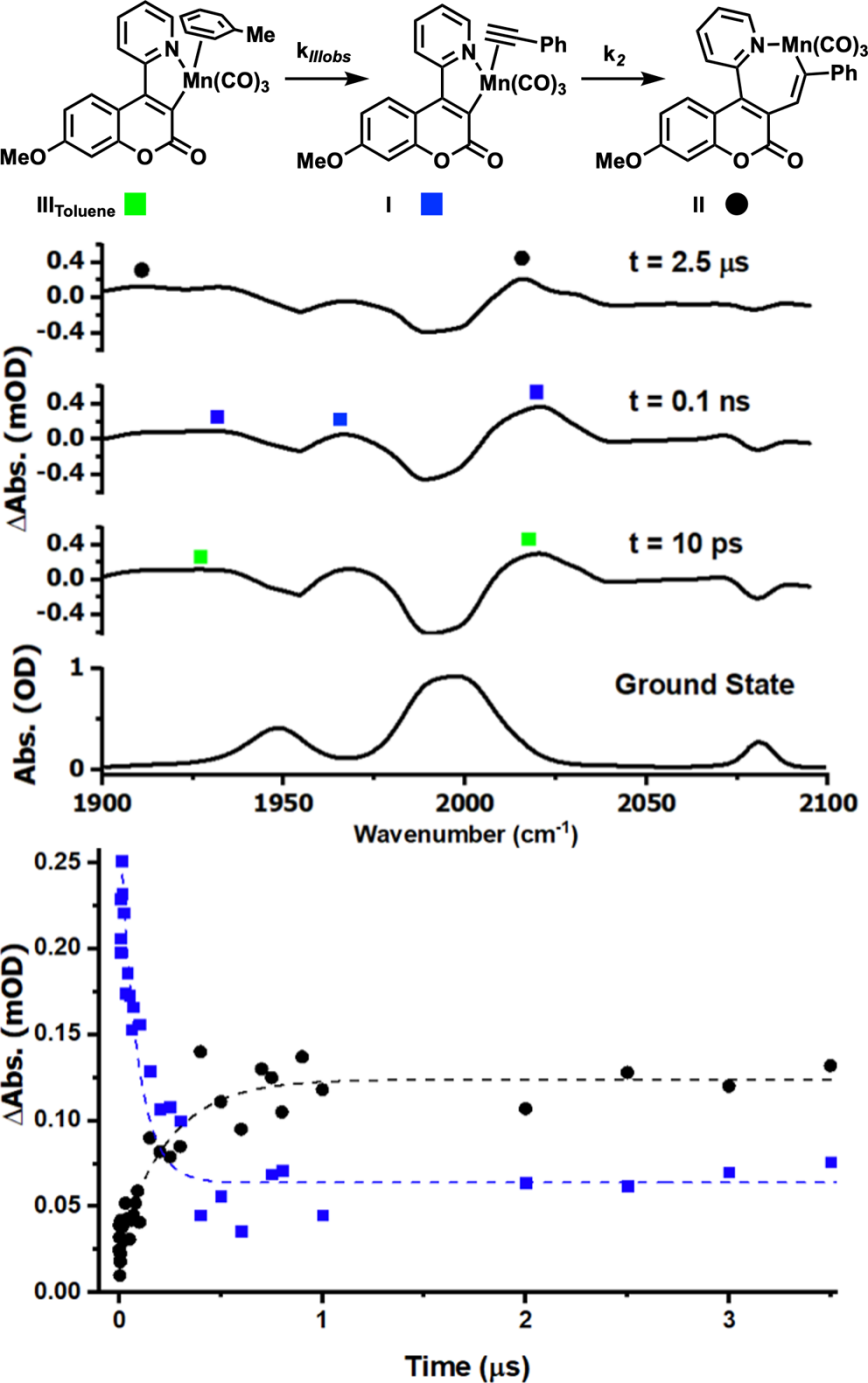
(Top) Reaction scheme of intermediates detected in TRIR of **6c** in the presence of phenylacetylene in toluene. (Middle) TRIR spectra recorded with pump–probe delays of 10 ps, 0.1 ns, and 2.5 μs. (Bottom) Kinetic plot loss of species **I** (blue square)­and gain of **II** (black circles) over time.

Varying the concentration of phenylacetylene facilitated the analysis of the kinetic steps. Under pseudo-first-order conditions, the observed first-order rate constants (*k*_IIIobs_ and *k*_2_) could be approximated from the fits of exponential growth and decay models of associated transient signals in the TRIR data as a function of alkyne concentration. That is the transformation of (**6c**) to (**III**) on to (**I**) and then (**II**). Plotting *k*_IIIobs_ versus alkyne concentration [PhC_2_H] (Figure 10a) gave a gradient equating to the second-order rate constant for the substitution of (**III**) to (**I**). This was observed to be (5.31 ± 0.91) × 10^7^ mol dm^3^ s^–1^. Further analysis (SI, Figure S7) plotting ln *k*_IIIobs_ versus ln­[PhC_2_H] also gave a linear fit. Under a similar study plotting *k*_2_ versus alkyne concentration [PhC_2_H], the intercept of this plot (Figure 10b) gave the first-order rate constant of (**I**) to (**II**) of (1.96 ± 0.04) × 10^6^ s^–1^. Additional analysis on this process in the form of ln *k*_2_ versus ln­[PhC_2_H] (SI, Figure S8) also gave a linear fit with a gradient of (0.16 ± 0.03), indicating zero order with respect to alkyne. This zero-order relationship for phenylacetylene in *k*_2_ supports the intramolecular nature of alkyne insertion into the Mn–C bond.

**Figure 10:**
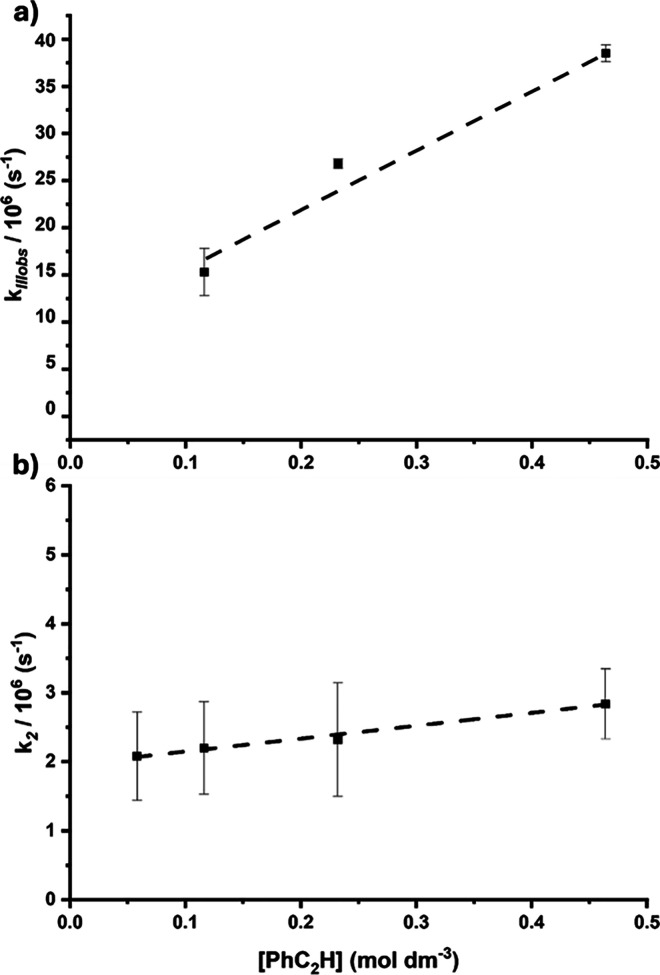
(a) Plot of *k*_IIIobs_ versus [PhC_2_H] determined from experiments of **6c** in toluene; error bars indicate 95% confidence for the rate constants. (b) Plot of *k*_2_ versus [PhC_2_H] determined from experiments of **6c** in toluene; error bars indicate 95% confidence for the rate constants.

### Catalytic C–H Bond Functionalization

Armed with the above information, we turned our attention to the catalytic C–H bond functionalization process.^33^ For the two isomeric compounds (**1c**) and (**5c**), different outcomes were observed, which may reflect the position of activation with the coumarin framework. The 3-(2-pyridyl)­coumarins (**1a**–**g**) series was found to undergo stoichiometric reductive elimination with various alkynes in a highly regioselective manner, affording products (**3a**–**g**). This transformation was exclusive, and despite trialing a range of reaction conditions, Mn­(I)-catalytic C–H alkenylation (hydroarylation) could not be achieved with these compounds (**1a**–**g**).^12^ Although 4-(2-pyridyl)­coumarin (**5c**) could be induced to undergo reductive elimination to form compound (**7**) (*vide supra*), application of catalytic C–H alkenylation conditions was successful in this case. As shown in Scheme 4, the coupling between (**5c**) delivered the π-extended styryl-coumarin compound (**7c′**). The optimization of this catalytic C–H functionalization is outlined in Table 3.

**Scheme 4:**
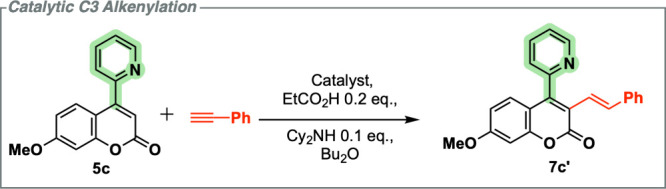
Catalytic C3-Alkenylation of 4-(2-Pyridyl)­coumarin **5c** with Phenylacetylene

**Table 3:** Optimization of Catalytic C3 Alkenylation of 4-(2-Pyridyl)­coumarins^a^

entry	catalyst (mol %)	*T*/°C	acid	yield of **7c′** (%)
1	none	100	EtCO_2_H	0
2	Mn_2_(CO)_10_ (10)	100	EtCO_2_H	0
3	MnBn(CO)_5_ (10)	100	EtCO_2_H	10
4	MnBn(CO)_5_(20)	100	EtCO_2_H	19
5	Re(CO)_5_Br (10)	100	EtCO_2_H	2
6	MnBr(NCMe)_2_(CO)_3_ (10)	35	4-F_3_C–C_6_H_4_–CO_2_H	0
**7**	**Mn(CO)**_**5**_ **Br** (10)	**100**	**EtCO**_**2**_ **H**	**31**

a Yields determined by ^1^H NMR spectroscopy.

Our optimization studies highlighted that despite manganese- and rhenium-containing complexes of (**5c**) being known, they are reluctant to undergo alkenylation with phenylacetylene, an observation in keeping with the previously reported isoelectronic 2-pyrones and pyridinone compounds.^27^ The best result can be seen in entry 7 (Table 3) using [MnBr­(CO)_5_] at 10 mol % catalyst loading, which afforded (**7c**′) in 31% yield (with TON ∼3). Even when the recently developed activated precatalyst reported by Larossa and co-workers, [MnBr­(NCMe)_2_(CO)_3_],^3^ was applied, it was unable to bring about formation of (**7c**′) at either 35 or 100 °C. Given the clear trends in thermal reductive elimination across 3- and 4-(2-pyridyl)­coumarins (**1a**–**g**) and (**5c**), with the former showing a high degree of functional group sensitivity but the latter less impacted so, a few 4-(2-pyridyl)­coumarins were explored under the hydroarylation reaction conditions. Under the optimized conditions from Table 3, entry 7, attempts to form alternative alkenylated coumarins were trialed. The catalytic reactions forming (**7a′**, **7e′**, **7k′**, **7l′**, and **7o′**) and the respective yields are collated in Scheme 5. The inclusion of an analogue featuring the scoparone core highlights the potential of this methodology for late-stage functionalization of natural product scaffolds.^34^

**Scheme 5:**
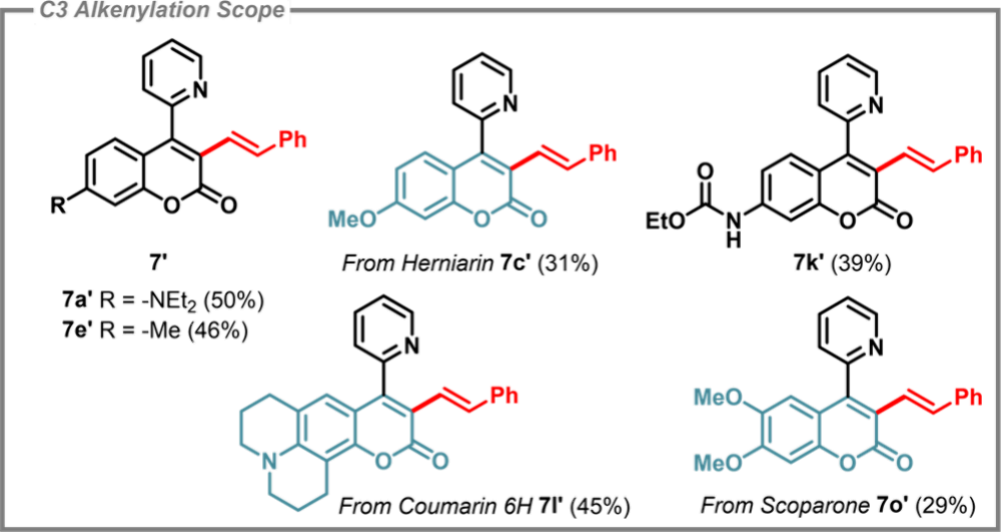
Scope of Catalytic C3 C–H Alkenylated 4-(2-Pyridyl)­coumarins **7a′**, **7c′**, **7e′**, **7k′**, **7l′,** and **7o′** Using Condition 7 in Table 3 (Isolated Yields in Parentheses)

These data demonstrate that there is a marked positional effect on the chemistry of the coumarin derivatives. Notably, catalytic C3 alkenylation could be induced using substrates (**5**), whereas in the case of (**1**), only products arising from C–N reductive elimination (**3**) could be obtained under all conditions tried. This demonstrates a fundamental difference in the reactivity between the two positional isomers. A series of DFT calculations (Figure 8) were then performed to rationalize these observations.

The TRIR studies had already established that following CO loss, the alkyne may bind to the metal in an η^2^-fashion (**2c** **_PhCCH_** and **6c** **_PhCCH_**), and a subsequent migratory insertion then gives manganacycles (**2c**-**7-a**) and (**6c**-**7-a**), respectively. Although there is a marked difference between the two systems, with the C3 isomer undergoing migratory insertion more rapidly, this step will not be rate controlling for either isomer.

In our previous studies,^22^ the seven-membered manganacycles were shown to be crucial branching points in the reactions. This also appears to be the case in this work. Transition states were located for the formal C–N reductive elimination for both isomers with the energetic span from the corresponding manganacycles being +72 and +68 kJ mol^–1^ for the C4 and C3 isomers, respectively. This small difference again would not be expected to influence the reaction outcome.

Our attention then focused on the role of acid additive (EtCO_2_H) in the reaction. In previous mechanistic studies,^35,36^ we have demonstrated that acid additives play a crucial role in the liberation of the product from the manganese, a process that involves Mn–C bond protonolysis. Furthermore, using our TRIR method, we were able to directly observe this reaction step,^37^ illustrating how carboxylic acids have a key role in product liberation. Therefore, pathways leading to intramolecular protonolysis of the Mn–C bond were calculated.

Using the C4-isomer as an example, the acid coordinates to the seven-membered manganacycle through the carbonyl oxygen to give (**4c**). This was followed by the formation of (**4c-7-a**) in which there is a weak interaction between the O–H proton and the Mn-bound carbon atom: we^37^ and others^38,39^ have previously identified related states in proton-transfer reactions. The state (**4c-7-a**) then undergoes proton transfer through the transition state (**TS** **_4c‑7‑a_**) to give (**10c_iso**) that then converts to a lower-energy isomer (**10c**). Essentially, structurally analogous states were located for the C3-isomer.

Analysis of the relative energies of the protonation pathways for the two isomers provides insight into the potential reason for the difference in reactivity. The protonation surface for the C3-isomer (which does undergo successful catalysis) is notably lower than that for the unsuccessful C4-isomer. Specifically, the energic span for the protonation of the manganacycle (**6c-7-a**) is much lower (+51 kJ mol^–1^, difference between (**6c-7-a**) and (**TS** **_8c‑7‑a_**)) in the case of the C3-isomer compared to the C4-isomer (+71 kJ mol^–1^, difference between (**2c-7-a**) and (**TS** **_4c‑7‑a_**)). Although it can be challenging to compare the quantitative difference between DFT-calculated unimolecular and bimolecular pathways, it is argued that the rate of protonation of the C3-isomer will be more rapid; hence, it is able to outcompete nonproductive reductive elimination through (**TS** **_6c‑7‑a_**), thus providing a route to the desired products (**7′**). In the case of the C4-isomer, the higher barrier to protonation precludes the possibility, and hence, only the reductive elimination products are obtained.

## Conclusions

Herein, we have demonstrated an exemplar proving ground for the origins and exploitation of site-selective C–H functionalization of a coumarin framework drawing on the intrinsic reactivity of the different positions at C3–H and C4–H. The delicate interplay between the ICT properties of coumarins has been manipulated through judicial positioning of electron-donating and -withdrawing groups (on the coumarin) to either enhance or reduce the nucleophilicity of the model reaction of (**1**) or (**5**) with alkynes. Under a thermal regime, the effect of such a substituent is observed, but when explored photochemically, a consistent behavior is found in the observed insertion rate *k*_2_ with altering substituents. The position of the 2-pyridyl group, required for directing the position of C–H functionalization, has also been applied to take advantage of enhanced push–pull dynamics and highlight the difference in electronics between the C3 and C4 positions on the coumarin framework. The results from the experimental work (NMR and TRIR) and computational studies (DFT) highlight the differences in reactivity at C3–H and C4–H in the coumarin moiety. The contrast in the nature of the C3–H site compared to the C4–H site opens up a previously forbidden pathway for catalytic C–H hydroarylation, which is now a feasible catalytic process. The results demonstrate the first directed earth-abundant metal catalyzed C3-functionalization of the coumarin ring system. Through deployment of the five-membered manganacycle of 4-(2-pyridyl)­coumarins (**6**), the insertion of alkynes have been studied by TRIR spectroscopy to confirm whether they proceed under the same reactive intermediates as 3-(2-pyridyl)­coumarins (**2**). As a direct result, the electronics and coumarin positioning result in a greater insertion rate *k*_2_, further supporting the nucleophilic nature of the coumarin C3 site. More generally, our studies have alluded to the importance of site selectivity on heteroaromatic frameworks through exploiting measurable intrinsic properties and how this can be tuned for exploitation in the development of synthetic transformations in the future.

## Experimental Section

### General Synthetic Methods

#### General Procedure for Substituted 3-(2-Pyridyl)­coumarins

To a round-bottomed flask equipped with a magnetic stirrer bar were added substituted salicylaldehyde (0.5 mmol, 1.0 equiv), pyridine-2-acetonitrile (0.5 mmol, 1.0 equiv), and piperidine (0.01 equiv) in ethanol (5 mL mmol^–1^). The solution was heated to reflux for 4 h and was left to stir at room temperature for a further 18 h. To the solution, 3% aqueous hydrochloric acid (10 mL mmol^–1^) was added, and the resulting solution was stirred at reflux for 6 h. The solution was then neutralized with aqueous ammonium hydroxide until pH = 7. The precipitate was then filtered by vacuum filtration, and the solid was washed with cold water (ca. 25 mL) and then dried *in vacuo.*

#### General Procedure for the Cyclomanganation of Coumarins

To a flame-dried Schlenk tube under N_2_ equipped with a magnetic stirrer bar were added coumarin (1.0 equiv) and [MnBn­(CO)_5_] (1.0 equiv) followed by dry toluene (50 mL mmol^–1^). The solution was heated to 95 °C with stirring and was left to continue stirring for a further 2.5 h. Upon completion, the reaction was cooled to room temperature, and the mixture was concentrated *in vacuo.*

#### General Procedure for the Reductive Elimination Reaction in the Absence of TMNO

To a flame-dried Schlenk tube under N_2_ equipped with a magnetic stirrer bar was added cyclometalated coumarin (1.0 equiv) in dry diethyl ether (60 mL mmol^–1^). To the solution, alkyne (1.5 equiv) was added, and the solution was heated to 80 °C and allowed to continue stirring for a further 18 h. Upon cooling to room temperature, the reaction mixture was diluted with EtOAc (15 mL), and the solution was concentrated *in vacuo*. The crude product was dissolved in minimal dichloromethane, precipitated out with excess hexane (ca. 10-fold excess to dichloromethane), precipitate filtered, and dried *in vacuo.*

#### General Procedure for the Reductive Elimination Reaction in the Presence of TMNO

To a flame-dried Schlenk tube under N_2_ equipped with a magnetic stirrer bar was added cyclometalated coumarin (1.0 equiv) in dry diethyl ether (60 mL mmol^–1^). To the solution was added alkyne (1.5 equiv), along with TMNO (1.0 equiv), and the solution was heated to 80 °C and left to continue stirring for a further 18 h. Upon cooling to room temperature, the reaction mixture was diluted with EtOAc (15 mL), and the solution was concentrated *in vacuo*. The crude product was dissolved in minimal dichloromethane, precipitated out with excess hexane (ca. 10-fold excess to dichloromethane), precipitate filtered, and dried *in vacuo.*

#### General Procedure for the Photoinduced Reductive Elimination Reaction

To an 8 mL vial with a magnetic stir bar were added cyclometalated coumarin (1.0 equiv) and alkyne (1.5 equiv); the vial lid was then crimped shut. To this was added dry diethyl ether (3 mL) through the septum in the lid. With stirring, a single 25 W, 355 nm LED was placed directly beneath the vial and irradiated for 2 h. Upon completion, the desired product was precipitated out with excess hexane (ca. 10-fold. excess to diethyl ether), precipitate filtered, and dried *in vacuo.*

## Supplementary Material



## Data Availability

The data underlying this study are available in the published article and its Supporting Information and are openly available on the York Research Data repository at 10.15124/c1b8bb34-b732-48c5-ae50-bf6e68d3a911 and upon request from the authors. The microED data can be accessed at 10.5281/zenodo.18700749.

## Notes

Experimental procedures, representative NMR spectra, DFT Cartesian coordinates (*XYZ*), additional TRIR spectra, details of computational chemistry methods, collated rate constants, and calculated energies (PDF)
