# Supplementary material for: Site-Selective C–H Functionalization on Coumarins Directed by Manganese: Mechanistic Insights from Time-Resolved Spectroscopy and Catalytic Development
Source: ACS Org Inorg Au. 2026 Apr 13;6(3):325–36. doi: 10.1021/acsorginorgau.6c00003 (PMC13237603; doi:10.1021/acsorginorgau.6c00003)
Supplement: Supplementary file 1 [file gg6c00003_si_001.pdf]

Site-Selective C–H Functionalization on Coumarins Directed by Manganese: Mechanistic Insights from Time-Resolved Spectroscopy and Catalytic Development

Thomas J. Burden,[a] Jonathan B. Eastwood,[a] Emily A. Thompson,[a] Matteo Albino,[a] Ian P. Clark,[b] Adrian C. Whitwood,[a] Jean-Philippe Krieger,[c] Matthew J. Harper,[d] Huw T. Jenkins,*[a] Ian J. S. Fairlamb*[a] and Jason M. Lynam*[a]

huw.jenkins@york.ac.uk; ian.fairlamb@york.ac.uk; jason.lynam@york.ac.uk

[a] Department of Chemistry, University of York, Heslington, York, YO10 5DD, UK

[b] Central Laser Facility, Research Complex at Harwell, STFC Rutherford Appleton Laboratory, Harwell Campus, Didcot, Oxfordshire, OX11 0QX, UK.

[c] Syngenta Crop Protection AG, Münchwilen, Breitenloh 5, 4333, Switzerland.

[d] Syngenta Crop Protection, Jealotts Hill, Bracknell, RG42 6EY, UK.

Important Safety Note

General safety precautions were enacted (use of blast shield; working on small-scale) when working with diethyl ether at 80 °C. No incidents occurred handling the solvent at this temperature, but readers should be aware of potential danger and exercise caution. A potential substitute is di-n-butyl ether.

1 Table of Contents

2 General Experimental Details ... 3

3 Experimental Procedures and Characterisation ... 7

4 Electronic spectra ... 30

5 NMR Studies ... 31

6 DFT Computational Calculations Using Density Functional Theory (DFT) Methods ... 32

7 Atomic Contribution, Mulliken Charges, and Electrostatic Potential Mapping ... 32

8 DFT xyz Coordinates ... 32

9 Atomic Contributions of the HOMO's and Mulliken Charges ... 36

10 Electrostatic Potential Maps ... 38

11 Coumarin Reaction Pathway DFT Methodology ... 39

12 Molecular NBO DFT Methodology ... 40

13 Coumarin Reaction Pathway DFT xyz Coordinates ... 40

14 Time Resolved Infrared Spectroscopy (TRIR) ... 100

15 Variable alkyne concentration studies ... 100

16 Studies in neat Phenylacetylene (PhC_2H) ... 104

17 X-Ray Crystal and MicroED Structures ... 131

18 X-Ray Crystallography ... 131

19 MicroED ... 131

20 NMR Spectra ... 145

21 References ... 178

2 General Experimental Details

Commercially sourced solvents and reagents including **5m** were purchased from Acros Organics, Alfa Aesar, Fisher Scientific, Fluorochem, Sigma-Aldrich or VWR and used as received unless otherwise noted. Petrol refers to the fraction of petroleum ether boiling in the range of 40–60 °C. Room temperature (RT) refers to reactions where no thermostatic control was applied and was recorded as 16–23 °C. Reactions performed at elevated temperatures were performed in a silicone oil bath on a stirrer hot plate with an internal temperature probe.

Thin layer chromatography (TLC) analysis was performed using Merck 5554 aluminium backed silica plates. Spots were visualised by the quenching of ultraviolet light (λ_{max} = 254 nm) or through staining with $KMnO_4$. Retention factors (R_f) are quoted to two decimal places and reported along with the solvent system used in parentheses. All flash column chromatography was performed using either Merck 60 or Fluorochem 60 Å silica gel (particle size 40–63 µm) and the solvent system used is reported in parentheses.

Melting points were recorded using a Stuart digital SMP3 machine using a temperature ramp of 5 °C min^{-1} and are quoted to the nearest whole number. Where applicable, decomposition (dec.) is noted.

All NMR spectra were recorded on either Jeol ECS400, Jeol ECX400, Bruker AVIIIHD 500, or Bruker AVIIIHD 600 (typically at 298 K). Chemical shifts are reported in parts per million (ppm) of tetramethylsilane. Coupling constants (J) are reported in Hz and quoted to ±0.5 Hz. Multiplicities are described as singlet (s), doublet (d), triplet (t), quartet (q), quintet (quin), sextet, (sext), heptet (hept), multiplet (m), apparent (app) and broad (br). Spectra were processed using MestReNova version 12. NMR spectra are representative of the compounds prepared. NMR samples of manganese-containing species are recorded at dilute concentrations to facilitate shimming correctly, these solvent impurities have no impact on final prepared compounds and yields of dried solids are representative.

Proton (^1H) spectra were typically recorded at 600, 500, and 400 MHz. Chemical shifts are internally referenced to residual non-deuterated solvent (CD_2Cl_2 δH = 5.32 ppm), ($CDCl_3$ δH = 7.26 ppm), and (DMSO-d_6 δH = 2.50 ppm), given to two decimal places. Carbon ($^{13}C\{^1H\}$) spectra were recorded at 125 and 101 MHz. Fluorine (^{19}F) NMR were recorded at 376 MHz. Chemical shifts are internally referenced to residual solvent (CD_2Cl_2 δC = 58.0 ppm), ($CDCl_3$ δC = 77.0 ppm), (CD_3CN δC = 118.3 ppm), and (DMSO-d_6 δC = 39.5 ppm) and given to one decimal place.

Electrospray ionisation (ESI) mass spectrometry was performed using a Bruker Daltronics microTOF spectrometer. Electron impact (EI) mass spectrometry was performed using a Waters GCT Premier mass spectrometer. Mass to charge ratios (m/z) are reported in Daltons with percentage abundance in parentheses along with the corresponding fragment ion, where known. Where complex isotope patterns were observed, the most abundant ion is reported. High resolution mass spectra (HRMS) are reported with less than 10 ppm error.

Infrared spectra were recorded on a Perkin Elmer UATR Two FT-IR spectrometer. Absorption maxima (ν_{max}) are reported in wavenumbers (cm^{-1}) to the nearest whole number. Time-resolved infra-red spectra were recorded on the LIFEtime instrument in the ULTRA facility at the STFC's Rutherford Appleton Laboratories. The pump source was the output of a Yb:KGW amplifier providing 15W, 260 fs pulses at 1030 nm with a 100 kHz repetition rate (Pharos) that is divided down to 1 kHz. This was used to drive a BBO-based 515 nm pumped optical parametric amplifier (OPA) to deliver pulses at 355 nm. The pump beam was collimated, travelled over a computer programmable 0 - 16 ns optical delay (1200 mm long, double pass), and focused onto the sample. The pump energy at the sample was attenuated down to 500 nJ and focused down to a 120 µm diameter spot. The probe source was the output of a Yb:KGW amplifier providing 6 W, 180 fs pulses at 1030 nm with a 100 kHz repetition rate (Pharos). This was split 50/50 to pump two 3 W BBO/KTA based OPAs. The two Pharos sources (pump and probe) share a common 80 MHz oscillator to allow for pump-probe delay steps of 12.5 ns. The probe beam was split to provide probe and reference pulses. To go beyond pump-probe delays of 12.5 ns, subsequent seed pulses were selected from the 80 MHz oscillator. Data were collected using pump-probe delays ranging from 1 ps to 988.8 µs. The probe beams were collimated, synchronised by a fixed optical delay, and focused by a single

gold parabolic mirror onto the sample. The three beams were overlapped on the sample with the aid of a 50 µm pinhole in the sample plain. The probe beams were measured by two separate spectrometers and 128-element mercury cadmium telluride (MCT) detectors. To cover the full spectroscopic window required, data from different detector positions were combined to generate the required spectra. Data were initially visualized in the ULTRA View version 2 software,[1] where baseline correction was undertaken. The resulting spectra were then exported as comma-separated variable files into OriginPro 24.[2] The spectra were calibrated against samples of polystyrene (200 µm thick), [Mn(ppy)$(CO)_4$] in heptane and 1,4-dioxane. Kinetic analysis was performed by fitting to the expgro, expdec, or expgrodec functions in OriginPro 24. Analysed TRIR spectra recorded after 800 ns were smoothed using LOESS smoothing (0.04) to remove instrument induced etaloning. All quoted errors are 95% confidence limits.

General Procedure for Substituted 3-(2-pyridyl)coumarins

To a round bottomed flask, equipped with a magnetic stirrer bar, was added substituted salicylaldehyde (0.5 mmol, 1.0 eq.), pyridine-2-acetonitrile (0.5 mmol, 1.0 eq.) and piperidine (0.01 eq.) in ethanol (5 mL $mmol^{-1}$). The solution was heated to reflux for 4 h, solution was left to stir at room temperature for a further 18 h. To the solution, 3% aqueous hydrochloric acid (10 mL $mmol^{-1}$) was added and resulting solution stirred at reflux for 6 h. The solution was then neutralised with aqueous ammonium hydroxide until pH = 7. The precipitate was then filtered by vacuum filtration and solid was washed with cold water (*ca.* 25 mL) solid was then dried *in vacuo*.

General Procedure for Cyclomanganation of Coumarins

To a flame-dried Schlenk tube under N_2, equipped with a magnetic stirrer bar, was added coumarin (1.0 eq.) and [$Mn(CO)_5Bn$] (1.0 eq.), followed by dry toluene (50 mL $mmol^{-1}$). The solution was heated to 95 ^oC with stirring, which was left to continue stirring for a further 2.5 h. Upon completion, the reaction was cooled to room temperature and the mixture concentrated *in vacuo*.

General Procedure for Reductive Elimination reaction in absence of TMNO

To a flame-dried Schlenk tube under N_2, equipped with a magnetic stirrer bar, was added cyclometalated coumarin (1.0 eq.) in dry diethyl ether (60 mL $mmol^{-1}$). To the solution, alkyne (1.5 eq.) added, and solution was heated to 80 ^oC, left to continue stirring for a further 18 h. Upon cooling to room temperature, the reaction mixture was diluted with EtOAc (15 mL) and the solution concentrated *in vacuo*. Crude product dissolved in minimal dichloromethane and precipitated out with excess hexane (*ca.* 10-fold excess to dichloromethane), precipitate filtered and dried *in vacuo*.

General Procedure for Reductive Elimination reaction in presence of TMNO

To a flame-dried Schlenk tube under N_2, equipped with a magnetic stirrer bar, was added cyclometalated coumarin (1.0 eq.) in dry diethyl ether (60 mL $mmol^{-1}$). To the solution, alkyne (1.5 eq.) added, along with TMNO (1.0 eq.), and solution was heated to 80 ^oC, left to continue stirring for a further 18 h. Upon cooling to room temperature, the reaction mixture was d l l

uted with EtOAc (15 mL) and the solution concentrated *in vacuo*. Crude product was dissolved in minimal amount of dichloromethane and precipitated out with excess hexane (*ca.* 10-fold excess to dichloromethane), and the precipitate filtered and dried *in vacuo*.

General Procedure for Photo-induced Reductive Elimination Reaction

To an 8 mL vial with magnetic stirrer bar, cyclometalated coumarin (1.0 eq.) and alkyne (1.5 eq.) were added, vial lid was then crimped shut. To this, dry diethyl ether (3 mL) was added through the septum in the lid. With stirring, a single 25 W, 355 nm LED was placed directly beneath the vial and irradiated for 2 h. Upon completion, desired product was precipitated out with excess hexane (*ca.* 10-fold. excess to diethyl ether), precipitate filtered and dried *in vacuo*.

3 Experimental Procedures and Characterisation

The preparation of benzyl manganese pentacarbonyl [MnBn(CO)$_5$] was previously reported by our group.[3]

Compounds **1a, 1b, 1c, 1e, 1g, 2a, 2b, 2b', 2c, 2e, 3a, 3b, 3c,** and **3g** were synthesised previously within the research group, details for their synthesis is available.[4] Single crystal XRD data has been reporte for **2b**[4a] and **1b**[4b].

Mn(CO)$_3$(MeCN)$_2$Br was synthesised according to literature.[5]

7-(diethylamino)-3-(pyridin-2-yl)-2*H*-chromen-2-one-^{13}C **1a'**

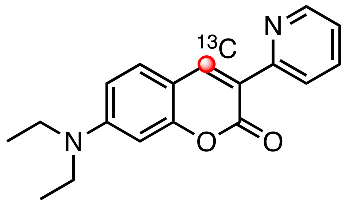

To a round bottomed flask, equipped with a magnetic stirrer bar, was added 4-(*N,N*-diethylamino)salicylaldehyde-^{13}C (10.0 mmol, 1.93 g, 1.0 eq.) and pyridine-2-acetonitrile (10.0 mmol, 1.18 g, 1.0 eq.) in absolute ethanol (20 mL). The solution was cooled to 0 ºC and then piperidine (0.6 mL) was added dropwise with stirring, resulting solution was left to continue stirring for a further 12 h at 0 °C. Upon completion, to the reaction was added HCl (3.5%, 50 mL) with refluxing for 8 h to hydrolyse the iminocoumarin. The solution was then neutralised with aqueous ammonia until the pH was 7. The solvent was then removed *in vacuo*, and the dried product was extracted with dichloromethane (2 x 50 mL). Organic phase

was washed with water (2 x 50 mL), the combined organic extracts were dried ($MgSO_4$), filtered and concentrated *in vacuo*. The crude product was suspended in minimal dichloromethane (4 mL) and purified by automated silica gel column chromatography (hexane:EtOAc, 8:1, v/v) to afford the product **1a'** as an orange solid (1.33 g, 45%).

^{1}H NMR (400 MHz, Chloroform-*d*) δ 8.70 (d, *J* = 162.0 Hz, 1H), 8.64 (ddd, *J* = 4.8, 1.9, 0.9 Hz, 1H), 8.43 (d, *J* = 8.1 Hz, 1H), 7.75 (td, *J* = 7.9, 1.9 Hz, 1H), 7.43 (dd, *J* = 8.9, 4.8 Hz, 1H), 7.22 (ddd, *J* = 7.7, 4.7, 1.1 Hz, 1H), 6.62 (dd, *J* = 8.9, 2.5 Hz, 1H), 6.53 (d, *J* = 2.4 Hz, 1H), 3.44 (q, *J* = 7.1 Hz, 4H), 1.24 (t, *J* = 7.1 Hz, 6H).

^{13}C{^{1}H} NMR (101 MHz, Chloroform-*d*) δ 161.6, 157.0, 152.7, 151.5, 149.1, 143.3*, 137.0, 130.3, 123.6, 122.3, 117.7, 109.5, 109.4, 96.9, 45.1, 12.6. *^{13}C enriched environment.

IR (solid-state ATR, cm^{-1}) 2981, 2930, 1713, 1599, 1513, 1467, 1409, 1345, 1271, 1166. MS;

HRMS (ESI+) m/z: [M-H]+ calcd for $C_{17}{}^{13}CH_{19}N_2O_2$ 296.1475; found 296.1482.

7-trifluoromethyl-3-(pyridin-2-yl)-*2H*-chromen-2-one **1d**

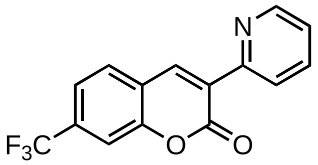

Synthesised according to general procedure for substituted 3-(2-pyridyl)coumarins from 2-hydroxy-4- (trifluoromethyl)benzaldehyde (125 mg, 0.657 mmol, 1.0 eq.), 2-pyridylacetonitrile (78 µL, 0.657 mmol, 1.0 eq.). Precipitate was then filtered by vacuum filtration and solid was washed with cold water (*ca.* 25 mL) solid was then dried *in vacuo*, to afford **1d** as a light brown powder (149 mg, 78%).

^{1}H NMR (400 MHz, Chloroform-*d*) δ 8.81 (d, *J* = 0.7 Hz, 1H), 8.71 (ddd, *J* = 4.8, 1.9, 1.0 Hz, 1H), 8.43 (dt, *J* = 8.1, 1.0 Hz, 1H), 7.82 (ddd, *J* = 8.1, 7.6, 1.9 Hz, 1H), 7.79 – 7.76 (m, 1H), 7.65 – 7.63 (m, 1H), 7.56 (ddd, *J* = 8.1, 1.7, 0.7 Hz, 1H), 7.35 (ddd, *J* = 7.6, 4.8, 1.1 Hz, 1H).

^{13}C{^{1}H} NMR (101 MHz, Chloroform-*d*) δ 159.5, 153.5, 150.6, 149.7, 141.1, 137.0, 133.6 (q, *J* = 33.5 Hz), 129.6, 127.6, 124.4, 124.2, 122.2, 122.0, 121.3 (q, *J* = 3.6 Hz), 113.9 (q, *J* = 4.0 Hz).

^{19}F NMR (376 MHz, Chloroform-*d*) δ -62.7.

IR (solid-state ATR, cm^{-1}) 3055, 1725, 1613, 1584, 1507, 1467, 1435, 1422, 1361, 1319, 1259, 1217, 1167, 994, 873, 797.

MS; HRMS (ESI+) m/z: [M-H]+ calcd for $C_{15}H_9F_3NO_2$ 292.0580; found 292.0581.

MP 127–129 °C.

7-methyl carboxylate-3-(pyridin-2-yl)-*2H*-chromen-2-one **1f**

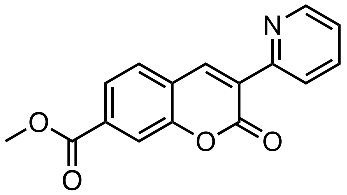

Synthesised according to general procedure for substituted 3-(2-pyridyl)coumarins from methyl 4-formyl-3-hydroxybenzoate (125 mg, 0.694 mmol, 1.0 eq.), 2-pyridylacetonitrile (82 mg, 0.694 mmol, 1.0 eq.). Precipitate was then filtered by vacuum filtration and solid was washed with cold water (*ca.* 25 mL) solid was then dried *in vacuo*, to afford **1f** as a light brown powder. (123 mg, 63%)

^{1}H NMR (400 MHz, Chloroform-*d*) δ 8.80 (s, 1H), 8.71 (dt, *J* = 4.8, 1.4 Hz, 1H), 8.44 (d, *J* = 8.0 Hz, 1H), 8.03 (s, 1H), 7.98 (dd, *J* = 8.1, 1.6 Hz, 1H), 7.82 (td, *J* = 7.8, 1.9 Hz, 1H), 7.72 (d, *J* = 8.1 Hz, 1H), 7.39 – 7.31 (m, 1H), 3.98 (s, 3H).

^{13}C{^{1}H} NMR (101 MHz, Chloroform-*d*) δ 165.9, 159.9, 153.6, 150.9, 149.7, 141.4, 137.0, 133.3, 129.0, 127.5, 125.5, 124.5, 124.1, 123.0, 117.7, 52.9.

IR (solid-state ATR, cm^{-1}) 3047, 1720, 1609, 1581, 1555, 1469, 1428, 1415, 1343, 1279, 1219, 1140, 1115, 1087, 998, 939, 896, 804, 768, 703.

MS; HRMS (ESI+) m/z: [M-H]+ calcd for $C_{16}H_{12}NO_4$ 282.0763; found 282.0763.

MP 190–192 °C.

7-(pyrrolidin-1-yl)-3-(pyridin-2-yl)-*2H*-chromen-2-one **1h**

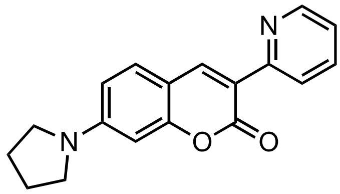

Synthesised according to general procedure for substituted 3-(2-pyridyl)coumarins from 2-hydroxy-4-(pyrrolidin-1- yl)benzaldehyde (55.0 mg, 0.288 mmol, 1.0 eq.), 2-pyridylacetonitrile (32 µL, 0.288 mmol, 1.0 eq.). Precipitate was then filtered by vacuum filtration and solid was washed with cold water (*ca.* 25 mL) solid was then dried *in vacuo*, to afford **1h** as a brown powder (83.3 mg, 99%). N.B. a small amount of unreacted 2-hydroxy-4-(pyrrolidin-1-yl)benzaldehyde, was observed, material was used in metalation where it was removed.

^{1}H NMR (400 MHz, Chloroform-*d*) δ 8.67 (s, 1H), 8.63 (ddt, *J* = 4.8, 1.8, 0.8 Hz, 1H), 8.41 (dd, *J* = 8.1, 0.8 Hz, 1H), 7.73 (dddd, *J* = 8.1, 7.4, 1.9, 0.6 Hz, 1H), 7.43 (d, *J* = 8.6 Hz, 1H), 7.22 – 7.15 (m, 1H), 6.57 – 6.47 (m, 1H), 6.42 (d, *J* = 2.3 Hz, 1H), 3.39 (t, *J* =, 6.5 Hz, 4H), 2.11 – 2.04 (m, 4H).

^{13}C{^{1}H} NMR (101 MHz, Chloroform-*d*) δ 161.7, 156.7, 152.8, 151.0, 149.2, 143.4, 136.6, 130.1, 123.5, 122.3, 117.8, 110.1, 109.5, 97.3, 48.0, 25.6.

IR (solid-state ATR, cm^{-1}) 2955, 2846, 1704, 1615, 1595, 1576, 1512, 1486, 1402, 1368, 1349, 1312, 1276, 1239, 1210, 1189, 1000, 925, 819, 799.

MS; HRMS (ESI+) m/z: [M-H]+ calcd for $C_{18}H_{17}N_2O_2$ 293.1285; found 293.1285.

MP 194–196 °C.

6-methoxy-3-(pyridin-2-yl)-2H-chromen-2-one **1cc**

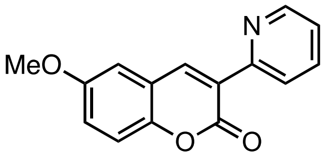

Synthesised according to general procedure for substituted 3-(2-pyridyl)coumarins afforded as a light brown powder (Yield: 0.100 g, 79%).

^{1}H NMR (400 MHz, Chloroform-*d*) δ 8.73 (s, 1H), 8.71 – 8.62 (m, 1H), 8.47 – 8.33 (m, 1H), 7.79 (td, *J* = 7.8, 1.9 Hz, 1H), 7.38 – 7.28 (m, 2H), 7.15 (dd, *J* = 9.0, 2.9 Hz, 1H), 7.07 (d, *J* = 2.9 Hz, 1H), 3.87 (s, 3H).

$^{13}C\{^1H\}$ NMR (101 MHz, Chloroform-*d*) δ 160.5, 156.2, 151.4, 149.4, 148.5, 142.4, 136.7, 125.7, 124.1, 123.5, 120.2, 119.9, 117.4, 110.4, 55.9.

The analytical data obtained was in accordance with the literature.[6]

6-(trifluoromethyl)-3-(pyridin-2-yl)-2H-chromen-2-one **1dd**

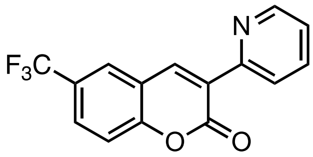

Synthesised according to general procedure for 6-Substituted 3-(2-pyridyl)coumarins, afforded as a brown powder (Yield: 0.105 g, 72%).

^1H NMR (400 MHz, Chloroform-d) δ 8.82 (s, 1H), 8.70 (ddd, *J* = 4.7, 1.9, 1.0 Hz, 1H), 8.42 (d, *J* = 8.1 Hz, 1H), 7.93 (d, *J* = 2.1 Hz, 1H), 7.81 (ddd, *J* = 10.1, 5.9, 2.0 Hz, 2H), 7.49 (d, *J* = 8.7 Hz, 1H), 7.34 (ddd, *J* = 7.6, 4.8, 1.2 Hz, 1H).

^{19}F NMR (377 MHz, Chloroform-*d*) δ -62.1.

$^{13}C\{^1H\}$ NMR (101 MHz, Chloroform-d) δ 159.5, 155.6, 150.6, 149.7, 141.4, 136.9, 128.6 (d, *J* = 3.6 Hz), 127.3 (q, *J* = 33.6 Hz), 126.8, 126.3 (d, *J* = 4.0 Hz), 124.9, 124.1 (d, *J* = 15.4 Hz), 122.2, 119.5, 117.3.

MS; HRMS (ESI+) m/z: [M-H]+ calcd for $C_{15}H_9F_3NO_2$ 292.0580; found 292.0582.

6-methyl-3-(pyridin-2-yl)-2*H*-chromen-2-one **1ee**

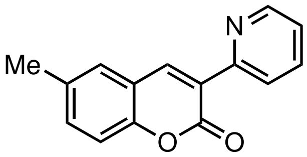

Synthesised according to general procedure for substituted 3-(2-pyridyl)coumarins affording a light brown powder (Yield: 0.104 g, 88%).

^{1}H NMR (400 MHz, Chloroform-*d*) δ 8.71 (s, 1H), 8.70 – 8.65 (m, 1H), 8.41 (d, *J* = 8.1 Hz, 1H), 7.79 (td, *J* = 7.8, 1.9 Hz, 1H), 7.42 (s, 1H), 7.38 (dd, *J* = 8.5, 2.1 Hz, 1H), 7.32 – 7.27 (m, 2H), 2.43 (s, 3H).

^{13}C{^{1}H} NMR (101 MHz, Chloroform-*d*) δ 159.8, 152.8, 150.9, 149.7, 141.1, 136.9, 135.0, 131.1, 128.7, 126.6, 124.3, 124.0, 121.2, 118.3, 21.0.

The analytical data obtained was in accordance with the literature.[7]

6-methyl-3-(pyridin-2-yl)-2*H*-chromen-2-one **1ff**

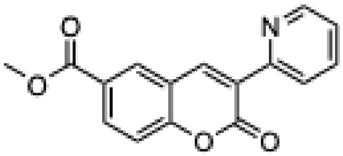

Synthesised according to general procedure for substituted 3-(2-pyridyl)coumarins affording a dark brown powder (Yield: 0.087 g, 62%).

^{1}H NMR (400 MHz, Chloroform-*d*) δ 8.80 (s, 1H), 8.73 – 8.62 (m, 1H), 8.38 (d, *J* = 8.1 Hz, 1H), 8.35 (d, *J* = 2.0 Hz, 1H), 8.21 (dd, *J* = 8.7, 2.1 Hz, 1H), 7.79 (td, *J* = 7.8, 1.9 Hz, 1H), 7.41 (d, *J* = 8.7 Hz, 1H), 7.32 (ddd, *J* = 7.6, 4.7, 1.1 Hz, 1H), 3.95 (s, 3H).

^{13}C{^{1}H} NMR (101 MHz, Chloroform-*d*) δ 165.8, 159.7, 156.7, 150.9, 149.7, 142.1, 136.9, 133.1, 131.0, 126.9, 126.3, 124.2, 123.9, 119.4, 116.7, 52.6.

MS; HRMS (ESI+) m/z: [M-H]+ calcd for $C_{16}H_{12}NO_4$ 282.0763; found 282.0763.

6-bromo-3-(pyridin-2-yl)-2H-chromen-2-one **1j**

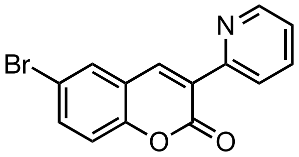

Synthesised according to general procedure for substituted 3-(2-pyridyl)coumarins affording a brown powder (Yield: 0.140 g, 93%).

^{1}H NMR (400 MHz, Chloroform-*d*) δ 8.70 – 8.68 (m, 2H), 8.41 (d, *J* = 8.1 Hz, 1H), 7.83 – 7.78 (m, 1H), 7.77 (d, *J* = 2.3 Hz, 1H), 7.65 (dd, *J* = 8.8, 2.3 Hz, 1H), 7.33 (ddd, *J* = 7.6, 4.8, 1.1 Hz, 1H), 7.28 (d, *J* = 8.9 Hz, 1H).

^{13}C{^{1}H} NMR (101 MHz, Chloroform-*d*) δ 159.7, 152.7, 150.8, 149.5, 141.0, 136.8, 134.8, 131.0, 126.5, 124.2, 123.8, 121.1, 118.1, 117.2.

MS; HRMS (ESI+) m/z: [M-H]+ calcd for $C_{14}H_{9}BrNO_{2}$ 301.9811; found 301.9797.

6-nitro-3-(pyridin-2-yl)-2H-chromen-2-one **1gg**

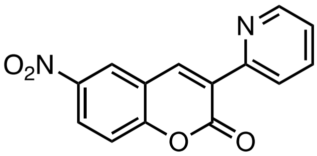

Synthesised according to general procedure for substituted 3-(2-pyridyl)coumarins, affording a light brown powder (Yield: 0.115 g, 86%).

^{1}H NMR (400 MHz, Chloroform-*d*) δ 8.88 (s, 1H), 8.72 (d, *J* = 4.2 Hz, 1H), 8.58 (d, *J* = 2.6 Hz, 1H), 8.46 – 8.39 (m, 2H), 7.83 (td, *J* = 7.8, 1.9 Hz, 1H), 7.52 (d, *J* = 9.1 Hz, 1H), 7.37 (ddd, *J* = 7.6, 4.8, 1.1 Hz, 1H).

^{13}C{^{1}H} NMR (101 MHz, Chloroform-*d*) δ 158.9, 157.3, 150.2, 149.9, 144.4, 141.0, 137.0, 127.6, 126.8, 124.7, 124.4, 124.3, 119.7, 117.7.

MS; HRMS (ESI+) m/z: [M-H]+ calcd for $C_{14}H_{9}N_{2}O_{4}$ 269.0557; Found 269.0554.

7-(diethylamino)-3-(pyridin-2-yl)-2*H*-chromen-2-one-^{13}C tetracarbonyl manganese **2a'**

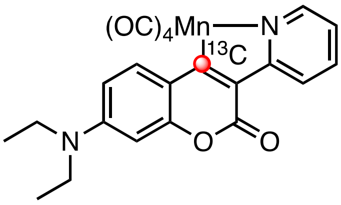

Synthesised according to general procedure for cyclomanganation of coumarins from **1a'** (70.1 mg, 0.237 mmol, 1.0 eq.), benzyl manganese pentacarbonyl (67.9 mg, 0.237 mmol, 1.0 eq.), in dry toluene. Product purified by removing solvent *in vacuo* to afford **2a'** as a dark brown solid (108.2 mg, 99%).

^{1}H NMR (400 MHz, Methylene Chloride-d_2) δ 8.90 (d, *J* = 8.4 Hz, 1H), 8.73 (ddd, *J* = 5.8, 1.7, 0.8 Hz, 1H), 8.05 (dd, *J* = 9.1, 4.5 Hz, 1H), 7.79 (ddd, *J* = 8.7, 7.3, 1.6 Hz, 1H), 7.07 (ddd, *J* = 7.3, 5.7, 1.5 Hz, 1H), 6.70 (dd, *J* = 9.1, 2.7 Hz, 1H), 6.47 (d, *J* = 2.7 Hz, 1H), 3.45 (q, *J* = 7.1 Hz, 4H), 1.22 (t, *J* = 7.1 Hz, 6H).

^{13}C{^{1}H} NMR (101 MHz, Methylene Chloride-d_2) δ 220.1, 217.7*, 214.0, 211.3, 170.7, 155.6, 152.9, 142.9, 134.9, 128.2, 124.9, 124.2, 121.1, 120.3, 109.1, 96.2, 44.9, 12.4. *^{13}C enriched environment.

IR (solid-state ATR, cm^{-1}) 2970, 2078, 1968, 1920, 1681, 1607, 1590, 1453, 1400, 1353, 1279, 1255, 1129, 1077, 1015, 950, 830, 788.

MS; HRMS (ESI+) m/z: [M-H]+ calcd for $C_{21}{}^{13}CH_{18}MnN_2O_6$ 462.0573; found 462.0574.

7-trifluoromethyl-3-(pyridin-2-yl)-2*H*-chromen-2-one tetracarbonyl manganese **2d**

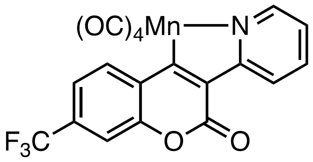

Synthesised according to general procedure cyclomanganation of coumarins from **1d** (85.0 mg, 0.292 mmol, 1.0 eq.), benzyl manganese pentacarbonyl (83.5 mg, 0.292 mmol, 1.0 eq.), in dry toluene. Product purified by removing solvent *in vacuo* to afford **114** as a dark brown solid (0.133 mg, 100%)

^{1}H NMR (400 MHz, Methylene Chloride-d_2) δ 9.08 (d, *J* = 8.4 Hz, 1H), 8.88 (d, *J* = 5.6 Hz, 1H), 8.45 (d, *J* = 8.9 Hz, 1H), 7.95 (td, *J* = 8.0, 7.4, 1.8 Hz, 1H), 7.66 – 7.60 (m, 3H), 7.30 (tt, *J* = 6.5, 1.2 Hz, 1H).

^{13}C{^{1}H} NMR (151 MHz, Methylene Chloride-d_2) δ 219.4, 218.0, 214.5, 211.3, 164.9, 154.0, 153.6, 150.6, 139.4, 134.7, 134.6, 133.5 (q, *J* = 33.2 Hz), 132.4, 129.1, 127.8 (d, *J* = 395.0 Hz), 126.4, 125.9, 124.1 (q, *J* = 272.5 Hz), 123.9, 121.1 (d, *J* = 3.8 Hz), 114.6 (d, *J* = 4.0 Hz).

^{19}F NMR (376 MHz, Methylene Chloride-d_2) δ -63.1.

IR (solid-state ATR, cm^{-1}) 2087, 1980, 1936, 1697, 1594, 1573, 1469, 1430, 1410, 1345, 1318, 1265, 1219, 1171, 1128, 1068, 991, 951, 884, 831, 796, 753.

UV-Vis (in Dichloromethane, nm): λ_{max} 338.

MS; HRMS (ESI+) m/z: [M-H]+ calcd for $C_{19}H_8F_3MnNO_6$ 457.9679; found 457.9678.

7-methyl carboxylate-3-(pyridin-2-yl)-2*H*-chromen-2-one tetracarbonyl manganese **2f**

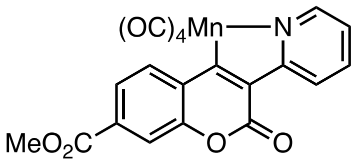

Synthesised according to general procedure for cyclomanganation of coumarins from **1f** (35.0 mg, 0.124 mmol, 1.0 eq.), benzyl manganese pentacarbonyl (35.5 mg, 0.124 mmol, 1.0 eq.), in dry toluene. Product **2f** purified by removing solvent *in vacuo* to afford dark brown solid (51.6 mg, 93%)

^{1}H NMR (400 MHz, Methylene Chloride-*d*$_2$) δ 9.06 (d, *J* = 8.2 Hz, 1H), 8.86 (dd, *J* = 5.7, 0.9 Hz, 1H), 8.36 (d, *J* = 8.2 Hz, 1H), 8.08 – 7.94 (m, 2H), 7.94 – 7.88 (m, 1H), 7.26 (ddd, *J* = 7.2, 5.8, 1.4 Hz, 1H), 3.94 (s, 3H).

^{13}C{^{1}H} NMR (126 MHz, Methylene Chloride-*d*$_2$) δ 219.3, 218.0, 214.6, 211.4, 166.3, 165.0, 154.0, 150.4, 139.3, 134.6, 133.9, 133.6, 133.1, 129.1, 126.4, 125.9, 125.1, 123.8, 118.3, 53.0.

IR (solid-state ATR, cm^{-1}) 3140, 2086, 1978, 1938, 1730, 1706, 1592, 1468, 1431, 1289, 1261, 1209, 1153, 1095, 1007, 894, 837, 801, 781.

UV-Vis (in Dichloromethane, nm): λ_{max} 322.

MS; HRMS (ESI+) m/z: [M-Na]+ calcd for $C_{20}H_{10}MnNNaO_8$ 469.9679; found 469.9706.

7-pyrrolidinyl-3-(pyridin-2-yl)-2*H*-chromen-2-one tetracarbonyl manganese **2h**

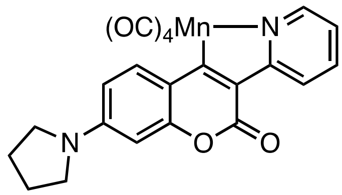

Synthesised according to general procedure B from **1h** (70.0 mg, 0.239 mmol, 1.0 eq.), benzyl manganese pentacarbonyl (68.0 mg, 0.239 mmol, 1.0 eq.), in dry toluene. Product **2h** purified by removing solvent *in vacuo* to afford dark brown solid. (109.5 mg, 100%)

^{1}H NMR (400 MHz, Methylene Chloride-*d*$_2$) δ 8.90 (d, *J* = 8.4 Hz, 1H), 8.73 (d, *J* = 5.7 Hz, 1H), 8.06 (d, *J* = 8.9 Hz, 1H), 7.79 (ddd, *J* = 8.6, 7.3, 1.7 Hz, 1H), 7.07 (ddd, *J* = 7.3, 5.7, 1.5 Hz, 1H), 6.61 (dd, *J* = 9.0, 2.5 Hz, 1H), 6.36 (d, *J* = 2.5 Hz, 1H), 3.43 – 3.35 (m, 4H), 2.09 – 1.98 (m, 4H).

^{13}C{^{1}H} NMR (126 MHz, Methylene Chloride-*d*$_2$) δ 220.0, 218.5, 215.1, 212.5, 166.5, 155.6, 153.4, 153.2, 151.7, 138.5, 135.3, 129.0, 126.4, 124.1, 121.6, 120.9, 110.2, 97.0, 48.4, 26.0.

IR (solid-state ATR, cm^{-1}) 2960, 2860, 2078, 1976, 1913, 1698, 1593, 1539, 1455, 1413, 1392, 1346, 1330, 1268, 1181, 1176, 1043, 971, 889, 809, 787.

MS; HRMS (LIFDI+) m/z: [M]+ calcd for $C_{22}H_{15}MnN_2O_6$ 458.0305; found 458.0304.

5-oxo-12-phenyl-8-(pyrrolidin-1-yl)-5*H*-chromeno[3,4-*a*]quinolizin-13-ium tricarbonyl manganese **3h**

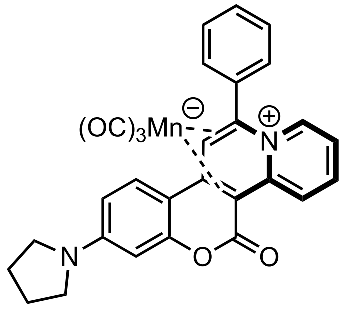

Synthesised according to general procedure for reductive elimination reaction in absence of TMNO from **2h** (41.4 mg, 0.090 mmol, 1.0 eq.), phenylacetylene (12 µL, 0.108 mmol, 1.2 eq.), in dry dibutyl ether. Product purified by dissolving in 1 mL dichloromethane and precipitated out with 20 mL pentane, solid filtered to afford **3h** as a dark brown solid (44.3 mg, 92%)

^{1}H NMR (400 MHz, Methylene Chloride-d_2) δ 7.97 (dt, *J* = 8.9, 2.6 Hz, 1H), 7.87 – 7.81 (m, 1H), 7.68 (d, *J* = 6.3 Hz, 1H), 7.59 (d, *J* = 7.5 Hz, 1H), 7.56 – 7.45 (m, 3H), 7.42 – 7.36 (m, 2H), 6.81 – 6.73 (m, 1H), 6.54 – 6.49 (m, 2H), 6.34 (q, *J* = 2.6 Hz, 1H), 3.46 – 3.14 (m, 4H), 2.25 – 1.92 (m, 4H).

^{13}C{^{1}H} NMR (126 MHz, Methylene Chloride-d_2) δ 231.9, 223.2, 218.6, 165.7, 157.0, 153.3, 150.2, 139.1, 137.7, 137.4, 134.2, 130.3, 130.0, 129.1, 128.7, 124.0, 122.6, 120.6, 108.6, 108.4, 99.2, 90.2, 72.8, 48.3, 26.0.

IR (solid-state ATR, cm^{-1}) 2847, 1978, 1874, 1710, 1620, 1484, 1443, 1351, 1258, 1143, 1114, 1017, 971, 898, 797.

MS; HRMS (ESI+) m/z: [M-H]+ calcd for $C_{29}H_{22}MnN_2O_5$ 533.0904; found 533.0900.

7-Diethylamino-4-(pyridin-2-yl)-2*H*-chromen-2-one **5a**

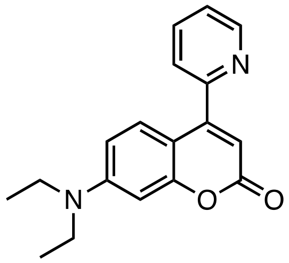

To a round bottomed flask, equipped with a magnetic stirrer bar, was added 3-diethylaminophenol (5.67 mmol, 0.937 g, 1.0 eq.), anhydrous zinc chloride (7.09 mmol, 0.966 g, 1.25 eq.), and ethyl 3-oxo-3-(pyridin-2-yl)propanoate (6.63 mmol, 1.28 g, 1.17 eq.) in absolute ethanol (25 mL). The solution was heated to reflux for 72 h. Upon cooling, to the reaction was added HCl (0.5%, 30 mL), solution was then extracted with dichloromethane (3 x 50 mL). Organic phase was washed with water (3 x 50 mL), the combined organic extracts was dry-loaded on silica and purified by automated silica gel column chromatography (Rf = 0.15 hexane:EtOAc, 1:1, v/v) to afford the product **5a** as an orange solid (0.398 g, 24%).

^{1}H NMR (400 MHz, Chloroform-*d*) δ 8.77 (d, *J* = 4.9 Hz, 1H), 7.85 (td, *J* = 7.8, 1.8 Hz, 1H), 7.55 (d, *J* = 7.8 Hz, 1H), 7.52 (d, *J* = 8.7 Hz, 1H), 7.44 – 7.36 (m, 1H), 6.58 – 6.50 (m, 2H), 6.16 (s, 1H), 3.41 (q, *J* = 7.1 Hz, 4H), 1.20 (t, *J* = 7.1 Hz, 6H).

^{13}C{^{1}H} NMR (101 MHz, Chloroform-*d*) δ 162.2, 157.1, 155.2, 153.6, 150.6, 149.6, 137.1, 128.0, 123.9, 122.1, 109.0, 108.8, 107.0, 97.8, 44.8, 12.5.

IR (solid-state ATR, cm^{-1}) 3066, 2960, 1702, 1601, 1590, 1562, 1488, 1361, 1226, 1075.

MS; HRMS (ESI+) m/z: [M-H]+ calcd for $C_{18}H_{19}N_2O_2$ 295.1441; found 295.1441.

7-methoxy-4-(pyridin-2-yl)-2*H*-chromen-2-one **5c**

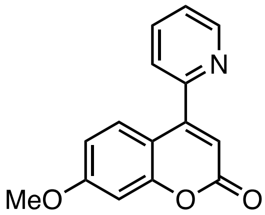

To a round bottom flask fitted with a condenser along with a stir bar, 7-hydroxy-4-(2-pyridyl)coumarin **5m** (0.300 g, 1.254 mmol, 1.0 eq.), along with K_2CO_3 (0.225 g, 1.630 mmol, 1.3 eq.), to this was added acetone (12 mL). To the mixture methyl iodide was added dropwise (80 µL, 1.317 mmol, 1.05 eq.). The resulting solution was refluxed at 80 °C for 4 h. Resulting mixture was cooled to room temperature and concentrated *in vacuo*. Crude product was

triturated with chloroform (8 mL), triturate was concentrated *in vacuo* to give purified product **5c** as a yellow powder (180.9 mg, 57%).

^{1}H NMR (400 MHz, Chloroform-*d*) δ 8.80 (dd, *J* = 4.9, 0.9 Hz, 1H), 7.89 (tt, *J* = 7.7, 1.3 Hz, 1H), 7.68 (d, *J* = 9.0 Hz, 1H), 7.58 (dt, *J* = 7.9, 0.9 Hz, 1H), 7.47 – 7.42 (m, 1H), 6.90 (d, *J* = 2.3 Hz, 1H), 6.83 (ddt, *J* = 8.9, 2.6, 0.9 Hz, 1H), 6.38 (s, 1H), 3.89 (s, 3H).

^{13}C{^{1}H} NMR (101 MHz, Chloroform-*d*) δ 163.0, 161.4, 158.9, 156.5, 154.7, 153.4, 149.9, 137.5, 128.4, 124.3, 124.1, 112.9, 112.7, 101.2, 55.9.

IR (solid-state ATR, cm^{-1}) 3057, 1715, 1614, 1604, 1587, 1569, 1550, 1508, 1470, 1456, 1435, 1380, 1345, 1279, 1261, 1245, 1185, 1177, 1125, 988, 904, 877, 818, 748.

MS; HRMS (ESI+) m/z: [M-H]+ calcd for $C_{15}H_{12}NO_3$ 254.0812; found 254.0819.

7-methyl-4-(pyridin-2-yl)-2*H*-chromen-2-one **5e**

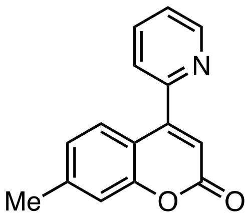

To a round bottomed flask, equipped with a magnetic stirrer bar, was added (*m*)-cresol (0.358 g, 3.31 mmol, 1.0 eq.), ethyl 3-oxo-3-(pyridin-2-yl)propanoate (0.767 g, 3.97 mmol, 1.2 eq.) and methanesulfonic acid (3 mL), resulting solution stirred overnight at room temperature. Reaction mixture then poured into ice water (50 mL) and extracted with ethyl acetate (3 x 50 mL). Combined organic fractions dried over sodium sulfate, filtered, and concentrated *in vacuo*. Crude solid product was recrystallized in minimal boiling methanol, product collected by filtration, washed with ice-cold methanol, dried to afford **5e** as a colourless crystalline solid. (0.200 g, 25%).

^{1}H NMR (400 MHz, Chloroform-*d*) δ 8.80 (d, *J* = 4.9 Hz, 1H), 7.97 – 7.82 (m, 1H), 7.63 (d, *J* = 8.2 Hz, 1H), 7.57 (dd, *J* = 7.8, 1.3 Hz, 1H), 7.49 – 7.40 (m, 1H), 7.21 (s, 1H), 7.07 (d, *J* = 8.2 Hz, 1H), 6.47 (d, *J* = 1.2 Hz, 1H), 2.45 (s, 3H).

^{13}C{^{1}H} NMR (101 MHz, Chloroform-*d*) δ 161.2, 154.7, 154.5, 153.3, 145.0, 143.4, 137.4, 127.0, 125.7, 124.3, 124.1, 117.5, 115.8, 115.1, 21.8.

IR (solid-state ATR, cm^{-1}) 3060, 1711, 1670, 1618, 1602, 1555, 1459, 1430, 1280, 1191, 1095, 955, 799, 740.

MS; HRMS (ESI+) m/z: [M-H]+ calcd for $C_{15}H_{12}NO_2$ 238.0863; found 238.0861.

7-Ethyl carbamate-4-(pyridin-2-yl)-2*H*-chromen-2-one **5k**

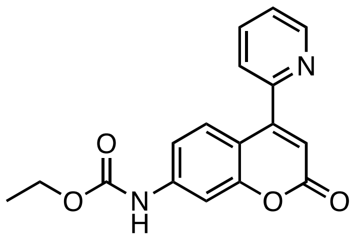

To a round bottomed flask, equipped with a magnetic stirrer bar, was added ethyl (3-hydroxyphenyl)carbamate (0.600 g, 3.31 mmol, 1.0 eq.), ethyl 3-oxo-3-(pyridin-2-yl)propanoate (0.767 g, 3.97 mmol, 1.2 eq.) and methanesulfonic acid (3 mL), resulting solution stirred overnight at room temperature. Reaction mixture then poured into ice water (50 mL) and extracted with ethyl acetate (3 x 50 mL). Combined organic fractions dried over sodium sulfate, filtered, and concentrated *in vacuo*. Crude solid product was recrystallized in minimal boiling methanol, product collected by filtration, washed with ice-cold methanol, dried to afford **5k** as a colourless crystalline solid (0.254 g, 25%).

^{1}H NMR (400 MHz, DMSO-d_6) δ 10.20 (s, 1H), 8.78 (dd, *J* = 4.8, 0.8 Hz, 1H), 8.01 (td, *J* = 7.8, 1.8 Hz, 1H), 7.77 (d, *J* = 7.9 Hz, 1H), 7.70 (d, *J* = 8.9 Hz, 1H), 7.64 (d, *J* = 2.2 Hz, 1H), 7.57 (ddd, *J* = 7.7, 4.8, 1.2 Hz, 1H), 7.35 (dd, *J* = 8.9, 2.1 Hz, 1H), 6.45 (s, 1H), 4.17 (q, *J* = 7.1 Hz, 2H), 1.26 (t, *J* = 7.1 Hz, 3H).

^{13}C{^{1}H} NMR (101 MHz, DMSO-d_6) δ 160.1, 154.8, 153.5, 153.3, 152.4, 149.5, 143.0, 137.7, 128.0, 124.6, 124.4, 114.3, 112.9, 112.1, 104.7, 60.8, 14.4.

IR (solid-state ATR, cm^{-1}) 3310, 2992, 1731, 1701, 1628, 1589, 1429, 1357, 1219, 1070.

MS; HRMS (ESI+) m/z: [M-H]+ calcd for $C_{17}H_{15}N_2O_4$ 311.1026; found 311.1031.

7-julolidinyl-4-(pyridin-2-yl)-2*H*-chromen-2-one **5l**

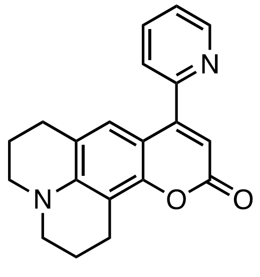

To a round bottomed flask, equipped with a magnetic stirrer bar, was added 8-hydroxyjulolidine (2.84 mmol, 0.538 g, 1.0 eq.), anhydrous zinc chloride (3.55 mmol, 0.483 g, 1.25 eq.), and ethyl 3-oxo-3-(pyridin-2-yl)propanoate (3.32 mmol, 0.680 g, 1.17 eq.) in

absolute ethanol (12 mL). The solution was heated to reflux for 18 h. Upon cooling, to the reaction was added HCl (0.5%, 15 mL), solution was then extracted with dichloromethane (3 x 25 mL). Organic phase was washed with water (3 x 25 mL), the combined organic extracts was dry-loaded on silica and purified by automated silica gel column chromatography (Rf = 0.33 hexane:EtOAc, 1:1, v/v) to afford the product **5l** as a yellow solid (0.409 g, 45%).

^{1}H NMR (400 MHz, Chloroform-*d*) δ 8.89 – 8.57 (m, 1H), 7.84 (td, *J* = 7.7, 1.8 Hz, 1H), 7.51 (d, *J* = 7.7 Hz, 1H), 7.40 (ddd, *J* = 7.7, 4.8, 1.2 Hz, 1H), 7.02 (s, 1H), 6.09 (s, 1H), 3.26 (td, *J* = 5.9, 2.4 Hz, 4H), 2.94 (t, *J* = 6.5 Hz, 2H), 2.68 (t, *J* = 6.3 Hz, 2H), 1.96 (m, 4H).

^{13}C{^{1}H} NMR (101 MHz, Chloroform-*d*) δ 162.6, 155.7, 154.0, 152.2, 149.7, 146.1, 137.1, (2C) 124.0, 123.8, 118.4, 108.4, 107.1, 107.0, 50.1, 49.7, 27.9, 21.6, 20.8, 20.6.

IR (solid-state ATR, cm^{-1}) 2933, 2842, 1702, 1610, 1584, 1513, 1370, 1325, 1310, 1245, 1049, 941, 908, 811, 727.

MS; HRMS (ESI+) m/z: [M-H]+ calcd for $C_{20}H_{19}N_2O_2$ 318.1368; found 318.1600.

6,7-methylenedioxy-4-(pyridin-2-yl)-2*H*-chromen-2-one **5n**

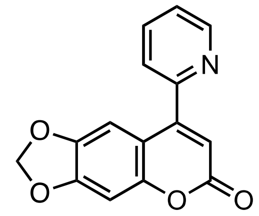

To a round bottomed flask, equipped with a magnetic stirrer bar, was added sesamol (0.457g, 3.31 mmol, 1.0 eq.), ethyl 3-oxo-3-(pyridin-2-yl)propanoate (0.767 g, 3.97 mmol, 1.2 eq.) and methanesulfonic acid (3 mL), resulting solution stirred overnight at room temperature. Reaction mixture then poured into ice water (50 mL) and extracted with ethyl acetate (3 x 50 mL). Combined organic fractions dried over sodium sulfate, filtered, and concentrated in vacuo. Crude solid product was recrystallized in minimal boiling methanol, product collected by filtration, washed with ice-cold methanol, dried to afford **5n** as a colourless crystalline solid (0.574 g, 65%).

^{1}H NMR (400 MHz, Chloroform-*d*) δ 8.79 (dd, *J* = 4.9, 0.9 Hz, 1H), 7.89 (td, *J* = 7.8, 1.8 Hz, 1H), 7.56 (d, *J* = 7.8 Hz, 1H), 7.45 (ddd, *J* = 7.7, 4.8, 1.1 Hz, 1H), 7.17 (s, 1H), 6.90 (s, 1H), 6.39 (s, 1H), 6.05 (s, 2H).

^{13}C{^{1}H} NMR (101 MHz, Chloroform-*d*) δ 161.3, 154.8, 153.4, 151.9, 151.4, 149.9, 145.1, 137.6, 124.4, 124.1, 113.2, 112.0, 104.9, 102.5, 98.6.

IR (solid-state ATR, cm^{-1}) 2960, 1702, 1589, 1361, 1226, 1075, 795.

MS; HRMS (ESI+) m/z: [M-H]+ calcd for $C_{15}H_{10}NO_4$ 268.0604; found 268.0607.

6,7-dimethoxy-4-(pyridin-2-yl)-2*H*-chromen-2-one **5o**

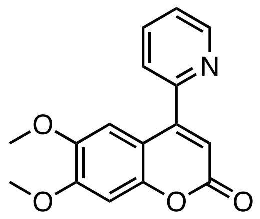

To a round bottomed flask, equipped with a magnetic stirrer bar, was added 3,4-dimethoxyphenol (0.510 g, 3.31 mmol, 1.0 eq.), ethyl 3-oxo-3-(pyridin-2-yl)propanoate (0.767 g, 3.97 mmol, 1.2 eq.) and methanesulfonic acid (3 mL), resulting solution stirred overnight at room temperature. Reaction mixture then poured into ice water (50 mL) and extracted with ethyl acetate (3 x 50 mL). Combined organic fractions dried over sodium sulfate, filtered, and concentrated *in vacuo*. Crude solid product was recrystallized in minimal boiling methanol, product collected by filtration, washed with ice-cold methanol, dried to afford **5o** as a colourless crystalline solid (0.160 g, 18%).

^{1}H NMR (400 MHz, Chloroform-*d*) δ 8.80 (ddd, *J* = 4.8, 1.8, 1.0 Hz, 1H), 7.90 (td, *J* = 7.7, 1.8 Hz, 1H), 7.60 (d, *J* = 7.9 Hz, 1H), 7.45 (ddd, *J* = 7.7, 4.8, 1.2 Hz, 1H), 7.27 (s, 1H), 6.90 (s, 1H), 6.39 (s, 1H), 3.96 (s, 3H), 3.79 (s, 3H).

^{13}C{^{1}H} NMR (101 MHz, Chloroform-*d*) δ 161.5, 154.9, 153.1, 153.0, 150.7, 149.8, 146.2, 137.6, 124.4, 124.1, 113.3, 110.6, 107.9, 100.2, 56.5, 56.4.

IR (solid-state ATR, cm^{-1}) 3014, 1716, 1618, 1549, 1411, 1379, 1230, 1146, 821.

MS; HRMS (ESI+) m/z: [M-H]+ calcd for $C_{16}H_{14}NO_4$ 284.0917; found 284.0919.

7-methoxy-4-(pyridin-2-yl)-*2H*-chromen-2-one tetracarbonyl manganese **6c**

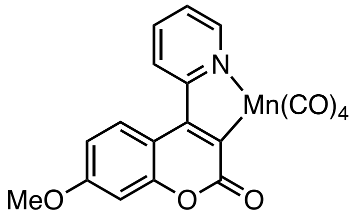

Synthesised according to general procedure for cyclomanganation of coumarins, **5c** (35.0 mg, 0.138 mmol, 1.0 eq.), benzyl manganese pentacarbonyl (39.0 mg, 0.138 mmol, 1.0 eq.), in dry

toluene. Product **6c** purified by removing solvent *in vacuo* to afford a yellow solid (56.8 mg, 98%).

^{1}H NMR (400 MHz, Methylene Chloride-d_2) δ 8.96 (dt, *J* = 5.5, 1.2 Hz, 1H), 8.36 (d, *J* = 8.3 Hz, 1H), 7.97 (td, *J* = 7.9, 1.6 Hz, 1H), 7.89 (d, *J* = 9.0 Hz, 1H), 7.34 (ddd, *J* = 7.2, 5.6, 1.4 Hz, 1H), 6.96 (d, *J* = 2.7 Hz, 1H), 6.86 (dd, *J* = 9.0, 2.7 Hz, 1H), 3.89 (s, 3H).

^{13}C{^{1}H} NMR (126 MHz, Methylene Chloride-d_2) δ 217.3, 214.3, 211.7, 170.2, 166.0, 164.4, 160.2, 155.8, 155.2, 153.5, 138.2, 124.7, 124.1, 122.7, 112.0, 110.8, 101.4, 55.7.

IR (solid-state ATR, cm^{-1}) 2075, 1966, 1929, 1677, 1609, 1580, 1503, 1470, 1354, 1284, 1259, 1205, 1144, 1124, 1108, 1032, 997, 959, 838, 809, 791, 758.

UV-Vis (in Dichloromethane, nm): λ_{max} 315.

MS; HRMS (ESI+) m/z: [M-H]+ calcd for $C_{19}H_{11}MnNO_7$ 419.9910; found 419.9909.

7-methyl-4-(pyridin-2-yl)-*2H*-chromen-2-one tetracarbonyl manganese **6e**

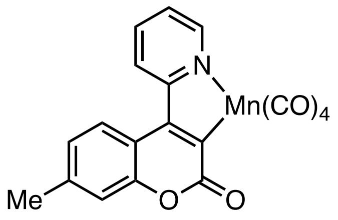

Synthesised according to general procedure for cyclomanganation of coumarins, **5e** (30.0 mg, 0.126 mmol, 1.0 eq.), benzyl manganese pentacarbonyl (36.1 mg, 0.126 mmol, 1.0 eq.), in dry toluene. Product **6e** purified by removing solvent *in vacuo* to afford a yellow solid (50.3 mg, 99%).

^{1}H NMR (500 MHz, Methylene Chloride-d_2) δ 8.96 (d, *J* = 5.6 Hz, 1H), 8.39 (d, *J* = 8.2 Hz, 1H), 7.98 (td, *J* = 7.9, 1.7 Hz, 1H), 7.86 (d, *J* = 8.2 Hz, 1H), 7.35 (ddd, *J* = 7.2, 5.5, 1.4 Hz, 1H), 7.24 (s, 1H), 7.11 (dd, *J* = 8.3, 1.8 Hz, 1H), 2.45 (s, 3H).

^{13}C{^{1}H} NMR (126 MHz, Methylene Chloride-d_2) δ 217.8, 214.9, 212.2, 174.6, 166.5, 165.0, 155.8, 154.7, 154.0, 140.4, 138.7, 125.4, 124.8, 124.6, 122.0, 118.2, 116.1, 21.6.

IR (solid-state ATR, cm^{-1}) 2083, 1997, 1950, 1887, 1738, 1712, 1697, 1487, 1146.

UV-Vis (in Dichloromethane, nm): λ_{max} 317.

MS; HRMS (ESI+) m/z: [M-H]+ calcd for $C_{19}H_{11}MnNO_6$ 403.9961; found 403.9942.

7-methoxy-6-oxo-8-phenyl-6H-chromeno[4,3-a]quinolizin-9-ium tricarbonyl manganese **7c**

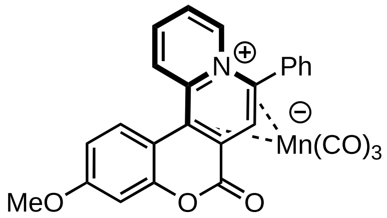

Synthesised according to general procedure for reductive elimination reaction in absence of TMNO from **6c** (16.4 mg, 0.0391 mmol, 1.0 eq.), phenylacetylene (6.5 µL, 0.0587 mmol, 1.5 eq.), in dry dibutyl ether. Product purified by dissolving in 1 mL dichloromethane and precipitated out with 20 mL pentane, solid filtered to afford **7c** as a dark brown solid (18.6 mg, 94%).

^{1}H NMR (400 MHz, DMSO-d_6) δ 7.84 (t, *J* = 7.8 Hz, 1H), 7.70 (d, *J* = 7.6 Hz, 1H), 7.66 (d, *J* = 6.4 Hz, 1H), 7.57 – 7.42 (m, 5H), 7.37 (t, *J* = 7.2 Hz, 1H), 7.13 (t, *J* = 6.9 Hz, 1H), 6.87 (dd, *J* = 8.7, 2.7 Hz, 1H), 6.84 (s, 1H), 6.82 (d, *J* = 2.6 Hz, 1H), 3.78 (s, 3H).

^{13}C{^{1}H} NMR (101 MHz, DMSO-d_6) δ 164.4, 158.2, 156.6, 149.6, 141.7, 139.4, 136.5, 131.9, 130.0, 129.9, 128.9, 128.0, 126.6, 122.5, 121.8, 115.4, 111.9, 102.8, 92.4, 84.4, 78.9, 61.0, 56.1.

IR (solid-state ATR, cm^{-1}) 2921, 2852, 1976, 1879, 1732, 1605, 1567, 1513, 1502, 1490, 1439, 1341, 1219, 1129, 1036, 966.

MS; HRMS (ESI+) m/z: [M-Na]+ calcd for $C_{26}H_{16}MnNNaO_6$ 516.0250; found 516.0252.

Synthesised according to general procedure for Photo-induced Reductive Elimination Reaction from **6c** (16.4 mg, 0.0391 mmol, 1.0 eq.), phenylacetylene (6.5 µL, 0.0587 mmol, 1.5 eq.), in dry diethyl ether. Solid filtered to afford **7c** as a dark brown solid (18.9 mg, 96%).

(*E*)-7-diethylamino-4-(pyridin-3-yl)-3-styryl-2*H*-chromen-2-one **7a’**

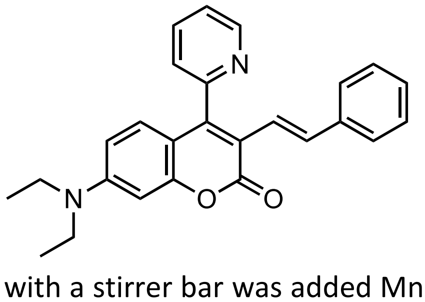

To a microwave vial equipped with a stirrer bar was added $MnBr(CO)_5$ (7.0 mg, 0.025 mmol, 10 mol%), dicyclohexylamine (10 µL, 0.025 mmol, 10 mol%), propanoic acid (4 µL, 0.05 mmol,

20 mol%), 7-diethylamino-4-(2-pyridyl)coumarin **5a** (73.8 mg, 0.25 mmol, 1 eq.) and phenylacetylene (27 µL 0.25 mmol, 1 eq.). *n*Bu_2O (0.6 mL) was then added and the solution was deoxygenated with argon balloon before heating at 100 °C for 13 hours. Resulting solution was filtered through a pad of Celite® with chloroform (25 mL). Crude solution concentrated *in vacuo* then purified *via* automated column chromatography, product **7a'** (Rf = 0.20 35% EtOAc in hexane v/v). Product was a yellow-green solid. (49.6 mg, 50%).

^{1}H NMR (400 MHz, Chloroform-*d*) δ 8.94 – 8.81 (m, 1H), 7.91 (td, *J* = 7.7, 1.7 Hz, 1H), 7.74 (d, *J* = 16.1 Hz, 1H), 7.47 (ddd, *J* = 7.7, 4.9, 1.2 Hz, 1H), 7.42 (d, *J* = 7.7 Hz, 1H), 7.29 (s, 1H), 7.24 (s, 3H), 7.22 – 7.16 (m, 1H), 6.78 (d, *J* = 9.0 Hz, 1H), 6.62 – 6.56 (m, 2H), 6.50 (d, *J* = 7.7 Hz, 1H), 3.43 (q, *J* = 7.1 Hz, 4H), 1.22 (t, *J* = 7.1 Hz, 6H).

^{13}C{^{1}H} NMR (101 MHz, Chloroform-*d*) δ 161.2, 155.3, 155.1, 150.4, 150.3, 149.2, 138.4, 136.8, 133.4, 128.6, 128.1, 127.5, 126.7, 125.2, 123.4, 121.9, 115.6, 109.4, 109.2, 97.3, 45.0, 12.6.

IR (solid-state ATR, cm^{-1}) 2973, 2924, 1698, 1601, 1559, 1415, 1340, 1215, 1185.

MS; HRMS (ESI+) m/z: [M-H]+ calcd for $C_{26}H_{25}N_2O_2$ 397.1911; found 397.1922.

(*E*)-7-methoxy-4-(pyridin-3-yl)-3-styryl-2*H*-chromen-2-one **7c'**

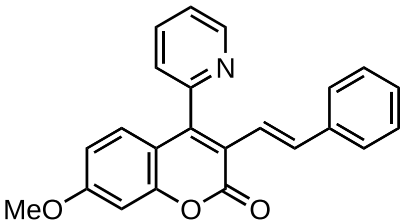

To a microwave vial equipped with a stirrer bar was added $MnBr(CO)_5$ (7.0 mg, 0.025 mmol, 10 mol%), dicyclohexylamine (10 µL, 0.025 mmol, 10 mol%), propanoic acid (4 µL, 0.05 mmol, 20 mol%), 7-methoxy-4-(2-pyridyl)coumarin **5c** (63.0 mg, 0.25 mmol, 1 eq.) and phenylacetylene (27 µL 0.25 mmol, 1 eq.). *n*Bu_2O (0.6 mL) was then added and the solution was deoxygenated with argon balloon before heating at 100 °C for 13 hours. Resulting solution was filtered through a pad of Celite® with chloroform (25 mL). Crude solution concentrated *in vacuo* then purified *via* automated column chromatography, product **7c'** (Rf = 0.05 20% EtOAc in hexane v/v). Product was a yellow-green solid (27.5 mg, 31%).

^{1}H NMR (600 MHz, Chloroform-*d*) δ 8.90 (d, *J* = 4.5 Hz, 1H), 7.93 (t, *J* = 7.7 Hz, 1H), 7.80 (d, *J* = 16.1 Hz, 1H), 7.52 – 7.47 (m, 1H), 7.43 (d, *J* = 7.7 Hz, 1H), 7.28 – 7.25 (m, 4H), 7.24 – 7.20 (m,

1H), 6.91 (s, 1H), 6.91 (d, *J* = 6.9 Hz, 1H), 6.76 (dd, *J* = 8.9, 2.5 Hz, 1H), 6.61 (d, *J* = 16.1 Hz, 1H), 3.90 (s, 3H).

$^{13}C\{^{1}H\}$ NMR (101 MHz, Chloroform-*d*) δ 162.6, 160.5, 154.5, 154.4, 150.5, 148.4, 137.8, 137.1, 135.6, 128.7, 128.1(2), 128.0(9), 126.9, 125.2, 123.7, 121.2, 119.1, 113.8, 112.9, 100.5, 55.9.

IR (solid-state ATR, cm^{-1}) 3053, 3024, 2937, 2842, 1716, 1609, 1582, 1534, 1507, 1489, 1466, 1448, 1429, 1378, 1336, 1294, 1254, 1201, 1163, 1140, 1125, 1068, 1027, 992, 959, 932, 887, 852, 809, 790, 746.

MS; HRMS (ESI+) m/z: [M-H]+ calcd for $C_{23}H_{18}NO_3$ 356.1281; found 356.1286.

(*E*)-7-methyl-4-(pyridin-3-yl)-3-styryl-2*H*-chromen-2-one **7e'**

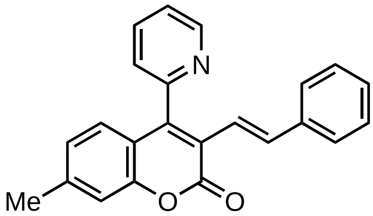

To a microwave vial equipped with a stirrer bar was added $MnBr(CO)_5$ (7.00 mg, 0.025 mmol, 10 mol%), dicyclohexylamine (10 µL, 0.025 mmol, 10 mol%), propanoic acid (4 µL, 0.05 mmol, 20 mol%), 7-methyl-4-(2-pyridyl)coumarin **5e** (59.3 mg, 0.25 mmol, 1 eq.) and phenylacetylene (27 µL 0.25 mmol, 1 eq.). *n*Bu_2O (0.6 mL) was then added and the solution was deoxygenated with argon balloon before heating at 100 °C for 13 hours. Resulting solution was filtered through a pad of Celite® with chloroform (25 mL). Crude solution concentrated *in vacuo* then purified *via* automated column chromatography, product **7e'** (Rf = 0.1 35% EtOAc in hexane v/v). Product was a yellow-green solid (39.0 mg, 46%)

^{1}H NMR (400 MHz, Chloroform-*d*) δ 8.92 – 8.87 (m, 1H), 7.99 – 7.90 (m, 1H), 7.82 (d, *J* = 16.2 Hz, 1H), 7.53 – 7.46 (m, 1H), 7.42 (d, *J* = 7.8 Hz, 1H), 7.30 – 7.19 (m, 7H), 6.87 (d, *J* = 8.1 Hz, 1H), 6.62 (d, *J* = 16.2 Hz, 1H), 2.45 (s, 3H).

$^{13}C\{^{1}H\}$ NMR (101 MHz, Chloroform-*d*) δ 160.4, 154.4, 152.7, 150.5, 148.1, 142.7, 137.7, 137.1, 136.3, 128.7, 128.2, 127.0, 126.8, 125.7, 125.2, 123.7, 121.1(4), 121.0(8), 117.8, 116.8, 21.8.

IR (solid-state ATR, cm^{-1}) 2914, 1683, 1594, 1565, 1491, 1445, 1366, 1330, 1310, 1158, 1072, 987.

MS; HRMS (ESI+) m/z: [M-H]+ calcd for $C_{23}H_{18}NO_2$ 340.1332; found 340.1335.

(*E*)-7-Ethyl carbamate-4-(pyridin-3-yl)-3-styryl-2*H*-chromen-2-one **7k’**

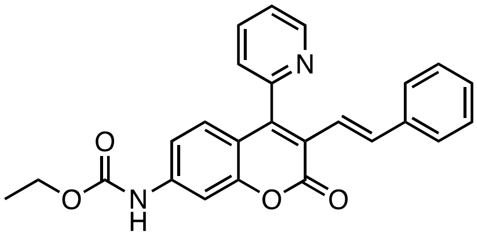

To a microwave vial equipped with a stirrer bar was added MnBr(CO)$_5$ (7.00 mg, 0.025 mmol, 10 mol%), dicyclohexylamine (10 µL, 0.025 mmol, 10 mol%), propanoic acid (4 µL, 0.05 mmol, 20 mol%), 7-Ethyl carbamate-4-(pyridin-2-yl)coumarin **5k** (103.1 mg, 0.25 mmol, 1 eq.) and phenylacetylene (27 µL 0.25 mmol, 1 eq.). *n*Bu$_2$O (0.6 mL) was then added and the solution was deoxygenated with argon balloon before heating at 100 °C for 13 hours. Resulting solution was filtered through a pad of Celite® with chloroform (25 mL). Crude solution concentrated *in vacuo* then purified *via* automated column chromatography, product **7k’** (Rf = 0.3 65% EtOAc in hexane v/v). Product was a yellow solid (40.2 mg, 39%).

^{1}H NMR (400 MHz, Chloroform-*d*) δ 8.92 – 8.88 (m, 1H), 7.95 (td, *J* = 7.7, 1.8 Hz, 1H), 7.81 (d, *J* = 16.2 Hz, 1H), 7.56 (d, *J* = 2.1 Hz, 1H), 7.51 (ddd, *J* = 7.7, 4.9, 1.2 Hz, 1H), 7.44 (d, *J* = 7.7 Hz, 1H), 7.28 (d, *J* = 5.1 Hz, 2H), 7.25 – 7.13 (m, 4H), 6.90 (d, *J* = 8.7 Hz, 1H), 6.60 (d, *J* = 16.2 Hz, 1H), 4.25 (q, *J* = 7.1 Hz, 2H), 1.32 (t, *J* = 7.1 Hz, 3H), N*H* not observed.

^{13}C{^{1}H} NMR (101 MHz, Chloroform-*d*) δ 160.3, 154.3, 153.5, 153.1, 150.5, 147.9, 141.3, 137.7, 137.2, 136.0, 128.7, 128.2, 127.8, 126.9, 125.3, 123.8, 121.1, 120.1, 115.6, 114.8, 105.5, 61.9, 14.6.

IR (solid-state ATR, cm^{-1}) 2981, 1719, 1623, 1601, 1509, 1422, 1211, 1063.

MS; HRMS (ESI+) m/z: [M-H]+ calcd for $C_{25}H_{21}N_2O_4$ 413.1496; found 413.1495.

(*E*)-7-julolidinyl-4-(pyridin-2-yl)-3-styryl-2*H*-chromen-2-one **7l’**

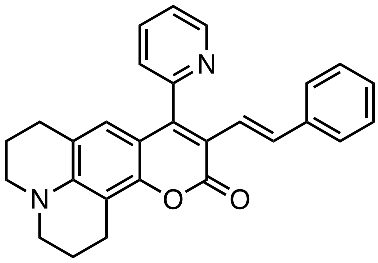

To a microwave vial equipped with a stirrer bar was added MnBr(CO)$_5$ (7.0 mg, 0.025 mmol, 10 mol%), dicyclohexylamine (10 µL, 0.025 mmol, 10 mol%), propanoic acid (4 µL, 0.05 mmol,

20mol%), 7-julolidinyl-4-(pyridin-2-yl)-2*H*-chromen-2-one **5l** (79.6 mg, 0.25 mmol, 1 eq.) and phenylacetylene (27 µL 0.25 mmol, 1 eq.). *n*Bu_2O (0.6 mL) was then added and the solution was deoxygenated with argon balloon before heating at 100 °C for 13 hours. Resulting solution was filtered through a pad of Celite® with chloroform (25 mL). Crude solution concentrated *in vacuo* then purified *via* automated column chromatography, product **7l’** (Rf = 0.55 45% EtOAc in hexane v/v). Product was an orange solid (48.3 mg, 45%).

^{1}H NMR (400 MHz, Chloroform-*d*) δ 8.84 (d, *J* = 5.0 Hz, 1H), 7.86 (td, *J* = 7.7, 1.8 Hz, 1H), 7.66 (d, *J* = 16.1 Hz, 1H), 7.43 (ddd, *J* = 7.7, 4.9, 1.2 Hz, 1H), 7.35 (d, *J* = 7.7 Hz, 1H), 7.22 – 7.18 (m, 4H), 7.17 – 7.10 (m, 1H), 6.52 (d, *J* = 16.1 Hz, 1H), 6.29 (s, 1H), 3.31 – 3.19 (m, 4H), 2.99 – 2.92 (m, 2H), 2.59 (s, 2H), 1.99 (p, *J* = 6.3 Hz, 2H), 1.89 (p, *J* = 6.1 Hz, 2H).

^{13}C{^{1}H} NMR (101 MHz, Chloroform-*d*) δ 161.2, 155.3, 150.3, 150.1, 149.4, 145.7, 138.4, 136.7, 132.7, 128.4, 127.2, 126.5, 125.0, 124.0, 123.1, 122.1, 118.6, 114.6, 109.1, 106.2, 50.0, 49.6, 27.7, 21.5, 20.7, 20.4.

IR (solid-state ATR, cm^{-1}) 2935, 1700, 1605, 1515, 1476, 1438, 1370, 1308, 1273, 1165, 1152, 1047, 992, 908, 803, 725, 644.

MS; HRMS (ESI+) m/z: [M-Na]+ calcd for $C_{28}H_{24}N_2NaO_2$ 433.1736; found 443.1742.

(*E*)-6,7-dimethoxy-4-(pyridin-3-yl)-3-styryl-2*H*-chromen-2-one **7o’**

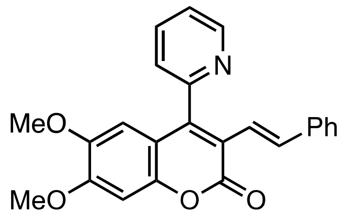

To a microwave vial equipped with a stirrer bar was added $MnBr(CO)_5$ (7.0 mg, 0.025 mmol, 10 mol%), dicyclohexylamine (10 µL, 0.025 mmol, 10 mol%), propanoic acid (4 µL, 0.05 mmol, 20 mol%), 6,7-dimethoxy-4-(2-pyridyl)coumarin **5o** (63.0 mg, 0.25 mmol, 1 eq.) and phenylacetylene (27 µL 0.25 mmol, 1 eq.). *n*Bu_2O (0.6 mL) was then added and the solution was deoxygenated with argon balloon before heating at 100 °C for 13 hours. Resulting solution was filtered through a pad of Celite® with chloroform (25 mL). Crude solution concentrated *in vacuo* then purified *via* automated column chromatography, product **7o’** (Rf = 0.20 25% EtOAc in hexane v/v). Product was a yellow solid (27.9 mg, 29%).

^{1}H NMR (400 MHz, Chloroform-*d*) δ 8.92 (ddd, *J* = 4.9, 1.8, 1.0 Hz, 1H), 7.96 (td, *J* = 7.7, 1.8 Hz, 1H), 7.82 (d, *J* = 16.2 Hz, 1H), 7.51 (ddd, *J* = 7.7, 4.9, 1.2 Hz, 1H), 7.46 (d, *J* = 7.8 Hz, 1H), 7.30

– 7.27 (m, 3H), 7.26 – 7.18 (m, 2H), 6.93 (s, 1H), 6.61 (d, *J* = 16.2 Hz, 1H), 6.38 (s, 1H), 3.99 (s, 3H), 3.69 (s, 3H).

^{13}C{^{1}H} NMR (101 MHz, Chloroform-*d*) δ 160.5, 154.5, 152.7, 150.4, 148.6, 148.0, 146.2, 137.7, 137.0, 135.5, 128.5, 128.0, 126.8, 125.2, 123.6, 121.2, 119.3, 112.6, 107.4, 99.6, 56.4, 56.2.

IR (solid-state ATR, cm^{-1}) 2935, 1709, 1612, 1584, 1513, 1431, 1467, 1392, 1277,1146, 1077, 992.

MS; HRMS (ESI+) m/z: [M-H]+ calcd for $C_{24}H_{20}NO_4$ 386.1387; found 386.1391.

Failed 4-(pyridin-2-yl)-2*H*-chromen-2-one Catalytic C–H Substrates (reactions with phenyl acetylene)

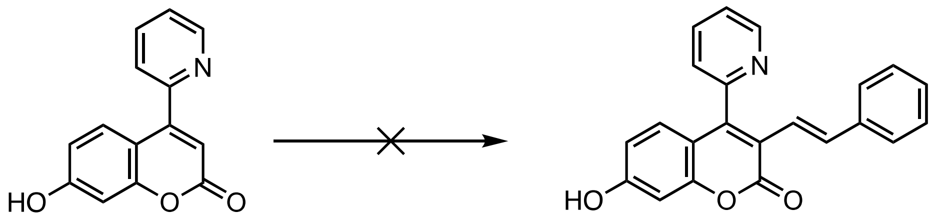

5m

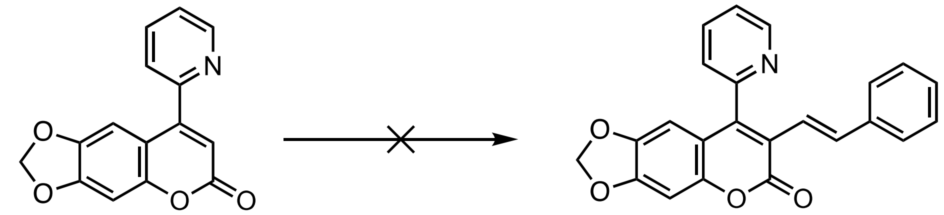

5n

4 Electronic spectra

Compounds **2a-g** recorded electronic spectra between 700-200 nm, in dichloromethane, λ_{max} taken from peak position at 2.39 x 10^{-5} mol dm^{-3}, using 1 cm quartz cuvettes on a JASCO V-560 UV-Visible spectrophotometer.

Table S1 Supplementary Table (top), λ_{max}, and Molar Absorption Coefficients, (bottom) spectra in dichloromethane at 2.39 x 10^{-5} mol dm^{-3}.

Compound	λ_{max} /nm	ε / M^{-1} cm^{-1}
**2a**	442	32,600
**2b**	343	7,600
**2c**	349	22,800
**2d**	338	14,900
**2e**	340	7,800
**2f**	322	10,800
**2g**	338	4,000

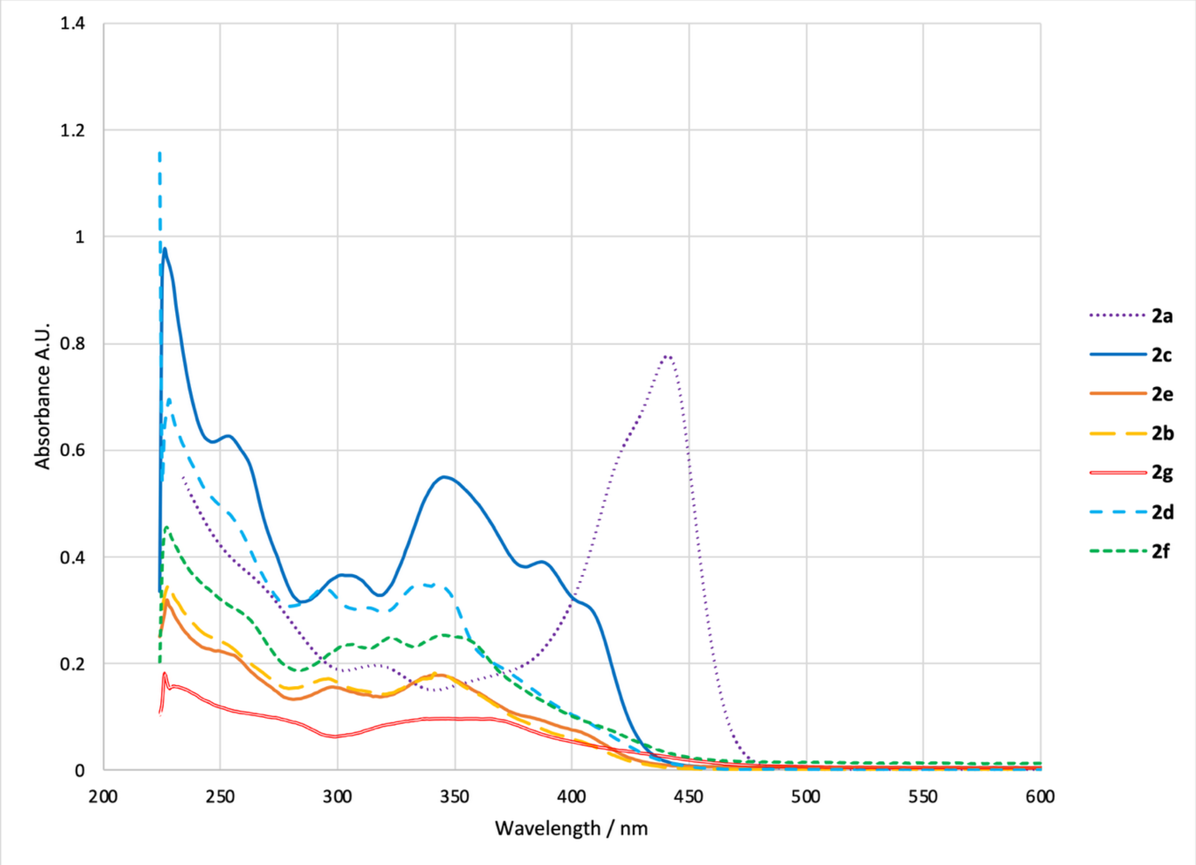

5 NMR Studies

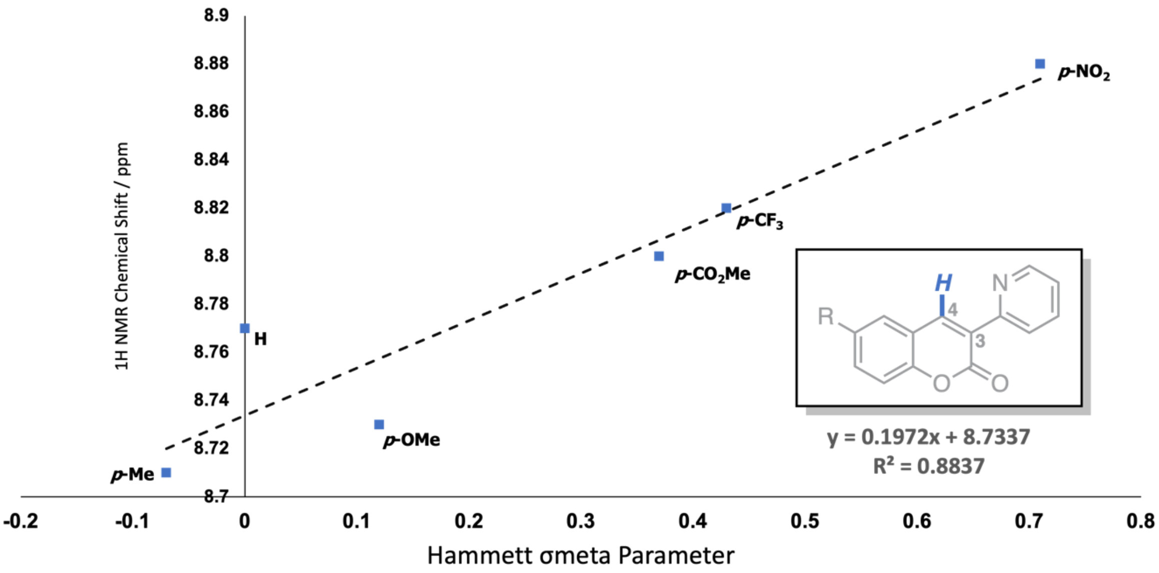

Figure S1 Hammett Plot of Hammett σ_{meta} values against coumarin C4-*H* ^{1}H NMR shift in $CDCl_3$ at 298 K, (400 MHz). General structures shown inset.

6 DFT Computational Calculations Using Density Functional Theory (DFT) Methods

7 Atomic Contribution, Mulliken Charges, and Electrostatic Potential Mapping

Atomic contribution, electrostatic potential mapping, and Mulliken charges were obtained from calculations carried out using the Gaussian16 package. Geometry optimisations were performed at the BP86/SV(P) level and corrected for dispersion effects using Grimme's D3 method with BJ dampening, in toluene using the IEFPCM solvent model, followed by frequency calculations at the same level. All minima were confirmed as such by the absence of imaginary frequencies. The atomic contributions were obtained *via* a population analysis through the summing of each atomic orbital on a specific atom for the relevant molecular orbital.

8 DFT xyz Coordinates

1c

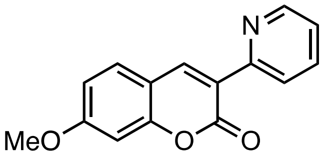

C	1.58831	8.14776	4.21936
C	2.76011	7.51598	4.84307
C	4.03078	7.32030	4.09135
C	4.20755	7.72108	2.74422
H	3.37641	8.20068	2.21299
C	5.44561	7.49218	2.12547
H	5.60070	7.79736	1.07854
C	6.47461	6.87472	2.85148
H	7.46053	6.67671	2.40433
C	6.20610	6.51130	4.18512
H	6.98782	6.02209	4.79532
C	2.67875	7.09447	6.15917
C	1.49878	7.24877	6.94453
C	1.36913	6.83305	8.30005
H	2.23073	6.35594	8.79211
C	0.18288	7.02257	8.99467
C	-0.92945	7.64111	8.35284
O	-2.03960	7.78144	9.11399
C	-0.83503	8.06363	7.01202
C	0.37521	7.86198	6.32888
N	5.03136	6.72313	4.78828

O	0.44380	8.27813	5.03663
O	1.46952	8.57739	3.08462
H	-1.66438	8.54347	6.47778
C	-3.19135	8.39228	8.53606
H	-3.96423	8.40289	9.32597
H	-3.56441	7.81105	7.66466
H	-2.97935	9.43562	8.21529
H	0.06832	6.70710	10.04161
H	3.57788	6.62529	6.58957

2c

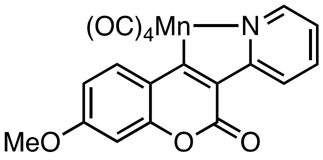

C	3.48183	4.45287	5.84173
C	5.98889	5.00483	6.81615
C	4.88704	7.46576	7.33894
C	4.00321	5.20651	8.27268
C	1.62358	8.26779	4.31532
C	2.72229	7.48114	4.88275
C	3.95026	7.28991	4.11499
C	4.20370	7.80879	2.82156
H	3.41370	8.40509	2.34929
C	5.42590	7.55329	2.19598
H	5.62556	7.95549	1.19092
C	6.39027	6.78035	2.86466
H	7.36758	6.55074	2.41677
C	6.08421	6.29431	4.13841
H	6.81357	5.68613	4.69007
C	2.65725	6.88825	6.15351
C	1.44957	7.07503	6.92181
C	1.20650	6.55478	8.22682
H	1.98368	5.95930	8.71370
C	0.01891	6.77386	8.90880
C	-1.01622	7.54324	8.31015
O	-2.13906	7.70530	9.04599
C	-0.82675	8.08025	7.02446
C	0.38658	7.84283	6.35718
Mn	4.38527	5.84272	6.63657
N	4.90390	6.53632	4.75169
O	2.86510	3.60869	5.34261
O	7.00711	4.45714	6.96140
O	5.14914	8.50600	7.77637
O	3.87764	4.73584	9.33455

O	0.48924	8.39768	5.12055
O	1.56795	8.82549	3.22904
H	-1.58592	8.68242	6.50997
C	-3.21131	8.46793	8.49513
H	-4.01270	8.46838	9.25616
H	-3.59486	8.00944	7.55762
H	-2.89965	9.51509	8.28887
H	-0.14710	6.36287	9.91487

5c

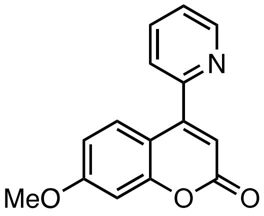

O	1.01861	0.15652	-0.21186
C	2.22519	-0.00783	-0.22027
C	2.93174	-1.27803	-0.22686
C	4.30691	-1.38203	-0.23855
C	4.92478	-2.74161	-0.23004
N	6.01785	-2.94644	-1.00001
C	6.55043	-4.17831	-1.03727
C	6.04031	-5.27011	-0.31264
C	4.91932	-5.05509	0.50445
C	4.35166	-3.77440	0.54980
C	5.10067	-0.16355	-0.24570
C	6.51712	-0.10651	-0.23427
C	7.20646	1.10671	-0.21634
C	6.48261	2.32798	-0.21055
O	7.05698	3.55609	-0.19571
C	8.47892	3.64699	-0.18563
C	5.07400	2.30682	-0.21800
C	4.39996	1.08121	-0.23362
O	3.03532	1.14089	-0.22778
H	2.28801	-2.16807	-0.25152
H	7.43888	-4.30525	-1.68199
H	6.51897	-6.25864	-0.38186
H	4.49385	-5.87357	1.10562
H	3.48577	-3.56575	1.19493
H	7.07472	-1.05094	-0.26750
H	8.30430	1.10051	-0.21120
H	8.71806	4.72622	-0.17493
H	8.92502	3.18362	-1.09289
H	8.91319	3.16830	0.71949
H	4.50580	3.24682	-0.20816

6c

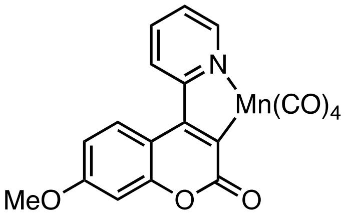

C	7.70391	3.28413	6.69660
C	8.64849	1.56111	5.01992
C	7.76565	2.99991	3.05466
C	9.57893	3.95345	4.83392
C	7.56618	6.23367	4.78617
H	8.66382	6.21502	4.74011
C	6.87894	7.44745	4.90040
H	7.44228	8.39105	4.92519
C	5.48185	7.41350	5.01974
H	4.90424	8.33889	5.16466
C	4.82735	6.17790	4.95930
H	3.74102	6.12601	5.09043
C	5.56650	4.98787	4.77874
C	5.01559	3.62395	4.71150
C	3.60798	3.29382	4.50905
C	2.57657	4.15655	4.04113
H	2.81732	5.18163	3.73369
C	1.26098	3.72516	3.89982
H	0.47702	4.40083	3.52856
C	0.90813	2.38781	4.21805
C	1.90848	1.48733	4.62331
H	1.71072	0.42840	4.83140
C	3.23505	1.94075	4.73619
C	5.54629	1.23408	5.01567
C	5.96846	2.62528	4.87397
C	-0.80088	0.72654	4.36205
H	-1.89266	0.68628	4.19382
H	-0.58225	0.45468	5.41828
H	-0.29966	-0.00308	3.68848
Mn	7.91985	3.21642	4.87285
N	6.94030	5.03809	4.74805
O	7.51827	3.29957	7.84078
O	9.23040	0.55964	5.11018
O	7.61888	2.84063	1.91597
O	10.64878	4.41938	4.81234
O	-0.40094	2.06327	4.07372
O	4.15919	0.99231	5.05871
O	6.26099	0.25506	5.15274

9 Atomic Contributions of the HOMO's and Mulliken Charges

For clarity, respective compound numbering has been assigned to non-hydrogen atoms only for **1c** and **5c**, and the contributions of the hydrogen atoms are not shown.

1c

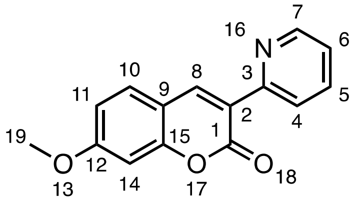

(Numbering assigned to non-hydrogen atoms)

Table S2 Atom Numbering, Type, Normalised Contribution, and Mulliken Charge for **1c**.

Atom Number	Atom Type	Normalized Contribution	Mulliken Charge
1	C	0.03	0.175918
2	C	0.10	-0.033133
3	C	0.04	-0.029296
4	C	0.06	-0.029501
5	C	0.01	0.041581
6	C	0.06	-0.022085
7	C	0.03	0.008469
8	C	0.04	0.001265
9	C	0.08	0.067424
10	C	0.03	-0.046189
11	C	0.07	-0.009048
12	C	0.07	0.187557
13	O	0.09	-0.262534
14	C	0.04	-0.120583
15	C	0.06	0.137106
16	N	0.04	-0.100003
17	O	0.03	-0.201774
18	O	0.08	-0.177687
19	C	0.02	0.092610

5c

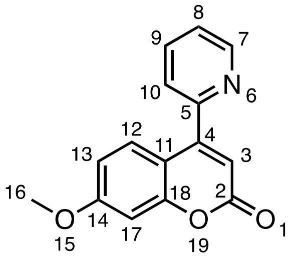

(Numbering assigned to non-hydrogen atoms)

Table S3 Atom Numbering, Type, Normalised Contribution, and Mulliken Charge for **5c**.

Atom Number	Atom Type	Normalized Contribution	Mulliken Charge
1	O	0.07	-0.188084
2	C	0.02	0.181296
3	C	0.09	-0.174815
4	C	0.05	0.169547
5	C	0.04	-0.05411
6	N	0.05	-0.104594
7	C	0.02	0.021940
8	C	0.03	-0.019995
9	C	0.02	0.037605
10	C	0.03	-0.006131
11	C	0.09	0.046686
12	C	0.05	-0.040502
13	C	0.07	-0.075808
14	C	0.07	0.200559
15	O	0.09	-0.266639
16	C	0.02	0.094507
17	C	0.05	-0.073675
18	C	0.04	0.120332
19	O	0.02	-0.206715

10 Electrostatic Potential Maps

Electrostatic potential maps shown at 0.0004 isovalue.

1c

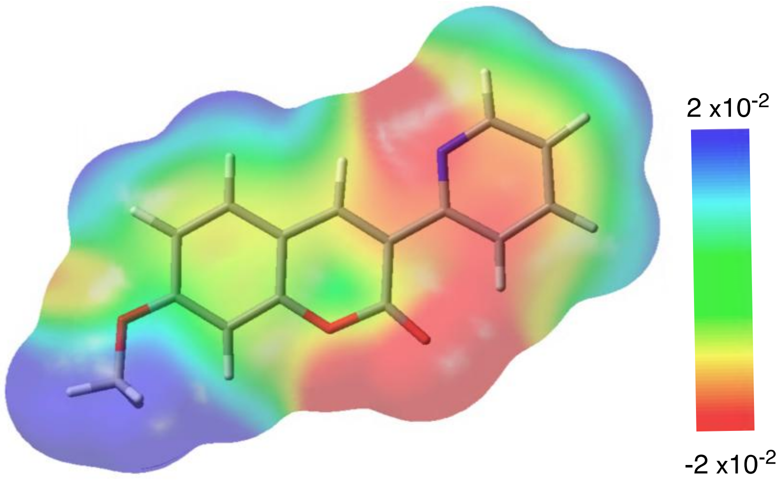

Figure S2 Electrostatic Potential Surface of **1c**, isovalue: 0.0004.

2c

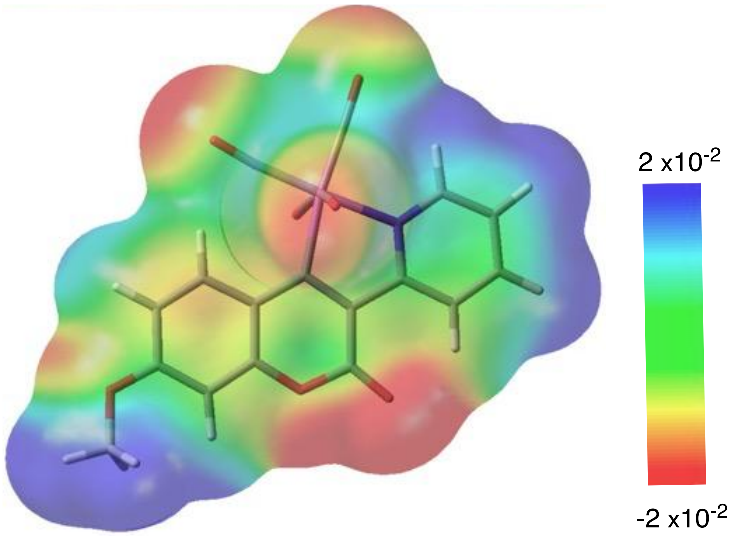

Figure S3 Electrostatic Potential Surface of **2c**, isovalue: 0.0004.

5c

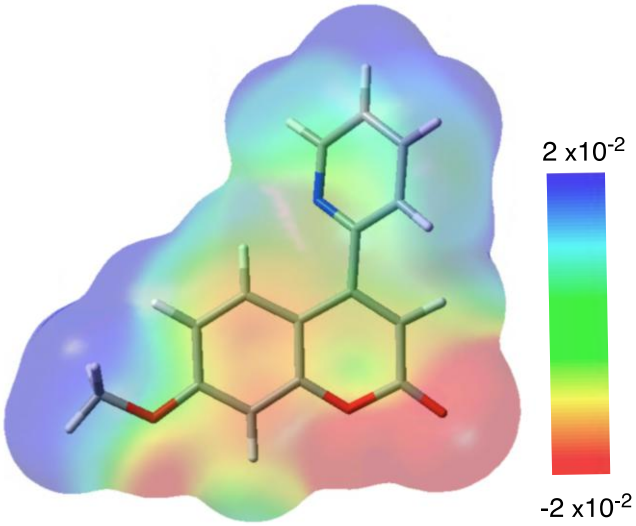

Figure S4 Electrostatic Potential Surface of **5c**, isovalue: 0.0004.

6c

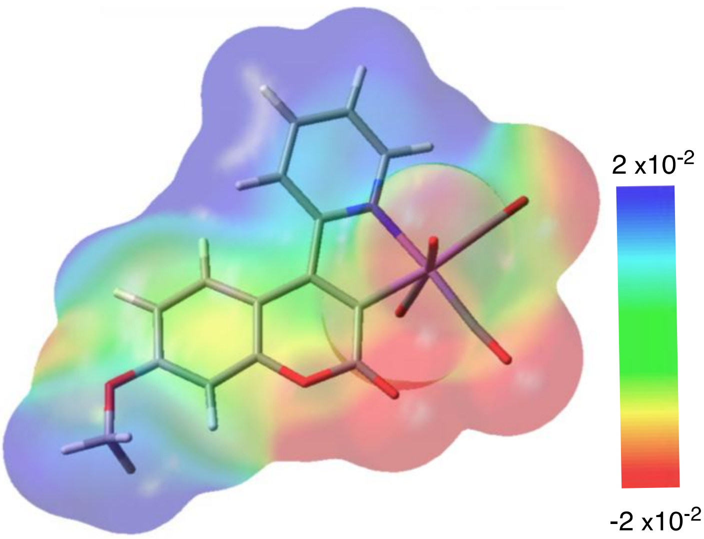

Figure S5 Electrostatic Potential Surface of **6c**, isovalue: 0.0004.

11 Coumarin Reaction Pathway DFT Methodology

Calculations to determine the relative energies of intermediates and transition states were performed using the TURBOMOLE V6.4 package using the resolution of identity (RI) approximation.[8–14] Initial optimisations were performed at the (RI-)BP86/SV(P) level, followed by frequency calculations at the same level. All minima were confirmed as such by the absence of imaginary frequencies. Single-point energies were then performed on the (RI-)BP86/SV(P) optimised geometries using the hybrid PBE0 functional and the flexible def2-TZVPP basis set. Energies, xyz coordinates and the first 50 lines of the vibrational spectra are presented. Solvation effects were modelled using COMSO[15] using the dielectronic constant of

2.38 for toluene and energies were corrected for dispersion using Grimme's D3-method[16] with Becke-Johnson dampening.[17]

12 Molecular NBO DFT Methodology

NBO calculations were performed with the NBO 7.0 software package.[18] Wavefunctions for analysis were generated at the PBE0/def2-TZVP level in Gaussian 16[19] using the (RI-)BP86/SV(P) optimised geometries. The resulting NBOs were visualised in Multiwfn,[20] the resulting *.cub files were then exported to GaussView[21] to create the images in the manuscript.

13 Coumarin Reaction Pathway DFT xyz Coordinates

$2c_{PhC \equiv CH}$

```
SCF Energy (au) BP86/SV(P)                   -2656.578050113
SCF Energy (au) PBE0/def2-TZVPP              -2656.119801066
SCF Energy (au) PBE0/def2-TZVPP              -2656.1297654432 (Toluene
Correction)
Zero Point Energy (au)                       0.3475438
Chemical Potential (kJ mol-1)                745.06
Dispersion Correction (au) PBE0/def2-TZVPP  -0.07516961

xyz coordinates
50

Mn    0.9292342    0.9598415    0.3660112
C     1.3591193    0.8792632    2.1136552
C     2.5505361    0.3947613   -0.1549347
C     1.5662001    2.6551499    0.2201419
O     2.0236367    3.7268963    0.1760045
O     3.6223911    0.1352886   -0.5421790
O     1.6411285    0.8158934    3.2405068
C    -1.9136697    0.6763725    1.1122512
C    -1.3990256    2.9307878    0.6940353
C    -2.7137126    3.3418571    0.9345705
C    -3.6642872    2.3636236    1.2725418
C    -3.2654404    1.0279927    1.3599619
C    -1.3586725   -0.6729354    1.1981174
C     0.0132336   -0.8234506    0.9353850
C     0.5838904   -2.1463699    1.0951899
C    -0.2768549   -3.2312066    1.4390382
O    -1.6096086   -3.0522202    1.6245906
C    -2.2328038   -1.8000995    1.5460219
H    -0.6285717    3.6684007    0.4242690
H    -2.9744319    4.4084995    0.8561321
H    -4.7131462    2.6402721    1.4697029
H    -3.9658511    0.2237958    1.6202705
N    -0.9985486    1.6422449    0.7758041
C     0.2760788    2.5301576   -2.6047423
C     1.4542925    3.1752095   -3.0611008
C     1.3682688    4.3219355   -3.8614158
C     0.1132811    4.8507301   -4.2143566
C    -1.0605915    4.2211105   -3.7647840
C    -0.9857169    3.0713263   -2.9651787
H     2.4376486    2.7627782   -2.7861336
```

H	2.2922778	4.8100646	-4.2125903
H	0.0509289	5.7555075	-4.8411712
H	-2.0473872	4.6280216	-4.0412621
H	-1.9032697	2.5724194	-2.6160245
C	0.3362871	1.3247250	-1.8157836
C	0.2016907	0.1211757	-1.4571365
H	-0.0294907	-0.9181252	-1.6912720
C	1.9648347	-2.4792887	0.9598089
C	0.1822885	-4.5497423	1.6130610
O	-3.4326589	-1.7928946	1.7635206
C	1.5512941	-4.8262041	1.4587780
H	-0.5592783	-5.3161851	1.8748298
H	2.6818568	-1.6872174	0.7155483
C	2.4445584	-3.7702894	1.1339392
H	3.5150623	-4.0019795	1.0273395
O	2.1071853	-6.0532730	1.6040962
C	1.2690830	-7.1517412	1.9357229
H	1.9343077	-8.0357875	1.9998458
H	0.4972745	-7.3280676	1.1508343
H	0.7651485	-6.9990246	2.9185391

$vibrational spectrum (first 50 lines)

# mode	symmetry	wave number	IR intensity	selection rules	
#		cm**(-1)	km/mol	IR	RAMAN
1		0.00	0.00000	-	-
2		0.00	0.00000	-	-
3		0.00	0.00000	-	-
4		0.00	0.00000	-	-
5		0.00	0.00000	-	-
6		0.00	0.00000	-	-
7	a	13.88	0.32034	YES	YES
8	a	18.26	0.62803	YES	YES
9	a	27.75	0.07716	YES	YES
10	a	32.36	0.66263	YES	YES
11	a	45.33	1.39975	YES	YES
12	a	60.25	0.21397	YES	YES
13	a	73.39	0.26742	YES	YES
14	a	78.99	0.06984	YES	YES
15	a	84.11	1.41635	YES	YES
16	a	88.87	0.01213	YES	YES
17	a	95.89	0.21375	YES	YES
18	a	105.20	0.36663	YES	YES
19	a	111.80	1.07576	YES	YES
20	a	118.69	1.43423	YES	YES
21	a	124.83	0.14837	YES	YES
22	a	136.53	1.42939	YES	YES
23	a	143.86	0.40188	YES	YES
24	a	159.92	8.00226	YES	YES
25	a	165.94	0.73517	YES	YES
26	a	194.32	6.10158	YES	YES
27	a	205.45	1.54396	YES	YES
28	a	213.05	0.00844	YES	YES
29	a	233.20	0.11142	YES	YES
30	a	248.69	0.81890	YES	YES
31	a	265.09	0.05223	YES	YES
32	a	279.29	1.11173	YES	YES
33	a	289.86	5.32417	YES	YES
34	a	316.14	28.15569	YES	YES
35	a	345.11	5.27628	YES	YES

36	a	352.84	1.17456	YES	YES
37	a	375.31	0.18093	YES	YES
38	a	399.36	0.04523	YES	YES
39	a	409.33	5.63320	YES	YES
40	a	434.50	1.56057	YES	YES
41	a	438.43	0.83516	YES	YES
42	a	451.29	9.75395	YES	YES
43	a	455.47	10.47908	YES	YES
44	a	474.52	1.70527	YES	YES
45	a	476.07	17.48061	YES	YES
46	a	477.97	1.72571	YES	YES
47	a	486.30	5.16810	YES	YES
48	a	496.54	18.83211	YES	YES
49	a	505.72	0.93534	YES	YES
50	a	513.13	22.95202	YES	YES

2carene

SCF Energy (au) BP86/SV(P) -2656.555351040
SCF Energy (au) PBE0/def2-TZVPP -2656.101373911
SCF Energy (au) PBE0/def2-TZVPP -2656.1127001471 (Toluene Correction)
Zero Point Energy (au) 0.3470150
Chemical Potential (kJ mol^{-1}) 742.75
Dispersion Correction (au) PBE0/def2-TZVPP -0.08034937

xyz coordinates
50

Mn	0.6747038	1.8385993	0.1752711
C	1.5652323	1.8347783	1.7021249
C	2.0843047	1.1984744	-0.7314169
C	1.2688328	3.5089696	-0.2365566
O	1.7275226	4.5654215	-0.4354871
O	3.0209226	0.8901273	-1.3589775
O	2.1497085	1.8221227	2.7113471
C	-1.9305812	1.5927137	1.5125063
C	-1.4624044	3.8473572	1.0217437
C	-2.6836420	4.2818671	1.5440085
C	-3.5652548	3.3159158	2.0603782
C	-3.1899071	1.9707774	2.0444781
C	-1.3928037	0.2339606	1.4782335
C	-0.1035947	0.0700966	0.9343061
C	0.4765516	-1.2556243	1.0220112
C	-0.3048820	-2.3252311	1.5549398
O	-1.5799723	-2.1327247	1.9808483
C	-2.1968689	-0.8754676	2.0023028
H	-0.7424100	4.5728594	0.6136137
H	-2.9274443	5.3553578	1.5436039
H	-4.5415297	3.6112364	2.4789917
H	-3.8375804	1.1763427	2.4376719
N	-1.0874828	2.5465055	0.9902139
C	-0.5235791	2.3665435	-2.1986007
C	0.3683524	2.6023729	-3.2779887
C	0.9258162	1.5356937	-3.9760454
C	0.6033232	0.1866798	-3.6277054
C	-0.2858505	-0.0459585	-2.5605690
C	-0.8483946	1.0356816	-1.8407994
H	0.6117017	3.6369174	-3.5691239
H	1.6163731	1.7174839	-4.8142273
C	1.1659772	-0.9024251	-4.3665017
H	-0.5562005	-1.0795185	-2.2958788
H	-1.6506957	0.8331938	-1.1178447
C	1.8092407	-1.6020466	0.6452113
C	0.1763490	-3.6411218	1.6797075
O	-3.3359224	-0.8523568	2.4394769
C	1.4939146	-3.9296021	1.2854214
H	-0.5042156	-4.3948664	2.0975869
H	2.4690299	-0.8215293	0.2495501
C	2.3129253	-2.8893474	0.7697079
H	3.3458359	-3.1308199	0.4766936
O	2.0668627	-5.1550408	1.3651471
C	1.3035008	-6.2374159	1.8791937
H	0.9877438	-6.0511958	2.9320244
H	1.9687299	-7.1231865	1.8482152
H	0.4006382	-6.4333970	1.2549552
H	-1.0697160	3.2084192	-1.7479562
C	1.6490628	-1.8285386	-5.0074927

H   2.0777766   -2.6490545   -5.5683137

$vibrational spectrum (first 50 lines)

# mode	symmetry	wave number cm**(-1)	IR intensity km/mol	selection rules IR	RAMAN
1		0.00	0.00000	-	-
2		0.00	0.00000	-	-
3		0.00	0.00000	-	-
4		0.00	0.00000	-	-
5		0.00	0.00000	-	-
6		0.00	0.00000	-	-
7	a	15.99	0.70577	YES	YES
8	a	22.95	0.03306	YES	YES
9	a	29.08	0.04728	YES	YES
10	a	40.45	2.53288	YES	YES
11	a	52.84	0.18703	YES	YES
12	a	62.48	0.11912	YES	YES
13	a	67.76	0.12926	YES	YES
14	a	74.63	0.53585	YES	YES
15	a	82.92	0.09771	YES	YES
16	a	87.40	0.10081	YES	YES
17	a	88.82	0.36247	YES	YES
18	a	94.05	0.19654	YES	YES
19	a	104.38	0.49677	YES	YES
20	a	109.83	1.16404	YES	YES
21	a	116.37	0.06675	YES	YES
22	a	121.72	0.77967	YES	YES
23	a	131.74	1.12913	YES	YES
24	a	144.00	2.88231	YES	YES
25	a	155.39	1.11525	YES	YES
26	a	178.13	1.61582	YES	YES
27	a	187.91	1.40743	YES	YES
28	a	195.68	0.19098	YES	YES
29	a	212.54	0.06460	YES	YES
30	a	215.13	1.39804	YES	YES
31	a	241.92	0.39351	YES	YES
32	a	264.65	0.08453	YES	YES
33	a	277.54	0.29405	YES	YES
34	a	294.48	7.94048	YES	YES
35	a	343.55	5.47533	YES	YES
36	a	351.83	1.57566	YES	YES
37	a	365.25	2.16057	YES	YES
38	a	376.27	0.29818	YES	YES
39	a	406.40	0.04267	YES	YES
40	a	424.53	2.04832	YES	YES
41	a	438.25	1.01116	YES	YES
42	a	448.97	2.99835	YES	YES
43	a	456.16	1.00128	YES	YES
44	a	458.12	0.23641	YES	YES
45	a	469.81	2.20085	YES	YES
46	a	477.76	1.67426	YES	YES
47	a	482.30	0.73152	YES	YES
48	a	504.43	3.88294	YES	YES
49	a	512.46	12.72546	YES	YES
50	a	515.89	2.33368	YES	YES

$TS_{2cPhC≡CH}$

SCF Energy (au) BP86/SV(P) -2656.572844027
SCF Energy (au) PBE0/def2-TZVPP -2656.111027299
SCF Energy (au) PBE0/def2-TZVPP -2656.1207091834 (Toluene Correction)
Zero Point Energy (au) 0.3473902
Chemical Potential (kJ mol^{-1}) 749.75
Dispersion Correction (au) PBE0/def2-TZVPP -0.07466039

xyz coordinates
50

Mn	0.7154779	1.2718944	0.8585579
C	0.6509166	1.0663208	2.6561362
C	2.4943899	1.0659812	0.8457605
C	1.0477907	3.0474458	0.9697937
O	1.2930982	4.1834156	1.0701627
O	3.6632254	1.0346249	0.8439793
O	0.6035825	0.9210347	3.8084852
C	-2.1526108	0.4936037	0.8376907
C	-1.9440271	2.8295670	0.6830202
C	-3.3275382	3.0108585	0.6189450
C	-4.1516453	1.8718905	0.6599127
C	-3.5655882	0.6099846	0.7646357
C	-1.4098119	-0.7551583	0.9475489
C	-0.0026070	-0.6887212	0.8286954
C	0.7388697	-1.9370362	0.9780637
C	0.0207313	-3.1344053	1.2485164
O	-1.3351428	-3.1535773	1.3482708
C	-2.1279553	-2.0132527	1.1798435
N	-1.3626427	1.6141058	0.7941076
C	0.2967599	2.4174150	-2.2858105
C	1.0852001	3.5964778	-2.3360922
C	0.9370602	4.5086942	-3.3900786
C	-0.0103784	4.2798806	-4.4042806
C	-0.8070630	3.1211541	-4.3610729
C	-0.6571112	2.1975209	-3.3179860
C	0.4433075	1.4251589	-1.2466822
C	0.5144164	0.1726742	-0.9443548
H	-1.2679226	3.6966624	0.6460343
H	-3.7411778	4.0279202	0.5382913
H	-5.2486555	1.9686332	0.6082838
H	-4.1608034	-0.3109533	0.8031514
H	1.8286258	3.7878723	-1.5481717
H	1.5672406	5.4129848	-3.4168267
H	-0.1298512	5.0049197	-5.2260762
H	-1.5542673	2.9332625	-5.1500163
H	-1.2818548	1.2908414	-3.2824476
H	0.7989254	-0.7890980	-1.3824576
O	-3.3319607	-2.1878626	1.2605275
C	0.6517991	-4.3800793	1.4261394
C	2.1502703	-2.0768869	0.8523120
H	2.7574896	-1.1928172	0.6232103
C	2.7964866	-3.2969295	1.0078178
H	3.8893431	-3.3816065	0.9091080
C	2.0488474	-4.4667252	1.3074966
H	0.0130419	-5.2466554	1.6427281
O	2.7626551	-5.6098718	1.4534306
C	2.0725350	-6.8141547	1.7576017
H	2.8493358	-7.6006475	1.8362663
H	1.3527940	-7.0878441	0.9511241

H 1.5263991 -6.7385165 2.7267044

$vibrational spectrum (first 50 lines)

# mode	symmetry	wave number cm**(-1)	IR intensity km/mol	selection rules IR	selection rules RAMAN
1	a	-187.84	0.00000	YES	YES
2		0.00	0.00000	-	-
3		0.00	0.00000	-	-
4		0.00	0.00000	-	-
5		0.00	0.00000	-	-
6		0.00	0.00000	-	-
7		0.00	0.00000	-	-
8	a	18.87	0.34062	YES	YES
9	a	21.25	0.13814	YES	YES
10	a	29.72	1.03703	YES	YES
11	a	36.00	0.79671	YES	YES
12	a	49.94	0.98335	YES	YES
13	a	71.66	0.02113	YES	YES
14	a	75.08	0.27736	YES	YES
15	a	85.06	0.08022	YES	YES
16	a	90.01	0.11418	YES	YES
17	a	94.56	0.79315	YES	YES
18	a	99.40	0.23814	YES	YES
19	a	107.78	1.28719	YES	YES
20	a	120.76	1.06321	YES	YES
21	a	123.89	0.34838	YES	YES
22	a	132.71	0.09873	YES	YES
23	a	141.13	1.81038	YES	YES
24	a	175.33	0.48357	YES	YES
25	a	185.52	0.52559	YES	YES
26	a	191.89	1.93423	YES	YES
27	a	201.19	1.53381	YES	YES
28	a	214.32	0.04745	YES	YES
29	a	226.33	0.06137	YES	YES
30	a	243.09	0.04802	YES	YES
31	a	265.90	0.46640	YES	YES
32	a	271.66	0.48355	YES	YES
33	a	288.62	7.04926	YES	YES
34	a	344.01	4.62284	YES	YES
35	a	350.57	4.43378	YES	YES
36	a	357.95	1.76400	YES	YES
37	a	378.68	0.49936	YES	YES
38	a	396.67	1.50759	YES	YES
39	a	402.19	0.03833	YES	YES
40	a	432.76	0.68419	YES	YES
41	a	438.31	1.15349	YES	YES
42	a	445.78	1.61172	YES	YES
43	a	455.17	12.15254	YES	YES
44	a	470.57	2.27048	YES	YES
45	a	474.77	3.30834	YES	YES
46	a	477.93	0.76845	YES	YES
47	a	486.56	9.06500	YES	YES
48	a	497.35	7.00575	YES	YES
49	a	507.38	7.85608	YES	YES
50	a	512.33	23.14243	YES	YES

2c-7-a

SCF Energy (au) BP86/SV(P) -2656.607907290
SCF Energy (au) PBE0/def2-TZVPP -2656.153135620
SCF Energy (au) PBE0/def2-TZVPP -2656.1645801340 (Toluene Correction)
Zero Point Energy (au) 0.3493258
Chemical Potential (kJ mol^{-1}) 752.88
Dispersion Correction (au) PBE0/def2-TZVPP -0.07461645

xyz coordinates
50

Mn	0.5568591	1.6985024	0.7449664
C	0.8758006	1.9861767	2.5282479
C	2.2078072	1.0288669	0.4699194
C	1.0526202	3.3443855	0.3448072
O	1.3365016	4.4542972	0.1068805
O	3.2763629	0.6056276	0.2718350
O	1.1290498	2.1917017	3.6460551
C	-1.9525464	0.9135951	1.2008560
C	-2.2087949	3.1806015	0.6545160
C	-3.5980339	3.1001936	0.8220117
C	-4.1673662	1.8747623	1.2072006
C	-3.3327150	0.7619569	1.4020454
C	-0.9332938	-0.1724436	1.3702024
C	-0.2970291	-0.7992692	0.2679151
C	0.4112892	-2.0445118	0.5070275
C	0.4494609	-2.5782122	1.8214508
O	-0.1634867	-1.9517265	2.8657549
C	-0.9237244	-0.7845283	2.7125607
H	-1.7034714	4.1156269	0.3639822
H	-4.2199830	3.9938631	0.6578844
H	-5.2555278	1.7857413	1.3571158
H	-3.7365026	-0.2122097	1.7147241
N	-1.4192473	2.1075062	0.8540589
C	0.1273676	1.8126810	-2.4486191
C	1.2341260	2.6188559	-2.8166996
C	1.2761824	3.2656171	-4.0606346
C	0.2027201	3.1461026	-4.9615437
C	-0.9107624	2.3625977	-4.6094765
C	-0.9447463	1.6982062	-3.3742644
H	2.0823162	2.7177983	-2.1215768
H	2.1554299	3.8754995	-4.3273601
H	0.2315797	3.6668903	-5.9330948
H	-1.7626879	2.2682927	-5.3038944
H	-1.8244065	1.0931309	-3.0985686
C	0.0752056	1.1030222	-1.1609834
C	-0.3320264	-0.2005457	-1.0745874
H	-0.5423004	-0.8487183	-1.9498833
O	-1.5334671	-0.3860322	3.6829937
C	1.1222703	-3.7736200	2.1254391
C	1.1113793	-2.7590508	-0.5054851
C	1.7849947	-4.4662007	1.0944442
H	1.1079008	-4.1211548	3.1668329
C	1.7752518	-3.9466582	-0.2298861
O	2.4637288	-5.6237115	1.2683922
H	2.3176879	-4.4987806	-1.0120455
H	1.1374423	-2.3461280	-1.5251060
C	2.5408172	-6.1906012	2.5705790
H	1.5303268	-6.4530720	2.9614159
H	3.1436490	-7.1142847	2.4659778

```
H     3.0459919   -5.5006391    3.2856165


$vibrational spectrum (first 50 lines)
#  mode     symmetry     wave number   IR intensity    selection rules
#                         cm**(-1)        km/mol          IR     RAMAN
     1                      0.00         0.00000         -        -
     2                      0.00         0.00000         -        -
     3                      0.00         0.00000         -        -
     4                      0.00         0.00000         -        -
     5                      0.00         0.00000         -        -
     6                      0.00         0.00000         -        -
     7         a           20.10         1.38279        YES      YES
     8         a           27.07         0.15528        YES      YES
     9         a           35.65         0.22206        YES      YES
    10         a           44.68         0.07873        YES      YES
    11         a           48.47         0.36976        YES      YES
    12         a           60.26         1.22560        YES      YES
    13         a           64.46         0.79056        YES      YES
    14         a           70.48         0.77911        YES      YES
    15         a           75.09         0.86971        YES      YES
    16         a           83.62         0.20181        YES      YES
    17         a           92.93         0.27690        YES      YES
    18         a           95.50         0.12153        YES      YES
    19         a          100.53         0.97210        YES      YES
    20         a          107.24         0.83357        YES      YES
    21         a          130.34         2.19441        YES      YES
    22         a          144.71         1.02406        YES      YES
    23         a          152.27         0.06224        YES      YES
    24         a          160.22         0.42503        YES      YES
    25         a          183.62         0.63352        YES      YES
    26         a          188.35         3.29820        YES      YES
    27         a          207.23         0.41514        YES      YES
    28         a          220.11         1.25560        YES      YES
    29         a          243.46         2.26744        YES      YES
    30         a          254.64         0.28180        YES      YES
    31         a          269.93         0.38755        YES      YES
    32         a          284.00         7.05249        YES      YES
    33         a          296.15         0.31720        YES      YES
    34         a          319.97         2.14924        YES      YES
    35         a          354.29         3.72748        YES      YES
    36         a          377.61         3.01901        YES      YES
    37         a          406.38         1.16579        YES      YES
    38         a          420.47         2.99717        YES      YES
    39         a          424.43         1.09786        YES      YES
    40         a          435.13         2.18478        YES      YES
    41         a          452.35         3.61046        YES      YES
    42         a          457.27         0.59089        YES      YES
    43         a          459.72         3.29054        YES      YES
    44         a          471.63         5.10425        YES      YES
    45         a          483.33         1.76675        YES      YES
    46         a          492.29         0.31516        YES      YES
    47         a          495.34         3.08382        YES      YES
    48         a          508.56        24.25269        YES      YES
    49         a          511.88        15.26427        YES      YES
    50         a          525.03         6.13792        YES      YES
```

TS2c-7-a

SCF Energy (au) BP86/SV(P) -2656.589681302
SCF Energy (au) PBE0/def2-TZVPP -2656.127734051
SCF Energy (au) PBE0/def2-TZVPP -2656.1384501169 (Toluene Correction)
Zero Point Energy (au) 0.3486472
Chemical Potential (kJ mol^{-1}) 756.21
Dispersion Correction (au) PBE0/def2-TZVPP -0.07459590

xyz coordinates
50

C	1.2905287	0.9535112	2.5616201
C	-0.9197071	1.9646055	2.9271287
C	-0.4122741	1.6921269	0.0922010
C	-0.7191385	-0.7114350	2.8596177
C	0.2808264	-0.7397716	-0.0014070
C	1.4430731	-1.6440924	0.0193646
O	0.0650652	-3.4714966	-0.8196560
C	-1.0947594	-2.6945831	-0.8894214
C	-1.0038764	-1.3290190	-0.3691034
C	-2.1526603	-0.5108069	-0.2695098
C	-3.4269389	-0.7584972	-0.8758850
H	-3.5156183	-1.6499252	-1.5102759
C	-4.4925416	0.0871642	-0.6328729
H	-5.4723434	-0.1100011	-1.0971309
C	-4.3133652	1.2243363	0.2103065
H	-5.1468514	1.9032497	0.4469242
C	-3.0656403	1.4763681	0.7330381
H	-2.8640061	2.3597780	1.3555406
Mn	-0.3090436	0.7213320	1.8095903
N	-1.9836035	0.6457690	0.5132453
O	2.3451360	1.1188886	3.0393080
O	-1.2838547	2.7836959	3.6811210
C	0.5044019	0.6762796	-0.2021139
O	-1.0236187	-1.6185322	3.5215185
C	1.2699759	-2.9871371	-0.3844024
H	1.5157228	0.9911719	-0.5146495
C	-0.5908668	2.9837538	-0.5534044
C	-0.9336001	5.4719500	-1.8565274
C	-1.0344547	5.3901909	-0.4554332
C	-0.8706989	4.1580880	0.1921495
C	-0.4950841	3.0825020	-1.9682475
C	-0.6628515	4.3151183	-2.6115347
H	-1.0661298	6.4422245	-2.3634287
H	-1.2382709	6.2969495	0.1372254
H	-0.9337993	4.0962851	1.2897309
H	-0.2902434	2.1714512	-2.5544636
H	-0.5828506	4.3774239	-3.7093201
C	2.7521751	-1.2589951	0.4082312
C	2.3299364	-3.9136233	-0.3944629
O	-2.0787877	-3.2446713	-1.3561039
C	3.6132604	-3.4987326	0.0024058
H	2.1078043	-4.9369764	-0.7248281
H	2.9335833	-0.2302077	0.7548869
C	3.8187840	-2.1536623	0.4025414
H	4.8261932	-1.8452618	0.7200742
O	4.7053255	-4.3058779	0.0361834
C	4.5573855	-5.6692308	-0.3301264
H	5.5598442	-6.1282601	-0.2164421
H	4.2237162	-5.7766278	-1.3891005

H 3.8347410 -6.1967897 0.3358980

$vibrational spectrum (first 50 lines)

# mode	symmetry	wave number cm**(-1)	IR intensity km/mol	selection rules IR	RAMAN
1	a	-311.38	0.00000	YES	YES
2		0.00	0.00000	-	-
3		0.00	0.00000	-	-
4		0.00	0.00000	-	-
5		0.00	0.00000	-	-
6		0.00	0.00000	-	-
7		0.00	0.00000	-	-
8	a	26.69	1.15702	YES	YES
9	a	36.19	0.28582	YES	YES
10	a	39.19	0.08389	YES	YES
11	a	44.88	0.33612	YES	YES
12	a	51.87	1.20632	YES	YES
13	a	58.11	0.16865	YES	YES
14	a	70.99	0.44455	YES	YES
15	a	77.15	0.35889	YES	YES
16	a	86.07	0.00832	YES	YES
17	a	93.49	0.88785	YES	YES
18	a	95.91	0.59730	YES	YES
19	a	108.81	0.18521	YES	YES
20	a	114.12	2.24890	YES	YES
21	a	124.01	0.45238	YES	YES
22	a	132.60	2.26653	YES	YES
23	a	147.98	1.19223	YES	YES
24	a	154.50	1.05969	YES	YES
25	a	185.04	0.97549	YES	YES
26	a	203.18	1.07057	YES	YES
27	a	214.14	0.49738	YES	YES
28	a	229.64	1.27678	YES	YES
29	a	231.82	0.16224	YES	YES
30	a	260.67	4.11465	YES	YES
31	a	269.98	1.21811	YES	YES
32	a	284.08	5.05891	YES	YES
33	a	291.39	6.50273	YES	YES
34	a	337.62	2.02035	YES	YES
35	a	361.05	2.32153	YES	YES
36	a	384.22	5.16560	YES	YES
37	a	395.73	4.88767	YES	YES
38	a	407.52	0.97007	YES	YES
39	a	417.73	3.01938	YES	YES
40	a	425.89	3.59428	YES	YES
41	a	437.57	0.48217	YES	YES
42	a	460.42	1.71742	YES	YES
43	a	465.86	3.53113	YES	YES
44	a	475.83	1.97454	YES	YES
45	a	480.14	1.40535	YES	YES
46	a	483.36	0.49255	YES	YES
47	a	501.80	6.50303	YES	YES
48	a	512.40	17.74772	YES	YES
49	a	517.82	8.99431	YES	YES
50	a	529.84	12.50918	YES	YES

2c-iso

SCF Energy (au) BP86/SV(P) -2656.604365285
SCF Energy (au) PBE0/def2-TZVPP -2656.145976270
SCF Energy (au) PBE0/def2-TZVPP -2656.1564350334 (Toluene Correction)
Zero Point Energy (au) 0.3501179
Chemical Potential (kJ mol^{-1}) 759.37
Dispersion Correction (au) PBE0/def2-TZVPP -0.07544400

xyz coordinates
50

C	1.8352499	-0.0078585	2.3105139
C	0.9355727	2.2288279	2.6374111
C	0.4408044	1.6060097	-0.1919301
C	-0.7246458	0.0501743	2.9262900
C	-0.2488772	-0.7483215	0.0378792
C	0.0817073	-2.1824707	0.0701206
O	-2.2277590	-2.7506888	-0.5002873
C	-2.6461910	-1.4289604	-0.6009059
C	-1.6552244	-0.3989222	-0.2636973
C	-2.0099113	0.9428767	-0.1792390
C	-3.2944181	1.5342289	-0.4076285
H	-4.0606953	0.8767678	-0.8393329
C	-3.5593472	2.8332716	-0.0314126
H	-4.5639870	3.2574755	-0.1876011
C	-2.5297107	3.6369286	0.5717362
H	-2.7364886	4.6590414	0.9228321
C	-1.2640490	3.1412855	0.6820972
H	-0.4121915	3.7274168	1.0509110
Mn	0.3851275	0.8085083	1.6956558
N	-0.9425429	1.8206402	0.2837025
O	2.7981279	-0.5447690	2.7033317
O	1.3164825	3.1312259	3.2762070
C	0.7475410	0.2264693	-0.3061637
O	-1.4462229	-0.4080504	3.7153421
C	-0.9370106	-3.1229531	-0.1996943
H	1.7401393	-0.0658062	-0.6815356
C	1.2010741	2.7007008	-0.8501249
C	2.6869398	4.7100591	-2.1776520
C	3.3115776	3.8742038	-1.2339749
C	2.5738936	2.8844960	-0.5682050
C	0.5824465	3.5397589	-1.8062658
C	1.3206824	4.5409959	-2.4580996
H	3.2651395	5.4955734	-2.6912518
H	4.3811468	4.0050537	-1.0009852
H	3.0547630	2.2537820	0.1972900
H	-0.4820438	3.3971038	-2.0544396
H	0.8225838	5.1877053	-3.1990933
C	1.3775347	-2.7045113	0.3165273
C	-0.7021620	-4.5097046	-0.2100000
O	-3.8053734	-1.2516026	-0.9359657
C	0.5936231	-4.9932189	0.0466360
H	-1.5524750	-5.1679078	-0.4325225
H	2.2040941	-2.0157930	0.5470916
C	1.6391314	-4.0729533	0.3067340
H	2.6472861	-4.4632203	0.5116445
O	0.9330225	-6.3093488	0.0643662
C	-0.0781671	-7.2763543	-0.1714814
H	0.4182788	-8.2646390	-0.0972520
H	-0.5239277	-7.1664762	-1.1883494

H   -0.8905492   -7.2160504   0.5907716

$vibrational spectrum (first 50 lines)

# mode	symmetry	wave number cm**(-1)	IR intensity km/mol	selection rules IR	RAMAN
1		0.00	0.00000	-	-
2		0.00	0.00000	-	-
3		0.00	0.00000	-	-
4		0.00	0.00000	-	-
5		0.00	0.00000	-	-
6		0.00	0.00000	-	-
7	a	27.06	1.17038	YES	YES
8	a	37.28	0.12865	YES	YES
9	a	39.61	0.03152	YES	YES
10	a	41.68	0.51189	YES	YES
11	a	46.59	0.38969	YES	YES
12	a	59.80	0.97041	YES	YES
13	a	68.34	0.23894	YES	YES
14	a	77.15	1.35316	YES	YES
15	a	83.93	0.06991	YES	YES
16	a	92.89	1.67474	YES	YES
17	a	95.06	0.31309	YES	YES
18	a	109.82	0.32314	YES	YES
19	a	110.61	2.12247	YES	YES
20	a	124.91	1.80525	YES	YES
21	a	136.10	0.73632	YES	YES
22	a	146.10	4.01492	YES	YES
23	a	152.94	3.97656	YES	YES
24	a	188.45	0.90987	YES	YES
25	a	196.20	0.78423	YES	YES
26	a	212.19	0.30553	YES	YES
27	a	224.11	0.51245	YES	YES
28	a	244.09	0.15789	YES	YES
29	a	249.09	0.36990	YES	YES
30	a	268.15	2.91556	YES	YES
31	a	279.52	10.65655	YES	YES
32	a	285.58	2.78974	YES	YES
33	a	344.29	1.52592	YES	YES
34	a	367.35	0.99108	YES	YES
35	a	387.39	3.87057	YES	YES
36	a	402.59	1.56725	YES	YES
37	a	404.45	3.35257	YES	YES
38	a	421.70	6.30994	YES	YES
39	a	426.30	1.35630	YES	YES
40	a	443.69	1.35499	YES	YES
41	a	455.50	1.35935	YES	YES
42	a	458.73	4.63318	YES	YES
43	a	462.70	1.79395	YES	YES
44	a	472.23	3.74048	YES	YES
45	a	483.75	17.48431	YES	YES
46	a	484.50	6.33786	YES	YES
47	a	497.16	16.43181	YES	YES
48	a	503.19	2.99507	YES	YES
49	a	515.72	5.46606	YES	YES
50	a	523.54	24.40343	YES	YES

3c

SCF Energy (au) BP86/SV(P) -2656.636753068
SCF Energy (au) PBE0/def2-TZVPP -2656.182859910
SCF Energy (au) PBE0/def2-TZVPP -2656.1950967934 (Toluene Correction)
Zero Point Energy (au) 0.3514231
Chemical Potential (kJ mol^{-1}) 764.89
Dispersion Correction (au) PBE0/def2-TZVPP -0.07528434

xyz coordinates
50

C	1.0687119	2.0069427	1.9665847
C	-1.2384117	0.9513710	2.7497399
C	-0.8527293	1.4808069	-0.1498394
C	1.0987521	-0.3905050	2.6579553
C	0.3837910	-0.6142086	-0.1514984
C	1.4747795	-1.5671939	-0.3590826
O	0.3147847	-3.1419622	1.0968061
C	-0.8815130	-2.4224473	1.1166727
C	-0.8677119	-1.0919841	0.4768707
C	-2.1321475	-0.5779118	-0.0577463
C	-3.3322453	-1.2999187	-0.2205829
H	-3.3536073	-2.3393139	0.1303148
C	-4.4633098	-0.6850021	-0.7683071
H	-5.4034837	-1.2514151	-0.8649074
C	-4.3870236	0.6529161	-1.1936850
H	-5.2478570	1.1751017	-1.6370243
C	-3.1848738	1.3373216	-1.0322851
H	-3.0483606	2.3861746	-1.3292693
Mn	0.0593317	0.5916599	1.5584773
N	-2.1063242	0.7458552	-0.4495133
O	1.7318709	2.9330923	2.2294967
O	-2.1045189	1.2037223	3.4923417
C	0.3667136	0.7548972	-0.5032525
O	1.7803020	-1.0128731	3.3730781
C	1.3915213	-2.8095532	0.3068887
H	1.2349818	1.2606109	-0.9510912
C	-0.9281689	2.9598387	-0.3910823
C	-1.0661764	5.7571731	-0.8491858
C	-0.4299455	4.9208193	-1.7806751
C	-0.3691591	3.5345370	-1.5563256
C	-1.5643126	3.8167545	0.5382249
C	-1.6375446	5.1989257	0.3088166
H	-1.1123071	6.8452307	-1.0212866
H	0.0220844	5.3477568	-2.6913725
H	0.1194990	2.8857267	-2.3023345
H	-1.9914626	3.3875132	1.4588956
H	-2.1289506	5.8493621	1.0510925
C	2.6226905	-1.3279633	-1.1522849
O	-1.8314373	-2.9544721	1.6578152
C	2.4077981	-3.7758538	0.2171020
H	2.7125080	-0.3754135	-1.6981224
C	3.6429685	-2.2711661	-1.2603751
H	4.5359683	-2.0832697	-1.8754506
C	3.5453734	-3.5043515	-0.5669520
H	2.2750220	-4.7133923	0.7728747
O	4.5895803	-4.3604712	-0.7226024
C	4.5618536	-5.6026148	-0.0359984
H	3.6992266	-6.2321562	-0.3588260
H	5.5069350	-6.1192192	-0.2981709

```
H     4.5165344   -5.4594776    1.0690817


$vibrational spectrum (first 50 lines)
#  mode     symmetry     wave number   IR intensity    selection rules
#                         cm**(-1)        km/mol         IR     RAMAN
     1                       0.00         0.00000        -        -
     2                       0.00         0.00000        -        -
     3                       0.00         0.00000        -        -
     4                       0.00         0.00000        -        -
     5                       0.00         0.00000        -        -
     6                       0.00         0.00000        -        -
     7         a            29.65         2.00273       YES      YES
     8         a            33.47         0.41735       YES      YES
     9         a            40.46         0.01014       YES      YES
    10         a            48.74         0.43432       YES      YES
    11         a            58.42         0.01446       YES      YES
    12         a            62.34         0.36332       YES      YES
    13         a            66.07         0.30527       YES      YES
    14         a            82.83         1.16751       YES      YES
    15         a            85.99         0.51443       YES      YES
    16         a            91.75         0.26804       YES      YES
    17         a            95.34         0.76366       YES      YES
    18         a           104.04         0.40281       YES      YES
    19         a           107.69         0.12115       YES      YES
    20         a           125.08         5.27675       YES      YES
    21         a           137.47         0.70154       YES      YES
    22         a           156.33         0.34074       YES      YES
    23         a           167.18         4.21591       YES      YES
    24         a           200.38         0.28690       YES      YES
    25         a           207.66         0.89867       YES      YES
    26         a           217.62         0.83086       YES      YES
    27         a           222.67         0.86049       YES      YES
    28         a           238.79         5.00728       YES      YES
    29         a           267.57         0.91353       YES      YES
    30         a           271.94         0.41201       YES      YES
    31         a           280.31         2.18658       YES      YES
    32         a           322.97         7.03945       YES      YES
    33         a           347.11         0.62276       YES      YES
    34         a           377.82         5.97088       YES      YES
    35         a           403.32         4.81655       YES      YES
    36         a           404.95         0.03837       YES      YES
    37         a           419.30         4.11660       YES      YES
    38         a           430.41         2.72495       YES      YES
    39         a           438.52         0.83465       YES      YES
    40         a           441.59         0.86807       YES      YES
    41         a           457.91         0.78437       YES      YES
    42         a           467.72         5.83182       YES      YES
    43         a           476.59         6.33916       YES      YES
    44         a           482.34         1.64979       YES      YES
    45         a           487.38         4.97373       YES      YES
    46         a           493.27         2.76695       YES      YES
    47         a           509.68         0.57012       YES      YES
    48         a           520.36         3.95999       YES      YES
    49         a           524.96         8.63861       YES      YES
    50         a           532.22        26.46998       YES      YES
```

4c
SCF Energy (au) BP86/SV(P) -2924.819241356
SCF Energy (au) PBE0/def2-TZVPP -2924.370284789
SCF Energy (au) PBE0/def2-TZVPP -2924.3824927155 (Toluene Correction)
Zero Point Energy (au) 0.4390866
Chemical Potential (kJ mol^{-1}) 962.72
Dispersion Correction (au) PBE0/def2-TZVPP -0.09318764

xyz coordinates
61

Mn	1.6721258	0.3233780	0.7682253
C	0.6290315	0.9245852	2.0707775
C	2.0550050	-1.0336076	1.8570844
C	3.0601257	1.3275961	1.4185111
O	3.9316002	1.9570623	1.8701459
O	2.2957536	-1.8998182	2.6093924
O	0.0109594	1.2614496	3.0046807
C	-0.0291774	2.4815272	-0.7380298
C	2.1682354	2.2173775	-1.4901833
C	2.0246879	3.1929678	-2.4777195
C	0.7868185	3.8415986	-2.6019047
C	-0.2326342	3.4837304	-1.7194735
C	-1.1760001	2.1748866	0.1586722
C	-1.7460900	0.9093781	0.3068782
C	-2.9862520	0.7878006	1.0629702
C	-3.5480493	1.9492691	1.6487208
O	-2.9546972	3.1656584	1.5150018
C	-1.7757103	3.3637387	0.7849561
H	3.1179590	1.6797958	-1.3684138
H	2.8789795	3.4387703	-3.1279037
H	0.6196821	4.6170883	-3.3670344
H	-1.2131348	3.9756240	-1.7732790
N	1.1773366	1.8499077	-0.6359658
C	0.4481001	-2.0199008	-0.7972068
C	0.5927469	-3.2065433	-0.0274475
C	0.8527832	-4.4454938	-0.6441088
C	0.9972855	-4.5289499	-2.0394714
C	0.8658417	-3.3621248	-2.8140191
C	0.5914783	-2.1266530	-2.2043386
H	0.4586110	-3.1583561	1.0649587
H	0.9381617	-5.3529626	-0.0231250
H	1.2087599	-5.4976151	-2.5210810
H	0.9707581	-3.4159213	-3.9110675
H	0.4811210	-1.2181651	-2.8185804
C	0.1411849	-0.7115088	-0.1620570
C	-1.1591254	-0.3018709	-0.2740757
H	-1.8901567	-0.9811490	-0.7603031
O	-1.3578629	4.5056159	0.7249099
C	-4.7318733	1.9174478	2.4081179
C	-3.6661310	-0.4405583	1.2972634
C	-5.3852382	0.6880988	2.6028337
H	-5.0956790	2.8635461	2.8300677
C	-4.8387209	-0.4973012	2.0405922
O	-6.5289648	0.5332372	3.3162921
H	-5.3592731	-1.4500174	2.2208355
H	-3.2431370	-1.3716262	0.8921326
C	-7.1130398	1.6742823	3.9280738
H	-7.4096119	2.4381182	3.1716383
H	-8.0184415	1.3104871	4.4537757

```
H    -6.4213348    2.1409628    4.6679711
H     2.6540429   -2.6688393   -0.1641272
O     3.5867363   -2.5861256   -0.5291045
C     3.8159642   -1.3463668   -0.9429591
C     5.1312687   -1.1646193   -1.6763212
O     3.0556851   -0.3892640   -0.7400582
H     5.7775215   -0.5613385   -0.9954134
H     4.9186880   -0.4957832   -2.5398119
C     5.8491648   -2.4438361   -2.1156549
H     6.7948463   -2.1878161   -2.6400836
H     5.2216458   -3.0435361   -2.8085806
H     6.0996395   -3.0873179   -1.2465751

$vibrational spectrum (first 50 lines)
#  mode     symmetry     wave number   IR intensity    selection rules
#                         cm**(-1)        km/mol          IR     RAMAN
     1                       0.00         0.00000         -       -
     2                       0.00         0.00000         -       -
     3                       0.00         0.00000         -       -
     4                       0.00         0.00000         -       -
     5                       0.00         0.00000         -       -
     6                       0.00         0.00000         -       -
     7         a             3.99         0.00484        YES     YES
     8         a            21.62         1.20399        YES     YES
     9         a            32.56         0.27553        YES     YES
    10         a            35.70         0.13865        YES     YES
    11         a            38.73         0.21836        YES     YES
    12         a            43.75         0.23276        YES     YES
    13         a            51.54         0.20304        YES     YES
    14         a            59.03         1.72770        YES     YES
    15         a            66.16         0.80785        YES     YES
    16         a            69.25         1.01259        YES     YES
    17         a            80.15         0.45114        YES     YES
    18         a            81.60         0.48836        YES     YES
    19         a            87.61         0.13775        YES     YES
    20         a            94.41         0.27952        YES     YES
    21         a           100.61         0.28333        YES     YES
    22         a           101.53         1.26953        YES     YES
    23         a           110.36         0.26084        YES     YES
    24         a           120.20         0.42880        YES     YES
    25         a           132.84         0.96065        YES     YES
    26         a           139.30         2.40215        YES     YES
    27         a           148.19         0.30040        YES     YES
    28         a           154.03         0.95487        YES     YES
    29         a           163.07         0.40821        YES     YES
    30         a           171.82         0.47126        YES     YES
    31         a           182.98         1.73953        YES     YES
    32         a           192.92         0.45780        YES     YES
    33         a           211.45         3.97156        YES     YES
    34         a           216.91         0.29229        YES     YES
    35         a           228.34         1.75785        YES     YES
    36         a           232.69         1.56210        YES     YES
    37         a           249.52         1.09811        YES     YES
    38         a           253.86         0.11549        YES     YES
    39         a           266.44         0.91612        YES     YES
    40         a           276.92         2.11323        YES     YES
    41         a           280.77         1.35669        YES     YES
    42         a           303.11         1.47944        YES     YES
    43         a           327.27         4.01099        YES     YES
    44         a           348.33         3.35486        YES     YES
    45         a           395.09         0.42935        YES     YES
```

46	a	400.62	0.42437	YES	YES
47	a	431.83	4.83818	YES	YES
48	a	435.68	3.94660	YES	YES
49	a	444.57	0.44714	YES	YES
50	a	458.95	2.55383	YES	YES

TS4c-7-a
SCF Energy (au) BP86/SV(P) -2924.806043925
SCF Energy (au) PBE0/def2-TZVPP -2924.350291251
SCF Energy (au) PBE0/def2-TZVPP -2924.3626736269 (Toluene Correction)
Zero Point Energy (au) 0.4351288
Chemical Potential (kJ mol^{-1}) 959.77
Dispersion Correction (au) PBE0/def2-TZVPP -0.09161055

xyz coordinates
61

Mn	2.2285946	0.0665854	0.5929059
C	1.2657915	0.6747397	1.9682850
C	2.7682454	-1.3407231	1.5745320
C	3.6432744	0.9982468	1.2073121
O	4.5486049	1.5984109	1.6311564
O	3.1636605	-2.2204557	2.2257115
O	0.6860490	1.0366207	2.9150130
C	0.4475612	2.0665568	-1.0263083
C	2.7165501	2.0129415	-1.5776392
C	2.5326953	2.7987221	-2.7166684
C	1.2273495	3.1749897	-3.0631625
C	0.1920784	2.8188581	-2.1973753
C	-0.6771193	1.9508261	-0.0507188
C	-1.2925964	0.7883072	0.4190891
C	-2.4732837	0.9308504	1.2809095
C	-2.9221275	2.2326571	1.6205125
O	-2.2796808	3.3432600	1.1721071
C	-1.1608918	3.2886278	0.3407345
H	3.7214273	1.6614757	-1.3058929
H	3.4048715	3.0934145	-3.3208081
H	1.0218228	3.7631728	-3.9721617
H	-0.8390706	3.1403184	-2.4016771
N	1.7066131	1.6095258	-0.7623745
C	0.4527910	-2.5991409	0.0658712
C	0.1415733	-3.2079992	1.3083263
C	0.1307781	-4.6033621	1.4575436
C	0.4156173	-5.4345910	0.3610900
C	0.7116743	-4.8504182	-0.8820514
C	0.7394596	-3.4534555	-1.0282276
H	-0.0718737	-2.5650774	2.1787420
H	-0.1008893	-5.0436921	2.4417780
H	0.4049064	-6.5313323	0.4739405
H	0.9155793	-5.4895048	-1.7571633
H	0.9421392	-3.0168804	-2.0166851
C	0.4229450	-1.0974415	-0.0280511
C	-0.8437230	-0.5802475	0.1625210
H	-1.6522669	-1.3272096	0.2612593
O	-0.6803908	4.3562590	0.0052007
C	-4.0405281	2.4705930	2.4396392
C	-3.2144214	-0.1485881	1.8467454
C	-4.7518492	1.3779664	2.9626292
H	-4.3080810	3.5157218	2.6437496
C	-4.3226697	0.0587654	2.6582585
O	-5.8412918	1.4808928	3.7612061
H	-4.8824633	-0.7868280	3.0855131
H	-2.9061358	-1.1856541	1.6550659
C	-6.3123780	2.7747513	4.1136977
H	-6.6193888	3.3569830	3.2136411
H	-7.1957595	2.6125965	4.7627270

```
H    -5.5418390    3.3496288    4.6783690
H     1.1110293   -0.7254695   -1.2202484
O     1.6160976   -0.8662916   -2.3292343
C     2.8792047   -0.9856471   -2.0858409
C     3.7677103   -1.5319219   -3.1896777
O     3.3867029   -0.6976302   -0.9652066
H     3.7399909   -2.6423169   -3.0786954
H     4.8106595   -1.2273742   -2.9618007
C     3.3464975   -1.1373810   -4.6106907
H     4.0185024   -1.6119397   -5.3578949
H     3.3964063   -0.0364824   -4.7567713
H     2.3052650   -1.4582098   -4.8227570

$vibrational spectrum (first 50 lines)
#  mode     symmetry     wave number   IR intensity    selection rules
#                         cm**(-1)        km/mol          IR     RAMAN
     1        a            -824.57         0.00000        YES     YES
     2                        0.00         0.00000         -       -
     3                        0.00         0.00000         -       -
     4                        0.00         0.00000         -       -
     5                        0.00         0.00000         -       -
     6                        0.00         0.00000         -       -
     7                        0.00         0.00000         -       -
     8        a              17.88         0.77616        YES     YES
     9        a              28.49         0.09588        YES     YES
    10        a              29.80         0.51315        YES     YES
    11        a              35.27         0.20317        YES     YES
    12        a              41.28         0.12699        YES     YES
    13        a              47.50         0.14804        YES     YES
    14        a              52.87         2.03428        YES     YES
    15        a              56.47         0.07271        YES     YES
    16        a              68.68         0.92803        YES     YES
    17        a              76.38         0.60294        YES     YES
    18        a              84.30         1.24385        YES     YES
    19        a              87.24         0.33905        YES     YES
    20        a              87.75         0.31749        YES     YES
    21        a              99.64         0.38492        YES     YES
    22        a             104.07         0.09472        YES     YES
    23        a             111.72         0.11955        YES     YES
    24        a             125.85         0.52948        YES     YES
    25        a             130.72         1.04574        YES     YES
    26        a             135.74         2.92685        YES     YES
    27        a             159.80         0.39549        YES     YES
    28        a             163.46         0.50435        YES     YES
    29        a             175.70         0.60906        YES     YES
    30        a             182.51         0.33070        YES     YES
    31        a             192.22         0.69120        YES     YES
    32        a             201.90         1.24327        YES     YES
    33        a             206.39         0.90828        YES     YES
    34        a             216.46         5.60768        YES     YES
    35        a             219.39         1.23558        YES     YES
    36        a             226.23         0.36249        YES     YES
    37        a             240.01         3.36548        YES     YES
    38        a             250.17         7.14867        YES     YES
    39        a             256.67         1.19476        YES     YES
    40        a             264.84         0.22549        YES     YES
    41        a             272.94         1.71239        YES     YES
    42        a             301.99         1.66941        YES     YES
    43        a             331.88         2.88134        YES     YES
    44        a             338.08        11.63968        YES     YES
    45        a             342.14         0.12354        YES     YES
```

46	a	402.10	7.41533	YES	YES
47	a	404.30	2.03542	YES	YES
48	a	432.75	3.75956	YES	YES
49	a	437.73	1.99154	YES	YES
50	a	446.18	1.04785	YES	YES

10c

SCF Energy (au) BP86/SV(P) -2924.851437947
SCF Energy (au) PBE0/def2-TZVPP -2924.397338119
SCF Energy (au) PBE0/def2-TZVPP -2924.4107658946 (Toluene Correction)
Zero Point Energy (au) 0.4402802
Chemical Potential (kJ mol^{-1}) 970.92
Dispersion Correction (au) PBE0/def2-TZVPP -0.09090716

xyz coordinates
61

Mn	1.5124812	0.3336282	1.1821270
C	0.4880749	1.3367879	2.2584460
C	1.6032667	-0.9560281	2.4312379
C	2.9839276	1.0475001	2.0262104
O	3.7403271	1.6119459	2.7014245
O	1.7087133	-1.7609878	3.2664369
O	-0.1402232	1.9844027	3.0016471
C	0.7641033	2.6382204	-0.8292003
C	3.0362561	2.0725726	-0.7252749
C	3.3858341	3.0420575	-1.6715025
C	2.3719088	3.8374205	-2.2218563
C	1.0564811	3.6295242	-1.7981221
C	-0.6360098	2.4022379	-0.4166004
C	-1.1078504	1.1172284	-0.1329975
C	-2.5131414	0.9403461	0.1944378
C	-3.3071963	2.1046043	0.3874950
O	-2.7920309	3.3473892	0.2128703
C	-1.4997295	3.5831773	-0.2804018
H	3.8100082	1.4362821	-0.2698409
H	4.4426132	3.1621248	-1.9557943
H	2.5974961	4.6107463	-2.9741367
H	0.2331028	4.2300521	-2.2033747
N	1.7656620	1.8711224	-0.2986590
C	0.1045282	-2.5518948	0.2805469
C	-0.1274727	-3.5652723	1.2440210
C	0.1429184	-4.9115862	0.9606816
C	0.6475500	-5.2763124	-0.2988887
C	0.8730312	-4.2838803	-1.2709608
C	0.6066124	-2.9379487	-0.9868668
H	-0.5235342	-3.2873853	2.2348899
H	-0.0432585	-5.6804888	1.7283780
H	0.8587682	-6.3341006	-0.5273898
H	1.2565657	-4.5643658	-2.2659785
H	0.7786408	-2.1787610	-1.7632170
C	-0.2607429	-1.1622655	0.6179830
C	-0.1910928	-0.0461036	-0.2252076
H	0.3645743	-0.1417784	-1.1746109
O	-1.2134152	4.7427671	-0.5077946
C	-4.6658880	2.0465048	0.7432387
C	-3.1935926	-0.3110882	0.2862209
C	-5.2877828	0.7919441	0.8694538
H	-5.1962264	2.9970225	0.8873617
C	-4.5404235	-0.3908074	0.6126204
O	-6.5864317	0.6115337	1.2018593
H	-5.0581927	-1.3599785	0.6725214
H	-2.6502316	-1.2400851	0.0634815
C	-7.3943213	1.7520107	1.4666068
H	-7.4766749	2.4096724	0.5703247
H	-8.3978651	1.3615165	1.7271158

```
H    -6.9951113    2.3434161    2.3229500
O     4.5521334   -0.4777475    0.8501873
C     3.8165296   -1.0190090    0.0055999
C     4.3949188   -2.0242529   -1.0005914
O     2.5416448   -0.7871281   -0.1378444
H     3.8538640   -2.9883588   -0.8647919
H     5.4595906   -2.1877357   -0.7343699
C     4.2616525   -1.5636357   -2.4604208
H     4.6921326   -2.3179209   -3.1557856
H     4.8000756   -0.6047660   -2.6316129
H     3.1963889   -1.4077474   -2.7347452
H    -0.9039368   -1.0763377    1.5104625
```

```
$vibrational spectrum (first 50 lines)
#  mode     symmetry     wave number   IR intensity    selection rules
#                         cm**(-1)       km/mol          IR     RAMAN
     1                        0.00         0.00000         -        -
     2                        0.00         0.00000         -        -
     3                        0.00         0.00000         -        -
     4                        0.00         0.00000         -        -
     5                        0.00         0.00000         -        -
     6                        0.00         0.00000         -        -
     7         a             12.46         0.20650        YES      YES
     8         a             21.88         0.34460        YES      YES
     9         a             31.82         1.00498        YES      YES
    10         a             37.20         0.16191        YES      YES
    11         a             44.53         0.53957        YES      YES
    12         a             46.40         0.12926        YES      YES
    13         a             51.33         1.61504        YES      YES
    14         a             55.07         2.46979        YES      YES
    15         a             63.65         0.41269        YES      YES
    16         a             72.71         0.69104        YES      YES
    17         a             79.11         0.83186        YES      YES
    18         a             87.23         0.70623        YES      YES
    19         a             90.45         0.72809        YES      YES
    20         a             92.48         1.08273        YES      YES
    21         a            100.46         0.46003        YES      YES
    22         a            109.95         0.76197        YES      YES
    23         a            123.91         1.33714        YES      YES
    24         a            136.31         6.97465        YES      YES
    25         a            143.22         7.96025        YES      YES
    26         a            149.05         6.06043        YES      YES
    27         a            154.32         2.02769        YES      YES
    28         a            162.39         1.63508        YES      YES
    29         a            175.76         0.57654        YES      YES
    30         a            184.19         1.37886        YES      YES
    31         a            186.89         5.54747        YES      YES
    32         a            205.96         2.19190        YES      YES
    33         a            213.86         0.33013        YES      YES
    34         a            217.75         2.92924        YES      YES
    35         a            224.95         1.20193        YES      YES
    36         a            247.06         1.81118        YES      YES
    37         a            251.83         0.24427        YES      YES
    38         a            259.77         9.59612        YES      YES
    39         a            266.62         0.84079        YES      YES
    40         a            285.03         3.24684        YES      YES
    41         a            312.28         3.52900        YES      YES
    42         a            331.79         5.16631        YES      YES
    43         a            342.34         8.08994        YES      YES
    44         a            349.08         7.93477        YES      YES
    45         a            402.53         5.17831        YES      YES
```

46	a	403.38	1.76188	YES	YES
47	a	408.21	8.20888	YES	YES
48	a	420.33	22.50807	YES	YES
49	a	433.42	0.99473	YES	YES
50	a	443.11	5.99869	YES	YES

4c-7-a

SCF Energy (au) BP86/SV(P) -2924.812400941
SCF Energy (au) PBE0/def2-TZVPP -2924.364960514
SCF Energy (au) PBE0/def2-TZVPP -2924.377328856 (Toluene Correction)
Zero Point Energy (au) 0.4396646
Chemical Potential (kJ mol^{-1}) 967.31
Dispersion Correction (au) PBE0/def2-TZVPP -0.09353200

xyz coordinates
61

Mn	1.2393560	0.4206751	1.7766721
C	-0.3873218	0.5832637	2.4739421
C	1.4889388	-0.9027148	2.9495971
C	1.9008335	1.5819090	3.0200658
O	2.3007588	2.3038961	3.8439818
O	1.6337851	-1.7036044	3.7896034
O	-1.4211411	0.6473144	3.0158832
C	-0.1030688	2.4139124	-0.2656837
C	2.1431135	2.8256609	0.2442593
C	2.2146001	3.9260371	-0.6082678
C	1.0761377	4.2631085	-1.3566361
C	-0.0815984	3.5085170	-1.1676001
C	-1.4111573	1.7443318	-0.0418968
C	-1.6618974	0.3759269	-0.1706193
C	-3.0411019	-0.0907656	-0.0524219
C	-4.0651575	0.8510463	0.2150922
O	-3.7869206	2.1741620	0.3634759
C	-2.4968965	2.6977364	0.2506246
H	3.0178380	2.5315428	0.8417495
H	3.1508166	4.5012877	-0.6777268
H	1.0845470	5.1107605	-2.0608346
H	-1.0016072	3.7608713	-1.7117167
N	1.0314222	2.0566339	0.4103657
C	1.2936556	-2.0581719	-0.2512673
C	1.8513724	-3.0183979	0.6292313
C	2.4887626	-4.1748909	0.1494958
C	2.6117391	-4.4039629	-1.2310277
C	2.0777967	-3.4618869	-2.1276450
C	1.4293089	-2.3157193	-1.6448455
H	1.7575168	-2.8813675	1.7141736
H	2.8912176	-4.9064852	0.8701217
H	3.1199299	-5.3079026	-1.6061423
H	2.1678626	-3.6181655	-3.2160734
H	1.0314484	-1.5841123	-2.3678809
C	0.5598041	-0.8366881	0.2258049
C	-0.6338370	-0.6339717	-0.4321794
H	-0.9642305	-1.4110845	-1.1513440
O	-2.3852116	3.9019743	0.3953719
C	-5.4162900	0.4868295	0.3573456
C	-3.4413305	-1.4551763	-0.1422309
C	-5.7745775	-0.8675154	0.2413951
H	-6.1415101	1.2840286	0.5674001
C	-4.7691159	-1.8407119	-0.0067571
O	-7.0400520	-1.3387454	0.3613328
H	-5.0685453	-2.8973996	-0.0755043
H	-2.6790882	-2.2305448	-0.3060166
C	-8.0913279	-0.4195053	0.6234184
H	-8.1906445	0.3322887	-0.1941737
H	-9.0190157	-1.0231621	0.6793371

```
H    -7.9386613    0.1102910    1.5925909
H     2.0072955    0.3674151   -0.9057247
O     2.9652221    0.2964645   -1.1993260
C     3.6873024    0.0548965   -0.1067660
C     5.1302136   -0.3093249   -0.3672287
O     3.2144164    0.1010537    1.0385536
H     5.1340225   -1.4170851   -0.5058254
H     5.6895004   -0.1141476    0.5711645
C     5.7757413    0.3722472   -1.5810684
H     6.8220816    0.0191259   -1.7024054
H     5.8014156    1.4772015   -1.4628431
H     5.2215330    0.1408001   -2.5143700

$vibrational spectrum (first 50 lines)
#  mode     symmetry     wave number   IR intensity    selection rules
#                         cm**(-1)        km/mol          IR     RAMAN
     1                      0.00         0.00000         -        -
     2                      0.00         0.00000         -        -
     3                      0.00         0.00000         -        -
     4                      0.00         0.00000         -        -
     5                      0.00         0.00000         -        -
     6                      0.00         0.00000         -        -
     7         a           12.90         0.37662        YES      YES
     8         a           19.19         0.27594        YES      YES
     9         a           25.27         1.34229        YES      YES
    10         a           31.01         0.33071        YES      YES
    11         a           40.09         0.06721        YES      YES
    12         a           41.66         0.05837        YES      YES
    13         a           50.01         0.30046        YES      YES
    14         a           52.47         0.11046        YES      YES
    15         a           66.94         1.42648        YES      YES
    16         a           73.41         0.81051        YES      YES
    17         a           82.87         0.50863        YES      YES
    18         a           86.81         0.55154        YES      YES
    19         a           91.78         0.63162        YES      YES
    20         a           94.00         0.32823        YES      YES
    21         a          101.71         0.54446        YES      YES
    22         a          116.18         1.48178        YES      YES
    23         a          120.44         0.23781        YES      YES
    24         a          135.65         2.85958        YES      YES
    25         a          135.83         0.53178        YES      YES
    26         a          148.75         0.41663        YES      YES
    27         a          155.86         0.03959        YES      YES
    28         a          164.28         0.68581        YES      YES
    29         a          175.19         0.11744        YES      YES
    30         a          180.66         0.57190        YES      YES
    31         a          186.50         0.22128        YES      YES
    32         a          202.20         4.95507        YES      YES
    33         a          209.42         0.53630        YES      YES
    34         a          213.61         0.33465        YES      YES
    35         a          228.54         2.15751        YES      YES
    36         a          232.54         1.57198        YES      YES
    37         a          245.81         0.31752        YES      YES
    38         a          252.48         0.80562        YES      YES
    39         a          265.79         0.37664        YES      YES
    40         a          272.05         0.53237        YES      YES
    41         a          281.00         2.15346        YES      YES
    42         a          309.13         1.85110        YES      YES
    43         a          327.47         4.43262        YES      YES
    44         a          358.21         2.89188        YES      YES
    45         a          392.45         0.15160        YES      YES
```

46	a	406.93	0.77785	YES	YES
47	a	422.49	2.07742	YES	YES
48	a	437.78	2.77846	YES	YES
49	a	445.66	0.60798	YES	YES
50	a	457.73	3.31028	YES	YES

10c_iso

```
SCF Energy (au) BP86/SV(P)                    -2924.836778808
SCF Energy (au) PBE0/def2-TZVPP               -2924.383672847
SCF Energy (au) PBE0/def2-TZVPP               -2924.3963154936 (Toluene
Correction)
Zero Point Energy (au)                        969.22
Chemical Potential (kJ mol-1)                 0.4398892
Dispersion Correction (au) PBE0/def2-TZVPP -0.09055578

xyz coordinates
61

Mn    1.1466897    0.4177324    1.6255812
C    -0.3289157    0.7805172    2.5866699
C     1.4353631   -1.1131393    2.5329531
C     2.0613178    1.2959016    2.9079583
O     2.6372394    1.8370887    3.7593090
O     1.6355322   -2.0745438    3.1565930
O    -1.2396155    1.0350738    3.2692475
C    -0.0685364    2.3935792   -0.4501874
C     2.0770964    2.9008978    0.3278611
C     2.2514537    3.9293922   -0.5994314
C     1.2502096    4.1318034   -1.5556487
C     0.0885163    3.3615720   -1.4730744
C    -1.3945820    1.7448626   -0.2798048
C    -1.6021670    0.4182911    0.1050648
C    -2.9664878   -0.0725057    0.2783631
C    -4.0407841    0.8393973    0.1129393
O    -3.8155711    2.1346260   -0.2172151
C    -2.5415150    2.6621859   -0.4552877
H     2.8807659    2.6627013    1.0371249
H     3.1799155    4.5201919   -0.5868503
H     1.3616295    4.8865603   -2.3509106
H    -0.7267166    3.5157980   -2.1894197
N     0.9685682    2.1267367    0.4033973
C     1.2616769   -2.0826052   -0.6076828
C     0.9225777   -3.2406219    0.1389353
C     1.5007069   -4.4826809   -0.1502044
C     2.4268555   -4.6076517   -1.2025720
C     2.7678409   -3.4746475   -1.9599608
C     2.1977432   -2.2274402   -1.6646412
H     0.1972471   -3.1806881    0.9655828
H     1.2236961   -5.3644207    0.4508302
H     2.8783477   -5.5873825   -1.4304373
H     3.4886228   -3.5605351   -2.7900563
H     2.4802671   -1.3367300   -2.2468663
C     0.6199055   -0.7660655   -0.4213007
C    -0.5199624   -0.5568680    0.3607350
H    -0.8577007   -1.4222466    0.9472372
O    -2.5067108    3.8395111   -0.7571652
C    -5.3891868    0.4734561    0.2698640
C    -3.3318889   -1.4180226    0.5828723
C    -5.7037097   -0.8576198    0.5911743
H    -6.1486672    1.2534588    0.1277362
C    -4.6558382   -1.8067018    0.7368531
O    -6.9596611   -1.3278079    0.7708412
H    -4.9211917   -2.8486469    0.9708121
H    -2.5572266   -2.1908179    0.6862411
C    -8.0540257   -0.4272483    0.6515891
H    -8.1143304    0.0075277   -0.3730546
H    -8.9652147   -1.0258560    0.8490815
```

```
H    -7.9855309    0.3974598    1.3986796
H     0.9470881    0.0018040   -1.1418842
O     3.0177268    0.7567375   -1.3207834
C     3.5955347    0.3444363   -0.3010523
C     5.0943921   -0.0187744   -0.3447359
O     3.0691072    0.1402090    0.8742792
H     5.1391349   -1.1283286   -0.4564347
H     5.5408990    0.1974912    0.6498672
C     5.8646489    0.6626098   -1.4781261
H     6.9192934    0.3097180   -1.5112070
H     5.8790710    1.7676423   -1.3507003
H     5.3890567    0.4536259   -2.4595780

$vibrational spectrum (first 50 lines)
#  mode     symmetry     wave number   IR intensity    selection rules
#                         cm**(-1)        km/mol          IR     RAMAN
     1                       0.00         0.00000         -       -
     2                       0.00         0.00000         -       -
     3                       0.00         0.00000         -       -
     4                       0.00         0.00000         -       -
     5                       0.00         0.00000         -       -
     6                       0.00         0.00000         -       -
     7         a            16.46         0.29358        YES     YES
     8         a            21.67         0.17878        YES     YES
     9         a            27.28         0.59244        YES     YES
    10         a            35.36         0.23945        YES     YES
    11         a            42.63         0.14251        YES     YES
    12         a            46.80         0.64159        YES     YES
    13         a            52.62         0.33329        YES     YES
    14         a            54.57         1.21335        YES     YES
    15         a            69.09         1.58601        YES     YES
    16         a            73.72         1.41859        YES     YES
    17         a            84.16         0.10842        YES     YES
    18         a            86.26         0.14559        YES     YES
    19         a            89.94         0.56919        YES     YES
    20         a            97.11         0.79027        YES     YES
    21         a           108.08         0.18874        YES     YES
    22         a           110.71         4.23796        YES     YES
    23         a           119.53         0.83771        YES     YES
    24         a           125.36         1.54332        YES     YES
    25         a           134.58         4.74663        YES     YES
    26         a           141.69         0.96203        YES     YES
    27         a           152.65         3.30161        YES     YES
    28         a           160.13         6.03292        YES     YES
    29         a           166.17         2.24425        YES     YES
    30         a           169.83         1.84290        YES     YES
    31         a           188.57         1.57499        YES     YES
    32         a           190.93         0.06514        YES     YES
    33         a           209.48         0.49792        YES     YES
    34         a           212.47         1.10769        YES     YES
    35         a           215.89         2.01272        YES     YES
    36         a           227.01         1.51459        YES     YES
    37         a           239.01         5.64779        YES     YES
    38         a           265.15         0.44503        YES     YES
    39         a           267.26         1.51520        YES     YES
    40         a           289.11         6.71692        YES     YES
    41         a           296.30         5.98857        YES     YES
    42         a           305.22         3.02978        YES     YES
    43         a           324.23        10.65051        YES     YES
    44         a           355.17         6.24168        YES     YES
    45         a           356.71         2.38427        YES     YES
```

46	a	405.23	0.05754	YES	YES
47	a	414.46	0.55708	YES	YES
48	a	421.57	1.12816	YES	YES
49	a	438.06	2.45623	YES	YES
50	a	454.84	2.88201	YES	YES

6c$_{PhC≡CH}$

SCF Energy (au) BP86/SV(P) -2656.562804484
SCF Energy (au) PBE0/def2-TZVPP -2656.104937508
SCF Energy (au) PBE0/def2-TZVPP -2656.1165523933 (Toluene Correction)
Zero Point Energy (au) 0.3469287
Chemical Potential (kJ mol^{-1}) 742.41
Dispersion Correction (au) PBE0/def2-TZVPP -0.07459439

xyz coordinates
50

Mn	1.9305527	0.7725666	0.1301740
C	2.3337078	0.5316158	1.8678145
C	3.5149309	0.0408346	-0.3646949
C	2.6752279	2.4285178	0.1284649
O	3.1676352	3.4857160	0.1821149
O	4.5632234	-0.3180299	-0.7130544
O	2.5943925	0.3525440	2.9878603
C	-0.9350690	0.5288330	0.8440566
C	-0.3655851	2.7663333	0.3966540
C	-1.6964153	3.1914640	0.4870594
C	-2.6882580	2.2239225	0.7069336
C	-2.3028750	0.8905508	0.8841026
C	-0.3921331	-0.8309502	0.9946206
C	0.9257608	-0.9551948	0.5637967
C	1.5337535	-2.2852181	0.4572210
O	0.7078174	-3.3879486	0.7579698
C	-0.5133230	-3.2578594	1.3455571
C	-1.1200256	-1.9850691	1.5254244
H	0.4353253	3.4894518	0.1802030
H	-1.9394619	4.2572100	0.3570324
H	-3.7546567	2.5009575	0.7354030
H	-3.0679018	0.1151810	1.0141492
N	0.0143542	1.4791686	0.5471199
C	1.4846614	2.5507004	-2.7696576
C	2.7073684	3.1992564	-3.0801730
C	2.7135818	4.3656591	-3.8568250
C	1.5073411	4.9139008	-4.3292222
C	0.2889625	4.2822612	-4.0239278
C	0.2726850	3.1120031	-3.2511298
H	3.6538178	2.7718301	-2.7146706
H	3.6730227	4.8534176	-4.0954998
H	1.5177509	5.8339130	-4.9366757
H	-0.6605717	4.7030571	-4.3947987
H	-0.6799847	2.6108692	-3.0177148
C	1.4483788	1.3187301	-2.0171544
C	1.2154435	0.1027958	-1.7565917
H	0.9499202	-0.9022127	-2.0846386
O	2.6577767	-2.5493422	0.0734167
C	-2.3304979	-1.9763313	2.2756547
C	-1.1137171	-4.4569467	1.7740935
C	-2.3399627	-4.4078647	2.4575945
H	-0.5742680	-5.3931594	1.5769939
O	-3.0098045	-5.4966060	2.9181246
C	-2.9315094	-3.1479923	2.7291392
H	-2.7938789	-1.0218172	2.5592903
H	-3.8623751	-3.1204929	3.3158179
C	-2.4520197	-6.7827218	2.6947821
H	-1.4588329	-6.8912692	3.1908869
H	-2.3420401	-6.9988099	1.6060783

```
H    -3.1622248   -7.5074249    3.1408242


$vibrational spectrum (first 50 lines)
#  mode     symmetry     wave number   IR intensity    selection rules
#                         cm**(-1)        km/mol         IR     RAMAN
     1                       0.00         0.00000         -       -
     2                       0.00         0.00000         -       -
     3                       0.00         0.00000         -       -
     4                       0.00         0.00000         -       -
     5                       0.00         0.00000         -       -
     6                       0.00         0.00000         -       -
     7         a            10.97         0.16773        YES     YES
     8         a            19.93         0.42105        YES     YES
     9         a            27.99         0.60575        YES     YES
    10         a            32.90         0.29692        YES     YES
    11         a            45.97         0.92913        YES     YES
    12         a            61.27         0.24926        YES     YES
    13         a            71.22         0.39202        YES     YES
    14         a            78.16         0.13563        YES     YES
    15         a            80.83         0.74397        YES     YES
    16         a            85.09         0.38221        YES     YES
    17         a            92.61         0.78927        YES     YES
    18         a            99.53         0.94955        YES     YES
    19         a           110.01         2.19465        YES     YES
    20         a           113.10         0.51266        YES     YES
    21         a           120.94         0.27451        YES     YES
    22         a           132.12         1.83056        YES     YES
    23         a           152.06         3.13004        YES     YES
    24         a           156.77         2.25924        YES     YES
    25         a           170.90         1.72611        YES     YES
    26         a           191.83         0.29330        YES     YES
    27         a           207.46         5.25101        YES     YES
    28         a           216.67         0.21586        YES     YES
    29         a           226.23         1.61891        YES     YES
    30         a           240.29         0.39111        YES     YES
    31         a           268.41         0.12415        YES     YES
    32         a           273.36         1.03148        YES     YES
    33         a           296.78         4.78651        YES     YES
    34         a           316.62        23.46812        YES     YES
    35         a           331.83         4.61229        YES     YES
    36         a           347.51         6.49838        YES     YES
    37         a           373.97         2.40738        YES     YES
    38         a           401.04         0.02426        YES     YES
    39         a           416.38        13.28056        YES     YES
    40         a           428.14         2.60519        YES     YES
    41         a           440.84         1.04353        YES     YES
    42         a           447.79         4.29857        YES     YES
    43         a           456.48         6.70780        YES     YES
    44         a           466.20         9.96297        YES     YES
    45         a           473.42         2.81638        YES     YES
    46         a           478.91        12.82530        YES     YES
    47         a           484.86         5.86863        YES     YES
    48         a           489.41         2.39653        YES     YES
    49         a           493.52        11.59994        YES     YES
    50         a           499.95         9.17984        YES     YES
```

6c$_{arene}$

SCF Energy (au) BP86/SV(P) -2656.539820547
SCF Energy (au) PBE0/def2-TZVPP -2656.086038725
SCF Energy (au) PBE0/def2-TZVPP -2656.0995421911 (Toluene Correction)
Zero Point Energy (au) 0.3463423
Chemical Potential (kJ mol^{-1}) 739.40
Dispersion Correction (au) PBE0/def2-TZVPP -0.07916940

xyz coordinates
50

Mn	1.8058402	1.6064849	-0.1536745
C	2.6639738	1.6095099	1.3888860
C	3.1692293	0.7007979	-0.9294499
C	2.5644078	3.1861039	-0.6470795
O	3.1061377	4.1934950	-0.8951809
O	4.0807471	0.2295058	-1.4752471
O	3.2223547	1.6007524	2.4134617
C	-0.7600356	1.5946781	1.2989586
C	-0.1200023	3.7900108	0.7354442
C	-1.1673390	4.3552939	1.4723221
C	-2.0070208	3.4946556	2.1951281
C	-1.7990341	2.1131605	2.1063999
C	-0.4034671	0.1734317	1.1448878
C	0.8604852	-0.0278337	0.5912423
C	1.4419697	-1.3760030	0.6247780
O	0.6742569	-2.3961056	1.2385118
C	-0.6291481	-2.2287096	1.5861287
C	-1.2541833	-0.9527291	1.5320253
H	0.5968515	4.4328041	0.2018747
H	-1.2900684	5.4488158	1.4995005
H	-2.8129445	3.8932539	2.8326890
H	-2.4177471	1.4299001	2.7009323
N	0.1001161	2.4587844	0.6556425
C	0.7289098	2.1568174	-2.5971663
C	1.6894783	2.2992530	-3.6320378
C	2.1954552	1.1772357	-4.2817592
C	1.7505206	-0.1339516	-3.9280869
C	0.7921684	-0.2734372	-2.9046438
C	0.2825938	0.8637837	-2.2357402
H	2.0315539	3.3048496	-3.9251953
H	2.9412711	1.2869933	-5.0843842
C	2.2621234	-1.2795931	-4.6160538
H	0.4318945	-1.2779616	-2.6353361
H	-0.5601399	0.7413777	-1.5410362
O	2.5390090	-1.7149226	0.2254173
C	-2.6557505	-0.9362508	1.7854212
H	0.2331199	3.0480906	-2.1840994
C	2.7015364	-2.2555593	-5.2127726
H	3.0957586	-3.1217454	-5.7283086
C	-1.3139738	-3.3953464	1.9771759
C	-3.3585235	-2.0823422	2.1497015
H	-3.2279418	-0.0081418	1.6482981
H	-4.4431954	-2.0517061	2.3340750
C	-2.6846085	-3.3235573	2.2769518
H	-0.7428549	-4.3328493	2.0055623
O	-3.4456335	-4.3814991	2.6616470
C	-2.8260757	-5.6511580	2.7990326
H	-2.0112659	-5.6298205	3.5606590
H	-2.4094900	-6.0122701	1.8295916

H -3.6213193 -6.3463463 3.1349054

$vibrational spectrum (first 50 lines)

#		cm**(-1)	km/mol	IR	RAMAN
1		0.00	0.00000	-	-
2		0.00	0.00000	-	-
3		0.00	0.00000	-	-
4		0.00	0.00000	-	-
5		0.00	0.00000	-	-
6		0.00	0.00000	-	-
7	a	14.37	0.28323	YES	YES
8	a	22.89	0.38959	YES	YES
9	a	26.05	0.84457	YES	YES
10	a	45.73	0.97419	YES	YES
11	a	49.82	0.26506	YES	YES
12	a	60.67	0.11626	YES	YES
13	a	64.67	0.10338	YES	YES
14	a	72.14	0.53149	YES	YES
15	a	77.25	0.19516	YES	YES
16	a	80.25	0.02605	YES	YES
17	a	83.18	0.97705	YES	YES
18	a	90.51	0.04644	YES	YES
19	a	102.59	1.55568	YES	YES
20	a	107.34	0.25994	YES	YES
21	a	110.85	0.59620	YES	YES
22	a	115.51	1.64183	YES	YES
23	a	131.79	0.52490	YES	YES
24	a	154.06	1.09870	YES	YES
25	a	159.44	0.62398	YES	YES
26	a	174.83	2.25052	YES	YES
27	a	186.67	0.52515	YES	YES
28	a	197.07	0.70553	YES	YES
29	a	212.92	1.29216	YES	YES
30	a	223.77	0.50800	YES	YES
31	a	228.17	2.53141	YES	YES
32	a	268.64	0.94922	YES	YES
33	a	276.60	0.93478	YES	YES
34	a	297.39	1.43236	YES	YES
35	a	337.40	6.63041	YES	YES
36	a	346.04	5.78632	YES	YES
37	a	364.50	1.75097	YES	YES
38	a	374.72	1.94932	YES	YES
39	a	406.75	0.26465	YES	YES
40	a	421.81	5.42479	YES	YES
41	a	438.58	1.43213	YES	YES
42	a	444.01	0.88637	YES	YES
43	a	456.65	1.25785	YES	YES
44	a	461.76	0.07889	YES	YES
45	a	463.03	4.22297	YES	YES
46	a	476.13	1.77377	YES	YES
47	a	480.57	2.19183	YES	YES
48	a	489.46	1.79761	YES	YES
49	a	499.01	13.71890	YES	YES
50	a	509.32	19.46108	YES	YES

$TS_{6cPhC≡CH}$

SCF Energy (au) BP86/SV(P) -2656.560475820
SCF Energy (au) PBE0/def2-TZVPP -2656.100059998
SCF Energy (au) PBE0/def2-TZVPP -2656.1111345109 (Toluene Correction)
Zero Point Energy (au) 0.3468075
Chemical Potential (kJ mol^{-1}) 747.78
Dispersion Correction (au) PBE0/def2-TZVPP -0.07481216

xyz coordinates
50

Mn	1.8281311	1.2189663	0.7534670
C	1.7628281	0.9798159	2.5400317
C	3.5882912	0.8097226	0.7383581
C	2.2618200	2.9682791	0.9222985
O	2.5510479	4.0900270	1.0738215
O	4.7375583	0.6359100	0.7401443
O	1.7089559	0.8063896	3.6896280
C	-1.0440959	0.4818535	0.5917088
C	-0.7825266	2.8159816	0.4844743
C	-2.1397503	3.0187123	0.2145869
C	-2.9630152	1.8913598	0.0592264
C	-2.4100980	0.6205945	0.2424796
C	-0.3329021	-0.7831613	0.8048063
C	1.0577802	-0.6784748	0.6739542
C	1.8788256	-1.9027555	0.6878183
O	1.2107358	-3.1195767	0.8837643
C	-0.1207057	-3.2055705	1.1636513
C	-0.9583197	-2.0574003	1.1468254
N	-0.2365593	1.5918174	0.6506348
C	1.3844097	2.5878592	-2.3258213
C	2.1642347	3.7741652	-2.3120026
C	1.9627260	4.7703629	-3.2771396
C	0.9740349	4.6192652	-4.2663265
C	0.1882221	3.4529331	-4.2869146
C	0.3875963	2.4475104	-3.3313697
C	1.5819199	1.5098359	-1.3880670
C	1.7085710	0.2591068	-1.1321907
H	-0.0930759	3.6705994	0.5558443
H	-2.5263278	4.0424174	0.0965674
H	-4.0255517	2.0000328	-0.2128960
H	-3.0241185	-0.2737498	0.0753567
H	2.9430657	3.9044380	-1.5464043
H	2.5869797	5.6790246	-3.2551519
H	0.8160243	5.4089084	-5.0192341
H	-0.5891800	3.3225773	-5.0583072
H	-0.2264605	1.5329712	-3.3485430
H	2.0842216	-0.6859235	-1.5355317
C	-2.3018871	-2.2664452	1.5750027
O	3.0745450	-1.9711946	0.4764469
C	-0.5907290	-4.4921652	1.4851368
C	-2.7859290	-3.5273720	1.9112489
H	-2.9778440	-1.4092815	1.6971509
H	-3.8242676	-3.6666450	2.2485780
C	-1.9376213	-4.6626280	1.8466957
H	0.1321996	-5.3181859	1.4598685
O	-2.5032076	-5.8530856	2.1739241
C	-1.6931871	-7.0190402	2.1569463
H	-0.8528270	-6.9453218	2.8868100
H	-1.2783935	-7.2155272	1.1403928

```
H    -2.3561442   -7.8579331    2.4482508


$vibrational spectrum (first 50 lines)
#  mode     symmetry     wave number   IR intensity    selection rules
#                         cm**(-1)        km/mol         IR     RAMAN
     1        a           -158.20        0.00000        YES     YES
     2                       0.00        0.00000         -       -
     3                       0.00        0.00000         -       -
     4                       0.00        0.00000         -       -
     5                       0.00        0.00000         -       -
     6                       0.00        0.00000         -       -
     7                       0.00        0.00000         -       -
     8        a             17.35        0.38325        YES     YES
     9        a             22.47        0.23552        YES     YES
    10        a             25.20        0.14813        YES     YES
    11        a             47.48        0.00721        YES     YES
    12        a             53.03        1.23691        YES     YES
    13        a             69.66        0.10046        YES     YES
    14        a             74.65        0.37028        YES     YES
    15        a             81.75        0.13461        YES     YES
    16        a             88.47        0.19304        YES     YES
    17        a             92.01        0.18625        YES     YES
    18        a             97.65        1.11187        YES     YES
    19        a            110.36        1.21503        YES     YES
    20        a            112.86        1.20420        YES     YES
    21        a            122.78        0.93436        YES     YES
    22        a            131.79        0.95095        YES     YES
    23        a            154.64        1.00129        YES     YES
    24        a            163.03        0.12655        YES     YES
    25        a            179.98        1.39768        YES     YES
    26        a            188.15        0.27105        YES     YES
    27        a            209.00        0.07528        YES     YES
    28        a            211.36        0.74956        YES     YES
    29        a            225.10        0.38966        YES     YES
    30        a            231.83        1.39049        YES     YES
    31        a            261.72        0.47589        YES     YES
    32        a            271.38        0.32096        YES     YES
    33        a            293.95        2.14782        YES     YES
    34        a            312.77        8.63033        YES     YES
    35        a            335.01        0.39878        YES     YES
    36        a            351.08        9.27161        YES     YES
    37        a            373.85        2.44502        YES     YES
    38        a            401.76        0.07538        YES     YES
    39        a            410.03        7.71872        YES     YES
    40        a            429.31        4.81084        YES     YES
    41        a            439.92        1.57440        YES     YES
    42        a            443.89        2.46066        YES     YES
    43        a            453.03        8.02562        YES     YES
    44        a            467.08        8.53180        YES     YES
    45        a            473.04        0.62925        YES     YES
    46        a            478.86        0.77729        YES     YES
    47        a            487.12        2.09769        YES     YES
    48        a            490.03        2.61236        YES     YES
    49        a            497.57       14.08462        YES     YES
    50        a            508.00       33.20896        YES     YES
```

6c-7-a

SCF Energy (au) BP86/SV(P) -2656.600256764
SCF Energy (au) PBE0/def2-TZVPP -2656.144141626
SCF Energy (au) PBE0/def2-TZVPP -2656.1563473280 (Toluene Correction)
Zero Point Energy (au) 0.3490157
Chemical Potential (kJ mol^{-1}) 752.04
Dispersion Correction (au) PBE0/def2-TZVPP -0.0751816

xyz coordinates
50

Mn	1.1089950	1.2206489	-0.1988490
C	1.4397778	1.4729241	1.5842561
C	2.7903956	0.6483401	-0.5341635
C	1.5351177	2.8878965	-0.5738272
O	1.7812216	4.0090883	-0.8032215
O	3.8788059	0.3058243	-0.7656539
O	1.6801791	1.6633472	2.7097720
C	-1.4470487	0.4043096	0.3444174
C	-1.6701140	2.6282422	-0.3555515
C	-3.0642231	2.5667586	-0.2260608
C	-3.6580024	1.3702903	0.2091569
C	-2.8356595	0.2688418	0.5008793
C	-0.4545957	-0.6896549	0.5745841
C	0.2761786	-1.2062975	-0.5144754
C	1.0409261	-2.4712068	-0.3397700
O	1.0942015	-3.0179472	0.9472120
C	0.4409766	-2.4788651	2.0169078
C	-0.3628414	-1.3177758	1.8832802
H	-1.1540209	3.5406632	-0.6940327
H	-3.6723891	3.4522221	-0.4678197
H	-4.7519143	1.2928119	0.3169397
H	-3.2564842	-0.6907266	0.8382050
N	-0.8865982	1.5679209	-0.0688338
C	0.6087601	1.2690399	-3.4007301
C	1.6420870	2.1611284	-3.7860599
C	1.6481459	2.7590730	-5.0547107
C	0.6102965	2.5001054	-5.9680354
C	-0.4311855	1.6286487	-5.6013193
C	-0.4300974	1.0184685	-4.3384126
H	2.4635285	2.3676002	-3.0826826
H	2.4714385	3.4383111	-5.3322622
H	0.6108866	2.9797536	-6.9609815
H	-1.2539961	1.4232779	-6.3068156
H	-1.2542233	0.3444230	-4.0518106
C	0.5989951	0.6150389	-2.0873421
C	0.2403527	-0.6874928	-1.8984379
H	0.0915130	-1.4225846	-2.7151603
C	-1.0017002	-0.8360647	3.0595985
O	1.5947905	-3.0759129	-1.2300860
C	0.6014875	-3.1454048	3.2434954
H	1.2385031	-4.0395100	3.2608969
C	-0.0544668	-2.6479653	4.3848222
C	-0.8609156	-1.4821082	4.2820419
H	-1.6081049	0.0812242	3.0029245
H	-1.3506658	-1.1029920	5.1915652
O	0.0250434	-3.2063777	5.6166554
C	0.8315193	-4.3632577	5.7959723
H	1.8996581	-4.1577144	5.5514862
H	0.4685774	-5.2147401	5.1743078

```
H     0.7468883   -4.6316235    6.8677290


$vibrational spectrum (first 50 lines)
#  mode     symmetry     wave number   IR intensity    selection rules
#                         cm**(-1)        km/mol         IR     RAMAN
     1                      0.00         0.00000         -        -
     2                      0.00         0.00000         -        -
     3                      0.00         0.00000         -        -
     4                      0.00         0.00000         -        -
     5                      0.00         0.00000         -        -
     6                      0.00         0.00000         -        -
     7         a           22.69         0.91197        YES      YES
     8         a           25.40         0.27180        YES      YES
     9         a           36.23         0.24754        YES      YES
    10         a           40.36         1.17457        YES      YES
    11         a           48.59         0.06080        YES      YES
    12         a           58.61         0.70031        YES      YES
    13         a           67.07         0.90752        YES      YES
    14         a           70.92         0.67600        YES      YES
    15         a           78.41         0.12650        YES      YES
    16         a           84.56         0.09137        YES      YES
    17         a           89.18         0.45025        YES      YES
    18         a           97.23         0.31304        YES      YES
    19         a          101.65         1.27589        YES      YES
    20         a          107.03         0.88047        YES      YES
    21         a          130.58         1.89063        YES      YES
    22         a          138.70         0.32079        YES      YES
    23         a          150.37         2.73646        YES      YES
    24         a          160.01         0.60425        YES      YES
    25         a          181.93         1.64857        YES      YES
    26         a          193.61         2.65440        YES      YES
    27         a          204.16         0.11159        YES      YES
    28         a          222.77         0.06231        YES      YES
    29         a          230.33         0.30993        YES      YES
    30         a          268.35         0.90465        YES      YES
    31         a          269.46         0.30948        YES      YES
    32         a          282.94         2.77577        YES      YES
    33         a          286.80         2.32111        YES      YES
    34         a          334.43         2.91288        YES      YES
    35         a          352.56         0.35499        YES      YES
    36         a          373.85         5.51379        YES      YES
    37         a          389.43        16.75376        YES      YES
    38         a          406.76         0.11803        YES      YES
    39         a          414.61         2.31820        YES      YES
    40         a          436.41         0.06989        YES      YES
    41         a          452.72         4.28051        YES      YES
    42         a          459.52         1.40795        YES      YES
    43         a          461.09         2.01383        YES      YES
    44         a          468.18         9.54532        YES      YES
    45         a          476.71         2.98650        YES      YES
    46         a          479.96         2.98744        YES      YES
    47         a          493.16         3.51186        YES      YES
    48         a          496.74         4.07546        YES      YES
    49         a          501.88         9.55382        YES      YES
    50         a          522.59         2.72510        YES      YES
```

TS_{6c-7-a}
SCF Energy (au) BP86/SV(P) -2656.583795524
SCF Energy (au) PBE0/def2-TZVPP -2656.121960778
SCF Energy (au) PBE0/def2-TZVPP -2656.1322621071 (Toluene Correction)
Zero Point Energy (au) 0.3482746
Chemical Potential (kJ mol^{-1}) 755.58
Dispersion Correction (au) PBE0/def2-TZVPP -0.07439802

xyz coordinates
50

C	2.3188099	1.1104869	2.8102989
C	0.1392305	2.2073024	3.2785635
C	0.5086725	2.1040101	0.4113328
C	0.2162112	-0.4314743	3.1460413
C	1.1844851	-0.3113772	0.3226578
C	2.4475697	-1.1032213	0.2793176
C	1.1739703	-3.0165959	-0.5123385
C	-0.0680290	-2.3246662	-0.4876019
C	-0.0773917	-0.9331737	-0.0739886
C	-1.1977585	-0.0729389	-0.0081510
C	-2.3898300	-0.1402248	-0.8006629
H	-2.4417151	-0.8687227	-1.6228897
C	-3.4397431	0.7278397	-0.5733747
H	-4.3474136	0.6869500	-1.1961994
C	-3.3178815	1.7033181	0.4666326
H	-4.1553532	2.3685310	0.7276188
C	-2.1231437	1.8326656	1.1324417
H	-1.9641415	2.6122979	1.8909558
Mn	0.6690100	0.9802616	2.1007241
N	-1.0246073	1.0223512	0.8765124
O	3.3744772	1.2151013	3.2897363
O	-0.1873396	3.0075858	4.0681319
C	1.4318844	1.1160786	0.1139713
O	-0.1104216	-1.3334758	3.8045743
O	2.3564375	-2.4156866	-0.1489947
H	2.4640212	1.3929144	-0.1681113
C	0.3365301	3.4434001	-0.1096402
C	-0.0307452	6.0278002	-1.2197040
C	-0.1907202	5.8220979	0.1628270
C	-0.0148141	4.5458482	0.7145763
C	0.4880157	3.6680819	-1.5077603
C	0.3109485	4.9453369	-2.0525442
H	-0.1712554	7.0333601	-1.6494289
H	-0.4493304	6.6683090	0.8204768
H	-0.1195644	4.3983124	1.8003475
H	0.7478759	2.8176644	-2.1596803
H	0.4402838	5.1008011	-3.1365507
O	3.5518392	-0.6718079	0.5362748
C	-1.2193779	-3.1177728	-0.7758199
C	1.2904034	-4.3668023	-0.8802104
C	0.1349303	-5.0943754	-1.2186054
H	2.2949700	-4.8103922	-0.8727677
O	0.1288110	-6.3977295	-1.5974921
H	-2.2193843	-2.6759453	-0.6694432
C	-1.1293226	-4.4566001	-1.1415417
H	-2.0312418	-5.0497116	-1.3562978
C	1.3653992	-7.0907014	-1.6817826
H	1.8756997	-7.1414119	-0.6916080
H	2.0508116	-6.6161929	-2.4228759

H 1.1192280 -8.1177058 -2.0179479

$vibrational spectrum (first 50 lines)

#		cm**(-1)	km/mol	IR	RAMAN
1	a	-329.19	0.00000	YES	YES
2		0.00	0.00000	-	-
3		0.00	0.00000	-	-
4		0.00	0.00000	-	-
5		0.00	0.00000	-	-
6		0.00	0.00000	-	-
7		0.00	0.00000	-	-
8	a	21.88	0.72489	YES	YES
9	a	38.19	0.16612	YES	YES
10	a	42.60	0.18087	YES	YES
11	a	45.55	0.15830	YES	YES
12	a	51.84	0.47752	YES	YES
13	a	59.35	0.29568	YES	YES
14	a	71.27	0.70801	YES	YES
15	a	82.55	0.18363	YES	YES
16	a	88.91	0.02640	YES	YES
17	a	94.04	0.25291	YES	YES
18	a	99.32	0.85632	YES	YES
19	a	108.76	1.01603	YES	YES
20	a	112.23	0.48754	YES	YES
21	a	119.65	1.53857	YES	YES
22	a	138.65	1.11180	YES	YES
23	a	156.96	1.72357	YES	YES
24	a	177.36	2.12924	YES	YES
25	a	189.52	3.11655	YES	YES
26	a	198.86	0.95106	YES	YES
27	a	212.77	0.43772	YES	YES
28	a	218.84	0.48664	YES	YES
29	a	229.50	6.50597	YES	YES
30	a	257.12	0.45427	YES	YES
31	a	269.75	0.18015	YES	YES
32	a	295.66	1.51366	YES	YES
33	a	310.94	0.10657	YES	YES
34	a	332.15	3.18824	YES	YES
35	a	336.16	8.16397	YES	YES
36	a	374.95	2.37552	YES	YES
37	a	390.74	4.70581	YES	YES
38	a	407.22	1.09287	YES	YES
39	a	414.72	9.95081	YES	YES
40	a	438.07	0.86772	YES	YES
41	a	444.50	1.21423	YES	YES
42	a	460.48	10.42544	YES	YES
43	a	472.25	5.12497	YES	YES
44	a	474.55	0.46735	YES	YES
45	a	476.89	4.59172	YES	YES
46	a	481.46	2.69518	YES	YES
47	a	483.89	1.49571	YES	YES
48	a	495.04	13.08656	YES	YES
49	a	511.54	2.26527	YES	YES
50	a	528.44	8.65006	YES	YES

6c-iso

SCF Energy (au) BP86/SV(P) -2656.600946277
SCF Energy (au) PBE0/def2-TZVPP -2656.146299678
SCF Energy (au) PBE0/def2-TZVPP -2656.1569370822 (Toluene Correction)
Zero Point Energy (au) 0.3503009
Chemical Potential (kJ mol^{-1}) 761.39
Dispersion Correction (au) PBE0/def2-TZVPP -0.07535645

xyz coordinates
50

C	2.8396185	-0.5597292	2.4552299
C	2.1343434	1.7575029	2.8464762
C	1.4214929	1.3322531	0.0281388
C	0.3198003	-0.2098235	3.1974749
C	0.7064478	-0.9693845	0.3289227
C	1.1185648	-2.3827547	0.4472818
C	-1.1185643	-3.0519503	-0.1797387
C	-1.5390600	-1.7282221	-0.4859082
C	-0.7004382	-0.6284792	-0.0209808
C	-1.0311090	0.7049858	0.1582836
C	-2.3124519	1.3461070	0.0819046
H	-3.1626067	0.7434232	-0.2607382
C	-2.5264626	2.6336089	0.5124563
H	-3.5363422	3.0705825	0.4819599
C	-1.4232017	3.3946888	1.0492337
H	-1.5774905	4.4094259	1.4471702
C	-0.1741594	2.8626173	1.0889228
H	0.7010164	3.4024709	1.4724099
Mn	1.4319657	0.4064783	1.9032553
N	0.1048325	1.5371215	0.6276887
O	3.7640857	-1.1562636	2.8355618
O	2.6081279	2.6251540	3.4706767
C	1.7299448	-0.0360230	-0.1086121
O	-0.4116608	-0.6026274	4.0119378
O	0.0921999	-3.3255206	0.4161929
H	2.6849649	-0.3696378	-0.5409387
C	2.2055623	2.4518306	-0.5532772
C	3.7253968	4.5093121	-1.7570721
C	4.3465934	3.5766588	-0.9070717
C	3.5923201	2.5596079	-0.3039749
C	1.5890655	3.3892735	-1.4144735
C	2.3459162	4.4125771	-2.0071894
H	4.3172248	5.3144780	-2.2226698
H	5.4267627	3.6495800	-0.6995709
H	4.0721448	1.8466782	0.3860463
H	0.5122406	3.3075425	-1.6333859
H	1.8521743	5.1363857	-2.6763211
O	2.2536466	-2.7878563	0.5874122
C	-2.7503633	-1.6196135	-1.2266566
C	-1.8958546	-4.1823203	-0.4802941
H	-3.0729252	-0.6360434	-1.5956440
C	-3.5290124	-2.7273207	-1.5518249
H	-4.4600082	-2.6212207	-2.1290952
C	-3.1211503	-4.0248978	-1.1543058
O	-3.9500442	-5.0461705	-1.4903100
H	-1.4948449	-5.1630071	-0.1915591
C	-3.5855824	-6.3697375	-1.1262527
H	-3.4834147	-6.4770301	-0.0209384
H	-2.6323048	-6.6819285	-1.6138074

H -4.4074014 -7.0227820 -1.4820255

$vibrational spectrum (first 50 lines)

#		cm**(-1)	km/mol	IR	RAMAN
1		0.00	0.00000	-	-
2		0.00	0.00000	-	-
3		0.00	0.00000	-	-
4		0.00	0.00000	-	-
5		0.00	0.00000	-	-
6		0.00	0.00000	-	-
7	a	22.89	0.63808	YES	YES
8	a	42.39	0.11871	YES	YES
9	a	43.97	0.06826	YES	YES
10	a	47.21	0.04492	YES	YES
11	a	52.60	0.22882	YES	YES
12	a	60.92	0.60507	YES	YES
13	a	71.01	0.25751	YES	YES
14	a	76.65	0.33819	YES	YES
15	a	85.92	0.07808	YES	YES
16	a	92.22	0.84902	YES	YES
17	a	102.94	0.90432	YES	YES
18	a	114.70	0.18550	YES	YES
19	a	117.51	2.50046	YES	YES
20	a	121.37	0.99300	YES	YES
21	a	144.79	1.76873	YES	YES
22	a	158.81	0.87824	YES	YES
23	a	169.80	0.14247	YES	YES
24	a	180.48	0.99264	YES	YES
25	a	199.57	1.18801	YES	YES
26	a	210.61	1.43608	YES	YES
27	a	221.90	0.18296	YES	YES
28	a	234.17	2.30837	YES	YES
29	a	253.23	1.58471	YES	YES
30	a	268.43	0.39496	YES	YES
31	a	289.21	3.16638	YES	YES
32	a	314.29	2.13316	YES	YES
33	a	346.15	2.16733	YES	YES
34	a	356.40	0.71092	YES	YES
35	a	380.66	4.31554	YES	YES
36	a	402.41	1.53218	YES	YES
37	a	413.81	10.22711	YES	YES
38	a	420.77	5.57333	YES	YES
39	a	429.34	4.31096	YES	YES
40	a	445.40	5.59574	YES	YES
41	a	458.77	12.27728	YES	YES
42	a	461.51	0.88079	YES	YES
43	a	465.40	2.96051	YES	YES
44	a	471.01	4.70647	YES	YES
45	a	473.50	0.75784	YES	YES
46	a	484.71	4.31801	YES	YES
47	a	490.21	10.47036	YES	YES
48	a	502.86	23.14477	YES	YES
49	a	512.32	5.52601	YES	YES
50	a	524.55	11.67582	YES	YES

7c

```
SCF Energy (au) BP86/SV(P)                    -2656.630250302
SCF Energy (au) PBE0/def2-TZVPP               -2656.174836838
SCF Energy (au) PBE0/def2-TZVPP               -2656.1871822155 (Toluene
Correction)
Zero Point Energy (au)                         0.3513550
Chemical Potential (kJ mol-1)                  764.67
Dispersion Correction (au) PBE0/def2-TZVPP    -0.07603513
```

xyz coordinates
50

```
C     1.9485259    2.4324103    1.2662845
C    -0.4499140    1.4698555    1.9952879
C     0.0204573    1.7757468   -0.8621179
C     1.8427931    0.1016528    2.1762563
C     1.2510386   -0.2945041   -0.6670771
C     2.4194384   -1.1863390   -0.8587612
C     1.2504254   -2.8439205    0.4917678
C     0.0831983   -2.0620294    0.6521749
C     0.0062097   -0.7513294   -0.0250637
C    -1.2197455   -0.3104824   -0.6911627
C    -2.3650591   -1.0952413   -0.9470831
H    -2.3562231   -2.1437486   -0.6191123
C    -3.4747595   -0.5537454   -1.6033318
H    -4.3703548   -1.1724047   -1.7746725
C    -3.4278786    0.7786628   -2.0528430
H    -4.2740242    1.2495577   -2.5744706
C    -2.2673387    1.5172103   -1.8302434
H    -2.1477673    2.5568219   -2.1653536
Mn    0.9130815    1.0091292    0.9245016
N    -1.2121154    0.9951310   -1.1499046
O     2.6167231    3.3644292    1.4885844
O    -1.3669678    1.7706996    2.6562974
C     1.2503312    1.0431218   -1.1292654
O     2.4319081   -0.4732100    3.0039261
O     2.3170907   -2.4461035   -0.2953248
H     2.1387764    1.4821438   -1.6058689
C    -0.0733902    3.2360824   -1.1875598
C    -0.2416322    6.0022819   -1.7967190
C     0.4902976    5.1398331   -2.6285655
C     0.5663051    3.7681215   -2.3309895
C    -0.8050552    4.1188603   -0.3574872
C    -0.8933121    5.4852028   -0.6621154
H    -0.2994910    7.0788991   -2.0270908
H     1.0067984    5.5344999   -3.5191952
H     1.1326742    3.0997583   -3.0003849
H    -1.2947373    3.7250584    0.5477560
H    -1.4598190    6.1564556    0.0042726
O     3.4181673   -0.9073709   -1.4860033
C    -0.9108236   -2.5764195    1.5225568
C     1.4139020   -4.0913184    1.1205695
H     2.3522922   -4.6326166    0.9405170
C     0.3930586   -4.5877798    1.9515404
O     0.4450539   -5.7805268    2.6041286
H    -1.8038850   -1.9649319    1.7312070
C    -0.7743834   -3.8116926    2.1558596
H    -1.5502827   -4.1954410    2.8355582
C     1.6075169   -6.5816383    2.4628639
H     2.5175024   -6.0555476    2.8360967
H     1.7718925   -6.8858958    1.4020154
```

```
H     1.4335010   -7.4873886    3.0777447


$vibrational spectrum (first 50 lines)
#  mode     symmetry     wave number   IR intensity    selection rules
#                         cm**(-1)        km/mol          IR     RAMAN
     1                      0.00         0.00000         -        -
     2                      0.00         0.00000         -        -
     3                      0.00         0.00000         -        -
     4                      0.00         0.00000         -        -
     5                      0.00         0.00000         -        -
     6                      0.00         0.00000         -        -
     7         a           27.59         0.74198        YES      YES
     8         a           36.44         1.05887        YES      YES
     9         a           39.06         0.14663        YES      YES
    10         a           47.99         0.69550        YES      YES
    11         a           58.79         0.09813        YES      YES
    12         a           63.67         0.10532        YES      YES
    13         a           69.65         0.71456        YES      YES
    14         a           79.75         0.99426        YES      YES
    15         a           86.14         0.58640        YES      YES
    16         a           90.31         0.25259        YES      YES
    17         a           98.00         0.21827        YES      YES
    18         a          102.44         0.20427        YES      YES
    19         a          107.10         0.87529        YES      YES
    20         a          111.20         0.68295        YES      YES
    21         a          143.23         3.61637        YES      YES
    22         a          158.88         1.36224        YES      YES
    23         a          168.79         2.29016        YES      YES
    24         a          184.42         1.14106        YES      YES
    25         a          199.73         2.47328        YES      YES
    26         a          220.86         0.67634        YES      YES
    27         a          224.53         0.11411        YES      YES
    28         a          244.71         3.12968        YES      YES
    29         a          266.41         0.94921        YES      YES
    30         a          274.77         0.59244        YES      YES
    31         a          294.62         0.26298        YES      YES
    32         a          318.26         2.64167        YES      YES
    33         a          347.74         4.18065        YES      YES
    34         a          390.84         5.11372        YES      YES
    35         a          402.84         5.80188        YES      YES
    36         a          404.76         0.06604        YES      YES
    37         a          419.05         0.49561        YES      YES
    38         a          425.75         2.74248        YES      YES
    39         a          432.59         7.87588        YES      YES
    40         a          450.99         0.82934        YES      YES
    41         a          464.30         7.46944        YES      YES
    42         a          470.98         7.07696        YES      YES
    43         a          477.84         6.81679        YES      YES
    44         a          480.07         0.66897        YES      YES
    45         a          485.15         5.45936        YES      YES
    46         a          493.25         2.46543        YES      YES
    47         a          498.89         1.88374        YES      YES
    48         a          510.48         8.94701        YES      YES
    49         a          519.95         6.93072        YES      YES
    50         a          529.96        12.50663        YES      YES
```

8c

SCF Energy (au) BP86/SV(P) -2924.814230908
SCF Energy (au) PBE0/def2-TZVPP -2924.363976336
SCF Energy (au) PBE0/def2-TZVPP -2924.3769233080 (Toluene Correction)
Zero Point Energy (au) 0.4389289
Chemical Potential (kJ mol^{-1}) 966.15
Dispersion Correction (au) PBE0/def2-TZVPP -0.09353367

xyz coordinates
61

Mn	1.4954891	-0.2352743	0.4407375
C	0.7518860	0.3827935	1.9291213
C	2.3603740	-1.3737539	1.5055652
C	2.8329790	1.0071626	0.5761194
O	3.6774812	1.8029094	0.7013317
O	2.9336754	-2.0822403	2.2418271
O	0.3634248	0.7448325	2.9711470
C	-0.8236432	1.5178120	-0.6877177
C	1.0596448	1.3596172	-2.0569297
C	0.4918890	2.1600144	-3.0505728
C	-0.8054289	2.6563074	-2.8534472
C	-1.4543543	2.3338544	-1.6591708
C	-1.6266286	1.2033944	0.5284387
C	-1.9923677	-0.1051974	0.8356451
C	-3.1057007	-0.3541439	1.7819719
O	-3.6408692	0.7587382	2.4439460
C	-3.1780437	2.0261352	2.2600743
C	-2.1838473	2.3116517	1.2893618
H	2.0666870	0.9391215	-2.1805365
H	1.0686008	2.3870765	-3.9609041
H	-1.3024034	3.2819502	-3.6127017
H	-2.4735033	2.6961071	-1.4572791
N	0.4301614	1.0243939	-0.9004267
C	0.2643585	-2.9005833	-0.4571455
C	0.7903119	-3.9276210	0.3742527
C	1.0689506	-5.2084655	-0.1401958
C	0.8533973	-5.4916059	-1.4998542
C	0.3398575	-4.4849161	-2.3371059
C	0.0450341	-3.2109531	-1.8240463
H	0.9351445	-3.7281207	1.4478156
H	1.4524492	-5.9919632	0.5346964
H	1.0791689	-6.4921615	-1.9035517
H	0.1567463	-4.6975551	-3.4042740
H	-0.3693023	-2.4310256	-2.4838789
C	-0.0636971	-1.5535160	0.0778659
C	-1.3989930	-1.3129947	0.2750451
H	-2.1194318	-2.1449431	0.1445568
C	-1.7646997	3.6684508	1.1855370
O	-3.6082048	-1.4331329	2.0183343
H	2.6117741	-3.1885191	-0.4159249
O	3.3937674	-3.0508320	-1.0349311
C	3.3224644	-1.8670387	-1.6264599
C	4.3516887	-1.6512221	-2.7200500
O	2.5173508	-0.9756940	-1.3181135
H	5.0760781	-0.9063374	-2.3127591
H	3.8264456	-1.1225028	-3.5459955
C	5.0813189	-2.9001268	-3.2228438
H	5.8049868	-2.6233478	-4.0194045
H	4.3708507	-3.6425120	-3.6446013

```
H     5.6417640   -3.3983934   -2.4046283
C    -3.7506756    3.0217778    3.0715706
H    -0.9663275    3.9309326    0.4740104
C    -3.3215081    4.3533615    2.9301950
H    -4.5171354    2.7125381    3.7945065
C    -2.3153772    4.6692378    1.9790900
O    -3.8008501    5.3953649    3.6575806
H    -1.9784095    5.7139577    1.9002681
C    -4.7917828    5.1414032    4.6420163
H    -5.7212107    4.7218432    4.1901177
H    -5.0221924    6.1215226    5.1054687
H    -4.4196122    4.4424314    5.4272361

$vibrational spectrum (first 50 lines)
#  mode     symmetry     wave number   IR intensity    selection rules
#                         cm**(-1)        km/mol          IR     RAMAN
     1                      0.00         0.00000         -       -
     2                      0.00         0.00000         -       -
     3                      0.00         0.00000         -       -
     4                      0.00         0.00000         -       -
     5                      0.00         0.00000         -       -
     6                      0.00         0.00000         -       -
     7         a           18.14         0.08418        YES     YES
     8         a           23.53         1.47138        YES     YES
     9         a           26.78         0.27034        YES     YES
    10         a           36.48         0.24837        YES     YES
    11         a           40.12         0.47735        YES     YES
    12         a           49.22         0.28083        YES     YES
    13         a           50.18         0.03400        YES     YES
    14         a           58.97         1.49094        YES     YES
    15         a           67.23         1.03567        YES     YES
    16         a           70.67         0.80521        YES     YES
    17         a           79.41         0.46036        YES     YES
    18         a           82.14         0.48507        YES     YES
    19         a           88.40         0.07264        YES     YES
    20         a           91.86         0.42494        YES     YES
    21         a          100.66         1.04140        YES     YES
    22         a          103.35         0.24160        YES     YES
    23         a          111.33         0.38567        YES     YES
    24         a          121.61         0.89455        YES     YES
    25         a          128.72         1.28869        YES     YES
    26         a          137.11         0.87212        YES     YES
    27         a          144.80         1.45091        YES     YES
    28         a          154.50         0.16476        YES     YES
    29         a          168.88         2.13251        YES     YES
    30         a          173.83         1.08949        YES     YES
    31         a          184.76         0.66136        YES     YES
    32         a          187.60         2.31908        YES     YES
    33         a          206.37         0.55422        YES     YES
    34         a          219.93         0.69492        YES     YES
    35         a          226.16         1.60568        YES     YES
    36         a          234.13         0.58885        YES     YES
    37         a          245.61         0.28783        YES     YES
    38         a          262.13         0.98165        YES     YES
    39         a          268.93         0.17511        YES     YES
    40         a          276.81         1.13069        YES     YES
    41         a          282.74         0.89971        YES     YES
    42         a          287.31         1.38178        YES     YES
    43         a          338.81         1.50805        YES     YES
    44         a          358.55         0.95521        YES     YES
    45         a          400.38         3.04794        YES     YES
```

46	a	402.46	6.82673	YES	YES
47	a	408.29	3.04478	YES	YES
48	a	431.77	3.24378	YES	YES
49	a	448.50	4.40783	YES	YES
50	a	460.56	0.77831	YES	YES

TS_{8c-7-a}

SCF Energy (au) BP86/SV(P) -2924.804978267
SCF Energy (au) PBE0/def2-TZVPP -2924.349304437
SCF Energy (au) PBE0/def2-TZVPP -2924.3625898647 (Toluene Correction)
Zero Point Energy (au) 0.4353655
Chemical Potential (kJ mol^{-1}) 961.65
Dispersion Correction (au) PBE0/def2-TZVPP -0.09252315

xyz coordinates
61

Mn	1.7703501	-0.7064606	0.8272170
C	0.7872372	-0.0919992	2.1830324
C	2.2928175	-2.1017988	1.8360078
C	3.1917389	0.2336346	1.4279488
O	4.0924669	0.8424229	1.8527804
O	2.6776439	-2.9720521	2.5048438
O	0.1824570	0.2754084	3.1120901
C	0.0433327	1.3319543	-0.8127326
C	2.2964989	1.1902586	-1.3897068
C	2.1222242	1.9602061	-2.5425995
C	0.8258326	2.3711899	-2.8830699
C	-0.2096525	2.0676892	-1.9942174
C	-1.0918542	1.2411642	0.1575855
C	-1.7438242	0.0573450	0.4978090
C	-3.0664347	0.1289312	1.1853458
O	-3.5201939	1.3900988	1.5774091
C	-2.8110149	2.5324383	1.3763218
C	-1.5917415	2.5167787	0.6500336
H	3.2937210	0.8163734	-1.1197875
H	2.9953785	2.2128682	-3.1641947
H	0.6274202	2.9413613	-3.8051812
H	-1.2362287	2.4144193	-2.1862768
N	1.2846609	0.8288505	-0.5562605
C	-0.0046848	-3.3384580	0.4586351
C	-0.5489182	-3.8512371	1.6664469
C	-0.5465641	-5.2252216	1.9483792
C	-0.0073736	-6.1370722	1.0251860
C	0.5251008	-5.6529600	-0.1811856
C	0.5330454	-4.2763032	-0.4582934
H	-0.9628458	-3.1450865	2.4045122
H	-0.9698233	-5.5853881	2.9006470
H	-0.0041459	-7.2179542	1.2431694
H	0.9348624	-6.3572880	-0.9252999
H	0.9171474	-3.9316056	-1.4291833
C	-0.0263148	-1.8512109	0.2390120
C	-1.3018766	-1.3172011	0.3208774
H	-2.1437618	-2.0260657	0.4408022
C	-0.9239371	3.7691378	0.5087595
O	-3.7967671	-0.8112474	1.4195425
H	0.6520239	-1.5691791	-1.0564134
O	1.1604272	-1.7253340	-2.1013871
C	2.4303367	-1.8078020	-1.8492884
C	3.3282834	-2.3396449	-2.9516812
O	2.9247603	-1.4986450	-0.7326028
H	3.3977286	-3.4401733	-2.7778382
H	4.3497434	-1.9426236	-2.7716720
C	2.8394569	-2.0632134	-4.3781577
H	3.5309075	-2.5207656	-5.1181000
H	2.7898584	-0.9723018	-4.5858041

```
H     1.8248170   -2.4825757   -4.5416789
C    -3.3643294    3.7090491    1.9093174
H     0.0449384    3.8082713   -0.0109635
C    -1.4477711    4.9444137    1.0337320
C    -2.6830662    4.9270280    1.7353915
H    -4.3166910    3.6264814    2.4494876
O    -3.1183515    6.1232361    2.2019039
H    -0.9191873    5.9038108    0.9285215
C    -4.3381770    6.1724856    2.9296640
H    -4.4862100    7.2351445    3.2068367
H    -4.2864087    5.5545593    3.8561536
H    -5.1990680    5.8318584    2.3081734

$vibrational spectrum (first 50 lines)
#                        cm**(-1)         km/mol           IR      RAMAN
     1        a           -557.96          0.00000         YES      YES
     2                       0.00          0.00000          -        -
     3                       0.00          0.00000          -        -
     4                       0.00          0.00000          -        -
     5                       0.00          0.00000          -        -
     6                       0.00          0.00000          -        -
     7                       0.00          0.00000          -        -
     8        a             20.63          1.01139         YES      YES
     9        a             27.36          0.74551         YES      YES
    10        a             31.90          0.13391         YES      YES
    11        a             39.60          0.12988         YES      YES
    12        a             44.49          0.47395         YES      YES
    13        a             48.26          0.09063         YES      YES
    14        a             54.85          0.54325         YES      YES
    15        a             59.79          0.25070         YES      YES
    16        a             70.53          0.51876         YES      YES
    17        a             74.87          1.12917         YES      YES
    18        a             84.69          0.32473         YES      YES
    19        a             86.90          0.76311         YES      YES
    20        a             90.57          1.12702         YES      YES
    21        a             96.74          0.59795         YES      YES
    22        a            103.34          0.19459         YES      YES
    23        a            109.85          0.01482         YES      YES
    24        a            124.03          1.14277         YES      YES
    25        a            129.08          2.14560         YES      YES
    26        a            138.95          0.31908         YES      YES
    27        a            153.22          1.24000         YES      YES
    28        a            168.44          0.99246         YES      YES
    29        a            172.36          2.46066         YES      YES
    30        a            186.42          0.17938         YES      YES
    31        a            191.10          4.24953         YES      YES
    32        a            201.14          1.16342         YES      YES
    33        a            209.09          2.57263         YES      YES
    34        a            210.26          3.93988         YES      YES
    35        a            222.60          0.45481         YES      YES
    36        a            235.82          1.80149         YES      YES
    37        a            244.20          0.82704         YES      YES
    38        a            252.41         21.01169         YES      YES
    39        a            264.18          2.50799         YES      YES
    40        a            272.64          1.48893         YES      YES
    41        a            278.65          3.16746         YES      YES
    42        a            288.81          9.86704         YES      YES
    43        a            336.13          9.83127         YES      YES
    44        a            339.33         10.62922         YES      YES
    45        a            355.21          1.88097         YES      YES
    46        a            403.08          0.61868         YES      YES
```

47	a	409.64	6.19949	YES	YES
48	a	425.33	12.67522	YES	YES
49	a	438.48	8.94569	YES	YES
50	a	449.63	2.77588	YES	YES

9c

SCF Energy (au) BP86/SV(P) -2924.807514710
SCF Energy (au) PBE0/def2-TZVPP -2924.358001685
SCF Energy (au) PBE0/def2-TZVPP -2924.3713504342 (Toluene Correction)
Zero Point Energy (au) 0.439458
Chemical Potential (kJ mol^{-1}) 967.57
Dispersion Correction (au) PBE0/def2-TZVPP -0.09354008

xyz coordinates
61

Mn	0.1919104	-1.0326548	1.6996130
C	-0.6640409	0.4309177	2.2413566
C	-0.7683696	-1.8958470	2.9357012
C	1.4822080	-0.7946437	2.9650340
O	2.2833597	-0.6206944	3.7957060
O	-1.3352829	-2.3987732	3.8260546
O	-1.2548938	1.3352020	2.6874464
C	1.1532500	1.0578524	-0.4301443
C	2.7241689	-0.5614460	0.1647839
C	3.6802596	-0.0670600	-0.7229979
C	3.3372975	1.0246015	-1.5351872
C	2.0708426	1.5904391	-1.3696488
C	-0.1465664	1.7738850	-0.2949960
C	-1.3709323	1.1634455	-0.5650243
C	-2.5644936	2.0145432	-0.8219784
O	-2.4309863	3.3895640	-0.6058497
C	-1.2781413	3.9642844	-0.1659988
C	-0.0900058	3.2030638	-0.0212292
H	2.9604301	-1.4164525	0.8142538
H	4.6730936	-0.5404456	-0.7724851
H	4.0456753	1.4359175	-2.2724468
H	1.7629982	2.4622813	-1.9662571
N	1.4714679	-0.0478303	0.3037577
C	-1.8178315	-2.6222849	-0.2377026
C	-2.2660390	-3.5537389	0.7321310
C	-2.8849368	-4.7607796	0.3673247
C	-3.0593528	-5.0944611	-0.9859054
C	-2.6121290	-4.1963032	-1.9708713
C	-2.0070840	-2.9866888	-1.6019869
H	-2.1669201	-3.3190678	1.7990736
H	-3.2386545	-5.4448715	1.1568702
H	-3.5382890	-6.0457558	-1.2717073
H	-2.7336257	-4.4395716	-3.0400513
H	-1.6504213	-2.3070640	-2.3934009
C	-1.1875430	-1.3031119	0.1115064
C	-1.6588413	-0.2591032	-0.6659396
H	-2.5214176	-0.4544343	-1.3322787
C	1.0565954	3.9013373	0.4564395
O	-3.6391195	1.6233192	-1.2268080
H	0.4799778	-1.9377161	-0.8761002
O	1.0370383	-2.7487317	-1.1165755
C	1.3001305	-3.3815561	0.0215491
C	1.8736803	-4.7704191	-0.1370249
O	1.0766663	-2.8850328	1.1379981
H	0.9897561	-5.4461113	-0.2264807
H	2.3685104	-5.0320046	0.8211973
C	2.8026100	-4.9638326	-1.3434530
H	3.1439204	-6.0197648	-1.3947343
H	3.7049733	-4.3184294	-1.2720179

```
H     2.2823187   -4.7210282   -2.2932648
C    -1.3367121    5.3406300    0.1140954
H     1.9919080    3.3467926    0.6288119
C     1.0215914    5.2611757    0.7444426
C    -0.1797913    5.9981364    0.5690400
H    -2.2980748    5.8509446   -0.0314318
O    -0.1168687    7.3193311    0.8721922
H     1.9092308    5.7886451    1.1253249
C    -1.2905235    8.1084380    0.7388276
H    -1.0050336    9.1356294    1.0417075
H    -2.1071032    7.7429539    1.4044438
H    -1.6558445    8.1243802   -0.3147039

$vibrational spectrum (first 50 lines)
#  mode     symmetry     wave number   IR intensity    selection rules
#                         cm**(-1)        km/mol          IR     RAMAN
     1                      0.00         0.00000          -        -
     2                      0.00         0.00000          -        -
     3                      0.00         0.00000          -        -
     4                      0.00         0.00000          -        -
     5                      0.00         0.00000          -        -
     6                      0.00         0.00000          -        -
     7         a           16.18         0.41612         YES      YES
     8         a           17.31         0.33172         YES      YES
     9         a           22.33         1.22663         YES      YES
    10         a           34.62         0.54394         YES      YES
    11         a           38.84         0.13068         YES      YES
    12         a           42.62         0.12611         YES      YES
    13         a           49.96         0.11542         YES      YES
    14         a           58.53         0.38116         YES      YES
    15         a           66.91         0.95348         YES      YES
    16         a           79.30         0.80271         YES      YES
    17         a           82.29         0.58203         YES      YES
    18         a           85.84         0.27780         YES      YES
    19         a           91.40         1.13865         YES      YES
    20         a           92.67         0.57163         YES      YES
    21         a          104.00         0.32852         YES      YES
    22         a          118.62         2.18495         YES      YES
    23         a          122.88         0.40374         YES      YES
    24         a          131.83         0.95403         YES      YES
    25         a          137.05         1.95741         YES      YES
    26         a          147.52         1.41525         YES      YES
    27         a          158.69         0.74339         YES      YES
    28         a          167.60         0.35632         YES      YES
    29         a          172.95         3.31812         YES      YES
    30         a          184.72         1.66575         YES      YES
    31         a          193.46         0.62632         YES      YES
    32         a          195.58         0.78578         YES      YES
    33         a          204.52         0.20272         YES      YES
    34         a          215.74         0.36022         YES      YES
    35         a          224.39         0.66686         YES      YES
    36         a          234.72         0.33043         YES      YES
    37         a          245.35         0.21236         YES      YES
    38         a          267.34         0.31269         YES      YES
    39         a          272.53         0.57761         YES      YES
    40         a          273.49         0.58578         YES      YES
    41         a          277.35         2.49031         YES      YES
    42         a          296.91         2.59777         YES      YES
    43         a          339.35         2.31419         YES      YES
    44         a          360.52         2.08973         YES      YES
    45         a          399.73         0.91556         YES      YES
```

46	a	406.01	4.27079	YES	YES
47	a	407.47	1.30378	YES	YES
48	a	435.07	0.40578	YES	YES
49	a	448.84	3.55721	YES	YES
50	a	462.74	1.49495	YES	YES

8c-7-a

SCF Energy (au) BP86/SV(P) -2924.807514710
SCF Energy (au) PBE0/def2-TZVPP -2924.358001685
SCF Energy (au) PBE0/def2-TZVPP -2924.3713504342 (Toluene Correction)
Zero Point Energy (au) 967.57
Chemical Potential (kJ mol^{-1}) 0.4394582
Dispersion Correction (au) PBE0/def2-TZVPP -0.09354008

xyz coordinates
61

Mn	0.1919104	-1.0326548	1.6996130
C	-0.6640409	0.4309177	2.2413566
C	-0.7683696	-1.8958470	2.9357012
C	1.4822080	-0.7946437	2.9650340
O	2.2833597	-0.6206944	3.7957060
O	-1.3352829	-2.3987732	3.8260546
O	-1.2548938	1.3352020	2.6874464
C	1.1532500	1.0578524	-0.4301443
C	2.7241689	-0.5614460	0.1647839
C	3.6802596	-0.0670600	-0.7229979
C	3.3372975	1.0246015	-1.5351872
C	2.0708426	1.5904391	-1.3696488
C	-0.1465664	1.7738850	-0.2949960
C	-1.3709323	1.1634455	-0.5650243
C	-2.5644936	2.0145432	-0.8219784
O	-2.4309863	3.3895640	-0.6058497
C	-1.2781413	3.9642844	-0.1659988
C	-0.0900058	3.2030638	-0.0212292
H	2.9604301	-1.4164525	0.8142538
H	4.6730936	-0.5404456	-0.7724851
H	4.0456753	1.4359175	-2.2724468
H	1.7629982	2.4622813	-1.9662571
N	1.4714679	-0.0478303	0.3037577
C	-1.8178315	-2.6222849	-0.2377026
C	-2.2660390	-3.5537389	0.7321310
C	-2.8849368	-4.7607796	0.3673247
C	-3.0593528	-5.0944611	-0.9859054
C	-2.6121290	-4.1963032	-1.9708713
C	-2.0070840	-2.9866888	-1.6019869
H	-2.1669201	-3.3190678	1.7990736
H	-3.2386545	-5.4448715	1.1568702
H	-3.5382890	-6.0457558	-1.2717073
H	-2.7336257	-4.4395716	-3.0400513
H	-1.6504213	-2.3070640	-2.3934009
C	-1.1875430	-1.3031119	0.1115064
C	-1.6588413	-0.2591032	-0.6659396
H	-2.5214176	-0.4544343	-1.3322787
C	1.0565954	3.9013373	0.4564395
O	-3.6391195	1.6233192	-1.2268080
H	0.4799778	-1.9377161	-0.8761002
O	1.0370383	-2.7487317	-1.1165755
C	1.3001305	-3.3815561	0.0215491
C	1.8736803	-4.7704191	-0.1370249
O	1.0766663	-2.8850328	1.1379981
H	0.9897561	-5.4461113	-0.2264807
H	2.3685104	-5.0320046	0.8211973
C	2.8026100	-4.9638326	-1.3434530
H	3.1439204	-6.0197648	-1.3947343
H	3.7049733	-4.3184294	-1.2720179

```
H     2.2823187   -4.7210282   -2.2932648
C    -1.3367121    5.3406300    0.1140954
H     1.9919080    3.3467926    0.6288119
C     1.0215914    5.2611757    0.7444426
C    -0.1797913    5.9981364    0.5690400
H    -2.2980748    5.8509446   -0.0314318
O    -0.1168687    7.3193311    0.8721922
H     1.9092308    5.7886451    1.1253249
C    -1.2905235    8.1084380    0.7388276
H    -1.0050336    9.1356294    1.0417075
H    -2.1071032    7.7429539    1.4044438
H    -1.6558445    8.1243802   -0.3147039

$vibrational spectrum (first 50 lines)
#  mode     symmetry     wave number   IR intensity    selection rules
#                         cm**(-1)        km/mol          IR     RAMAN
     1                      0.00         0.00000         -        -
     2                      0.00         0.00000         -        -
     3                      0.00         0.00000         -        -
     4                      0.00         0.00000         -        -
     5                      0.00         0.00000         -        -
     6                      0.00         0.00000         -        -
     7         a           16.18         0.41612        YES      YES
     8         a           17.31         0.33172        YES      YES
     9         a           22.33         1.22663        YES      YES
    10         a           34.62         0.54394        YES      YES
    11         a           38.84         0.13068        YES      YES
    12         a           42.62         0.12611        YES      YES
    13         a           49.96         0.11542        YES      YES
    14         a           58.53         0.38116        YES      YES
    15         a           66.91         0.95348        YES      YES
    16         a           79.30         0.80271        YES      YES
    17         a           82.29         0.58203        YES      YES
    18         a           85.84         0.27780        YES      YES
    19         a           91.40         1.13865        YES      YES
    20         a           92.67         0.57163        YES      YES
    21         a          104.00         0.32852        YES      YES
    22         a          118.62         2.18495        YES      YES
    23         a          122.88         0.40374        YES      YES
    24         a          131.83         0.95403        YES      YES
    25         a          137.05         1.95741        YES      YES
    26         a          147.52         1.41525        YES      YES
    27         a          158.69         0.74339        YES      YES
    28         a          167.60         0.35632        YES      YES
    29         a          172.95         3.31812        YES      YES
    30         a          184.72         1.66575        YES      YES
    31         a          193.46         0.62632        YES      YES
    32         a          195.58         0.78578        YES      YES
    33         a          204.52         0.20272        YES      YES
    34         a          215.74         0.36022        YES      YES
    35         a          224.39         0.66686        YES      YES
    36         a          234.72         0.33043        YES      YES
    37         a          245.35         0.21236        YES      YES
    38         a          267.34         0.31269        YES      YES
    39         a          272.53         0.57761        YES      YES
    40         a          273.49         0.58578        YES      YES
    41         a          277.35         2.49031        YES      YES
    42         a          296.91         2.59777        YES      YES
    43         a          339.35         2.31419        YES      YES
    44         a          360.52         2.08973        YES      YES
    45         a          399.73         0.91556        YES      YES
```

46	a	406.01	4.27079	YES	YES
47	a	407.47	1.30378	YES	YES
48	a	435.07	0.40578	YES	YES
49	a	448.84	3.55721	YES	YES
50	a	462.74	1.49495	YES	YES

9c-iso

SCF Energy (au) BP86/SV(P) -2924.833704225
SCF Energy (au) PBE0/def2-TZVPP -2924.381328266
SCF Energy (au) PBE0/def2-TZVPP -2924.3940675948 (Toluene Correction)
Zero Point Energy (au) 0.4397997
Chemical Potential (kJ mol^{-1}) 970.90
Dispersion Correction (au) PBE0/def2-TZVPP -0.09084541

xyz coordinates
61

Mn	0.1390041	-1.1817288	1.7563854
C	-0.4737061	0.1751449	2.7593581
C	-0.9807556	-2.3338401	2.5693831
C	1.3548992	-1.4676524	3.0483397
O	2.1287516	-1.6533253	3.8964556
O	-1.6770671	-3.0786952	3.1272315
O	-0.8301287	1.0262448	3.4737793
C	1.1213755	1.0247424	-0.2298697
C	2.7290694	-0.5139533	0.4572310
C	3.6449091	-0.0938377	-0.5135532
C	3.2259413	0.8502831	-1.4554575
C	1.9543141	1.4148582	-1.3022085
C	-0.1325960	1.7892882	0.0232713
C	-1.3400456	1.1586108	0.2911695
C	-2.5691750	1.9551071	0.5225906
O	-2.4658721	3.3352861	0.3679054
C	-1.2771046	3.9737195	0.1913479
C	-0.0654028	3.2478166	0.0352978
H	2.9975317	-1.3212046	1.1521055
H	4.6419548	-0.5576173	-0.5541314
H	3.8771661	1.1543166	-2.2910385
H	1.5908628	2.1774659	-2.0072099
N	1.4834024	-0.0056052	0.5933631
C	-1.8200912	-2.5931889	-0.5955426
C	-3.0082084	-2.9610511	0.0869334
C	-3.7041896	-4.1265286	-0.2549334
C	-3.2415946	-4.9530606	-1.2958239
C	-2.0732615	-4.5991328	-1.9905088
C	-1.3681866	-3.4375580	-1.6426014
H	-3.4053678	-2.3294177	0.8969175
H	-4.6226648	-4.3911086	0.2946656
H	-3.7948116	-5.8683181	-1.5647236
H	-1.7042087	-5.2345777	-2.8129357
H	-0.4451760	-3.1684749	-2.1801183
C	-1.0907785	-1.3327323	-0.3498736
C	-1.5954701	-0.2869935	0.4218254
H	-2.5627932	-0.4597347	0.9214286
C	1.1257849	4.0307995	-0.0264502
O	-3.6635328	1.5061889	0.7999622
H	-0.2251961	-1.1763890	-1.0153646
O	1.5940993	-2.4253705	-1.2138319
C	1.5177137	-3.1785907	-0.2253460
C	2.0186619	-4.6344688	-0.3140882
O	1.0238517	-2.8883376	0.9434999
H	1.1131040	-5.2599451	-0.4988652
H	2.3931185	-4.9422140	0.6860119
C	3.0595479	-4.8678139	-1.4116505
H	3.3285295	-5.9450509	-1.4836471
H	3.9940627	-4.2973712	-1.2124318

```
H     2.6750163   -4.5310021   -2.3974122
C    -1.3318182    5.3781241    0.1991371
H     2.1023884    3.5274549   -0.0693687
C     1.0988446    5.4197167   -0.0032918
C    -0.1378694    6.1122262    0.0907424
H    -2.3170456    5.8485986    0.3155612
O    -0.0646960    7.4645394    0.0908211
H     2.0275361    6.0087608   -0.0445577
C    -1.2652218    8.2169928    0.2125653
H    -0.9619353    9.2824065    0.1870414
H    -1.7829622    8.0051552    1.1770109
H    -1.9625078    8.0120434   -0.6325027

$vibrational spectrum (first 50 lines)
#  mode     symmetry     wave number   IR intensity    selection rules
#                         cm**(-1)        km/mol          IR     RAMAN
     1                        0.00         0.00000         -       -
     2                        0.00         0.00000         -       -
     3                        0.00         0.00000         -       -
     4                        0.00         0.00000         -       -
     5                        0.00         0.00000         -       -
     6                        0.00         0.00000         -       -
     7         a             19.77         0.13529        YES     YES
     8         a             29.27         1.08951        YES     YES
     9         a             32.37         0.33874        YES     YES
    10         a             37.86         0.32887        YES     YES
    11         a             44.71         0.21406        YES     YES
    12         a             49.77         0.35820        YES     YES
    13         a             52.57         0.30622        YES     YES
    14         a             60.03         0.93848        YES     YES
    15         a             69.47         1.62825        YES     YES
    16         a             77.09         0.82173        YES     YES
    17         a             83.04         0.13679        YES     YES
    18         a             88.39         0.09586        YES     YES
    19         a             94.13         0.56773        YES     YES
    20         a             96.55         1.41722        YES     YES
    21         a            106.23         0.50396        YES     YES
    22         a            110.29         3.61349        YES     YES
    23         a            117.45         0.01415        YES     YES
    24         a            121.31         0.69953        YES     YES
    25         a            131.70         3.80424        YES     YES
    26         a            140.26         3.31092        YES     YES
    27         a            153.42         4.17844        YES     YES
    28         a            160.90         3.38984        YES     YES
    29         a            161.58         2.61426        YES     YES
    30         a            173.27         0.52095        YES     YES
    31         a            184.39         0.63571        YES     YES
    32         a            194.12         1.49480        YES     YES
    33         a            200.00         1.59333        YES     YES
    34         a            210.76         0.49894        YES     YES
    35         a            219.19         2.81268        YES     YES
    36         a            228.60         1.98133        YES     YES
    37         a            231.15         0.33563        YES     YES
    38         a            268.33         0.50226        YES     YES
    39         a            270.06         3.54350        YES     YES
    40         a            277.08         1.69772        YES     YES
    41         a            289.78         9.17646        YES     YES
    42         a            314.20         9.60603        YES     YES
    43         a            329.24         4.64226        YES     YES
    44         a            334.41         3.91341        YES     YES
    45         a            358.24         2.64569        YES     YES
```

46	a	404.70	0.56090	YES	YES
47	a	408.90	4.07684	YES	YES
48	a	422.60	0.53837	YES	YES
49	a	432.81	2.20998	YES	YES
50	a	451.90	2.45619	YES	YES

Propionic acid

SCF Energy (au) BP86/SV(P) -268.2010978843
SCF Energy (au) PBE0/def2-TZVPP -268.2100799738
SCF Energy (au) PBE0/def2-TZVPP -268.2153669317 (Toluene Correction)
Zero Point Energy (au) 0.0874781
Chemical Potential (kJ mol^{-1}) 151.51
Dispersion Correction (au) PBE0/def2-TZVPP -0.00582436

xyz coordinates
11

```
O     0.1186351    0.4044664    2.2097037
C    -0.3077270    0.5320475    0.9206333
C     0.3520649   -0.4917891    0.0128770
O    -1.1236074    1.3685280    0.5826179
H     0.0205700   -1.4986609    0.3587846
H     1.4481487   -0.4677580    0.2074330
C     0.0293823   -0.2782751   -1.4664552
H     0.5052562   -1.0665606   -2.0885111
H     0.3953538    0.7100834   -1.8179348
H    -1.0666460   -0.3047696   -1.6431447
H    -0.3714307    1.0926879    2.7239963
```

```
$vibrational spectrum
#  mode     symmetry     wave number   IR intensity    selection rules
#                         cm**(-1)        km/mol         IR     RAMAN
     1                        0.00         0.00000        -       -
     2                        0.00         0.00000        -       -
     3                        0.00         0.00000        -       -
     4                        0.00         0.00000        -       -
     5                        0.00         0.00000        -       -
     6                        0.00         0.00000        -       -
     7         a             32.92         0.01211       YES     YES
     8         a            210.75         0.02396       YES     YES
     9         a            243.04         2.70902       YES     YES
    10         a            455.08        18.99315       YES     YES
    11         a            512.46        20.28490       YES     YES
    12         a            601.31        18.61953       YES     YES
    13         a            674.57        86.07056       YES     YES
    14         a            794.52        20.22030       YES     YES
    15         a            802.31        16.47337       YES     YES
    16         a            991.08         4.33073       YES     YES
    17         a           1063.00        50.30643       YES     YES
    18         a           1073.33         1.12329       YES     YES
    19         a           1143.84       227.16479       YES     YES
    20         a           1238.13         0.11486       YES     YES
    21         a           1269.23         0.73349       YES     YES
    22         a           1363.32        24.54236       YES     YES
    23         a           1377.50        29.96208       YES     YES
    24         a           1399.67        13.42765       YES     YES
    25         a           1433.81         9.18633       YES     YES
    26         a           1441.34        15.06700       YES     YES
    27         a           1802.01       242.57944       YES     YES
    28         a           2951.71        10.78765       YES     YES
    29         a           2960.66        20.19435       YES     YES
    30         a           2995.46         4.00341       YES     YES
    31         a           3045.32        16.42188       YES     YES
    32         a           3052.25        14.20139       YES     YES
    33         a           3469.81        34.86138       YES     YES
$end
```

14 Time Resolved Infrared Spectroscopy (TRIR)

15 Variable alkyne concentration studies

Pseudo-first order experiments of **5c** were prepared in a similar fashion to the those in **Table S5** with the exception of the dilution of the alkyne phenylacetylene concentration in toluene, this was prepared from serial dilutions. Samples were then randomised and ran subsequently.

Table S4 Tabulated observed rate constants for k_{IIIobs} and k_2 as a function of phenylacetylene concentration with **5c** in toluene.

$[PhC_2H]$/ mol dm^{-3}	Peak at 2022 cm^{-1} **III** k_{IIIobs} / s^{-1}	Peak at 1916 cm^{-1} **II** k_2 / s^{-1}
0.058	N.D.	$(2.08 \pm 0.82) \times 10^6$
0.116	$(1.53 \pm 0.25) \times 10^7$	$(2.20 \pm 0.67) \times 10^6$
0.232	$(2.68 \pm 0.05) \times 10^7$	$(2.32 \pm 0.82) \times 10^6$
0.464	$(3.85 \pm 0.09) \times 10^7$	$(2.84 \pm 0.51) \times 10^6$

The quality of the data recorded at alkyne concentrations of 0.058 mol dm^{-3} was not sufficient to determine the rate constants for the formation of **I**.

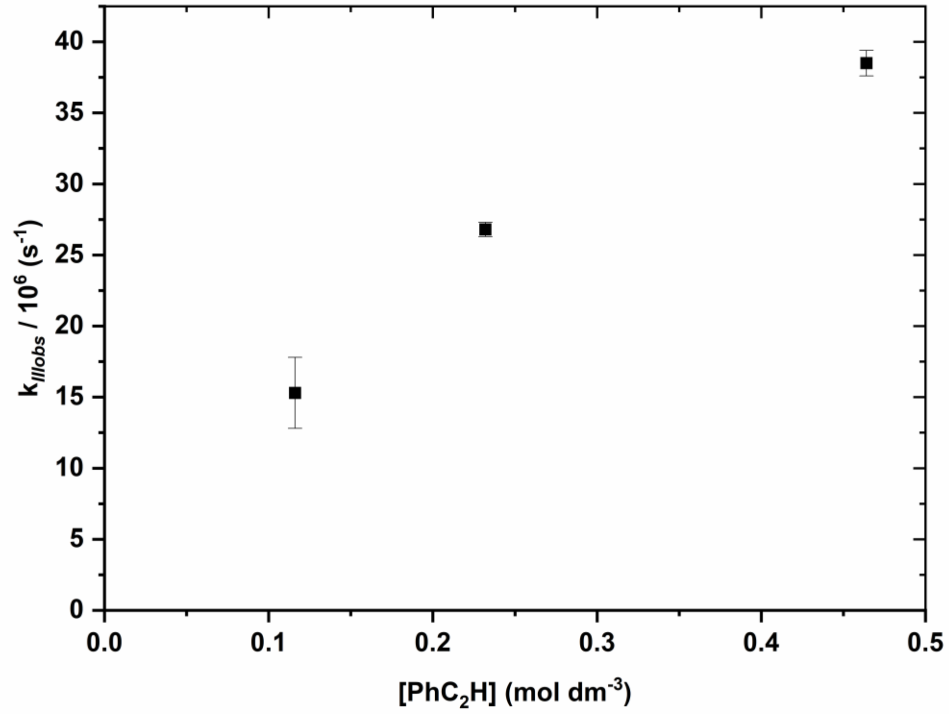

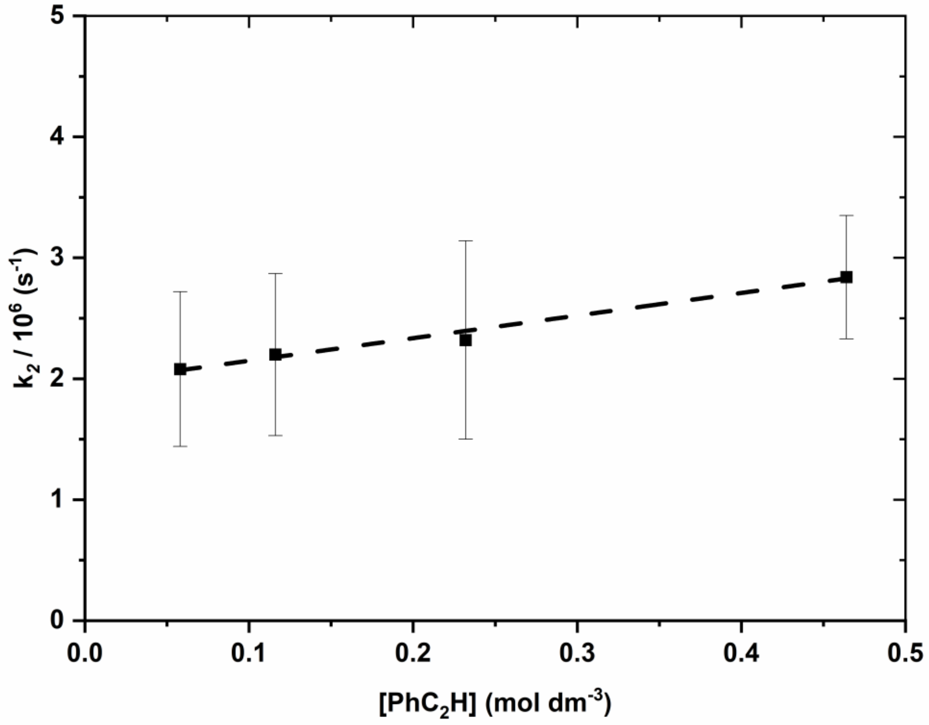

Figure S6 Plot of k_{IIIobs} *versus* [PhC_2H] determined from experiments of **6c** in toluene, error bars indicate 95% confidence for the rate constants. Bottom plot of k_2 *versus* [PhC_2H]

determined from experiments of **6c** in toluene, error bars indicate 95% confidence for the rate constants.

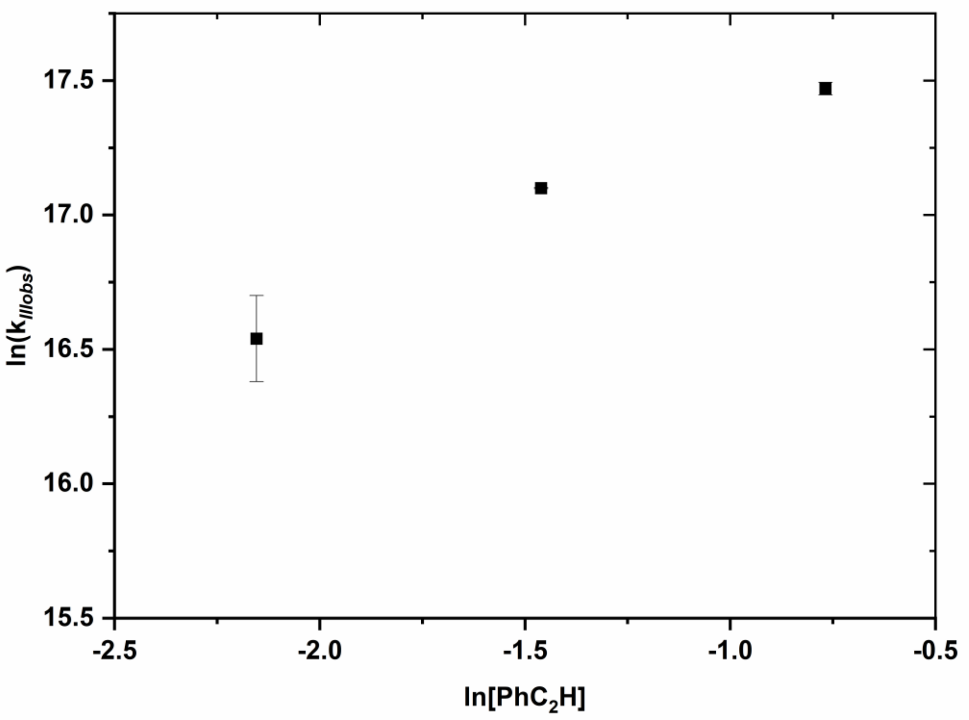

Figure S7 Plot of ln(k_{IIIobs}) *versus* ln([PhC_2H]) determined from experiments of **6c** in toluene, error bars indicate 95% confidence for the rate constants.

Further analysis could be completed on a logarithmic plot of ln(k_{IIIobs}) *versus* ln([PhC_2H]) which gave a linear fit of y = mx + c demonstrating the order with respect to phenylacetylene for the conversion of **III** to **I** was (0.54 ± 0.04), approximately first-order.

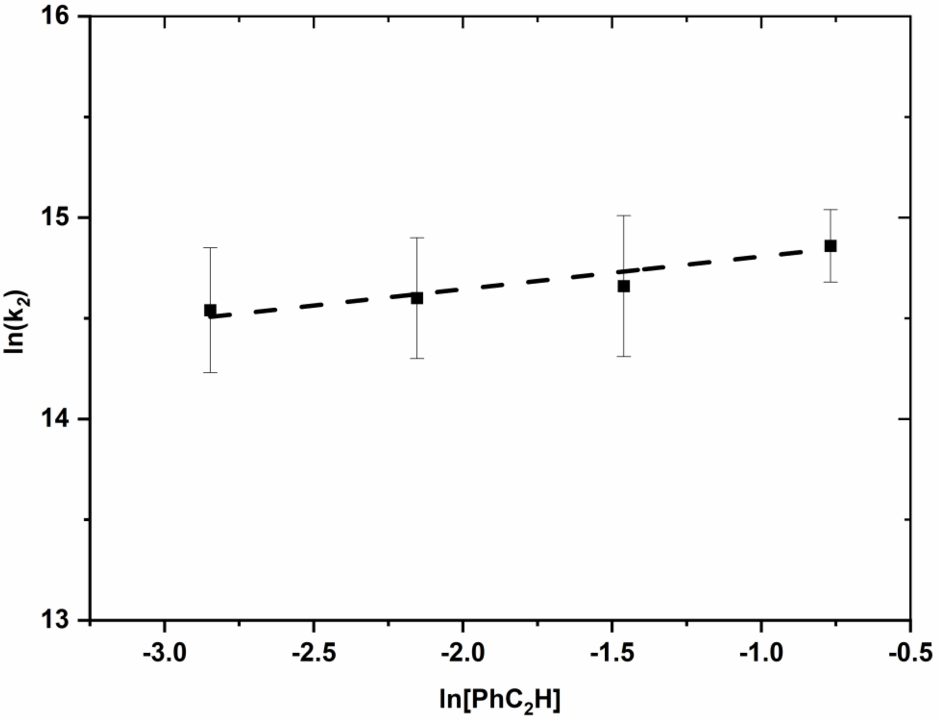

Figure S8 Plot of ln(k_2) *versus* ln([PhC_2H]) determined from experiments of **5c** in toluene, error bars indicate 95% confidence for the rate constants.

Further analysis could be completed on a logarithmic plot of ln(k_2) *versus* ln([PhC_2H]) which gave a linear fit of y = mx + c demonstrating the order with respect to phenylacetylene for the conversion of **I** to **II** was (0.16 ± 0.03), approximately zero-order.

16 Studies in neat Phenylacetylene (PhC_2H)

All cyclometalated coumarin compounds were studied by preparing solutions in neat phenylacetylene at concentration of 1.52 mmol dm^{-3}. The resulting solution was then studied for observed kinetics, kinetics of compound **2a** in phenylacetylene have previously been reported.[4]

Table S5 Reference Ground State Metal Carbonyl Frequencies of Cyclometalated Compounds in Phenylacetylene.

Compound	*7*-R =	v_1 / cm^{-1}	v_2 / cm^{-1}	v_3 / cm^{-1}	v_4 / cm^{-1}	σ_{para}[22]
**2a**	$-NEt_2$	1936	1981	2003	2081	-0.72
**2c**	-OMe	1939	1985	2005	2083	-0.27
**2e**	-Me	1940	1986	2005	2083	-0.17
**2b**	-H	1939	1986	2005	2083	0
**2f**	$-CO_2Me$	1945	1990	2009	2086	0.45
**2d**	$-CF_3$	1944	1988	2008	2085	0.53
**2g**	$-NO_2$	1943	1989	2008	2085	0.78
**6c**	-OMe	1949	1998*		2081	-0.27
**6e**	-Me	1950	1997*		2083	-0.17
**2b'**	-H	1936	1979	2002	2095	0
**2a**-^{13}C	$-NEt_2$	1935	1981	2002	2081	-0.72

*Likely two bands of similar frequency when recorded in phenylacetylene.

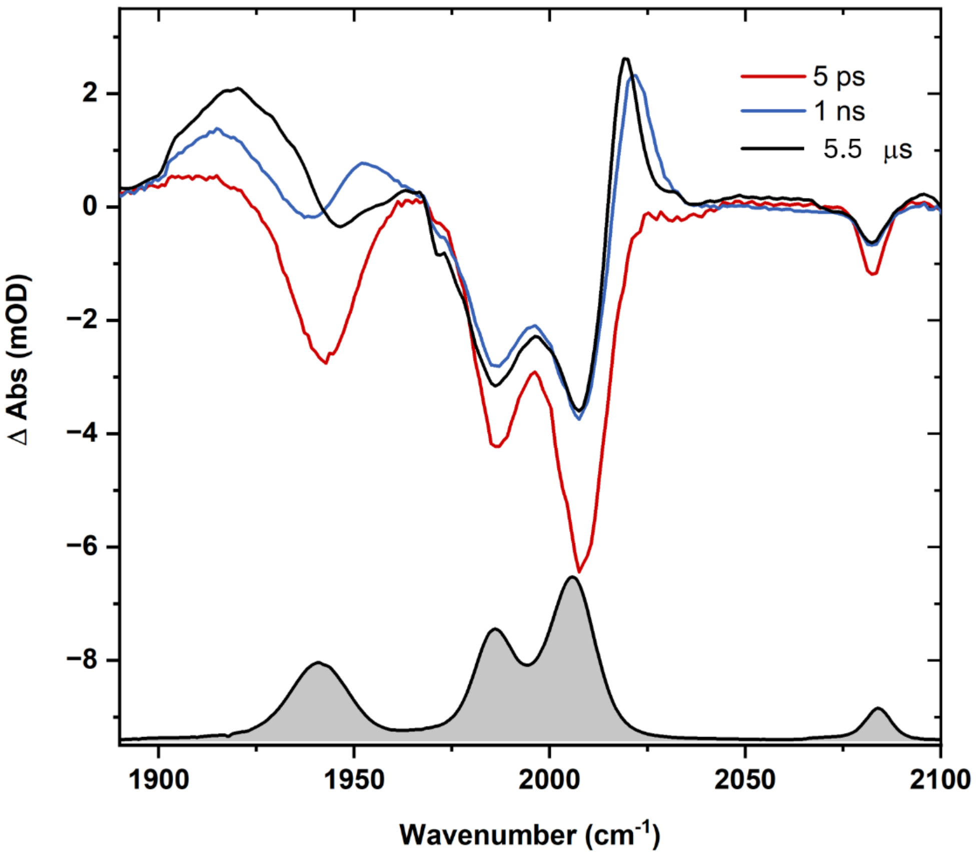

Figure S9 TRIR spectra for the reaction of **2b** in neat PhC_2H at 5 ps, 1 ns and 5.5 μs showing the formation of **III_{alkyne}**, **I** and **II** respectively. In the spectrum at 5 ps, the absorbance at *ca*. 1963 cm^{-1} is assigned to a short-lived ^{3}MLCT state. The ground state spectrum of **2b** is shown in grey at the bottom of the figure.

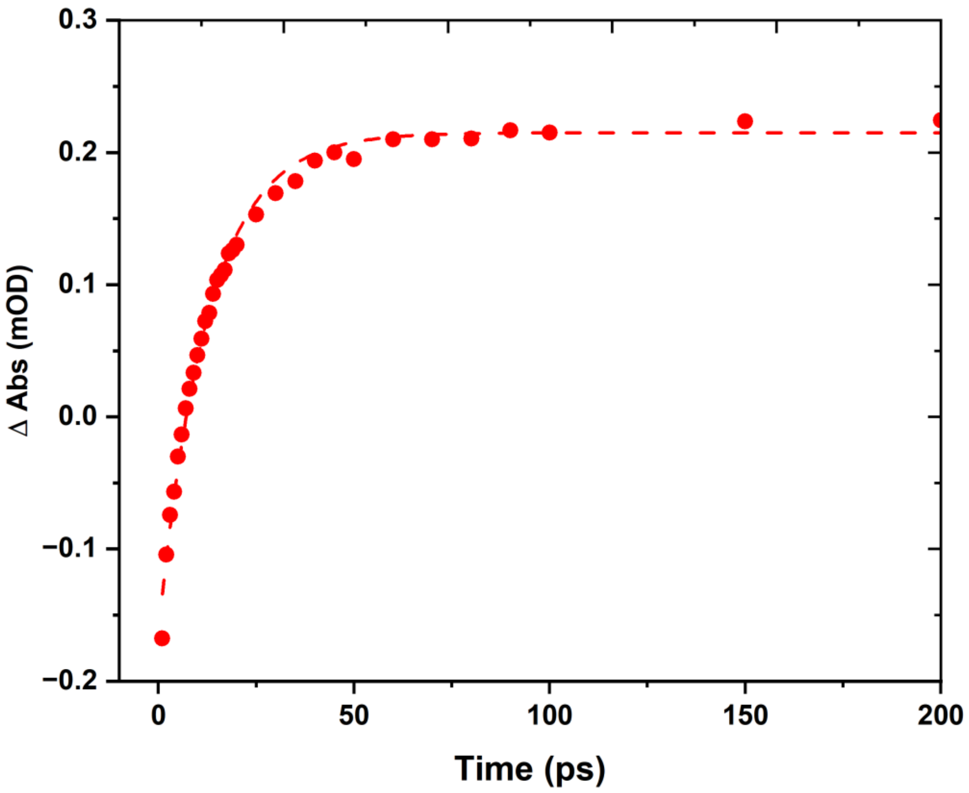

Figure S10 Kinetic profile showing the formation of **I** for a sample of **2b** in neat PhC_2H using the change in intensity of the peak at 2020 cm^{-1}. The experimental data points are shown as red circles and the fit to a monoexponential function with k = (5.92 ± 0.22) × 10^{10} s^{-1}, R^2 = 0.994, as a red dashed line.

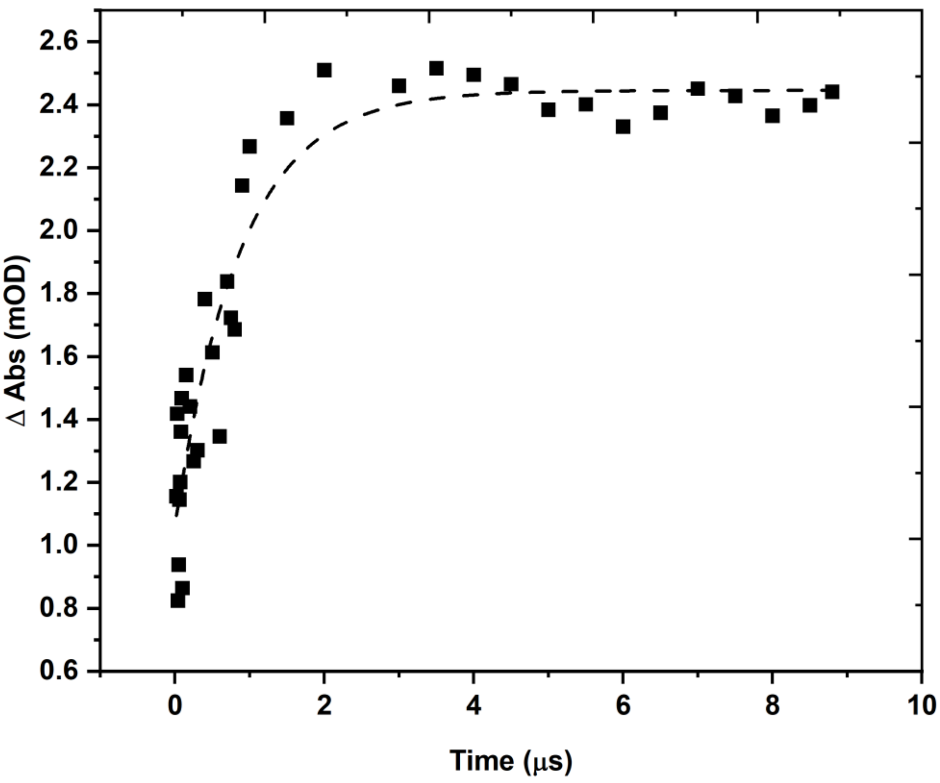

Figure S11 Kinetic profile showing the formation of **II** for a sample of **2b** in neat PhC_2H using the change in intensity of the peak at 1924 cm^{-1}. The experimental data points are shown as black squares and the fit to a monoexponential function with k = (11.4 ± 1.7) × 10^5 s^{-1}, R^2 = 0.904, as a black dashed line.

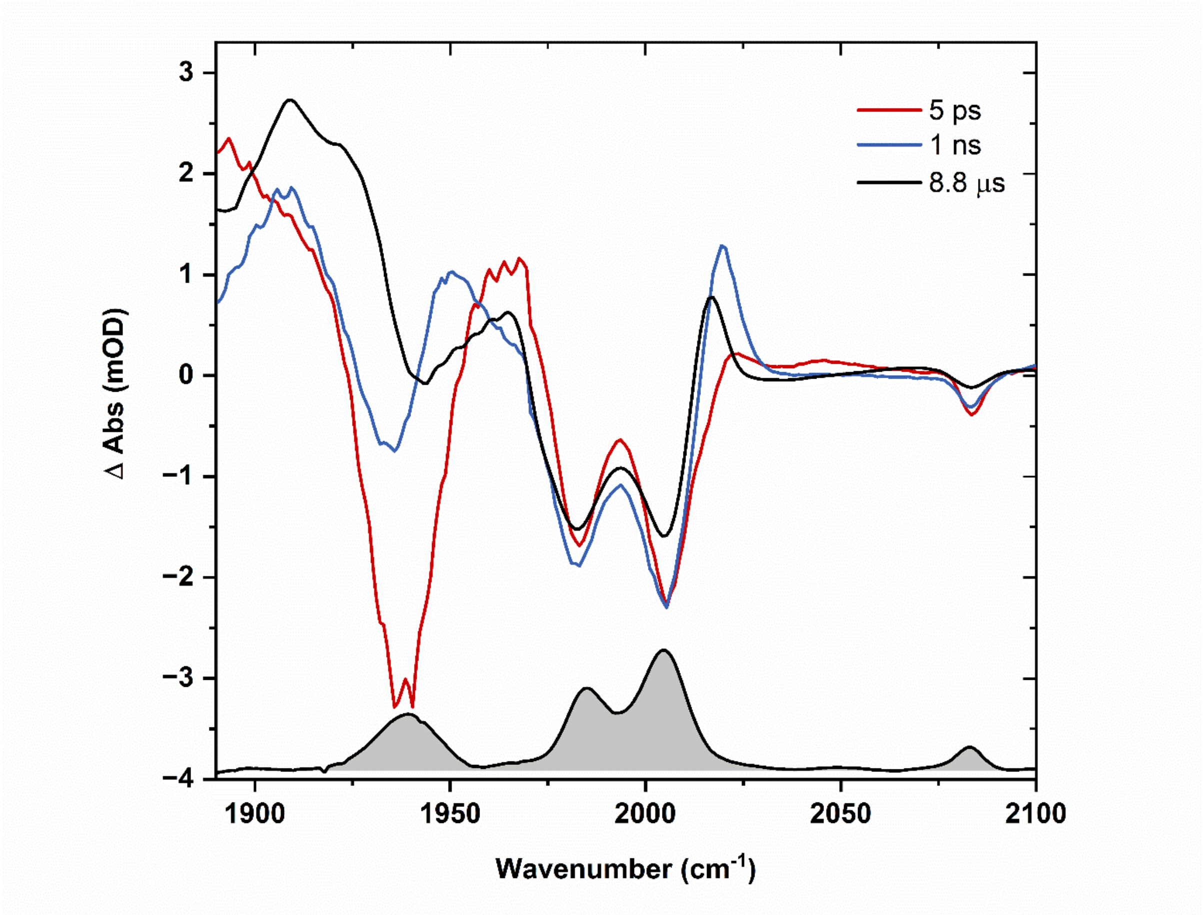

Figure S12 TRIR spectra for the reaction of **2c** in neat PhC_2H at 5 ps, 1 ns and 8.8 μs showing the formation of **III$_{alkyne}$**, **I** and **II** respectively. In the spectrum at 5 ps, the absorbance at *ca*. 1963 cm^{-1} is assigned to a short-lived ^{3}MLCT state. The ground state spectrum of **2c** is shown in grey at the bottom of the figure.

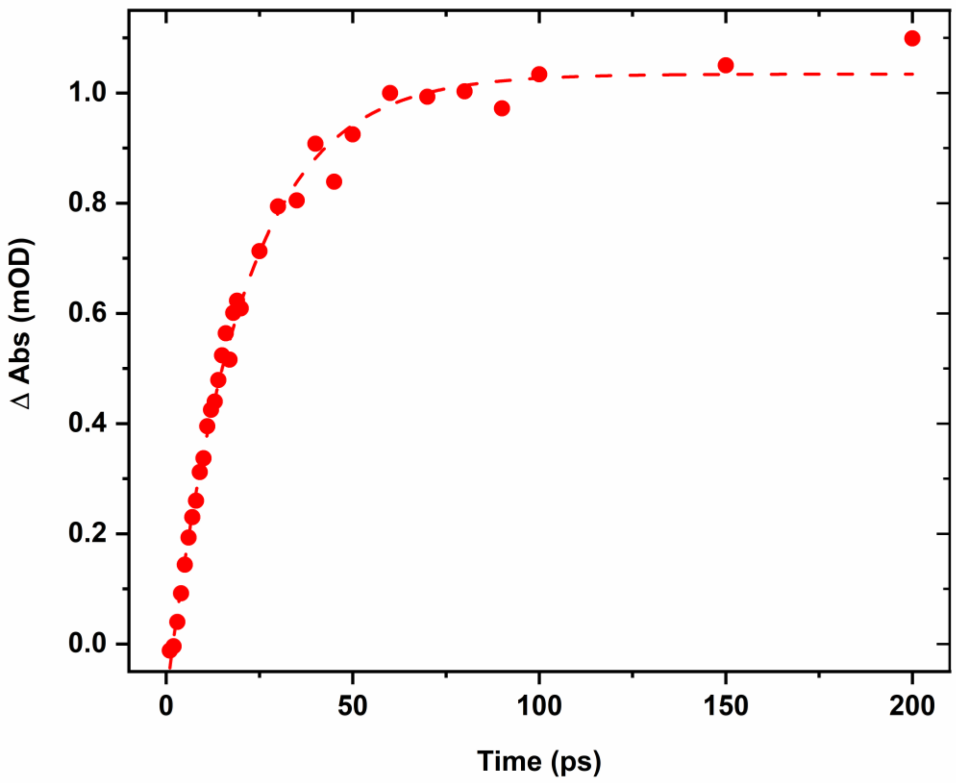

Figure S13 Kinetic profile showing the formation of I for a sample of **2c** in neat PhC_2H using the change in intensity of the peak at 2020 cm^{-1}. The experimental data points are shown as red circles and the fit to a monoexponential function with $k = (5.01 \pm 0.37) \times 10^{10}$ s^{-1}, $R^2 = 0.994$ as a red dashed line.

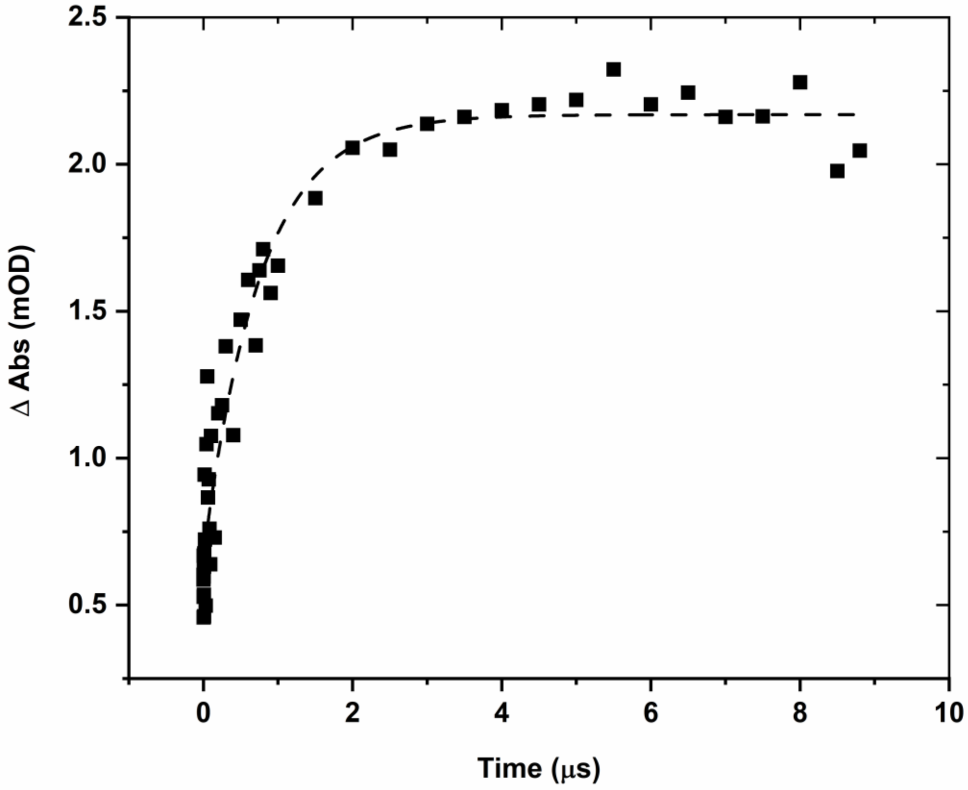

Figure S14 Kinetic profile showing the formation of **II** for a sample of **2c** in neat PhC_2H using the change in intensity of the peak at 1922 cm^{-1}. The experimental data points are shown as black squares and the fit to a monoexponential function with k = (13.2 ± 2.9) × 10^5 s^{-1}, R^2 = 0.944, as a black dashed line.

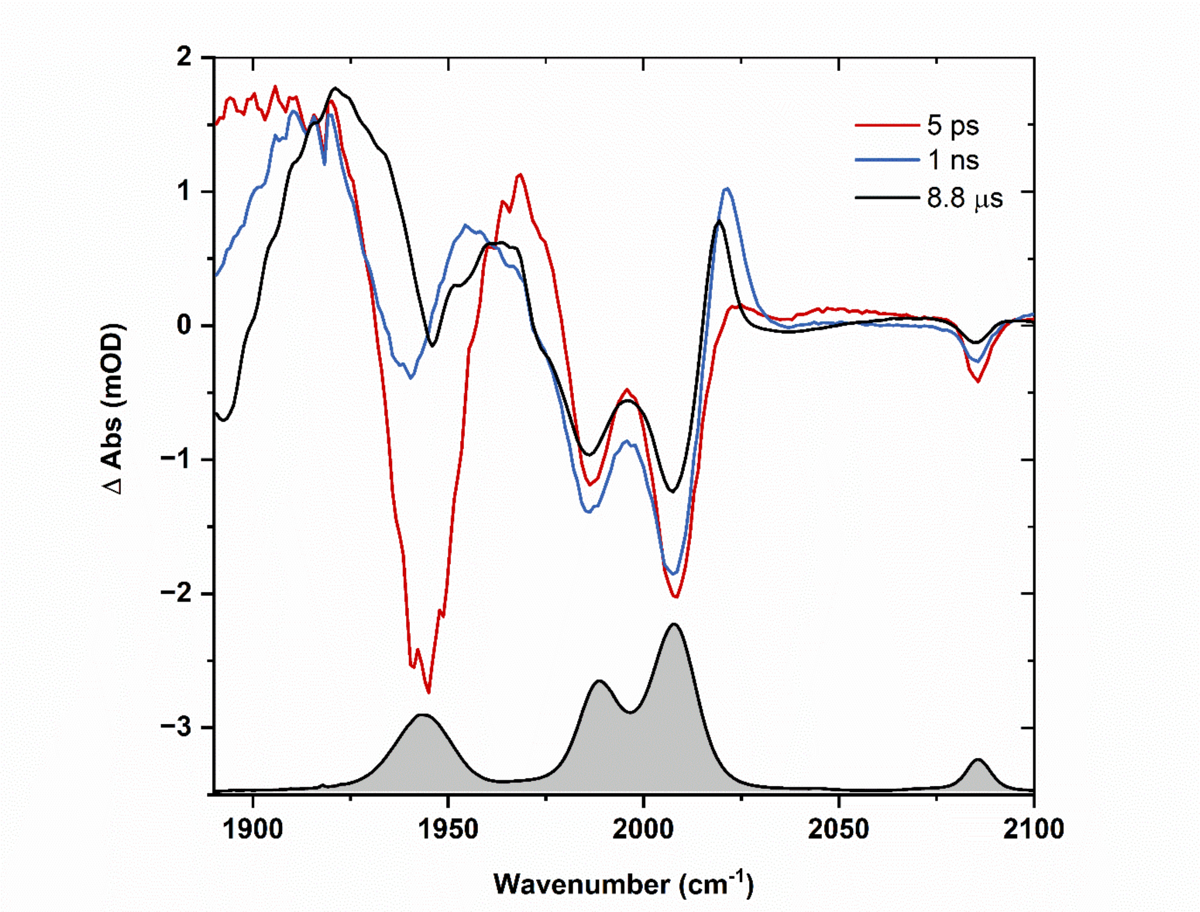

Figure S15 TRIR spectra for the reaction of **2d** in neat PhC_2H at 5 ps, 1 ns and 8.8 μs showing the formation of $\mathbf{III_{alkyne}}$, **I** and **II** respectively. In the spectrum at 5 ps, the absorbance at *ca*. 1968 cm^{-1} is assigned to a short-lived ^{3}MLCT state. The ground state spectrum of **2d** is shown in grey at the bottom of the figure.

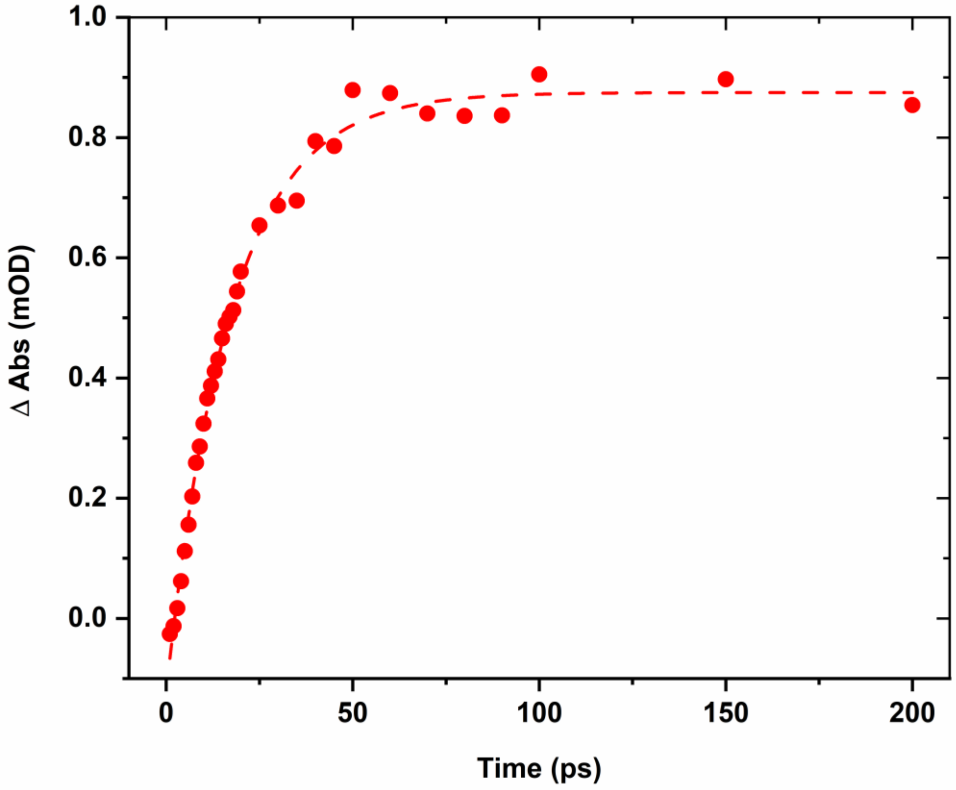

Figure S16 Kinetic profile showing the formation of **I** for a sample of **2d** in neat PhC_2H using the change in intensity of the peak at 2021 cm^{-1}. The experimental data points are shown as red circles and the fit to a monoexponential function with $k = (5.83 \pm 0.39) \times 10^{10}\ s^{-1}$, $R^2 = 0.994$ as a red dashed line.

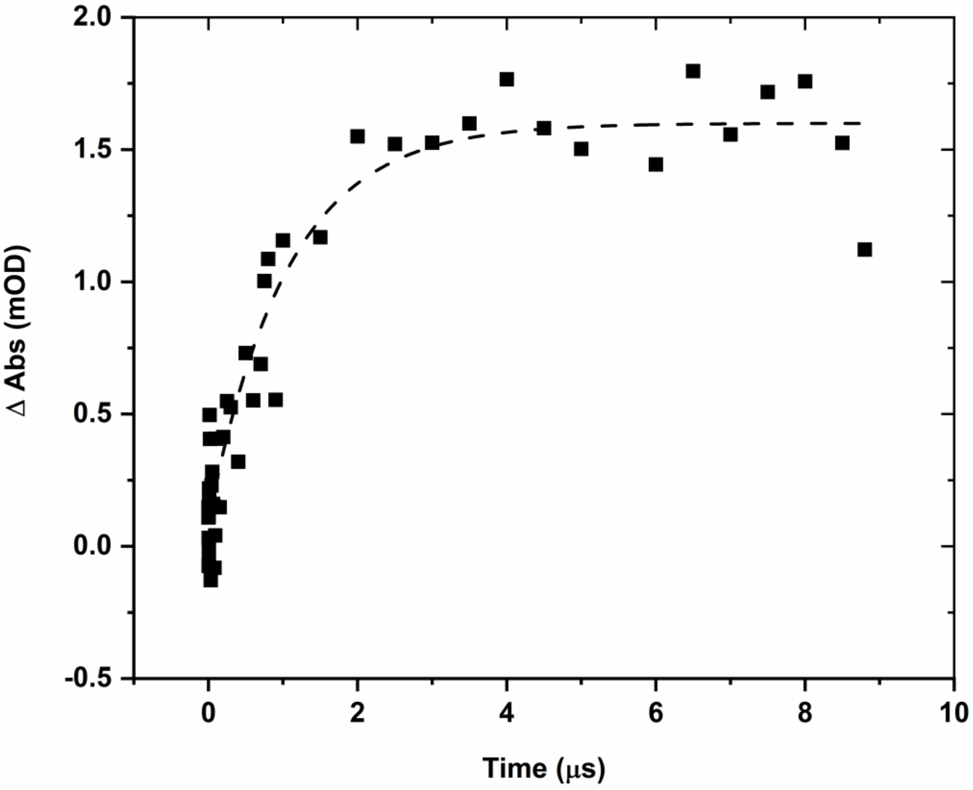

Figure S17 Kinetic profile showing the formation of **II** for a sample of **2d** in neat PhC_2H using the change in intensity of the peak at 1932 cm^{-1}. The experimental data points are shown as black squares and the fit to a monoexponential function with k = (9.43 ± 2.51) × 10^5 s^{-1}, R^2 = 0.924, as a black dashed line.

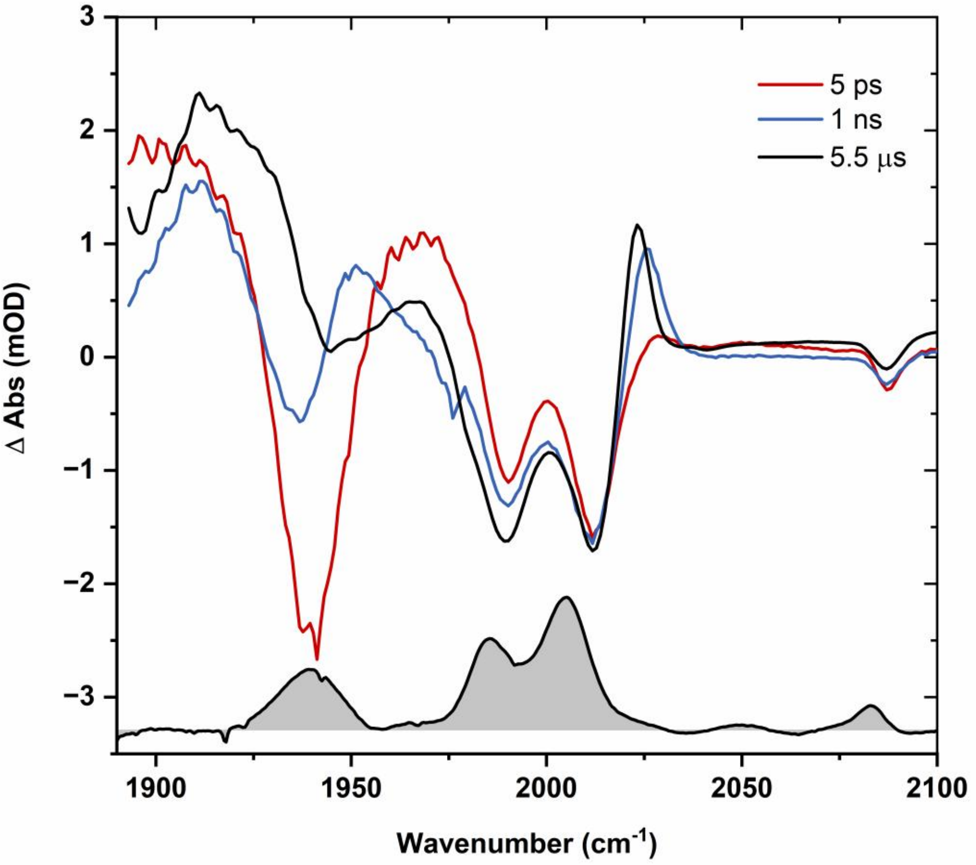

Figure S18 TRIR spectra for the reaction of **2e** in neat PhC_2H at 5 ps, 1 ns and 5.5 μs showing the formation of **III$_{\mathbf{alkyne}}$**, **I** and **II** respectively. In the spectrum at 5 ps, the absorbance at *ca*. 1966 cm^{-1} is assigned to a short-lived ^{3}MLCT state. The ground state spectrum of **2e** is shown in grey at the bottom of the figure.

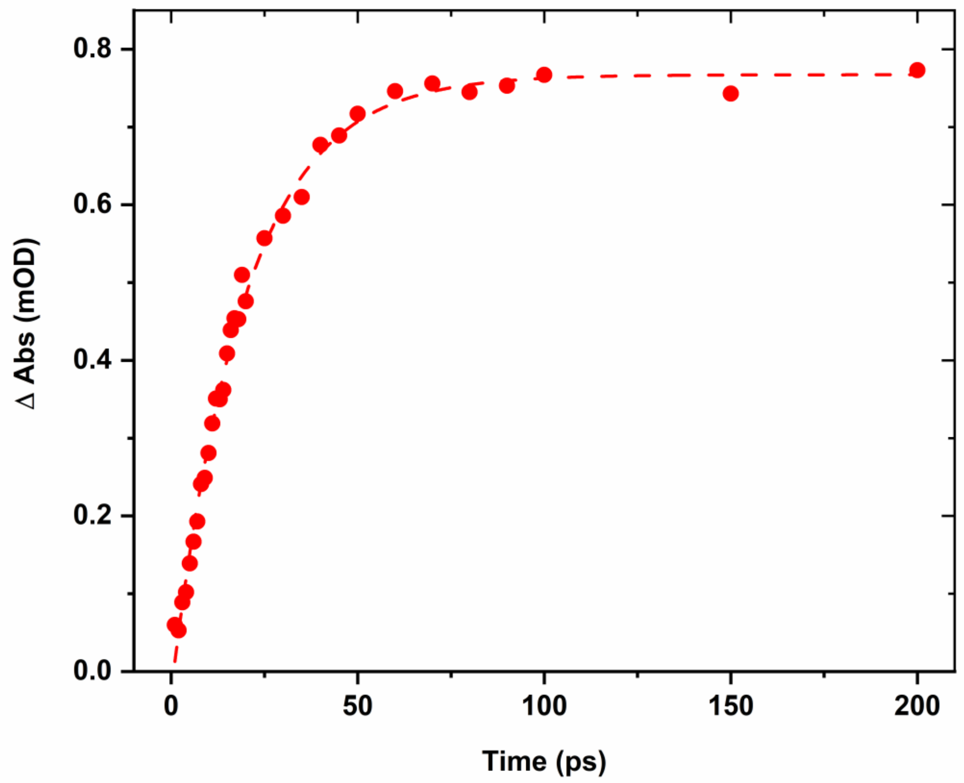

Figure S19 Kinetic profile showing the formation of **I** for a sample of **2e** in neat PhC_2H using the change in intensity of the peak at 2026 cm^{-1}. The experimental data points are shown as red circles and the fit to a monoexponential function with $k = (5.15 \pm 0.34) \times 10^{10}$ s^{-1}, $R^2 = 0.995$ as a red dashed line.

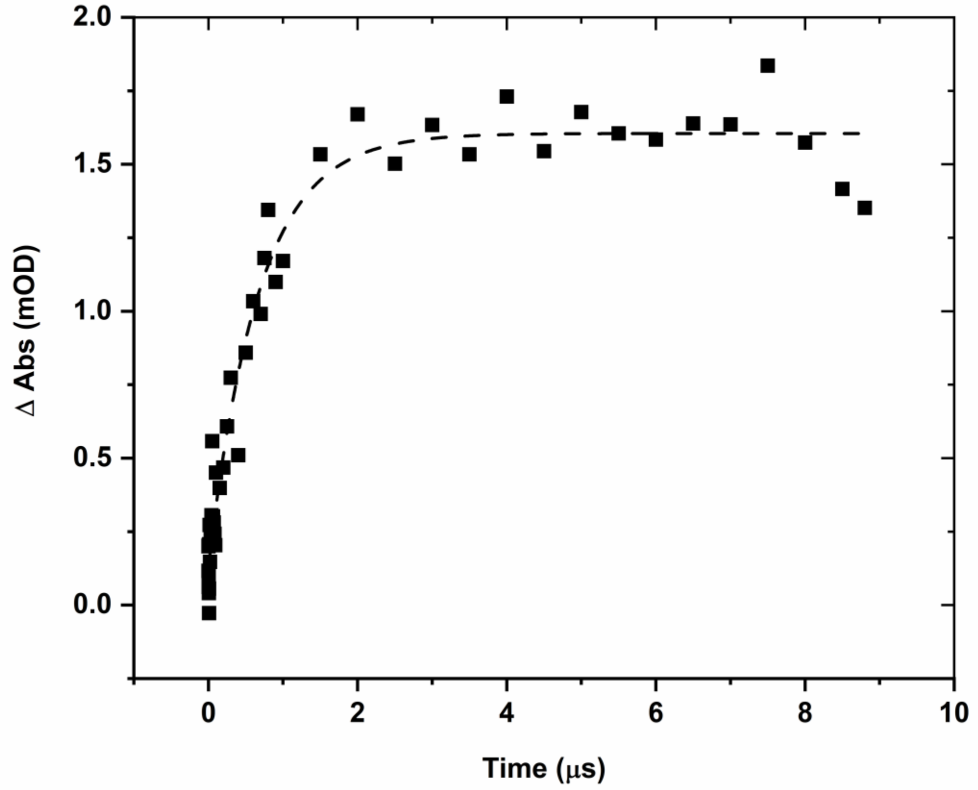

Figure S20 Kinetic profile showing the formation of **II** for a sample of **2e** in neat PhC_2H using the change in intensity of the peak at 1928 cm^{-1}. The experimental data points are shown as black squares and the fit to a monoexponential function with $k = (14.9 \pm 2.5) \times 10^5\ s^{-1}$, $R^2 = 0.966$, as a black dashed line.

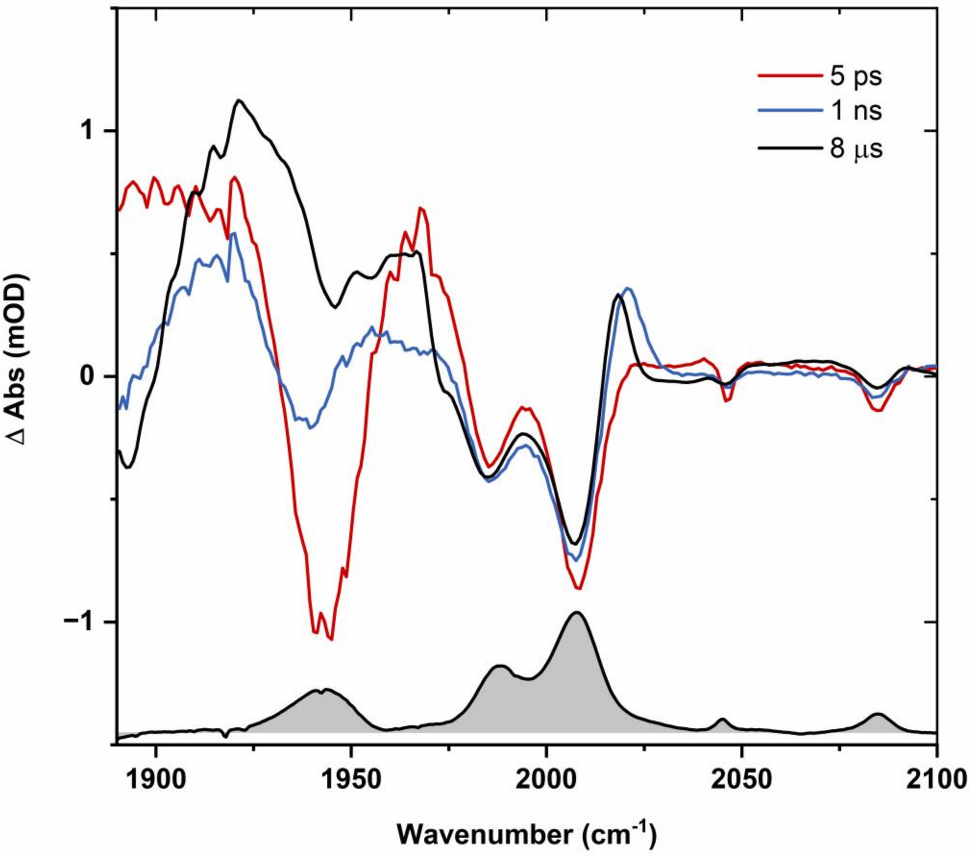

Figure S21 TRIR spectra for the reaction of **2f** in neat PhC_2H at 5 ps, 1 ns and 8 μs showing the formation of **III$_{alkyne}$**, **I** and **II** respectively. In the spectrum at 5 ps, the absorbance at *ca*. 1967 cm^{-1} is assigned to a short-lived ^{3}MLCT state. The ground state spectrum of **2f** is shown in grey at the bottom of the figure. The peak at 2045 cm^{-1} is due to a minor contamination from $[MnBn(CO)_5]$.

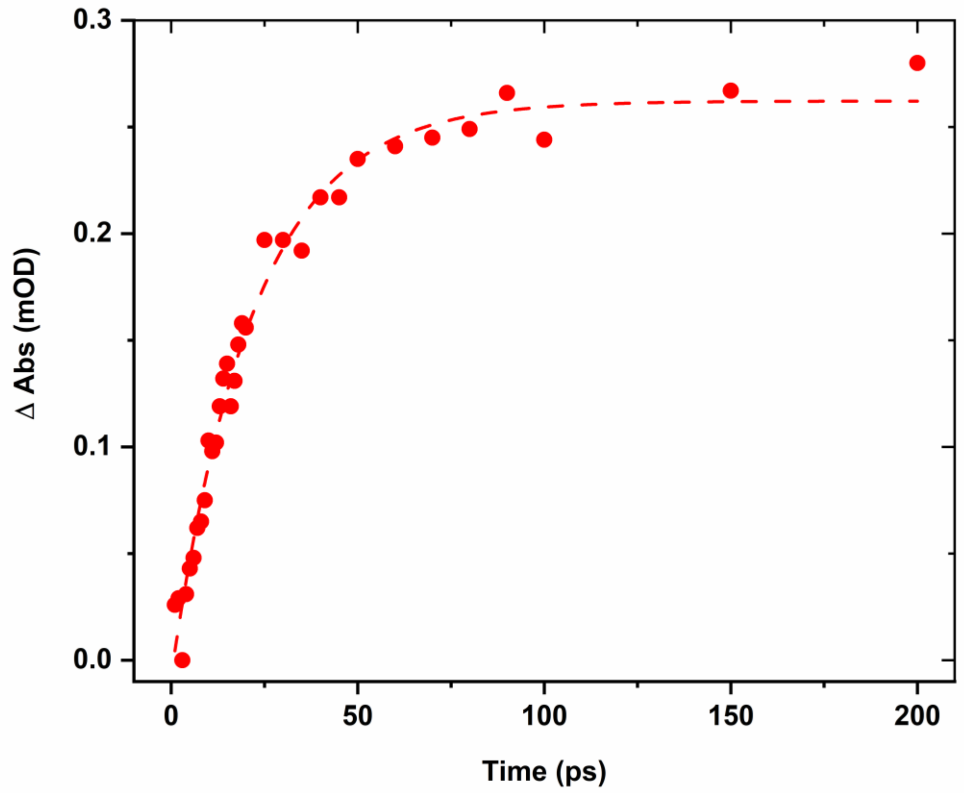

Figure S22 Kinetic profile showing the formation of **I** for a sample of **2f** in neat PhC_2H using the change in intensity of the peak at 2021 cm^{-1}. The experimental data points are shown as red circles and the fit to a monoexponential function with $k = (4.56 \pm 0.60) \times 10^{10}$ s^{-1}, R^2 = 0.980 as a red dashed line.

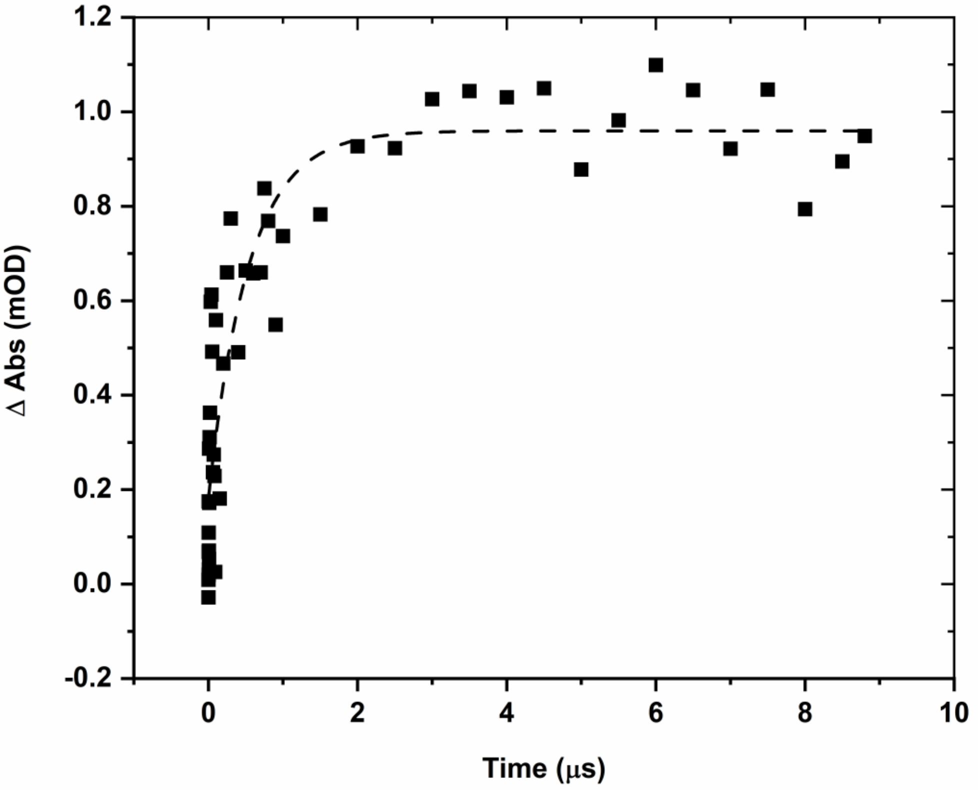

Figure S23 Kinetic profile showing the formation of **II** for a sample of **2f** in neat PhC_2H using the change in intensity of the peak at 1932 cm^{-1}. The experimental data points are shown as black squares and the fit to a monoexponential function with k = (18.5 ± 7.7)× 10^5 s^{-1}, R^2 = 0.826, as a black dashed line.

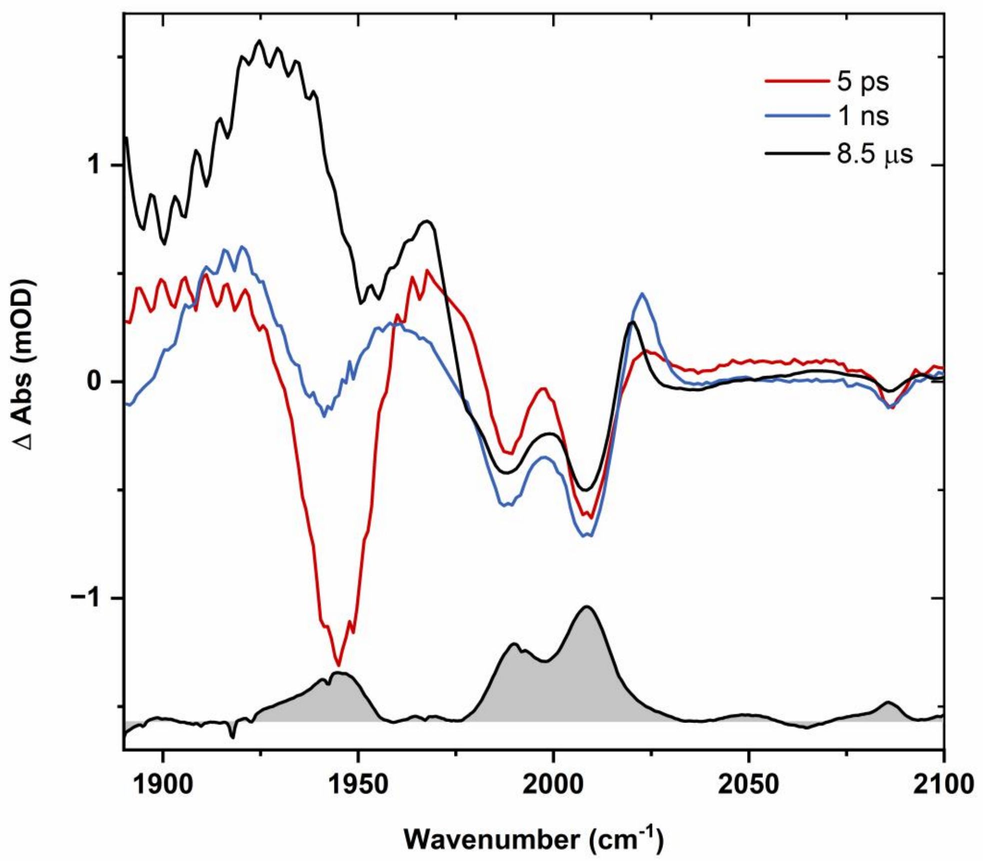

Figure S24 TRIR spectra for the reaction of **2g** in neat PhC_2H at 5 ps, 1 ns and 8.5 μs showing the formation of $\mathbf{III}_{\mathbf{alkyne}}$, **I** and **II** respectively. In the spectrum at 5 ps, the absorbance at *ca*. 1967 cm^{-1} is assigned to a short-lived ^{3}MLCT state. The ground state spectrum of **2g** is shown in grey at the bottom of the figure.

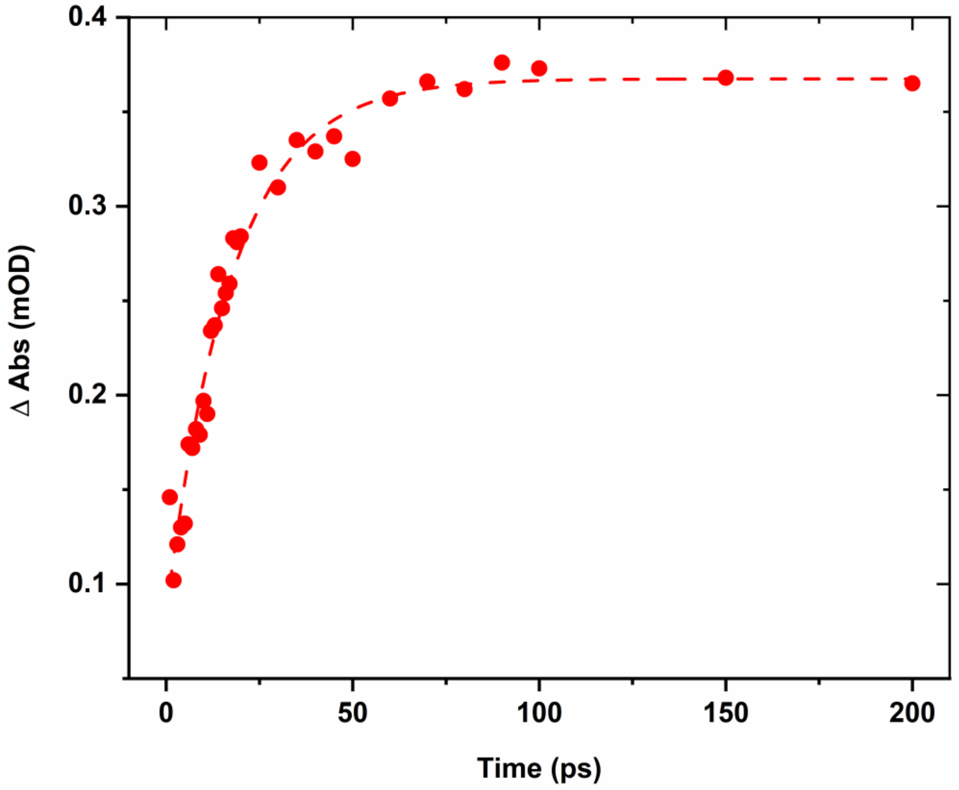

Figure S25 Kinetic profile showing the formation of **I** for a sample of **2g** in neat PhC_2H using the change in intensity of the peak at 2023 cm^{-1}. The experimental data points are shown as red circles and the fit to a monoexponential function with $k = (5.71 \pm 0.90) \times 10^{10}$ s^{-1}, $R^2 = 0.968$ as a red dashed line.

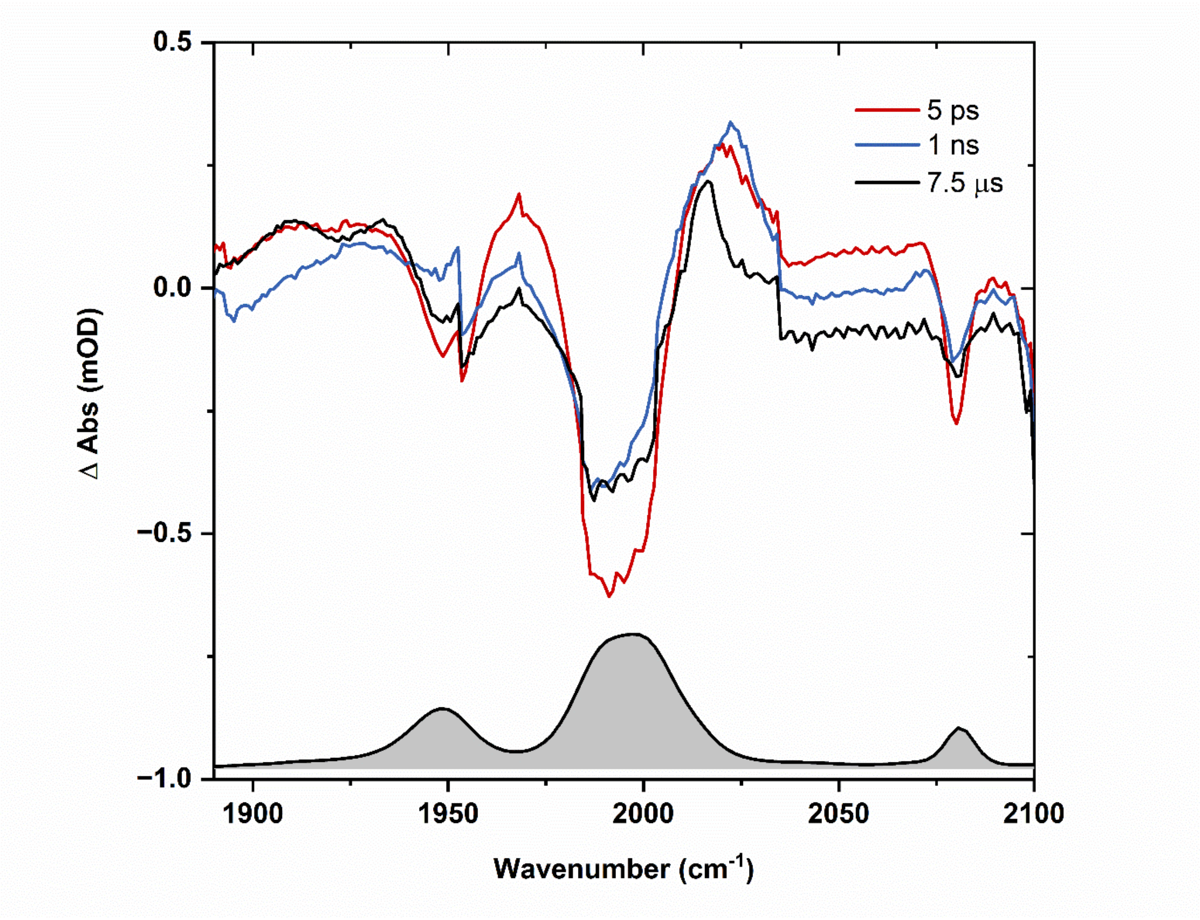

Figure S26 TRIR spectra for the reaction of **6c** in neat PhC_2H at 5 ps, 1 ns and 7.5 μs showing the formation of **III$_{alkyne}$**, **I** and **II** respectively. In the spectrum at 5 ps, the absorbance at *ca*. 1967 cm^{-1} is assigned to a short-lived ^{3}MLCT state. The ground state spectrum of **6c** is shown in grey at the bottom of the figure.

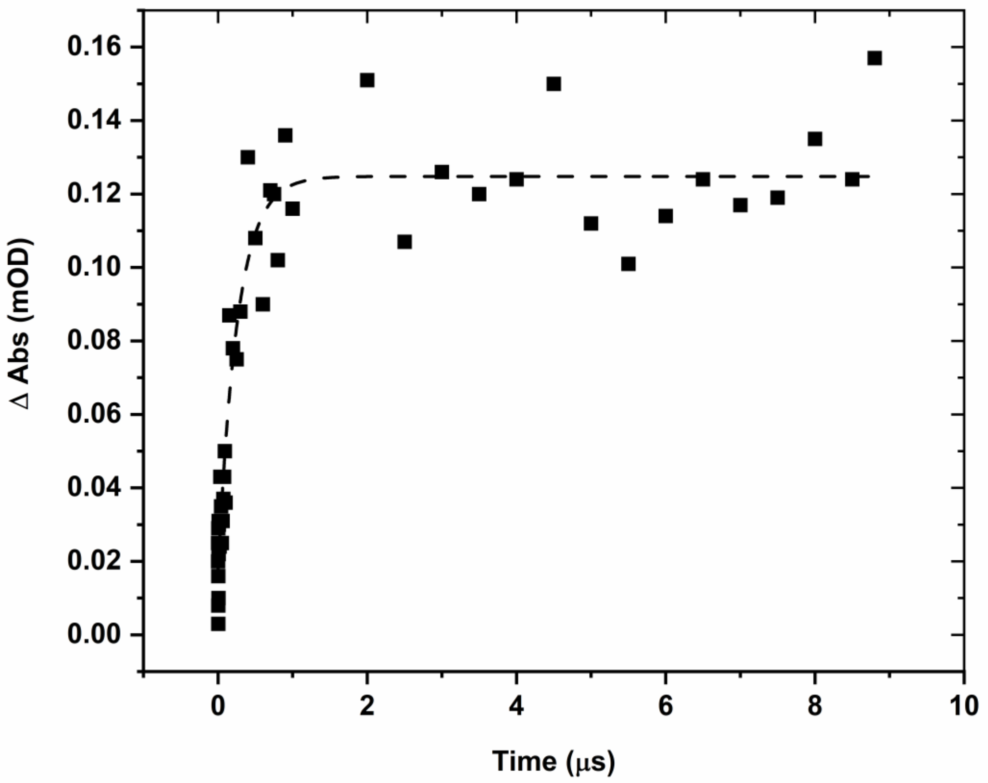

Figure S27 Kinetic profile showing the formation of **II** for a sample of **6c** in neat PhC_2H using the change in intensity of the peak at 1908 cm^{-1}. The experimental data points are shown as black squares and the fit to a monoexponential function with k = (38.6 ± 11.4)× 10^5 s^{-1}, R^2 = 0.926, as a black dashed line.

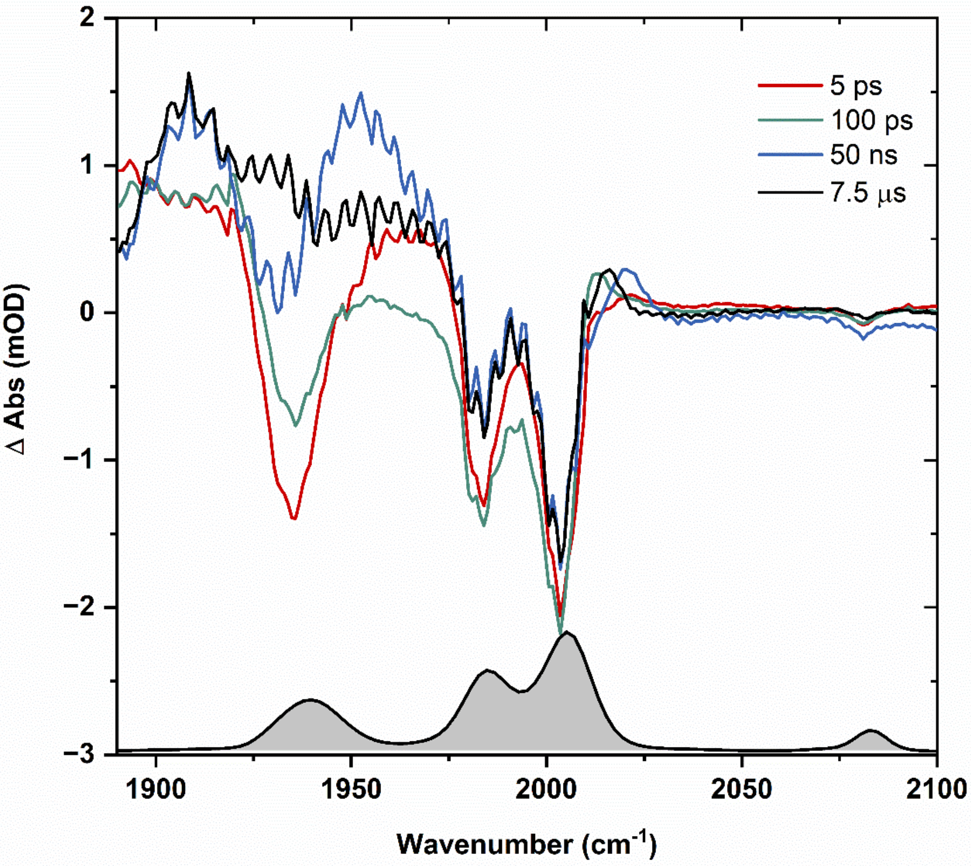

Figure S28 TRIR spectra for the reaction of **2a** in neat 4-F_3C-$C_6H_4C_2H$ at 5 ps, 100 ps, 50 ns and 7.5 μs showing the formation of **III$_{alkyne}$**, **I** and **II** respectively. In the spectrum at 5 ps, the absorbance at *ca.* 1967 cm^{-1} is assigned to a short-lived ^{3}MLCT state. The ground state spectrum of **2a** is shown in grey at the bottom of the figure.

In this instance, an additional intermediate species (green spectrum) was observed at *ca*. 100 ps, following the intimal formation of **III$_{alkyne}$** (red spectrum). This decayed over the course of 100 ns to form the characteristic peaks for **I** (blue spectrum) that then underwent a migratory insertion reaction to give **II** (black spectrum). The rate constants for the formation and loss of **I** were similar, however the quality of the data did not permit an effective fit to a sequential kinetic model. The successful fits below are to single monoexponential functions and therefore the measured rate constants do not account for the stepwise process.

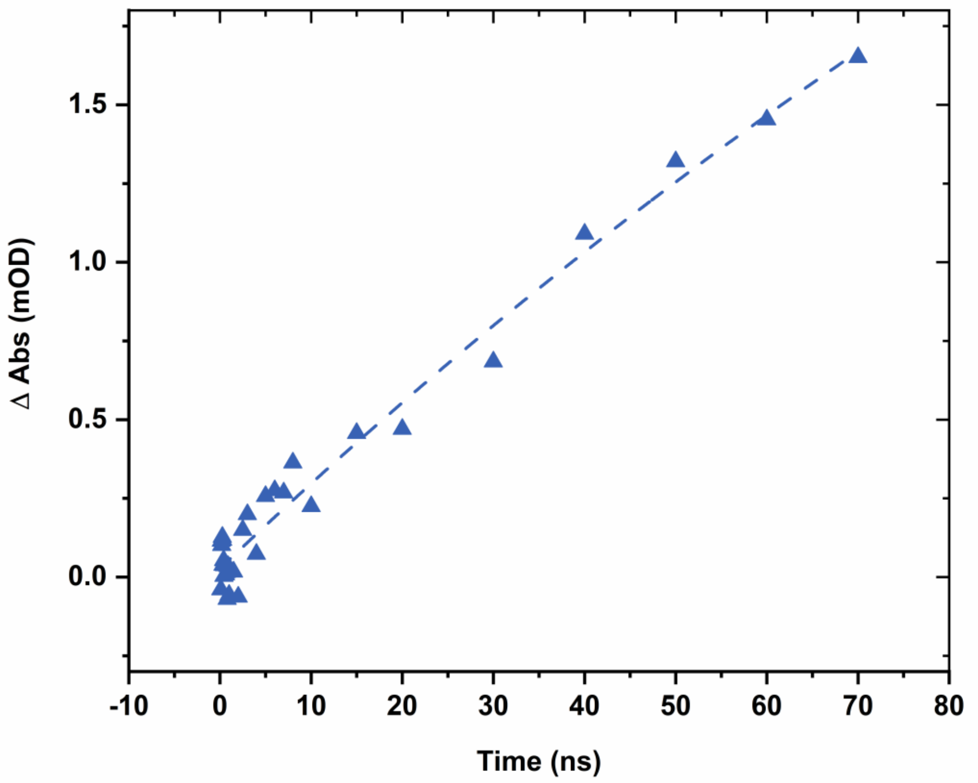

Figure S29 Kinetic profile showing the gain of **I** for a sample of **2a** in neat 4-F_3C-$C_6H_4C_2H$ using the change in intensity at 1941 cm^{-1}. The experimental data points are shown as blue triangles and the fit to a monoexponential function with k = (48.2 ± 7.9) × 10^5 s^{-1}, R^2 = 0.972, as a blue dashed line.

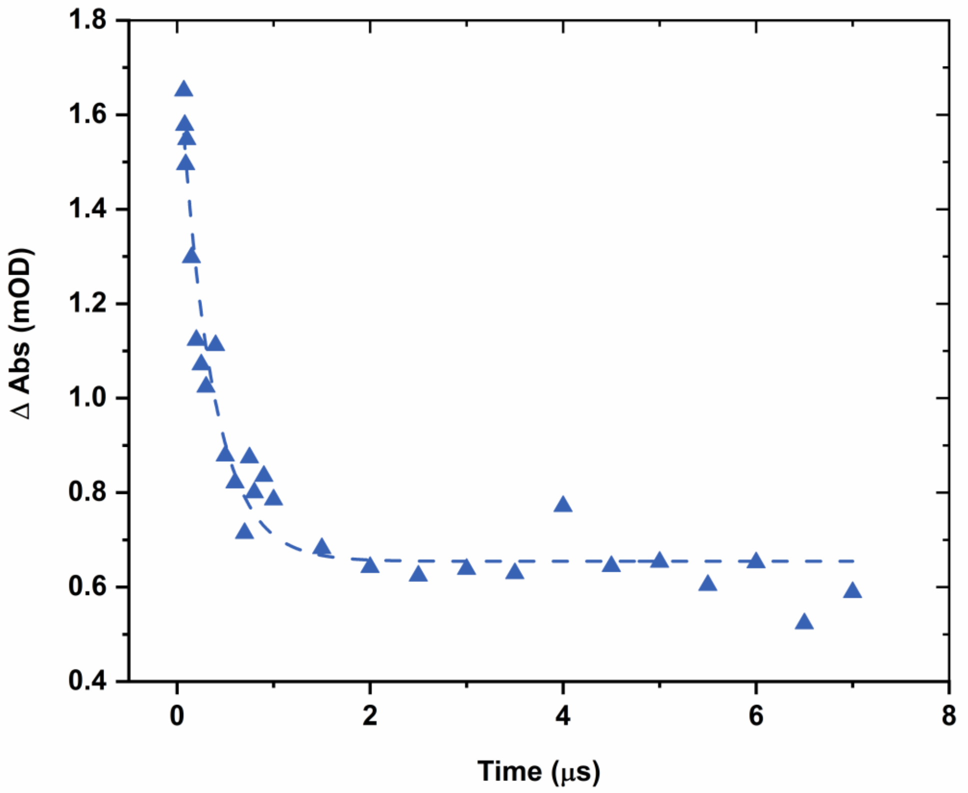

Figure S30 Kinetic profile showing the loss of **I** for a sample of **2a** in neat 4-F_3C-$C_6H_4C_2H$ using the change in intensity at 1941 cm^{-1}. The experimental data points are shown as blue triangles and the fit to a monoexponential function with k = (30.0 ± 7.4) × 10^5 s^{-1}, R^2 = 0.943, as a blue dashed line.

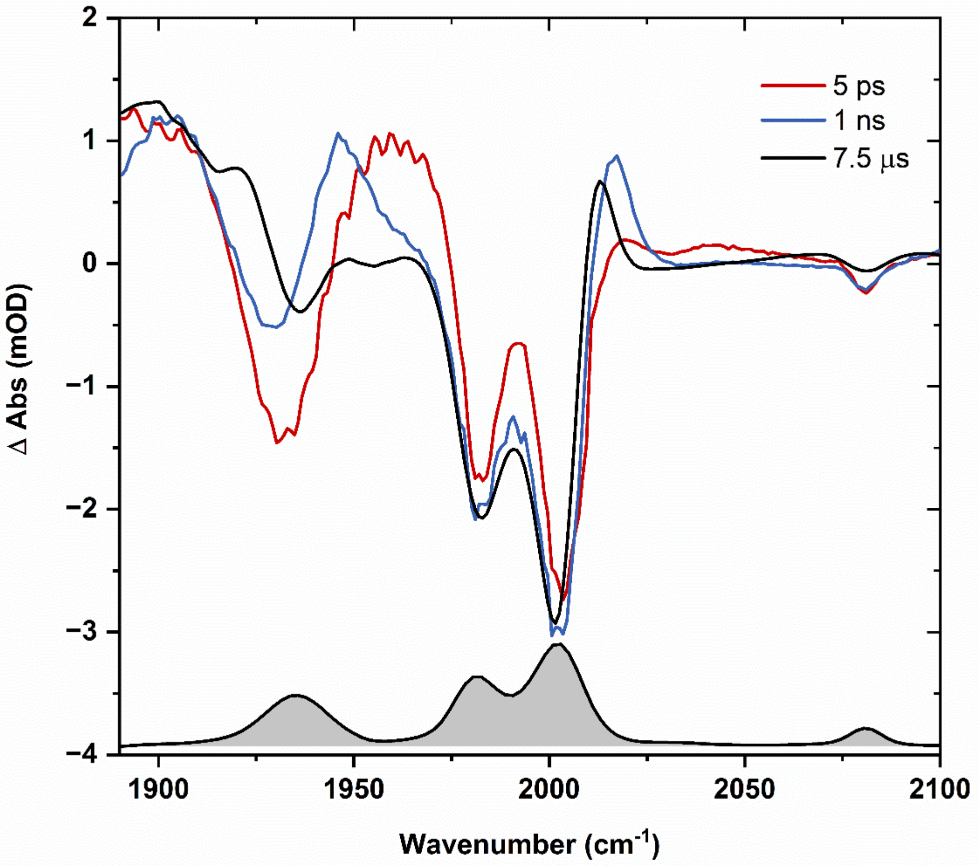

Figure S31 TRIR spectra for the reaction of **2a-^{13}C** in neat PhC_2H at 5 ps, 1 ns and 7.5 μs showing the formation of **III**$_{\mathbf{alkyne}}$, **I** and **II** respectively. In the spectrum at 5 ps, the absorbance at *ca*. 1967 cm^{-1} is assigned to a short-lived ^{3}MLCT state. The ground state spectrum of **2a-^{13}C** is shown in grey at the bottom of the figure.

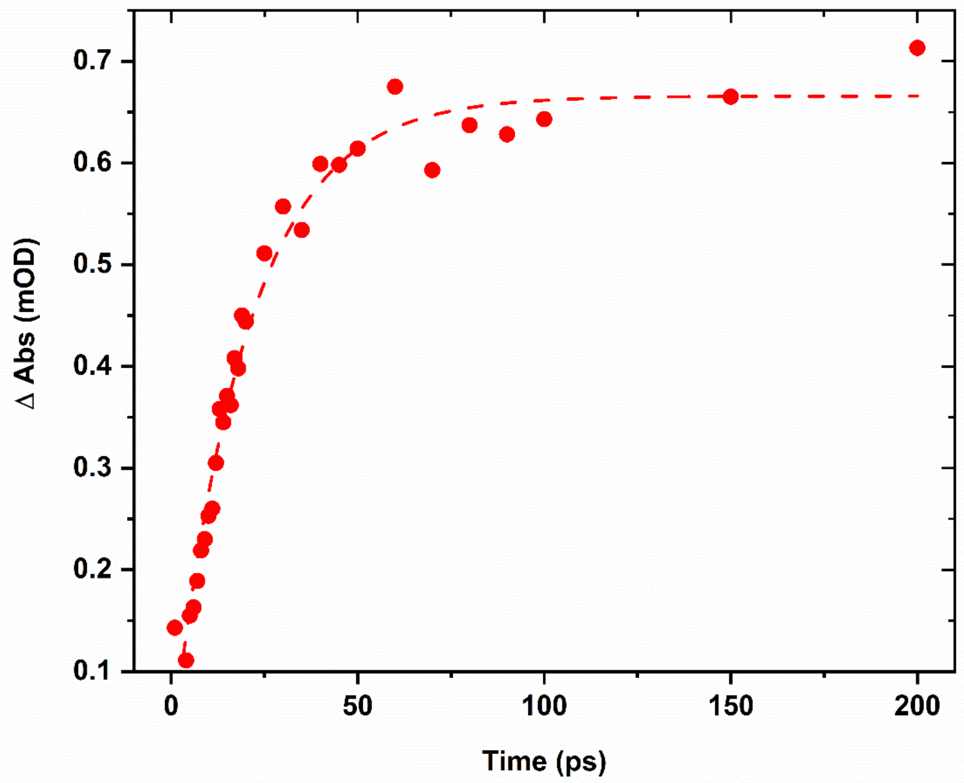

Figure S32 Kinetic profile showing the formation of **I** for a sample of **2a-^{13}C** in neat PhC_2H using the change in intensity of the peak at 2017 cm^{-1}. The experimental data points are shown as red circles and the fit to a monoexponential function with $k = (5.05 \pm 0.71) \times 10^{10}\ s^{-1}$, $R^2 = 0.977$ as a red dashed line.

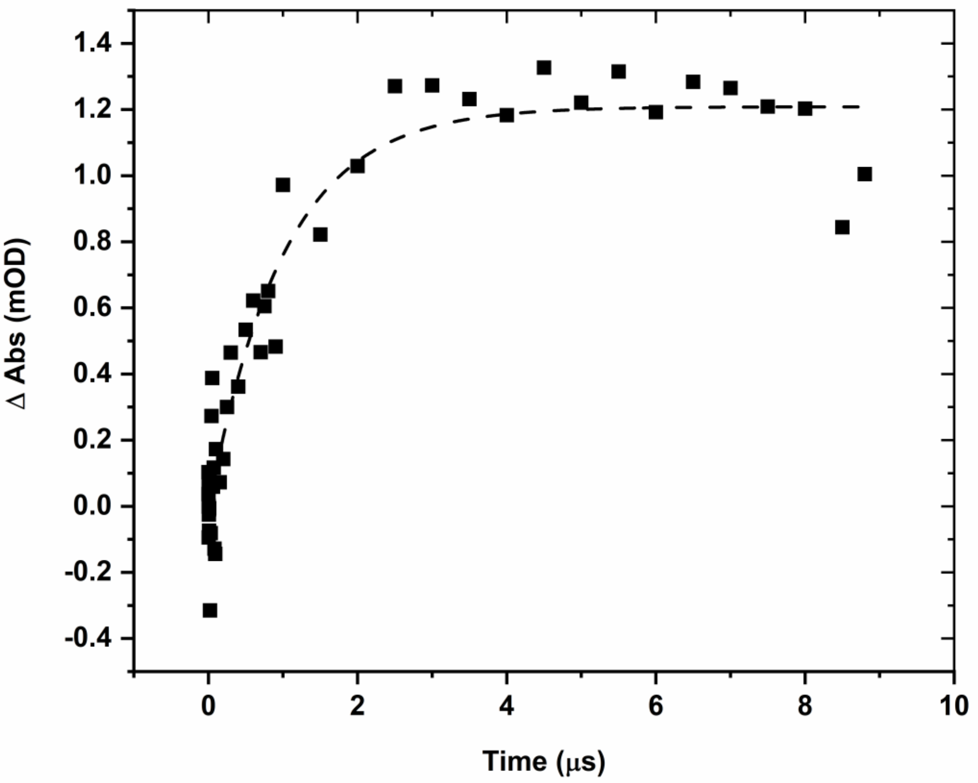

Figure S33 Kinetic profile showing the formation of **II** for a sample of **2a-^{13}C** in neat PhC_2H using the change in intensity of the peak at 1920 cm^{-1}. The experimental data points are shown as black squares and the fit to a monoexponential function with k = (9.99 ± 2.5)× 10^5 s^{-1}, R^2 = 0.926, as a black dashed line.

17 X-Ray Crystal and MicroED Structures

18 X-Ray Crystallography

All X-Ray diffraction data were collected at 110 K using an Oxford Diffraction SuperNova diffractometer with Cu-Kα radiation (λ = 1.54184 Å), using an EOS CCD camera. The crystal was cooled with an Oxford Instruments Cryojet. Diffractometer control, data collection, initial unit cell determination, frame integration and unit-cell refinement were carried out with "Crysalis".[23] Face-indexed absorption corrections were applied using spherical harmonics, implemented in SCALE3 ABSPACK scaling algorithm. OLEX2[24] was used for overall structure solution and refinement. Within OLEX2, the algorithm used for structure solution was "ShelXT dual-space".[25] Refinement was carried out by full- matrix least-squares using the SHELXL-97[25] algorithm within OLEX2.[24] All non-hydrogen atoms were refined anisotropically. Crystalmaker® software was used to visualise the structures as well as generating the figures presented herein, for clarity some figures show hydrogen atoms or solvent molecules removed.

19 MicroED

Quantifoil Cu R2/4 grids that had been assembled into autogrid cartridges were placed in individual glass vials containing micro-crystalline samples of **1b**, **2c**, and **2d**. Compound **2d** had previously been gently crushed in the vial using a micro-spatula. The vials were gently shaken to allow sample transfer and the autogrids were removed from the vials and cooled to liquid nitrogen temperature prior to transfer to the cassette and loading into the TEM.

MicroED data were collected at 80 K using a Thermo Scientific Glacios TEM operated at 200 kV and equipped with a Ceta-D camera. To obtain a low flux in nanoprobe mode the following illumination conditions were employed: gun lens 8, spot size 11. A 30 µm C2 aperture was used resulting in a parallel beam of 1.05 µm diameter and a flux of 0.07 $e^{-}Å^{-2}s^{-1}$. Data were acquired with EPU-D using the following settings: 2x binning, a rotation speed of 1.33 $°s^{-1}$ and an exposure time of 0.75 s.

All data were processed using DIALS.[28] The images recorded on Ceta-D camera show mean negative background values at high resolution which hampers background modelling so a pedestal of 64 ADU was added to every pixel value. Initially the detector distance was fixed to 958.5 mm (determined using powder diffraction from an aluminium powder calibration grid). When datasets from multiple crystals were combined (samples **1b** and **2d**) the strong reflections from each of the datasets were used to refine the detector distance and unit cell parameters of each of the datasets jointly. The optimal unit cell parameters for each of the combined datasets were then refined by fitting calculated to observed 2θ values. For both samples **1b** and **2d** five datasets from five crystals were combined to give a completeness of 92.5% to 0.83 Å resolution (**1b**) and 99.9% to 0.83 Å resolution (**2d**), respectively. For sample **2c** a single dataset from one crystal was recorded that was 93.1% complete to 0.83 Å resolution.

The structures were solved ab initio using SHELXT[25] and refined with SHELXL[29]. The electron scattering factors from Peng[30] were used in refinement. Hydrogen atoms were geometrically placed at the idealised internuclear X-H distances used in refinement of structures against neutron diffraction data with SHELXL[31] and allowed to ride on their parent atoms during refinement. For **1b** and **2c** no restraints were applied during refinement. For **2d** RIGU restraints[32] were applied to the ADPs of all non-hydrogen atoms. In all cases an extinction parameter was refined. Conversion of map values (in $Å^{-2}$) to values of electrostatic potential in $eÅ^{-1}$ used a factor of 47.87801 $Å^2V$ (International Tables for Crystallography (2006) Volume C section 4.3.1.7) followed by the conversion 1V = 0.069446154 $eÅ^{-1}$ (calculated with recommended values from CODATA 2018).

Crystallographic Data for Compound 1a (ijsf22003)

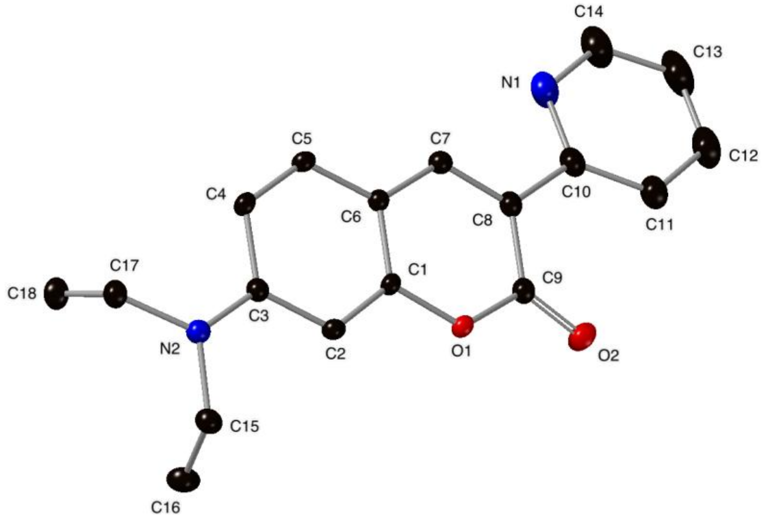

Figure S34 XRD Crystal structure of coumarin **1a**, thermal ellipsoids shown at 50% probability and hydrogen atoms removed for clarity.

Identification code	ijsf22003
Empirical formula	$C_{18}H_{18}N_2O_2$
Formula weight	294.34
Temperature/K	110.00(10)
Crystal system	triclinic
Space group	P-1
a/Å	9.2796(4)
b/Å	12.6862(4)
c/Å	14.3292(5)
α/°	103.195(3)
β/°	108.738(4)
γ/°	98.088(3)
Volume/Å^3	1512.81(10)
Z	4
ρ_{calc}g/cm^3	1.292
μ/mm^{-1}	0.683
F(000)	624.0
Crystal size/mm^3	0.35 × 0.31 × 0.24
Radiation	Cu Kα (λ = 1.54184)
2Θ range for data collection/°	6.81 to 142.134
Index ranges	-11 ≤ h ≤ 11, -12 ≤ k ≤ 15, -15 ≤ l ≤ 17
Reflections collected	10280
Independent reflections	5706 [R_{int} = 0.0137, R_{sigma} = 0.0213]
Data/restraints/parameters	5706/0/401
Goodness-of-fit on F^2	1.033
Final R indexes [I>=2σ (I)]	R_1 = 0.0353, wR_2 = 0.0937

F inal R indexes [all data]	$R_1 = 0.0398$, $wR_2 = 0.0974$
Largest diff. peak/hole / e Å^{-3}	0.21/-0.25

Crystallographic Data for Compound 1b (TJB-2-050)

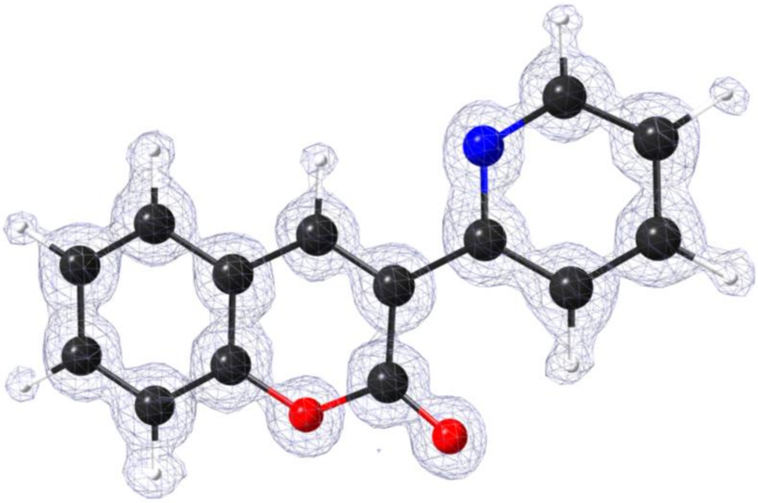

Figure S35 MicroED Refined structure of coumarin **1b**, ball and stick model, and Fourier map shown in grey, (Completeness = 92.5%, resolution = 0.83 Å).

Identification code	TJB-2-050
Empirical formula	$C_{14}H_9NO_2$
Formula weight	223.22
Temperature/K	80(2)
Crystal system	orthorhombic
Space group	Pbca
a/Å	7.0778(2)
b/Å	13.7315(8)
c/Å	21.3139(11)
α/°	90
β/°	90
γ/°	90
Volume/Å^3	2071.47(17)
Z	8
ρ_{calc} g/cm^3	1.432
μ/mm^{-1}	0.000
F(000)	368.0
Crystal size/mm^3	nano crystals
Radiation	electron (λ = 0.02508)
2Θ range for data collection/°	0.22 to 1.866
Index ranges	$-9 \le h \le 9$, $-17 \le k \le 17$, $-27 \le l \le 27$
Reflections collected	25596
Independent reflections	2219 [$R_{int} = 0.1939$, $R_{sigma} = 0.0898$]

Data/restraints/parameters	2219/0/155
Goodness-of-fit on F^2	1.952
Final R indexes [I>=2σ (I)]	R_1 = 0.1338, wR_2 = 0.3201
Final R indexes [all data]	R_1 = 0.1601, wR_2 = 0.3268
Largest diff. peak/hole / eÅ^{-1}	0.54/-0.65

Single crystal XRD data has been previously reported for **1b**.[4b]

Crystallographic Data for Compound 2b (ijsf21018)

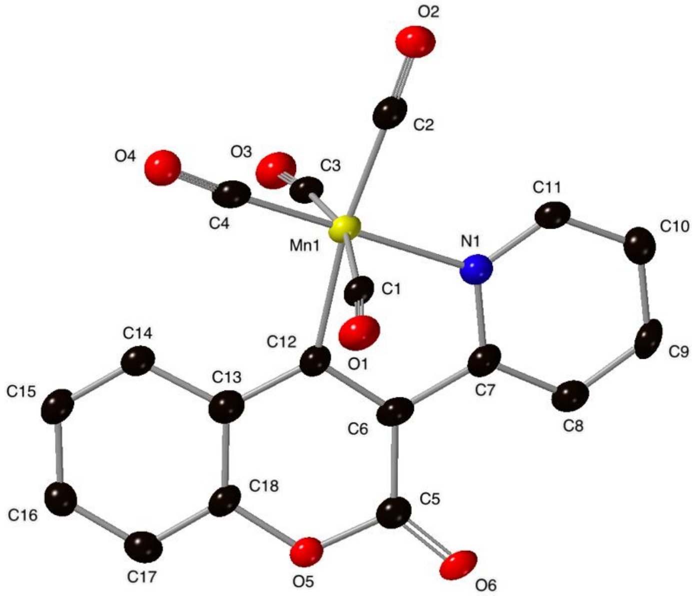

Figure S36 XRD Crystal structure of compound **2b**, thermal ellipsoids shown at 50% probability and hydrogen atoms not shown for clarity.

Crystallographic data for compound **2b** is previously reported by our group and is available.[4a]

Crystallographic Data for Compound 2c (TJB-2-072)

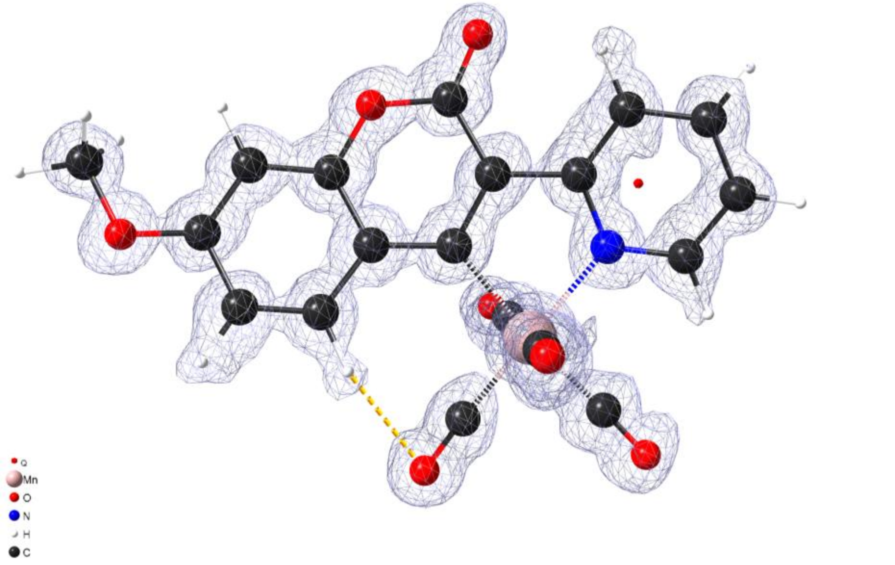

Figure S37 MicroED Refined structure of coumarin **2c**, ball and stick model, and Fourier map shown in grey, (Completeness = 93.1%, resolution = 0.83 Å).

Identification code	TJB-2-072
Empirical formula	$C_{19}H_{10}MnNO_7$
Formula weight	419.22
Temperature/K	80(2)
Crystal system	orthorhombic
Space group	Pbca
a/Å	17.1134(9)
b/Å	7.2889(4)
c/Å	27.560(2)
α/°	90
β/°	90
γ/°	90
Volume/Å^3	3437.8(4)
Z	8
ρ_{calc} g/cm^3	1.620
μ/mm^{-1}	0.000
F(000)	612.0
Crystal size/mm^3	nano crystals
Radiation	electron (λ = 0.02508)
2Θ range for data collection/°	0.134 to 1.732
Index ranges	-20 ≤ h ≤ 20, -8 ≤ k ≤ 8, -31 ≤ l ≤ 32
Reflections collected	12732
Independent reflections	3012 [R_{int} = 0.1628, R_{sigma} = 0.1324]
Data/restraints/parameters	3012/0/ 255
Goodness-of-fit on F^2	1.640

Final R indexes [I>=2σ (I)]	R_1 = 0.1441, wR_2 = 0.3190
Final R indexes [all data]	R_1 = 0.1877, wR_2 = 0.3323
Largest diff. peak/hole / eÅ^{-1}	0.65/-0.57

Crystallographic Data for Compound 2d (TJB-3-068)

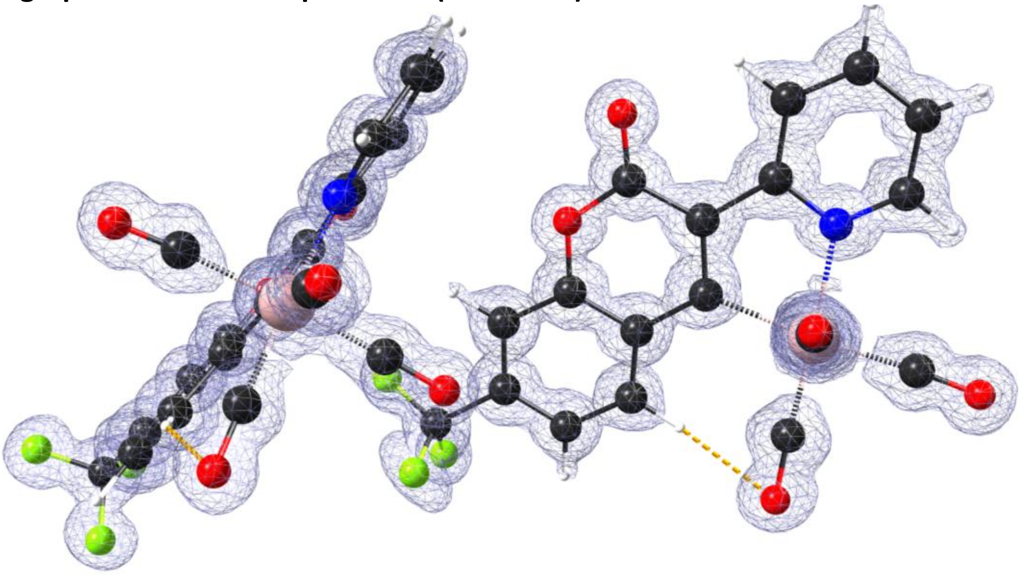

Figure S38 MicroED Refined structure of coumarin **2d**, ball and stick model, and Fourier map shown in grey, (Completeness = 99.9%, resolution = 0.83 Å).

Identification code	TJB-3-068
Empirical formula	$C_{19}H_7F_3MnNO_6$
Formula weight	457.20
Temperature/K	80(2)
Crystal system	triclinic
Space group	P -1
a/Å	11.7401(10)
b/Å	11.9216(14)
c/Å	14.2162(9)
α/°	103.081(8)
β/°	112.798(6)
γ/°	91.078(9)
Volume/Å^3	1774.1(3)
Z	4
ρ_{calc} g/cm^3	1.712
μ/mm^{-1}	0.000
F(000)	313.0
Crystal size/mm^3	nano crystals
Radiation	electron (λ = 0.02508)
2Θ range for data collection/°	0.114 to 1.866

Index ranges	$-15 \le h \le 15$, $-15 \le k \le 15$, $-18 \le l \le 18$
Reflections collected	30719
Independent reflections	7934 [R_{int} = 0.1646, R_{sigma} = 0.1080]
Data/restraints/parameters	7934/540/542
Goodness-of-fit on F^2	2.197
Final R indexes [I>=2σ (I)]	R_1 = 0.1749, wR_2 = 0.3904
Final R indexes [all data]	R_1 = 0.2129, wR_2 = 0.3987
Largest diff. peak/hole / eÅ^{-1}	0.96/-0.81

Crystallographic Data for Compound 5a (ijsf24001)

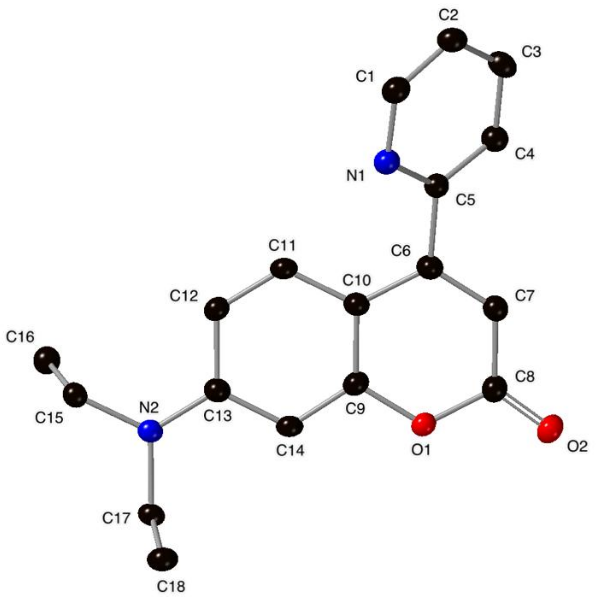

Figure S39 XRD Crystal structure of **5a** is shown as thermal ellipsoids 50% hydrogens atoms omitted for clarity.

Identification code	ijsf24001
Empirical formula	$C_{18}H_{18}N_2O_2$
Formula weight	294.34
Temperature/K	110.00(10)
Crystal system	triclinic
Space group	P-1
a/Å	9.2762(3)
b/Å	9.5433(4)
c/Å	9.6551(3)
α/°	75.508(3)
β/°	63.587(4)
γ/°	88.620(3)
Volume/Å^3	737.38(5)
Z	2
ρ_{calc}g/cm^3	1.326
μ/mm^{-1}	0.701
F(000)	312.0
Crystal size/mm^3	0.2 × 0.13 × 0.05
Radiation	Cu Kα (λ = 1.54184)
2Θ range for data collection/°	9.622 to 134.142
Index ranges	-11 ≤ h ≤ 10, -11 ≤ k ≤ 11, -11 ≤ l ≤ 11
Reflections collected	7002
Independent reflections	2626 [R_{int} = 0.0233, R_{sigma} = 0.0270]
Data/restraints/parameters	2626/0/201
Goodness-of-fit on F^2	1.048

Final R indexes [I>=2σ (I)]	R_1 = 0.0336, wR_2 = 0.0925
Final R indexes [all data]	R_1 = 0.0366, wR_2 = 0.0949
Largest diff. peak/hole / e $Å^{-3}$	0.21/-0.20

Crystallographic Data for Compound 6c (ijsf22040)

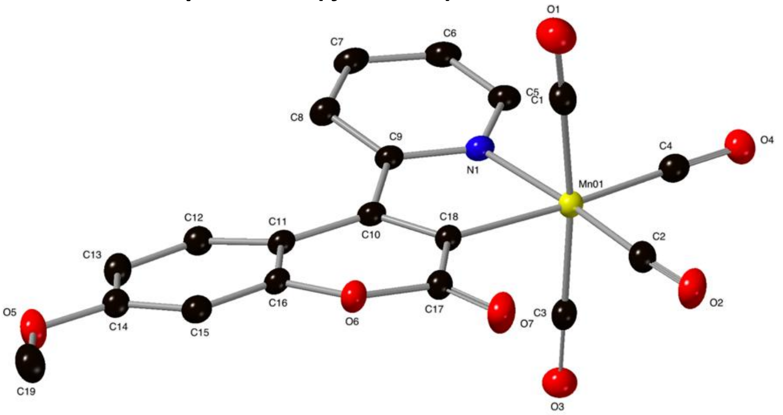

Figure S40 XRD Crystal structure of **6c** is shown as thermal ellipsoids 50% hydrogens atoms omitted for clarity.

Identification code	ijsf22040
Empirical formula	$C_{19}H_{10}MnNO_7$
Formula weight	419.22
Temperature/K	110.00(10)
Crystal system	monoclinic
Space group	$P2_1/c$
a/Å	13.3177(3)
b/Å	10.23185(17)
c/Å	14.0274(3)
α/°	90
β/°	117.477(3)
γ/°	90
Volume/Å^3	1695.82(8)
Z	4
ρ_{calc}g/cm^3	1.642
μ/mm^{-1}	6.750
F(000)	848.0
Crystal size/mm^3	0.219 × 0.13 × 0.02
Radiation	Cu Kα (λ = 1.54184)
2Θ range for data collection/°	7.482 to 134.04
Index ranges	-15 ≤ h ≤ 15, -12 ≤ k ≤ 12, -16 ≤ l ≤ 12
Reflections collected	10339
Independent reflections	3022 [R_{int} = 0.0365, R_{sigma} = 0.0367]
Data/restraints/parameters	3022/0/293
Goodness-of-fit on F^2	1.056
Final R indexes [I>=2σ (I)]	R_1 = 0.0271, wR_2 = 0.0668
Final R indexes [all data]	R_1 = 0.0308, wR_2 = 0.0687
Largest diff. peak/hole / e Å^{-3}	0.24/-0.26

Crystallographic Data for Compound 7c (ijsf22041)

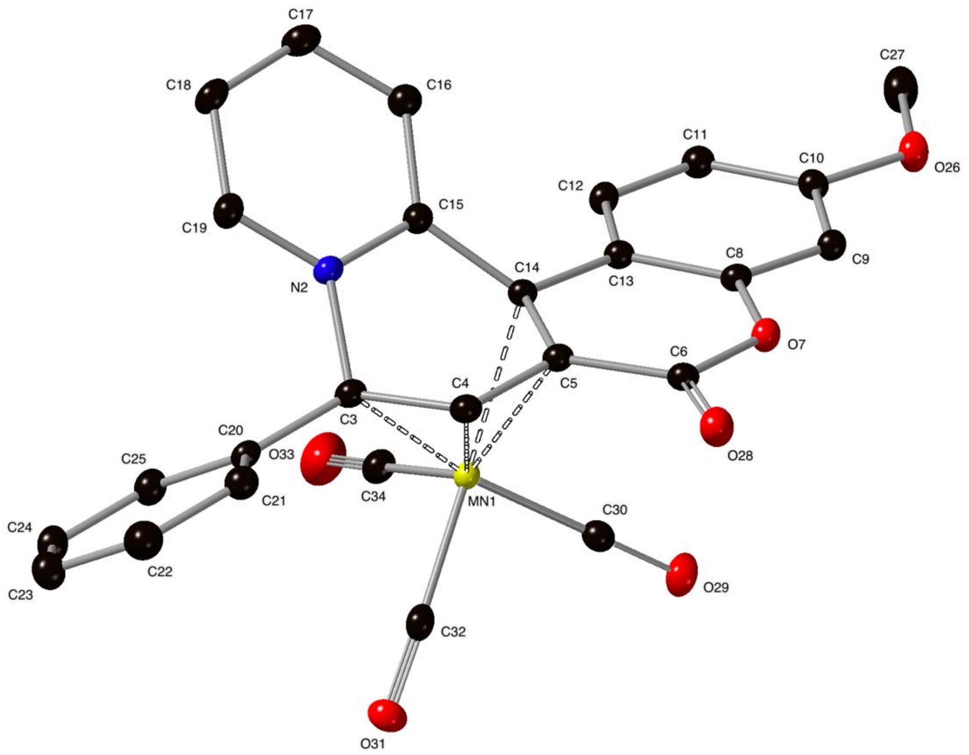

Figure S41 XRD crystal structure of complex **7c**, thermal ellipsoids shown at 50% probability, hydrogen atoms omitted for clarity.

Identification code	ijsf22041
Empirical formula	$C_{26}H_{16}NO_6Mn$
Formula weight	493.34
Temperature/K	110.05(10)
Crystal system	triclinic
Sp 1 ace group	P-1
a/Å	7.82899(14)
b/Å	10.54322(16)
c/Å	13.58845(18)
α/°	74.1326(13)
β/°	88.1497(13)
γ/°	75.3292(15)
Volume/Å^3	1042.89(3)
Z	2
ρ_{calc}g/cm^3	1.571
μ/mm^{-1}	5.551
F(000)	504.0
Crystal size/mm^3	0.277 × 0.228 × 0.172
Radiation	Cu Kα (λ = 1.54184)
2Θ range for data collection/°	9.016 to 134.142
Index ranges	-9 ≤ h ≤ 8, -12 ≤ k ≤ 12, -16 ≤ l ≤ 15

Reflections collected	10402
Independent reflections	3736 [R_{int} = 0.0279, R_{sigma} = 0.0302]
Data/restraints/parameters	3736/0/372
Goodness-of-fit on F^2	1.066
Final R indexes [I>=2σ (I)]	R_1 = 0.0250, wR_2 = 0.0631
Final R indexes [all data]	R_1 = 0.0261, wR_2 = 0.0636
Largest diff. peak/hole / e Å^{-3}	0.24/-0.26

Table S6 Tabulated bond lengths between carbon positions on coumarin derivatives 1a, 1b, 2b, 2c, 2d, 5a and 6c.[a]

Compound	CCDC Nº	C5–C6 / Å	C8–C9/ Å	C6–C7/ Å	C7–C8/ Å
**1a**	2505686	1.3695(33)	1.3803(34)	1.4278(51)	1.4135(23)
**1b**	2533270	1.3976(221)	1.4166(225)	1.4160(196)	1.3836(218)
**2b**[4]	2204319	1.3668(82)	1.3794(78)	1.3745(69)	1.3663(67)
**2c**	2533271	1.3515(174)	1.3988(174)	1.4134(236)	1.3942(183)
**2d**	2533272	1.4182(168)	1.4090(174)	1.3917(205)	1.3892(296)
**5a**	2505687	1.3706(26)	1.3791(27)	1.4261(30)	1.4061(32)
**6c**	2505688	1.3770(32)	1.3862(31)	1.4001(30)	1.3852(29)

[a] The sc-XRD data for complex **2b** has been previously reported.[4a] The data is included here to allow for comparison with related compounds.

20 NMR Spectra

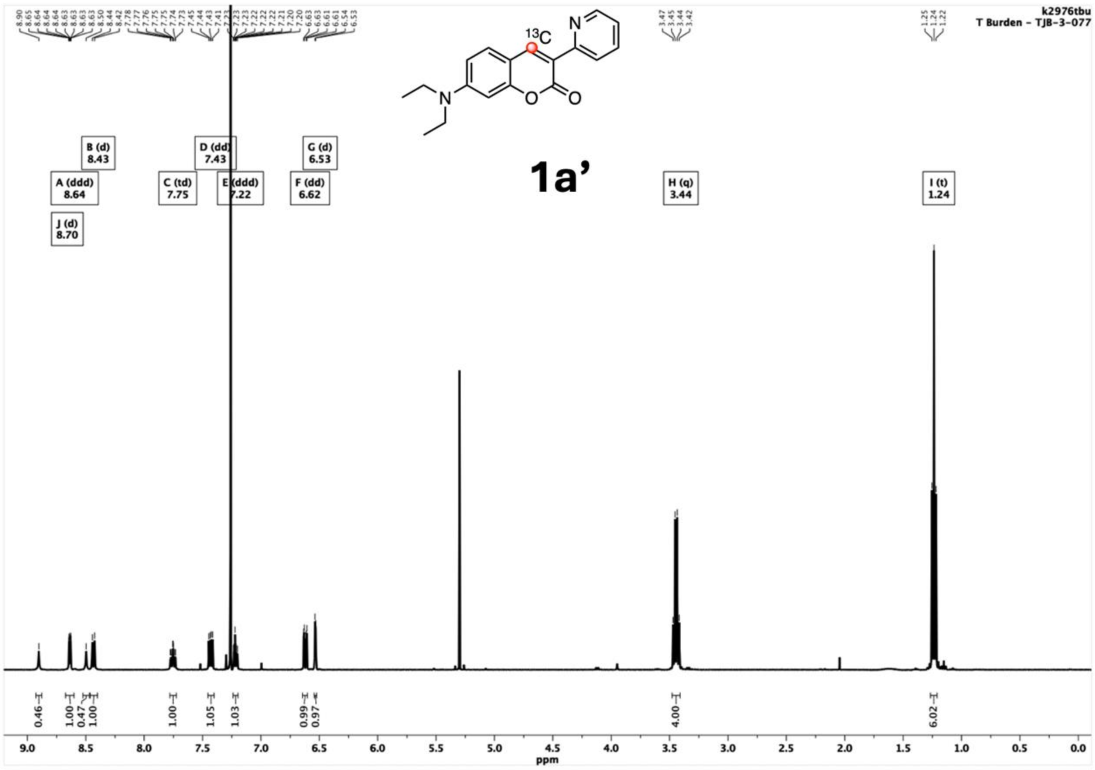

Figure S42 ^{1}H NMR Spectrum (400 MHz, $CDCl_3$) of **1a'**.

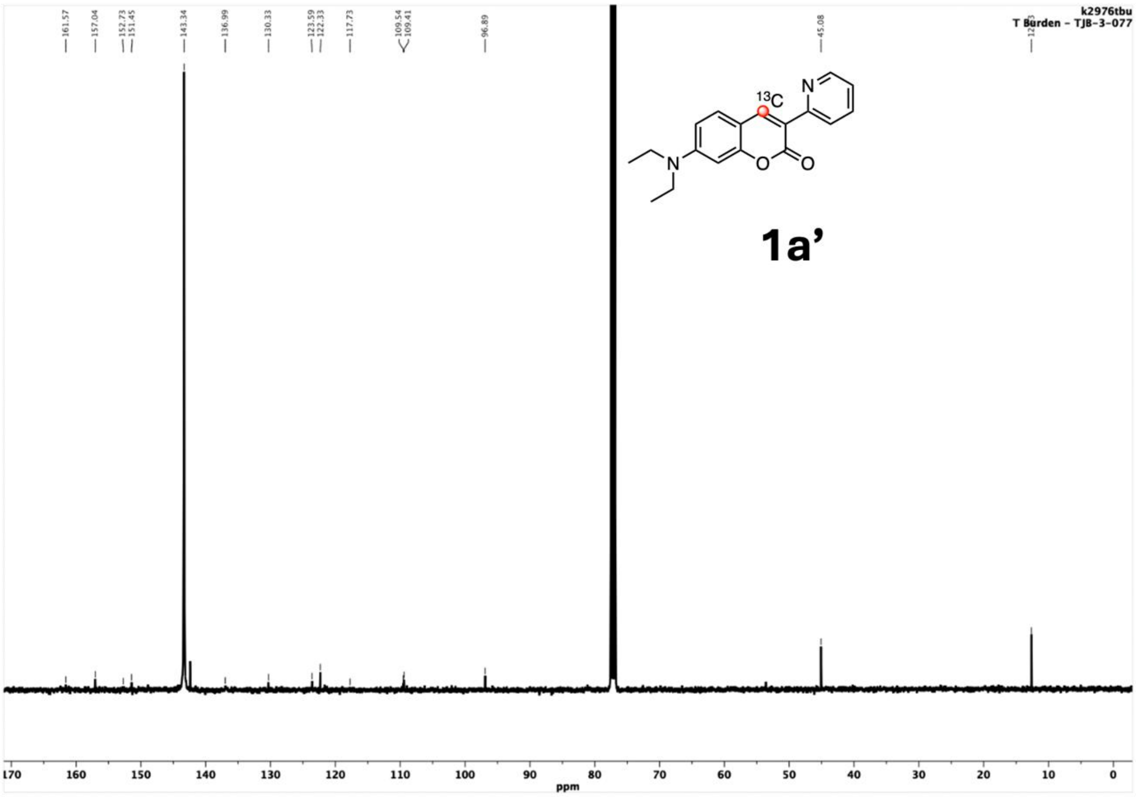

Figure S43 $^{13}C\{^{1}H\}$ NMR Spectrum (101 MHz, $CDCl_3$) of **1a'**.

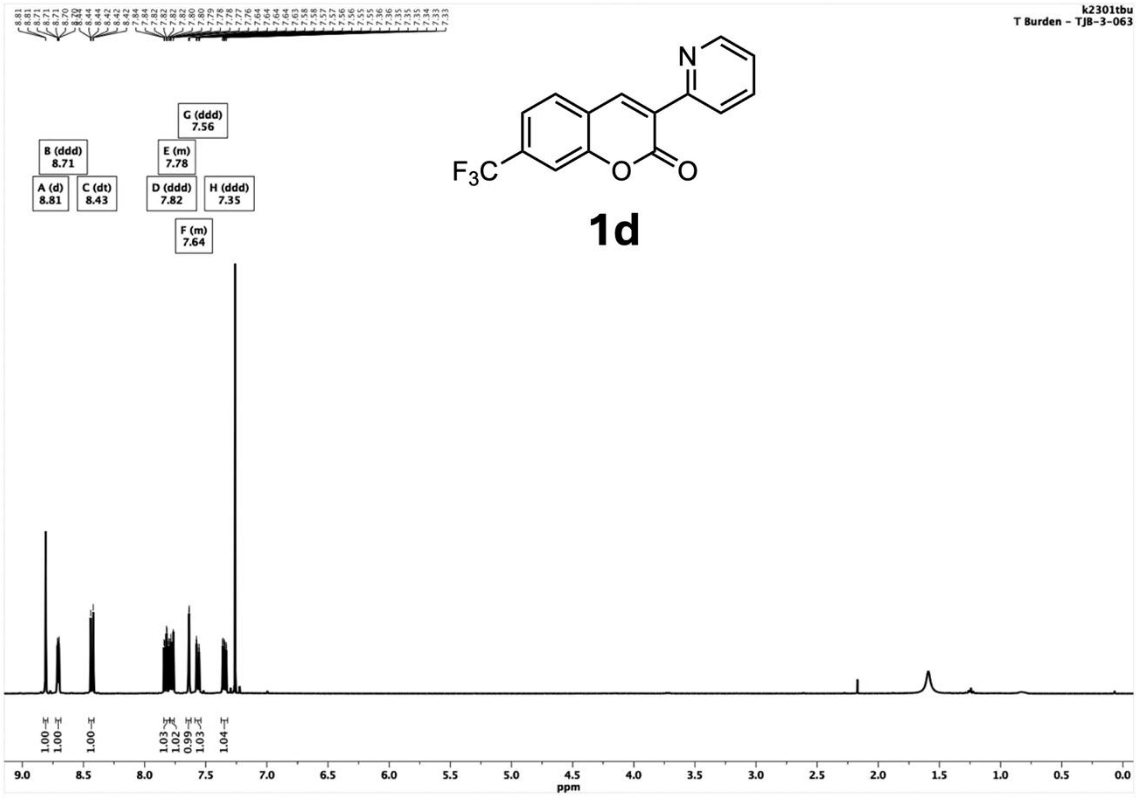

Figure S44 ^{1}H NMR Spectrum (400 MHz, $CDCl_3$) of **1d**.

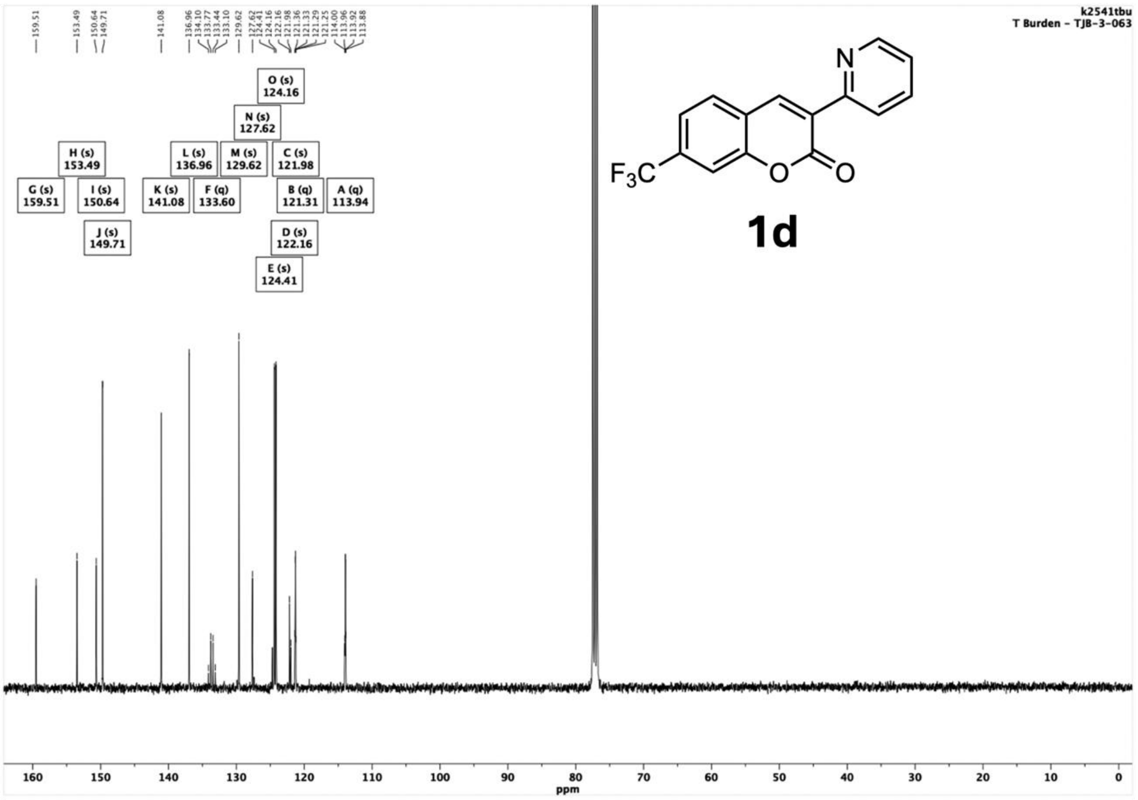

Figure S45 $^{13}C\{^{1}H\}$ NMR Spectrum (101 MHz, $CDCl_3$) of **1d**.

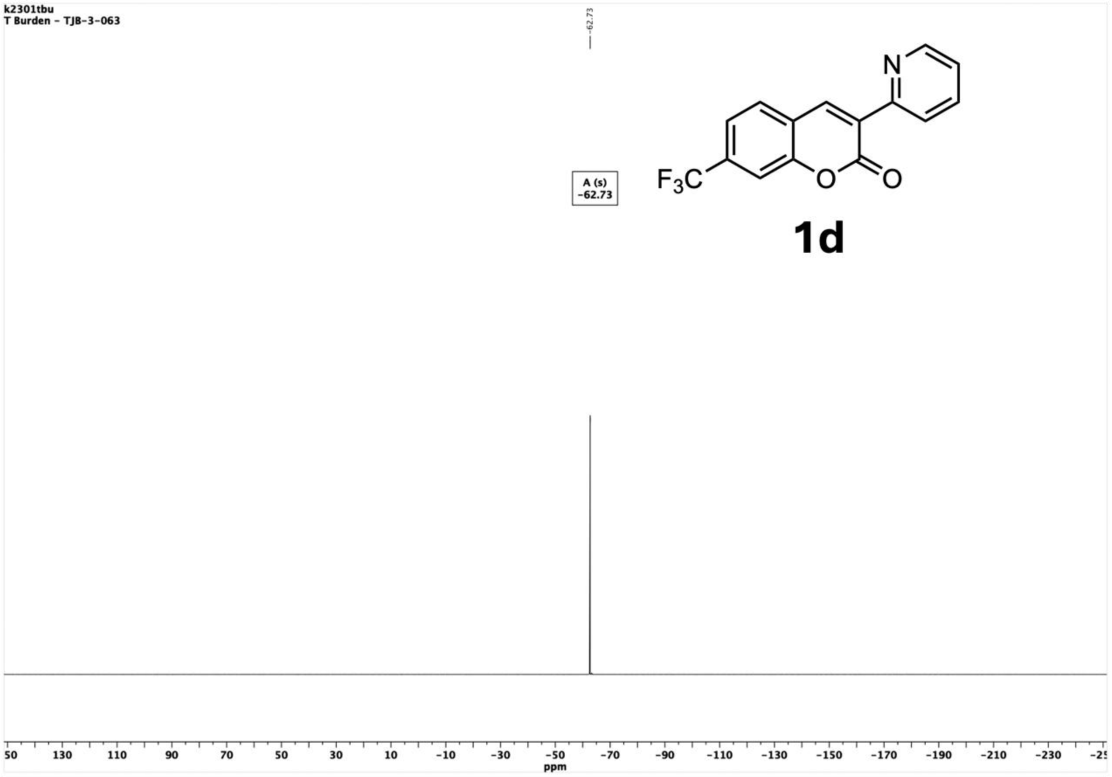

Figure S46 ^{19}F NMR Spectrum (376 MHz, $CDCl_3$) of **1d**.

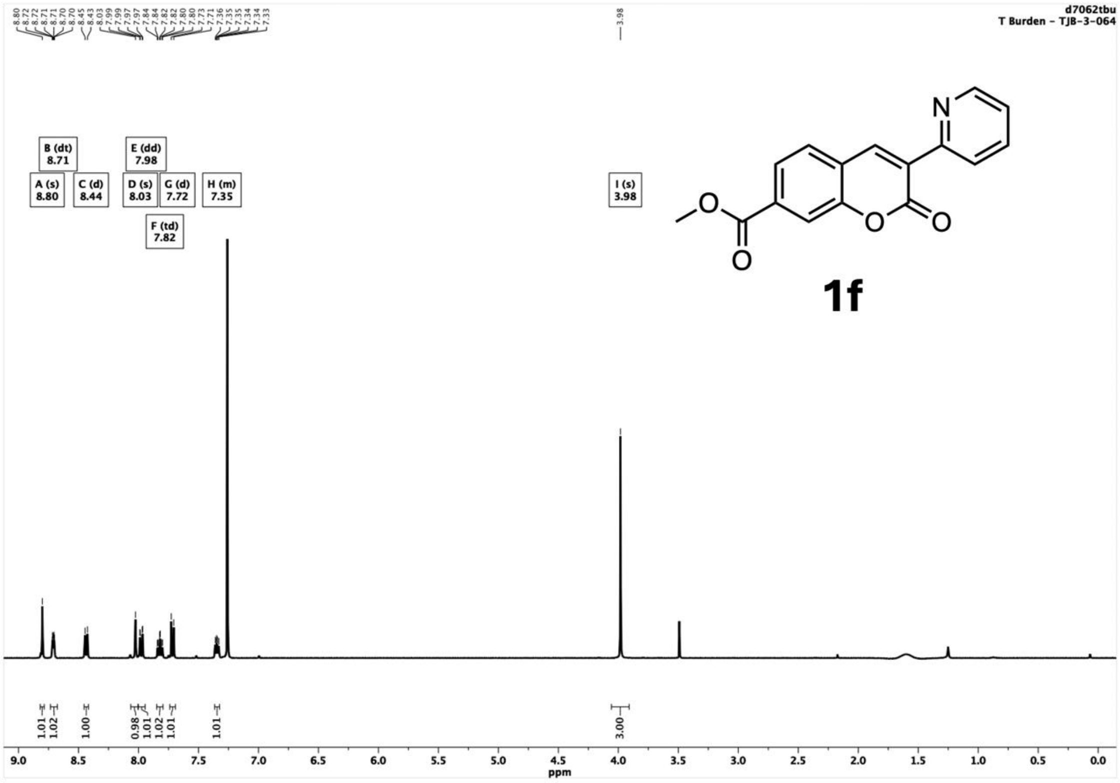

Figure S47 ^{1}H NMR Spectrum (400 MHz, $CDCl_3$) of **1f**.

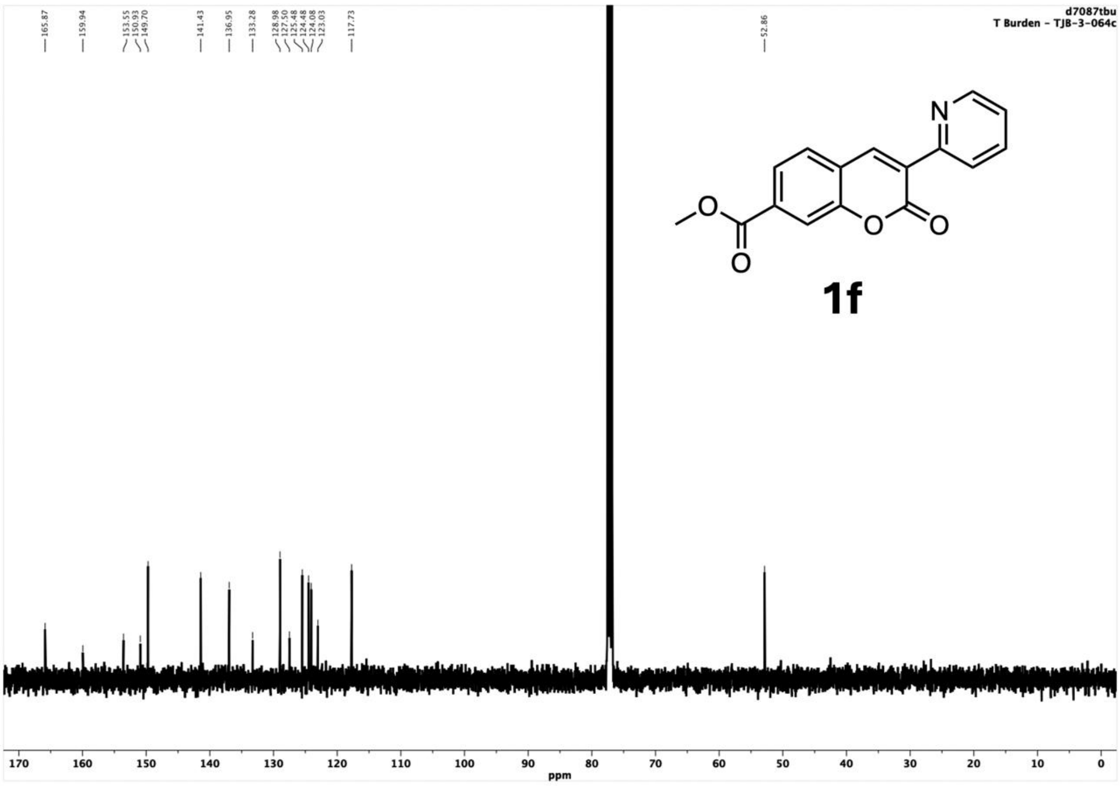

Figure S48 $^{13}C\{^{1}H\}$ NMR Spectrum (101 MHz, $CDCl_3$) of **1f**.

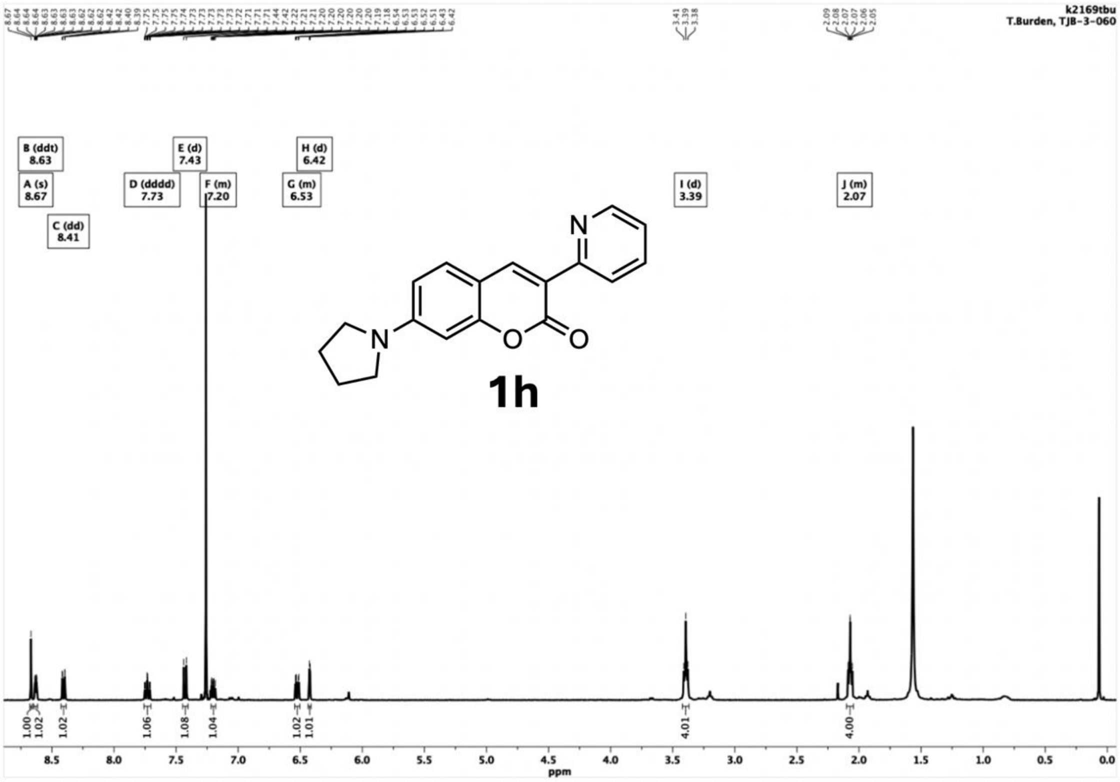

Figure S49 ^{1}H NMR Spectrum (400 MHz, $CDCl_3$) of **1h**.

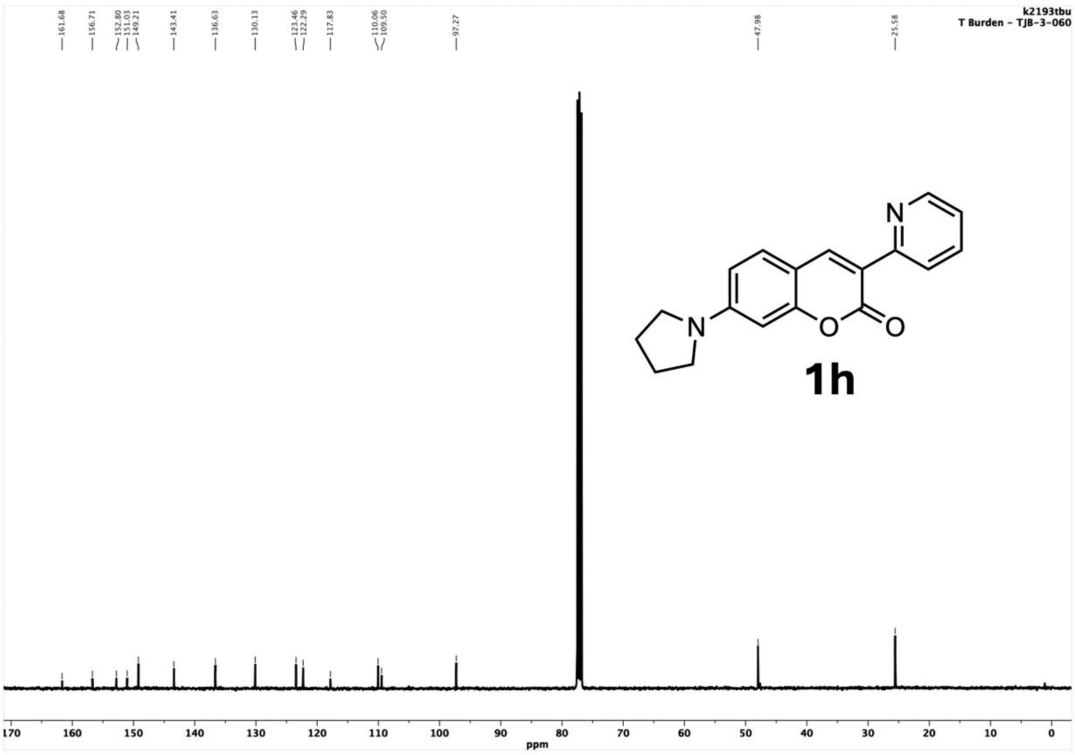

Figure S50 $^{13}C\{^{1}H\}$ NMR Spectrum (101 MHz, $CDCl_3$) of **1h**.

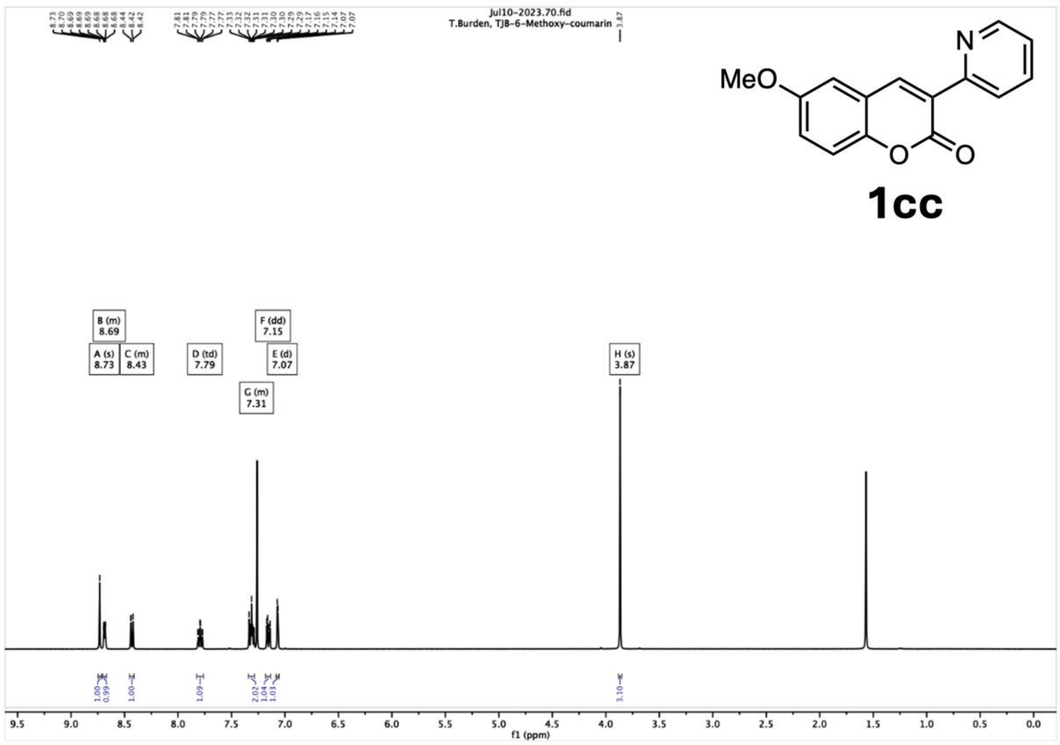

Figure S51 ^{1}H NMR Spectrum (400 MHz, $CDCl_3$) of **1cc**.

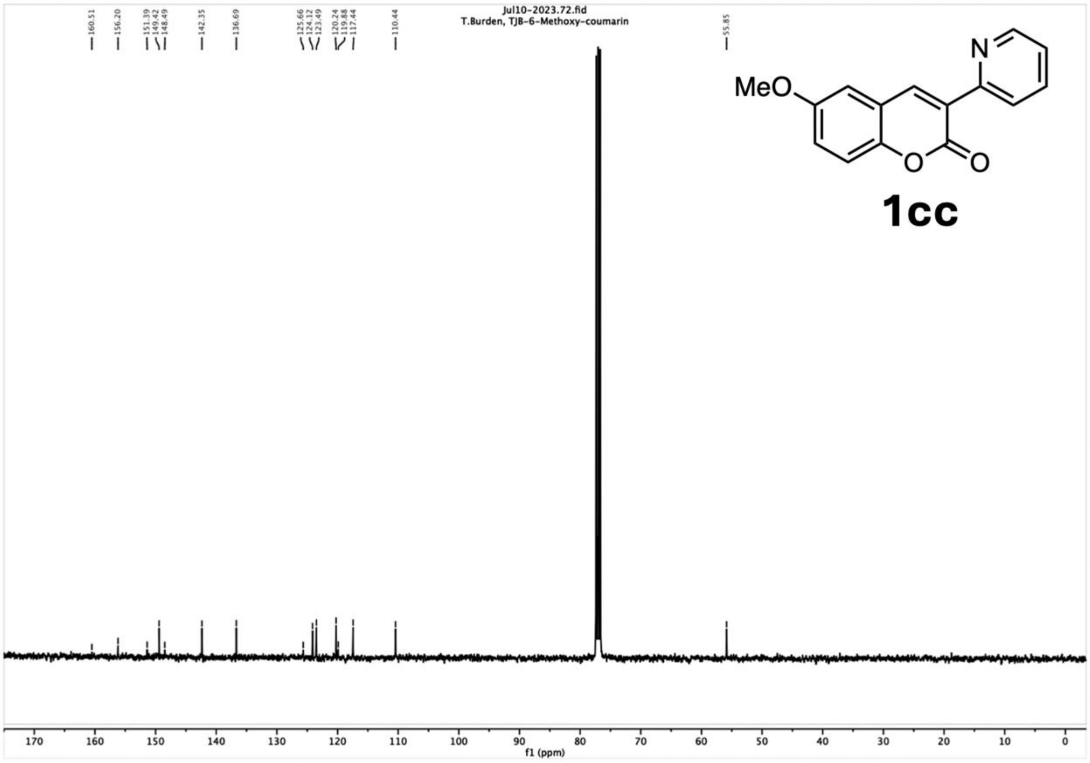

Figure S52 $^{13}C\{^{1}H\}$ NMR Spectrum (101 MHz, $CDCl_3$) of **1cc**.

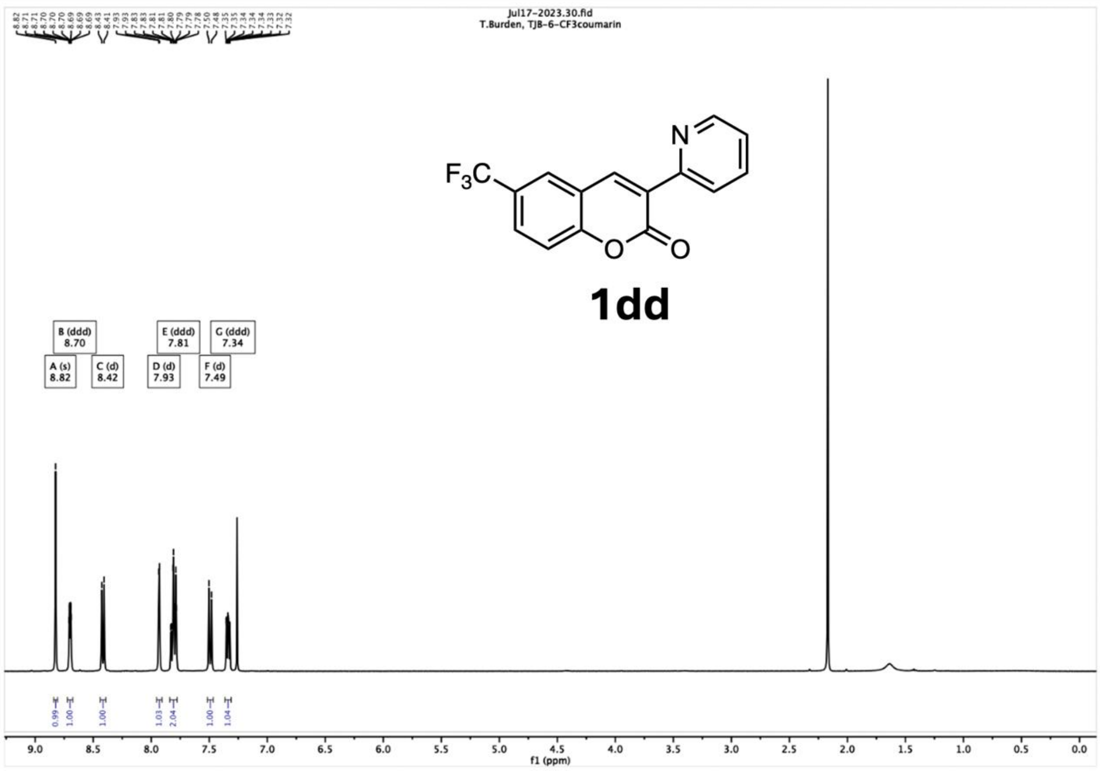

Figure S53 ^{1}H NMR Spectrum (400 MHz, $CDCl_3$) of **1dd**.

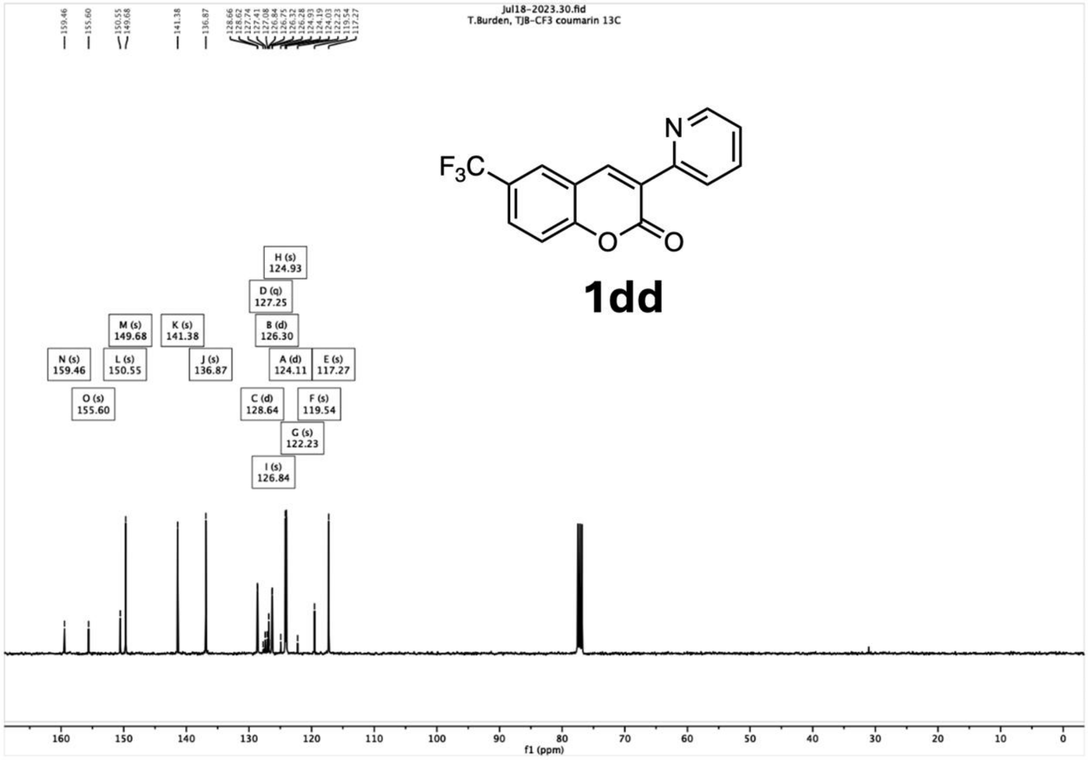

Figure S54 $^{13}C\{^1H\}$ NMR Spectrum (101 MHz, $CDCl_3$) of **1dd**.

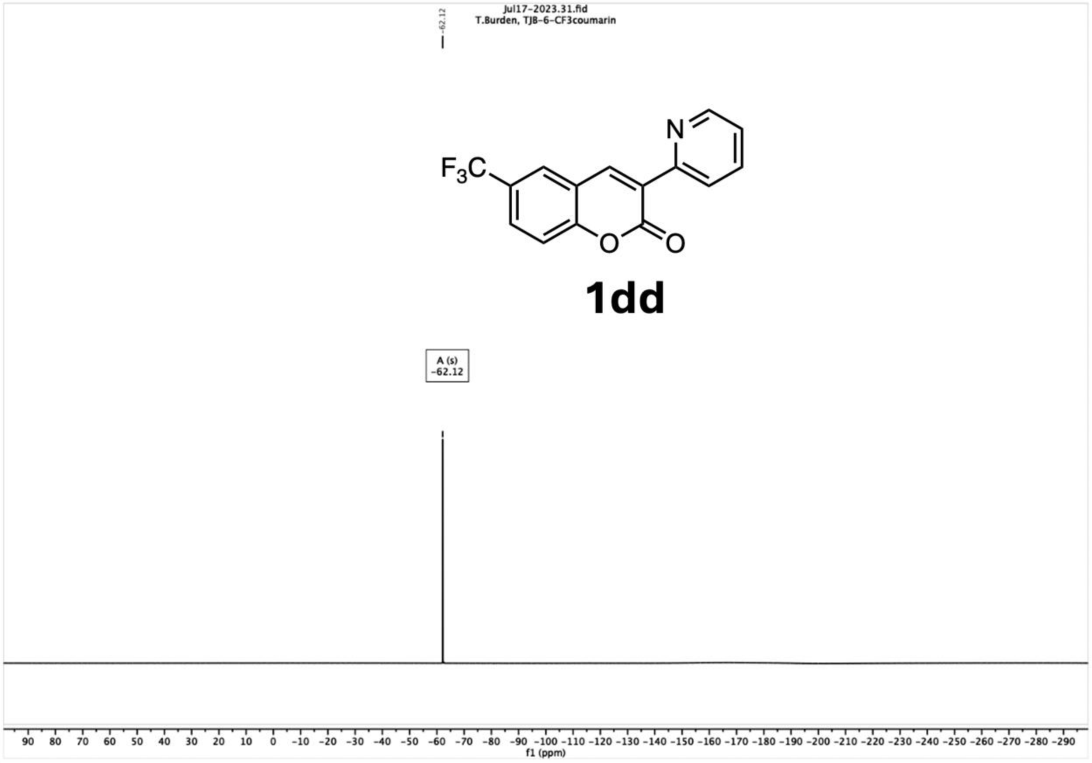

Figure S55 ^{19}F NMR Spectrum (376 MHz, $CDCl_3$) of **1dd**.

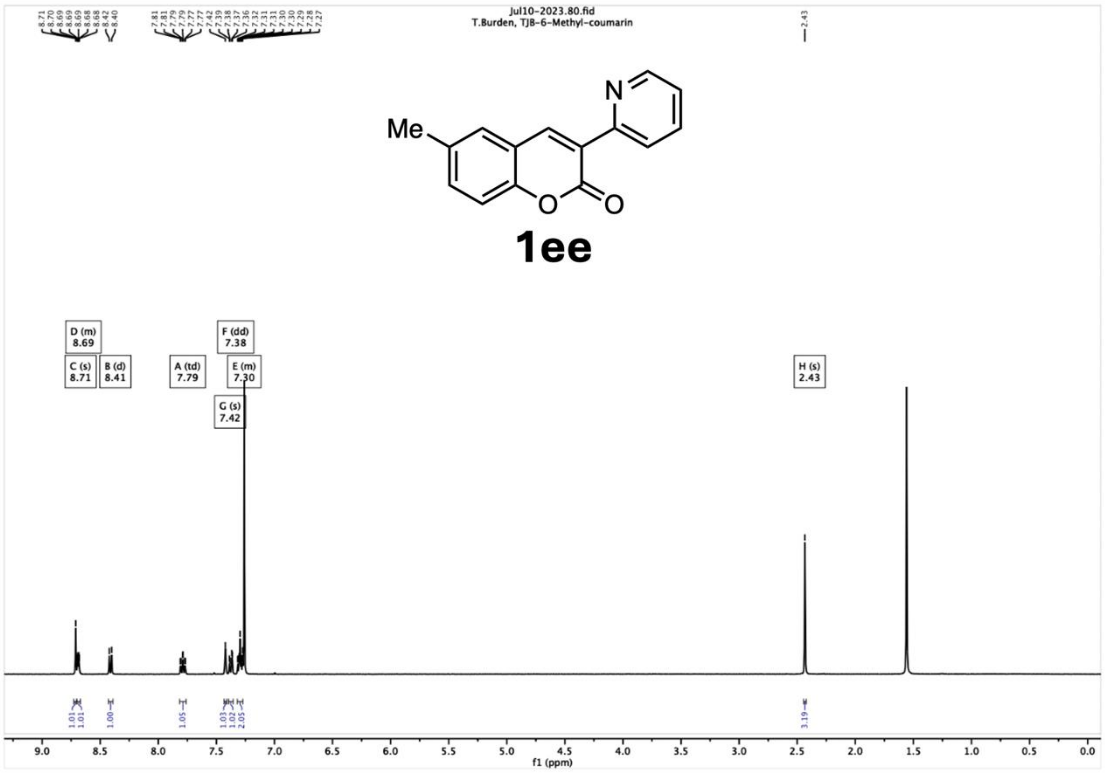

Figure S56 ^{1}H NMR Spectrum (400 MHz, $CDCl_3$) of **1ee**.

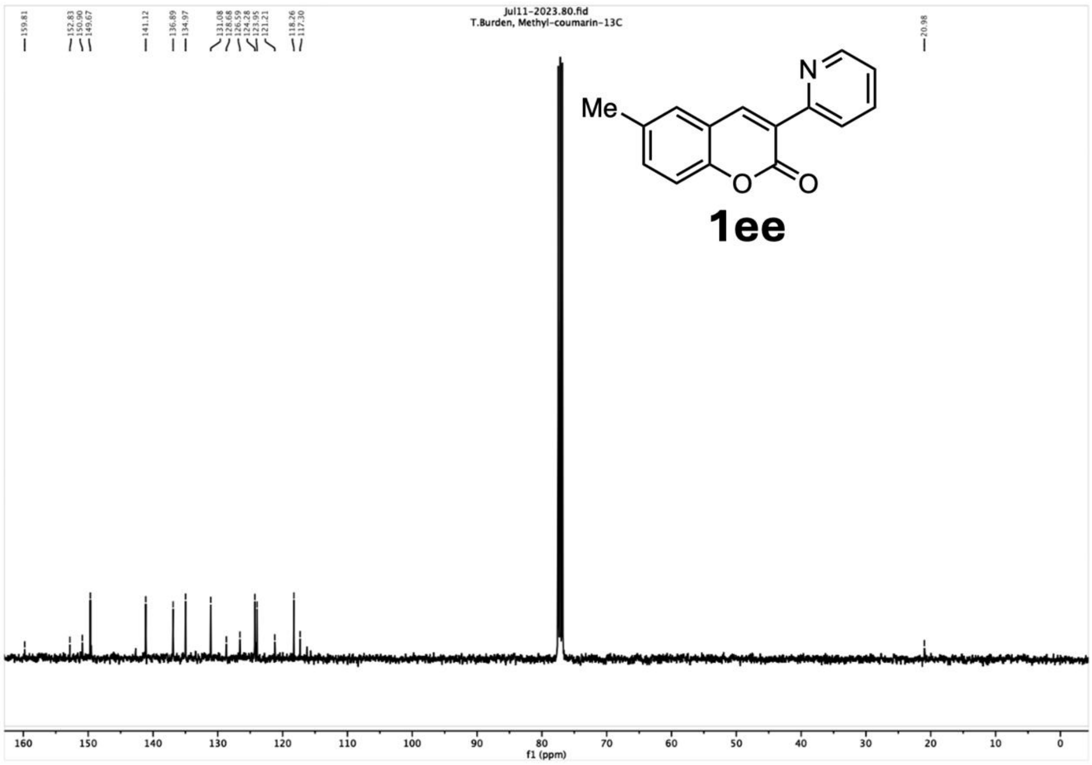

Figure S57 $^{13}C\{^{1}H\}$ NMR Spectrum (101 MHz, $CDCl_3$) of **1ee**.

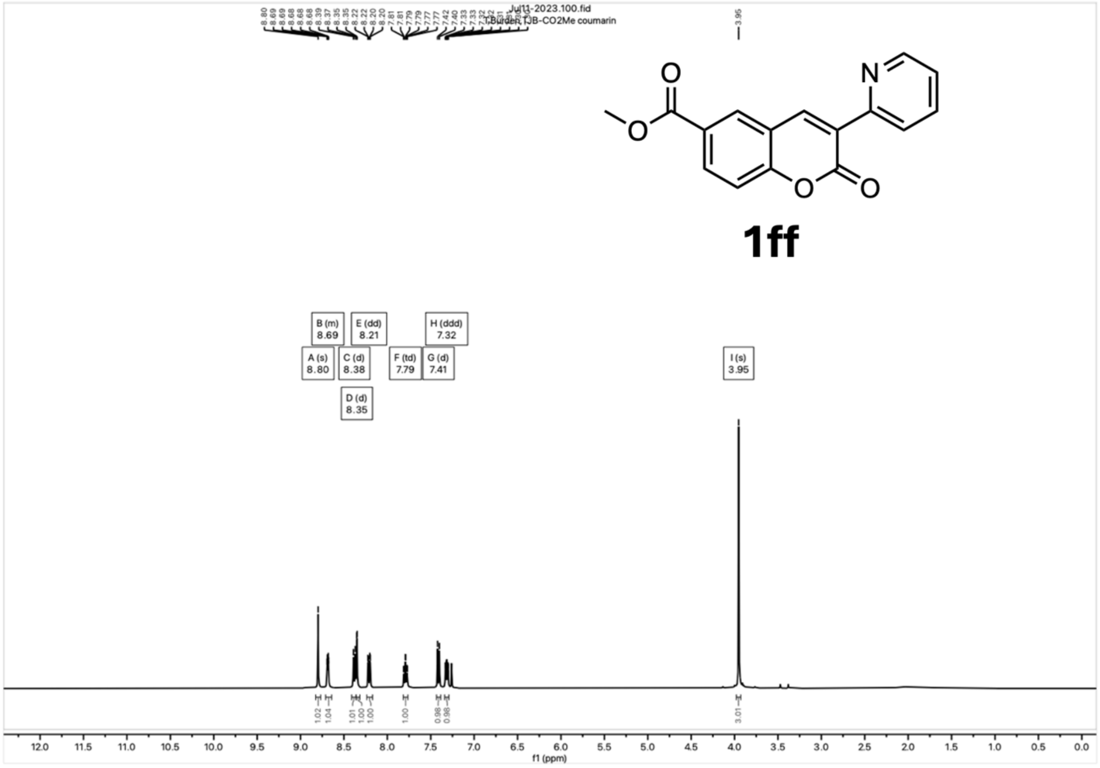

Figure S58 ^{1}H NMR Spectrum (400 MHz, $CDCl_3$) of **1ff**.

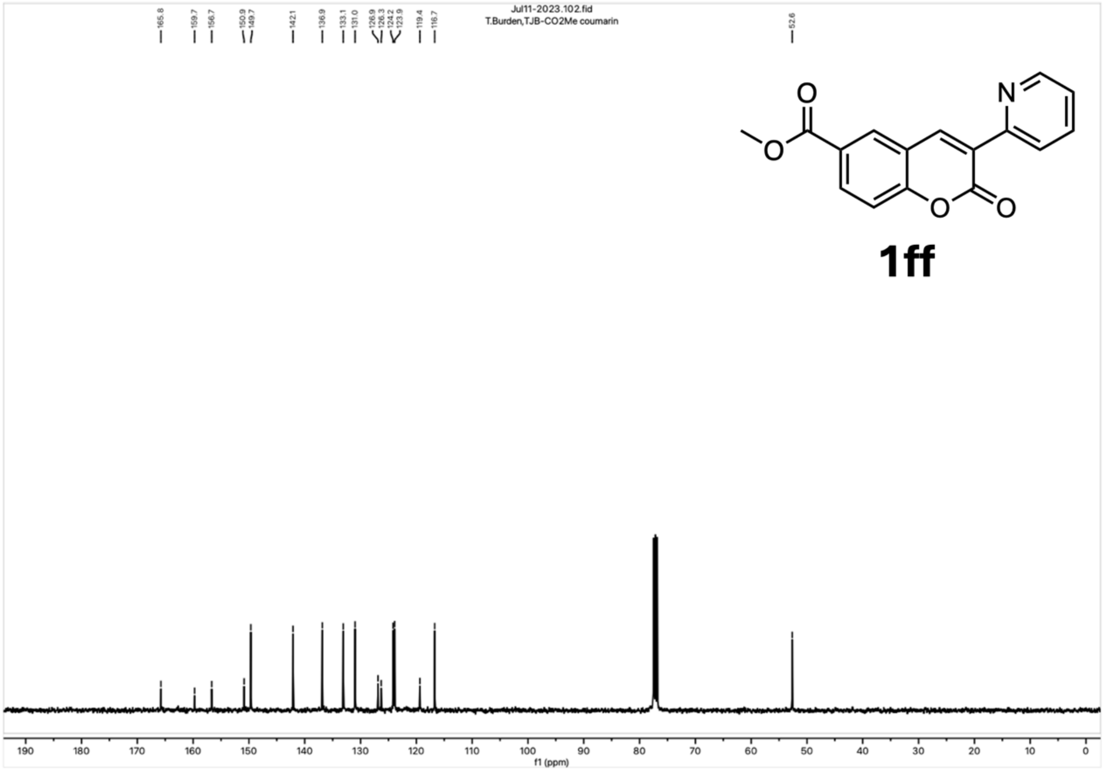

Figure S59 $^{13}C\{^{1}H\}$ NMR Spectrum (101 MHz, $CDCl_3$) of **1ff**.

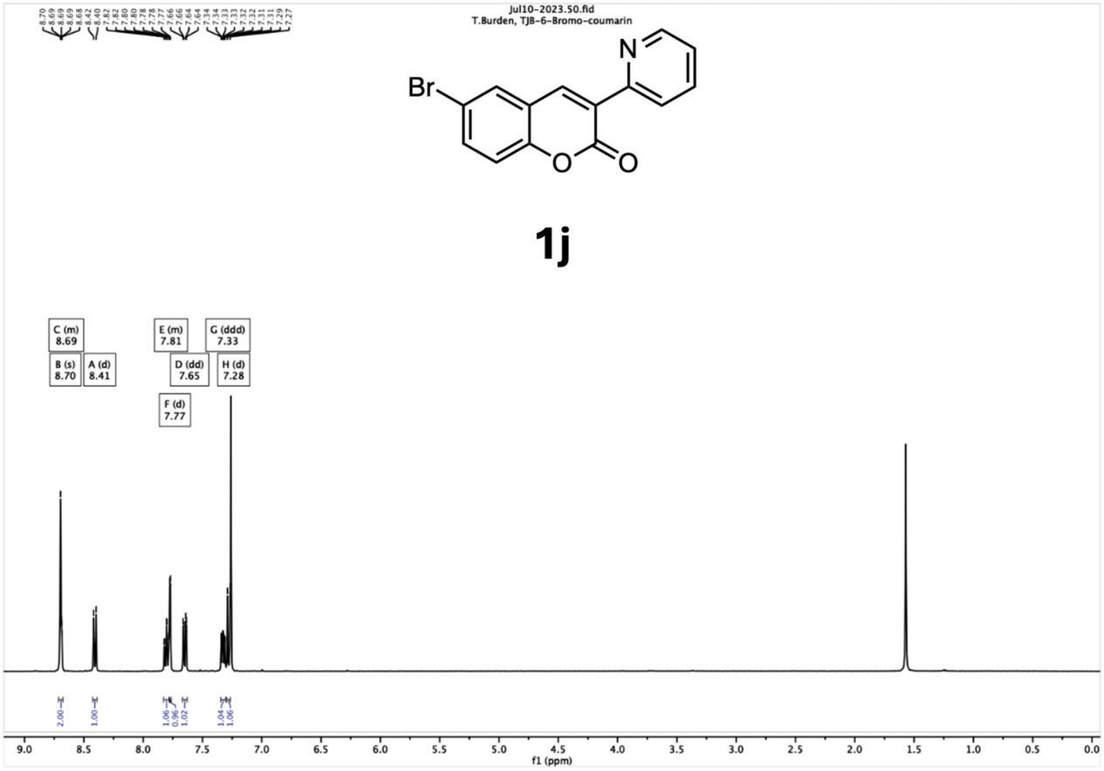

Figure S60 ^{1}H NMR Spectrum (400 MHz, $CDCl_3$) of **1j**.

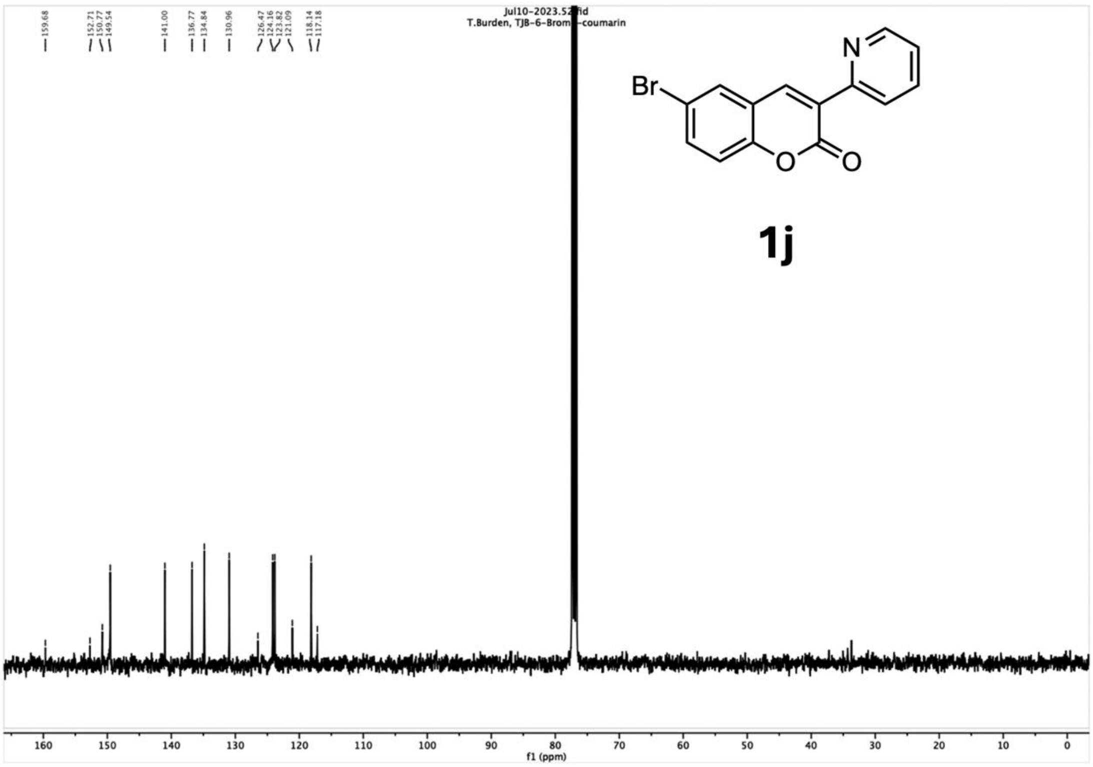

Figure S61 $^{13}C\{^{1}H\}$ NMR Spectrum (101 MHz, $CDCl_3$) of **1j**.

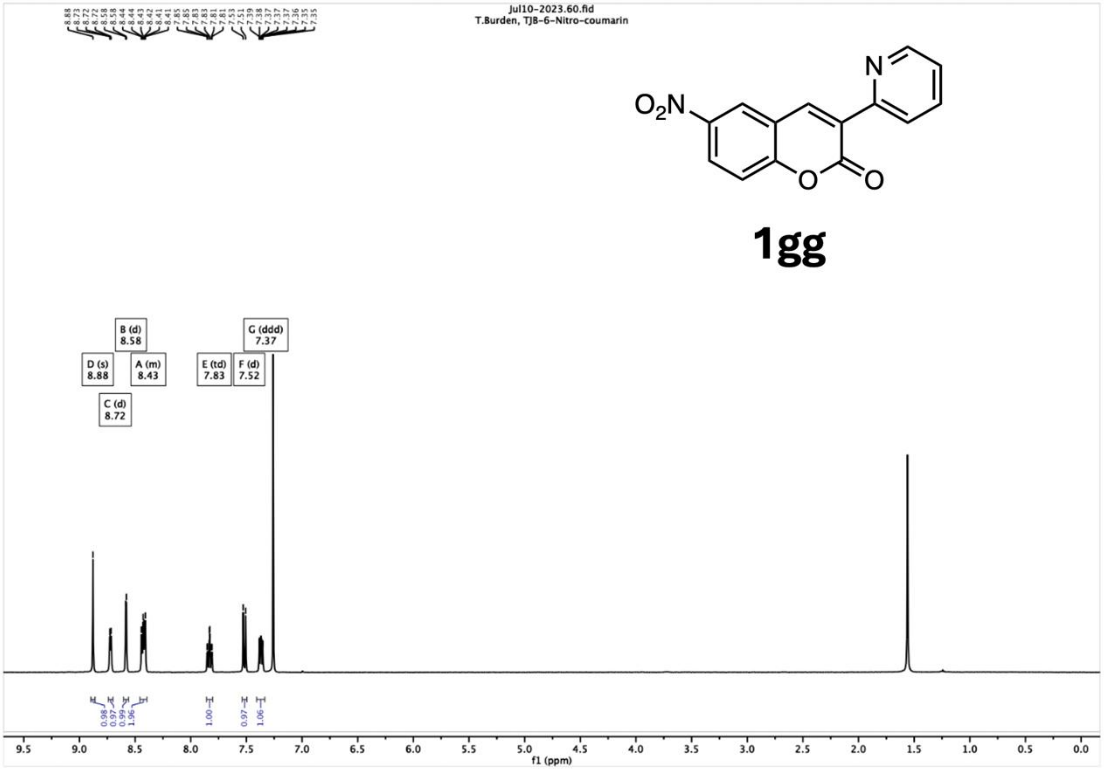

Figure S62 ^{1}H NMR Spectrum (400 MHz, $CDCl_3$) of **1gg**.

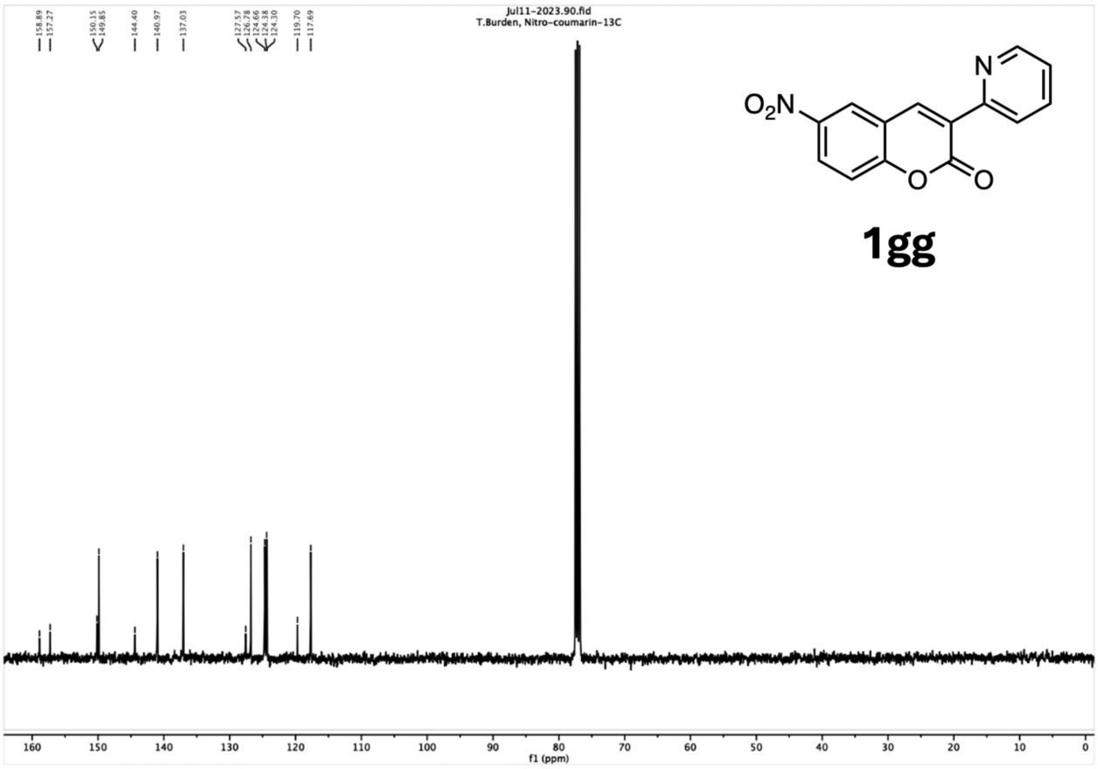

Figure S63 $^{13}C\{^{1}H\}$ NMR Spectrum (101 MHz, $CDCl_3$) of **1gg**.

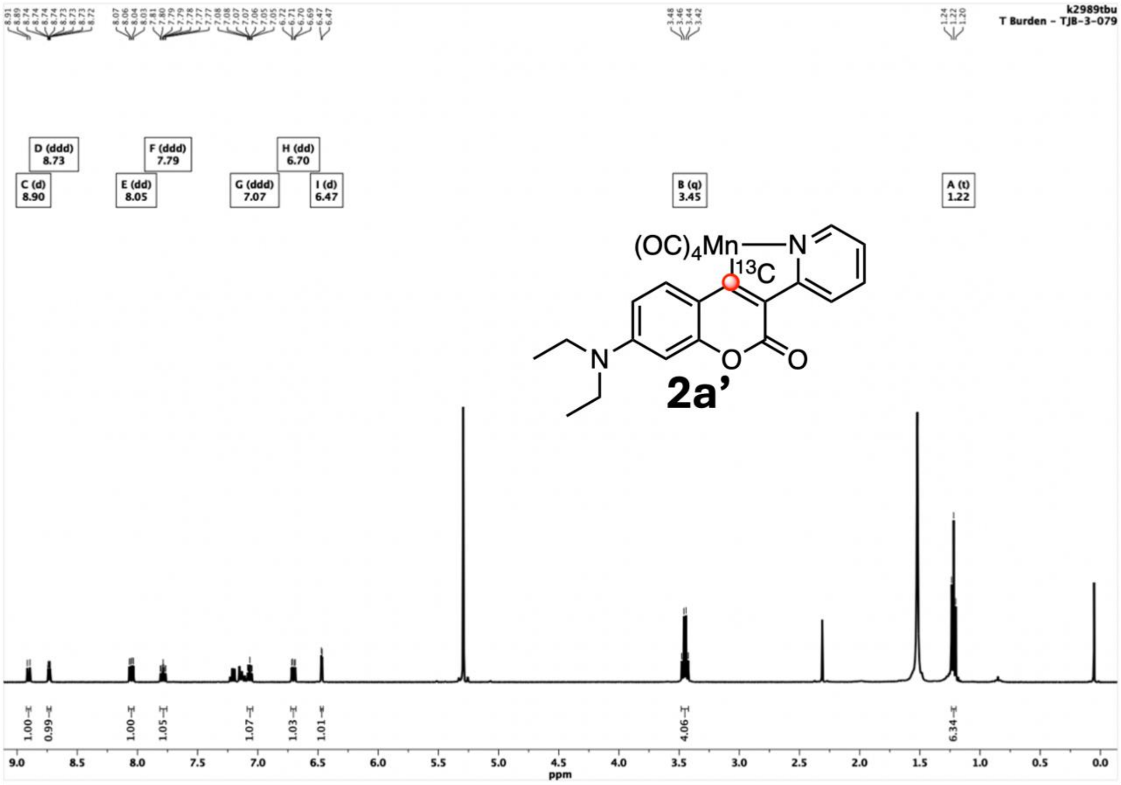

Figure S64 ^{1}H NMR Spectrum (400 MHz, CD_2Cl_2) of **2a'**.

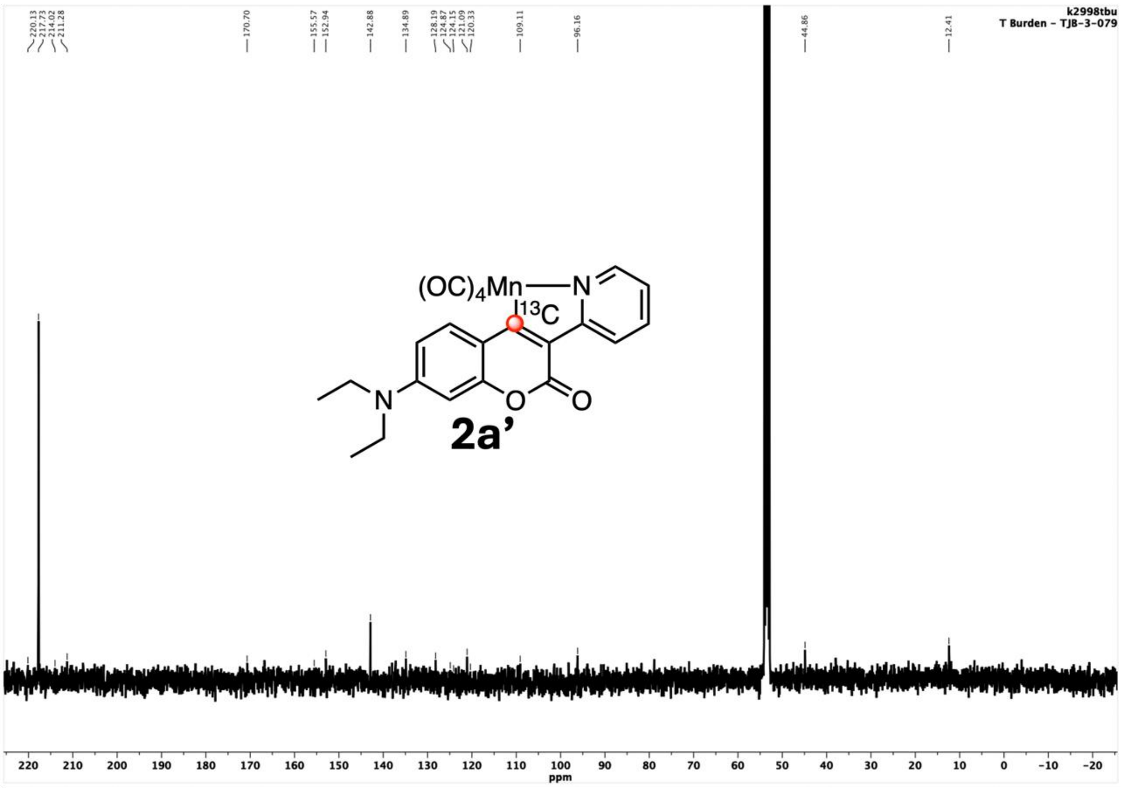

Figure S65 $^{13}C\{^{1}H\}$ NMR Spectrum (101 MHz, $CDCl_3$) of **2a'**.

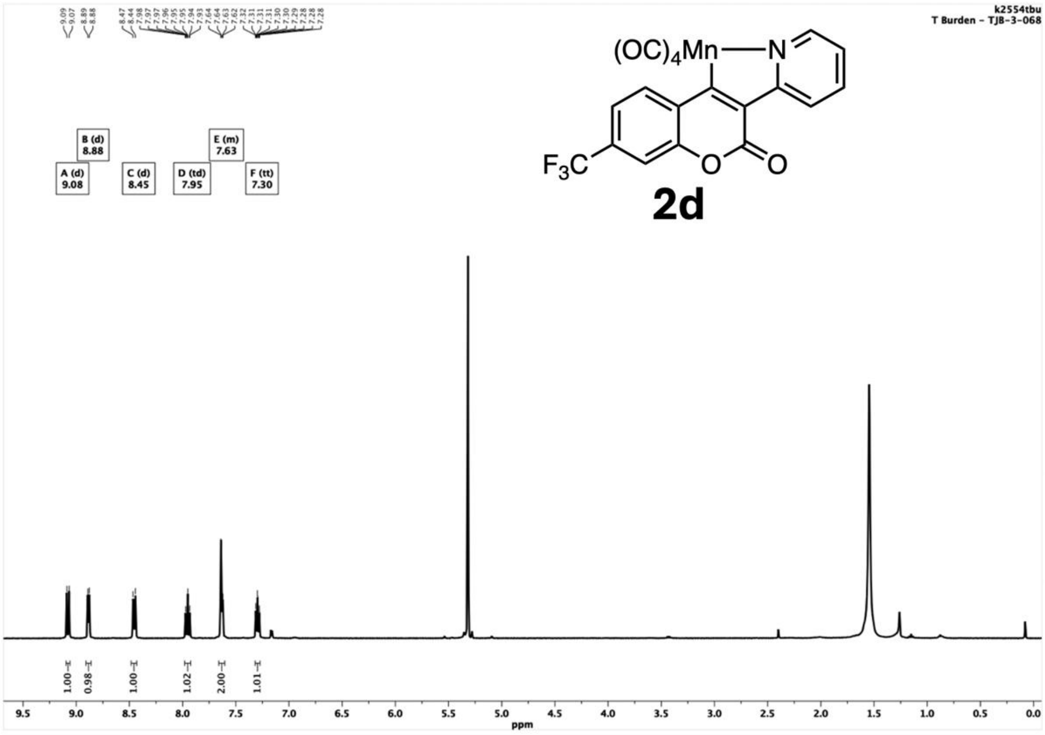

Figure S66 ^{1}H NMR Spectrum (400 MHz, CD_2Cl_2) of **2d**.

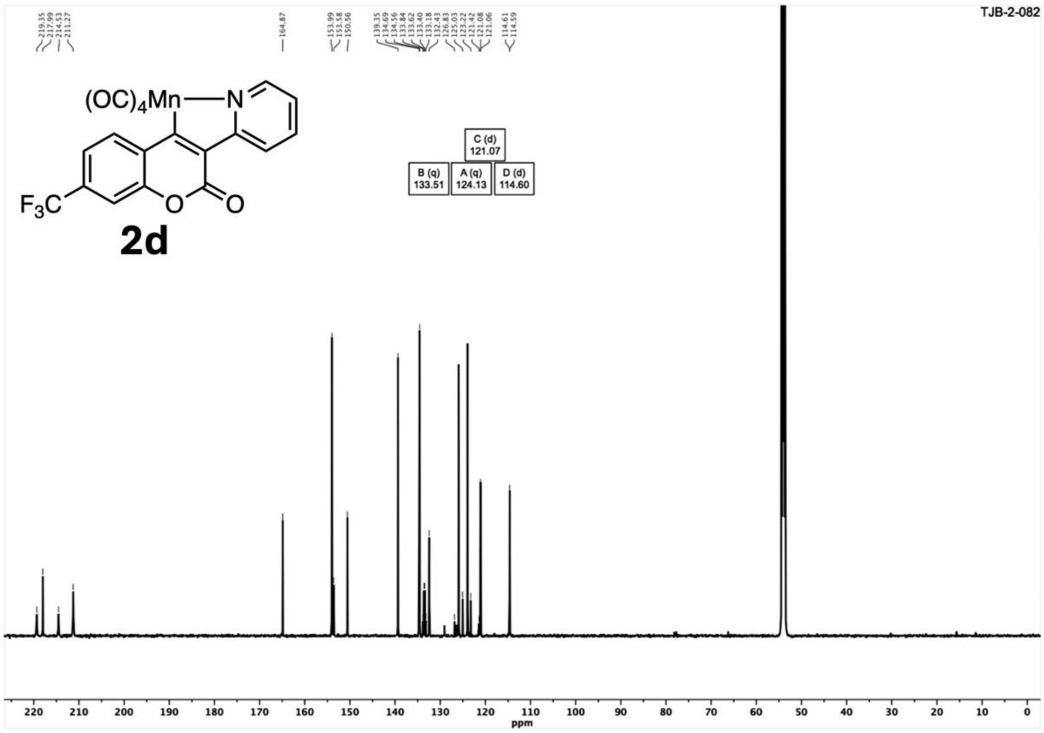

Figure S67 $^{13}C\{^{1}H\}$ NMR Spectrum (151 MHz, CD_2Cl_2) of **2d**.

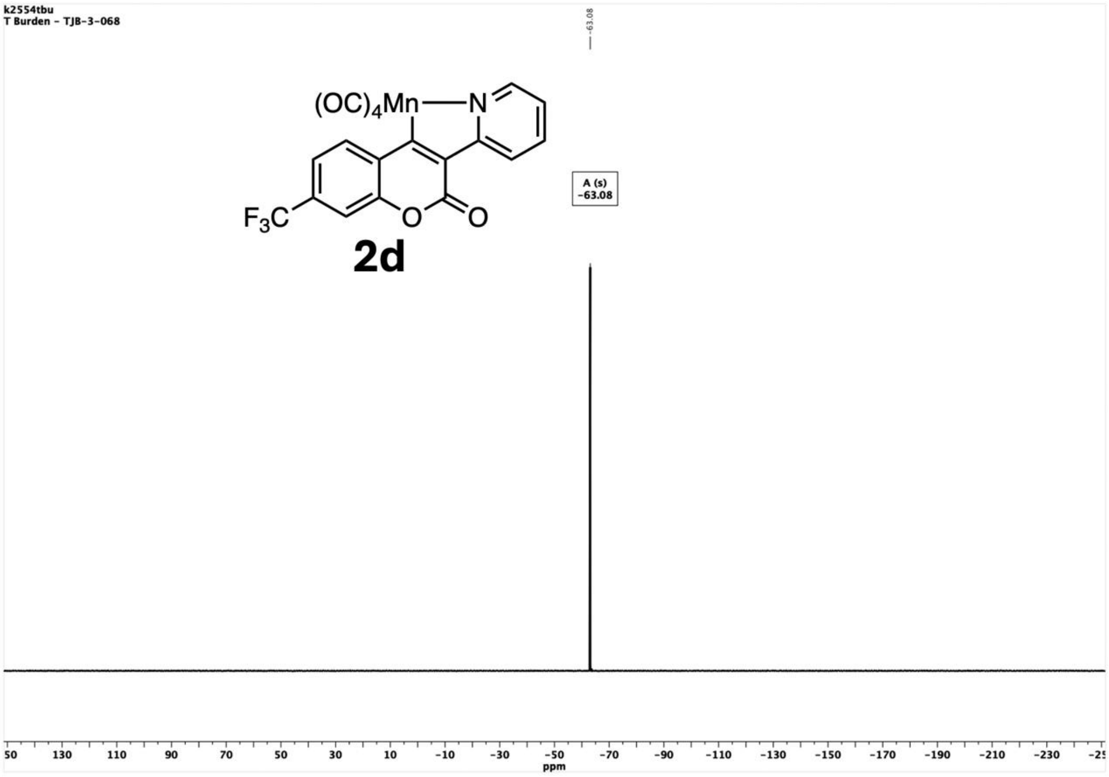

Figure S68 ^{19}F NMR Spectrum (376 MHz, CD_2Cl_2) of **2d**.

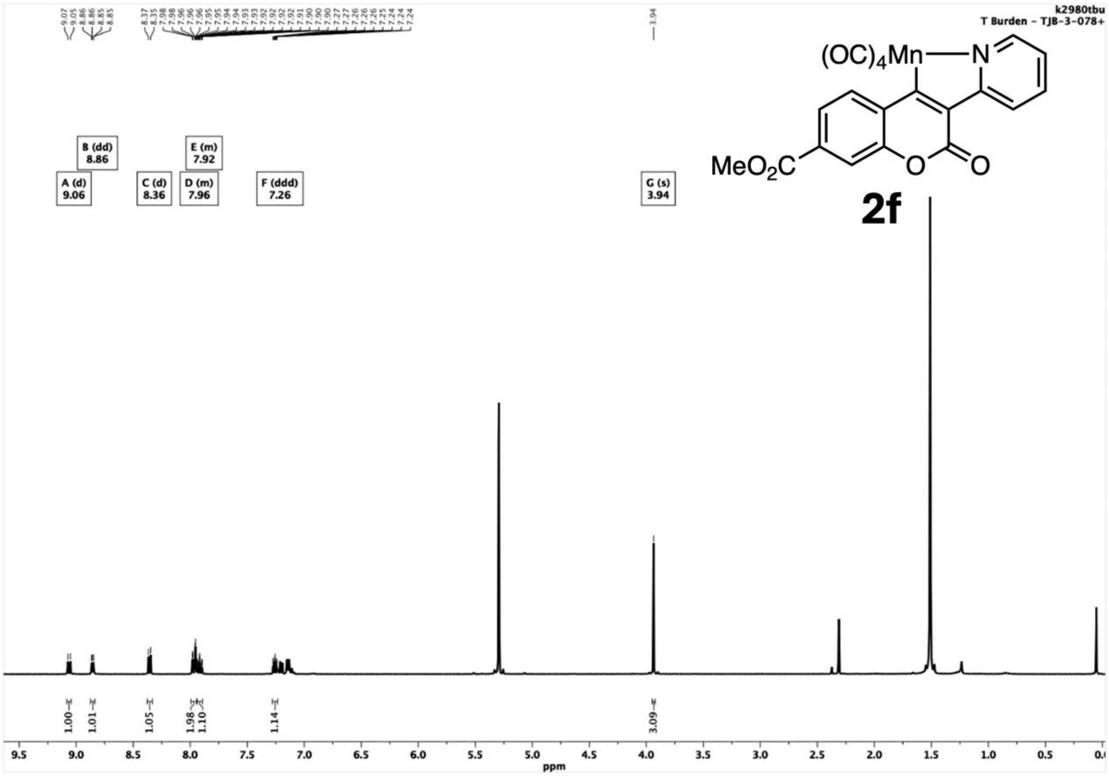

Figure S69 ^{1}H NMR Spectrum (400 MHz, CD_2Cl_2) of **2f**.

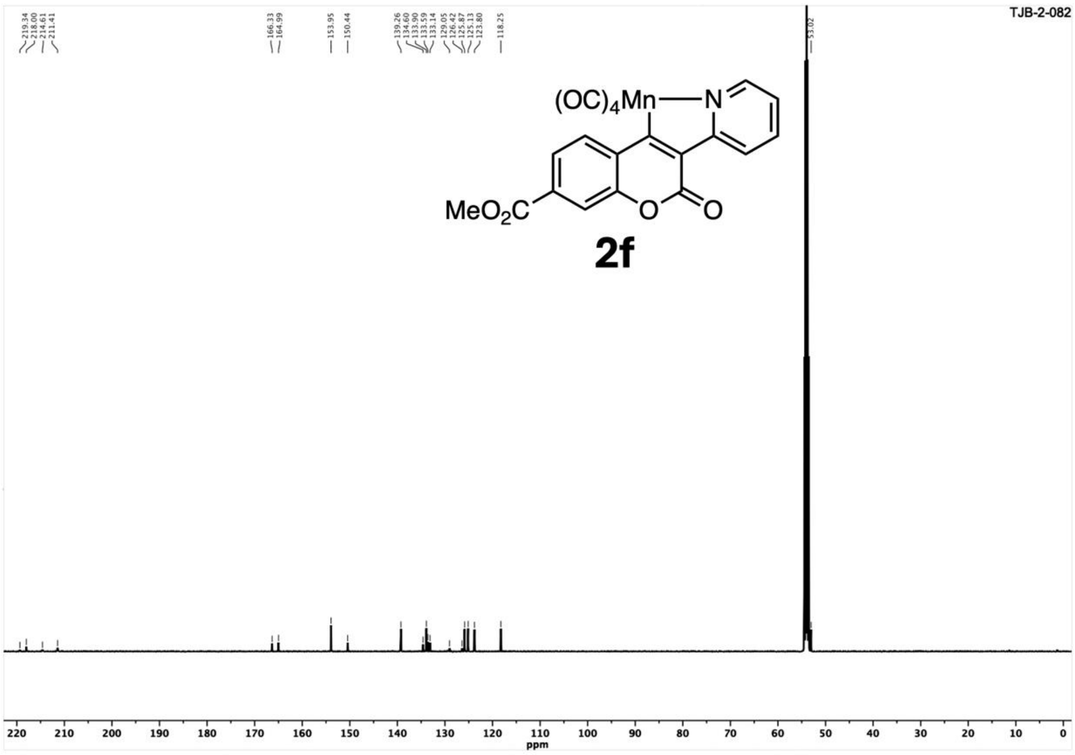

Figure S70 $^{13}C\{^{1}H\}$ NMR Spectrum (126 MHz, CD_2Cl_2) of **2f**.

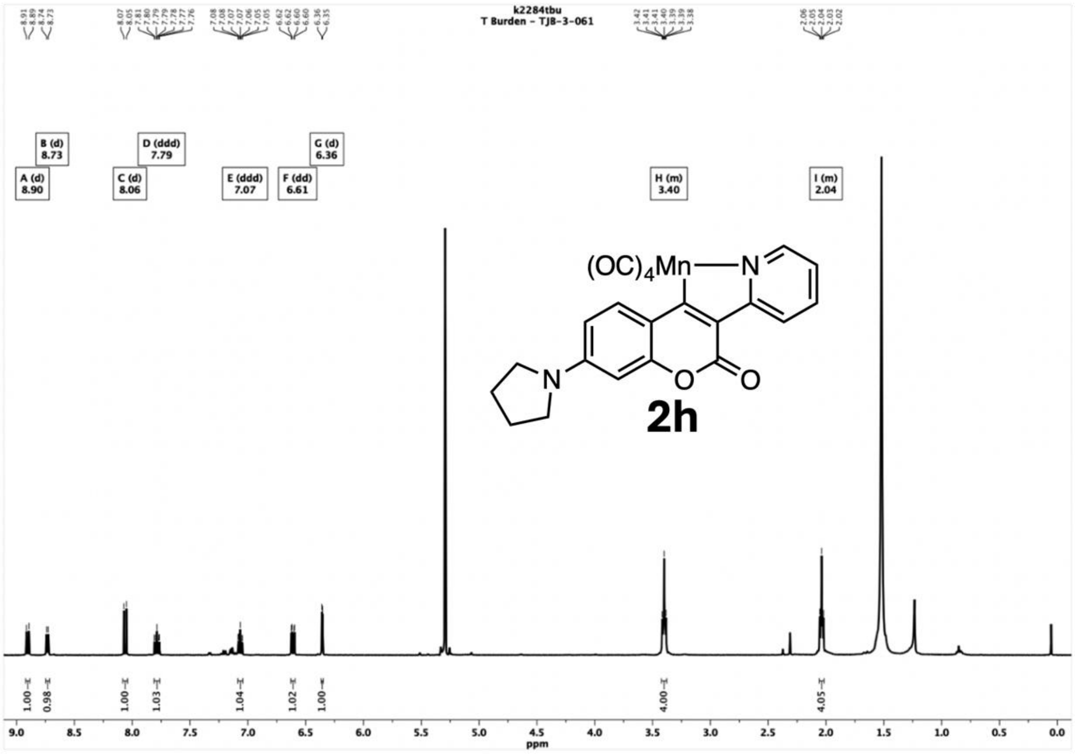

Figure S71 ^{1}H NMR Spectrum (400 MHz, CD_2Cl_2) of **2h**.

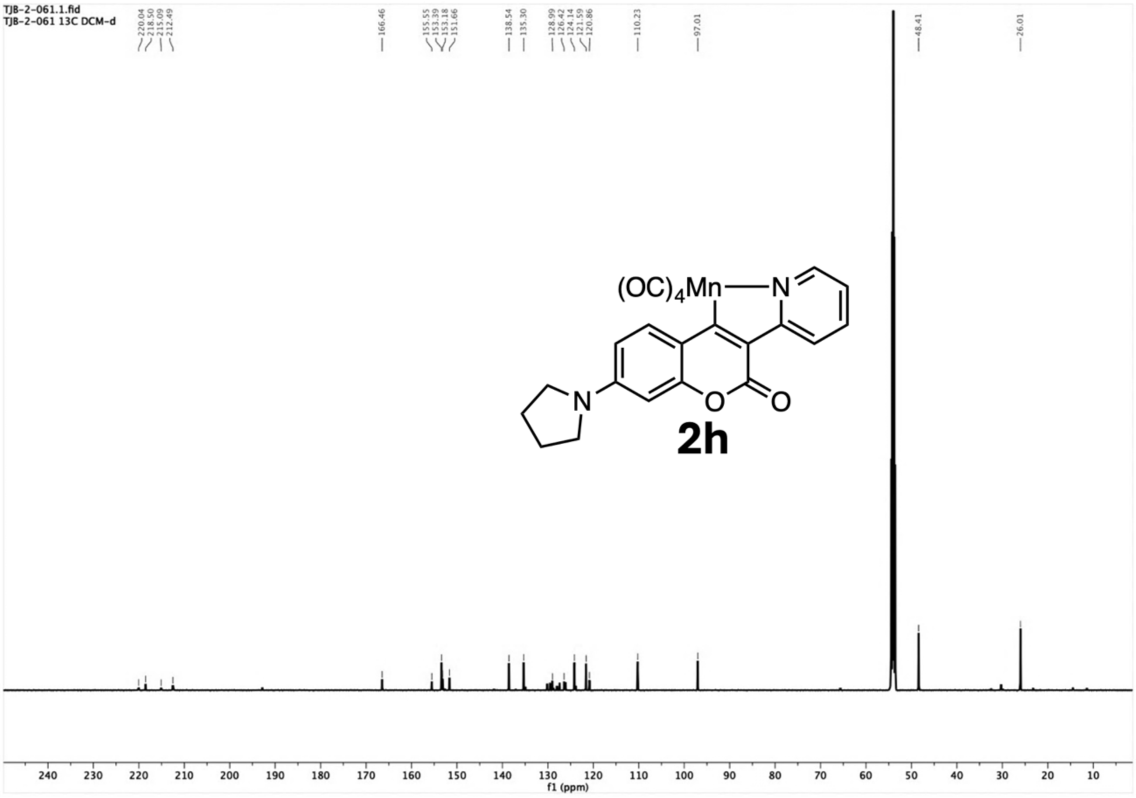

Figure S72 $^{13}C\{^1H\}$ NMR Spectrum (126 MHz, CD_2Cl_2) of **2h**.

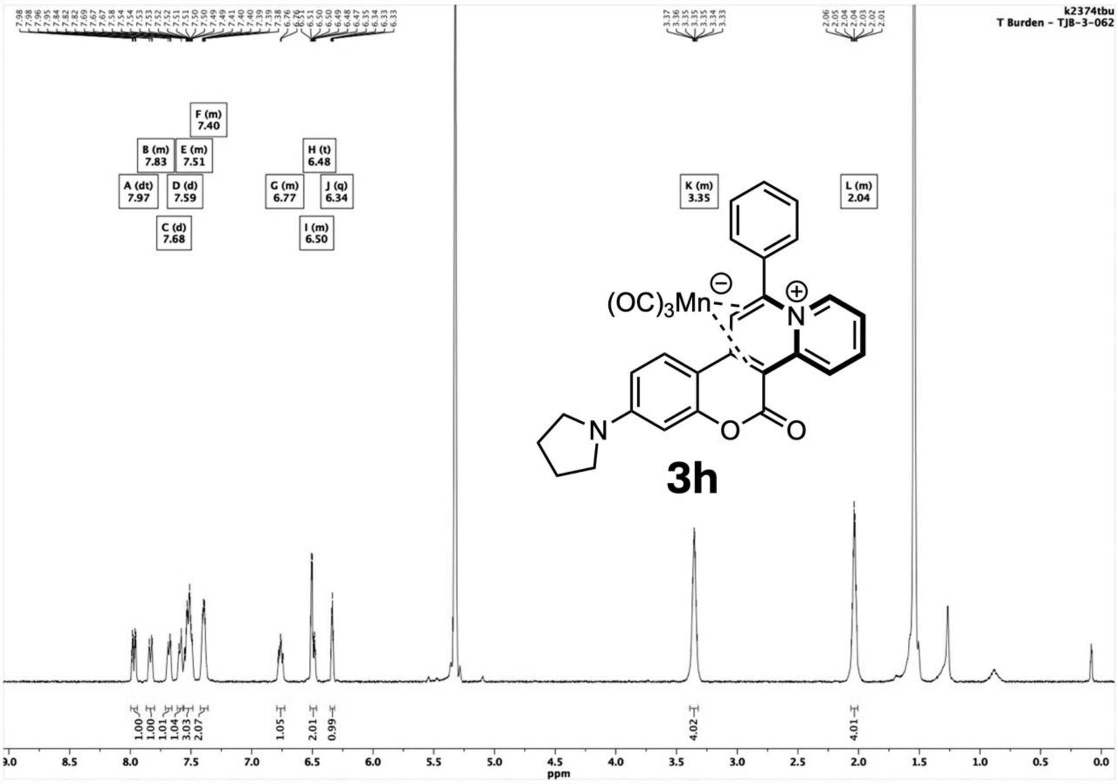

Figure S73 ^1H NMR Spectrum (400 MHz, CD_2Cl_2) of **3h**.

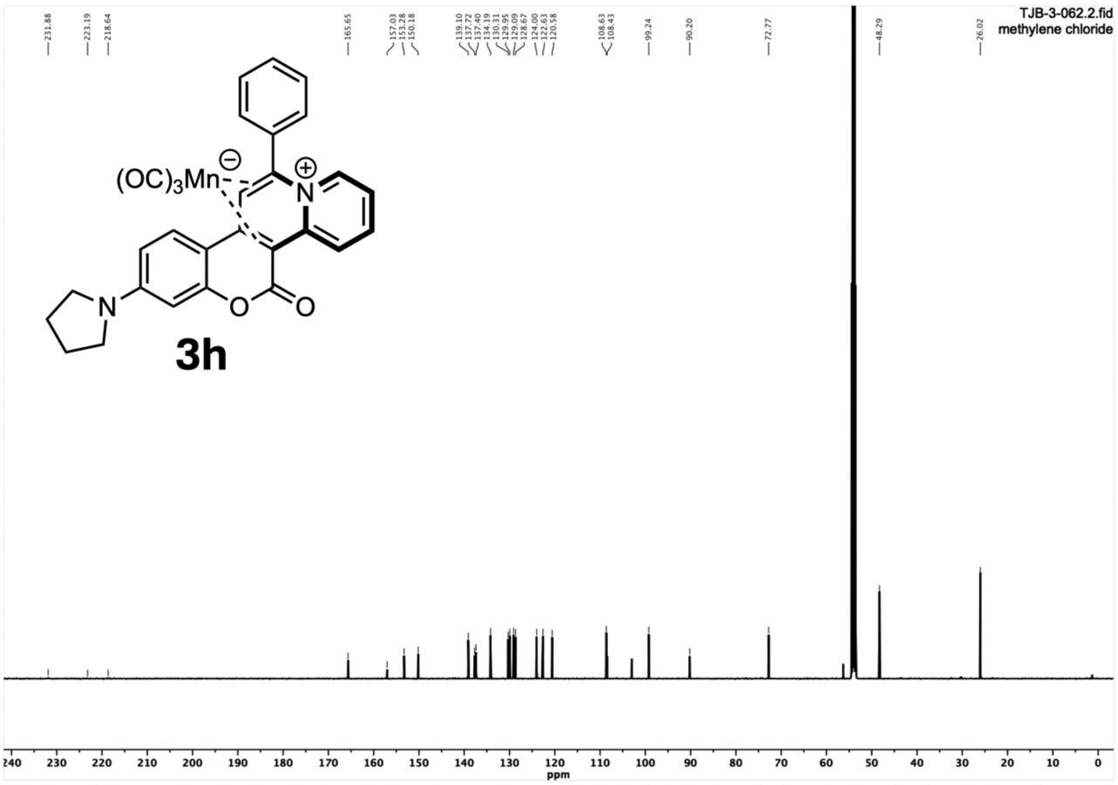

Figure S74 $^{13}C\{^{1}H\}$ NMR Spectrum (126 MHz, CD_2Cl_2) of **3h**.

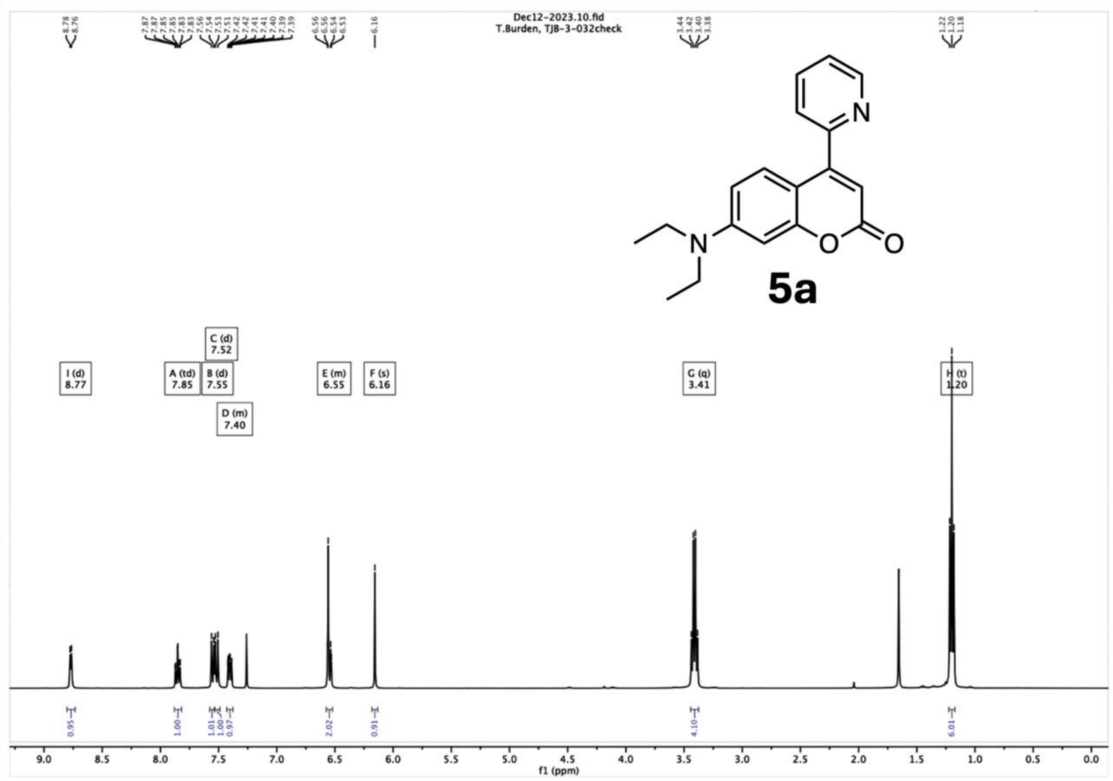

Figure S75 ^{1}H NMR Spectrum (400 MHz, $CDCl_3$) of **5a**.

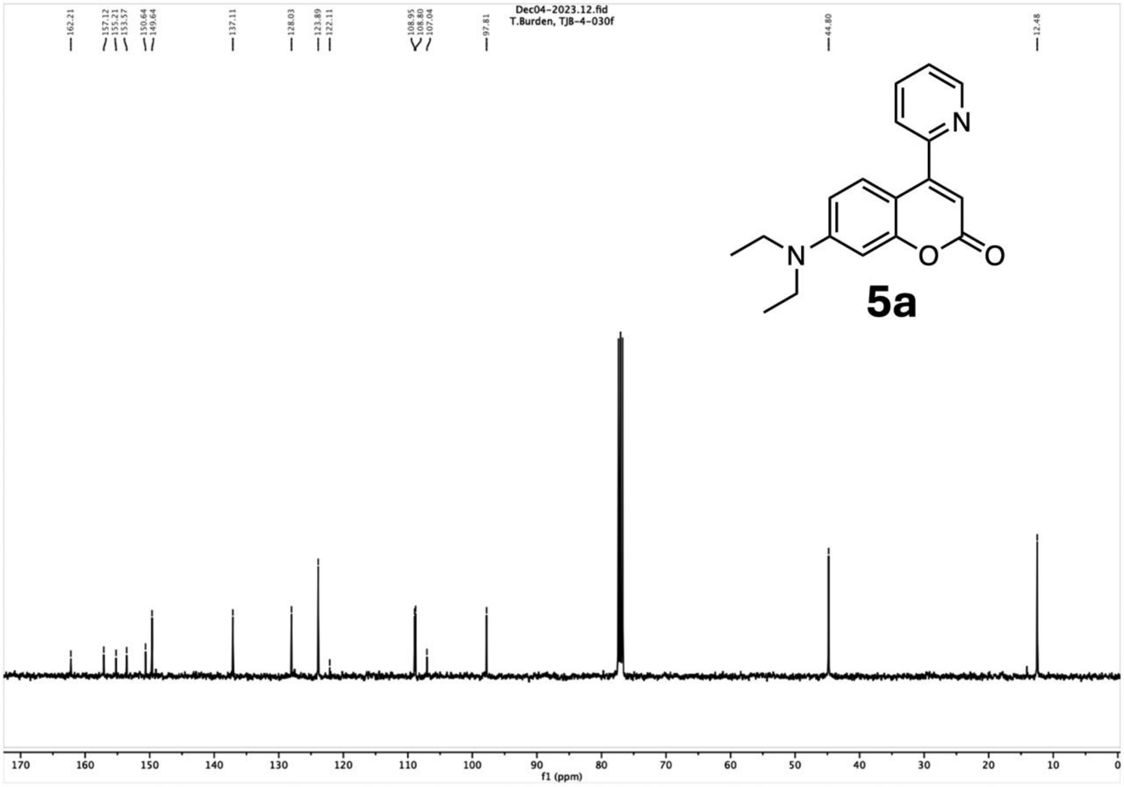

Figure S76 $^{13}C\{^1H\}$ NMR Spectrum (101 MHz, $CDCl_3$) of **5a**.

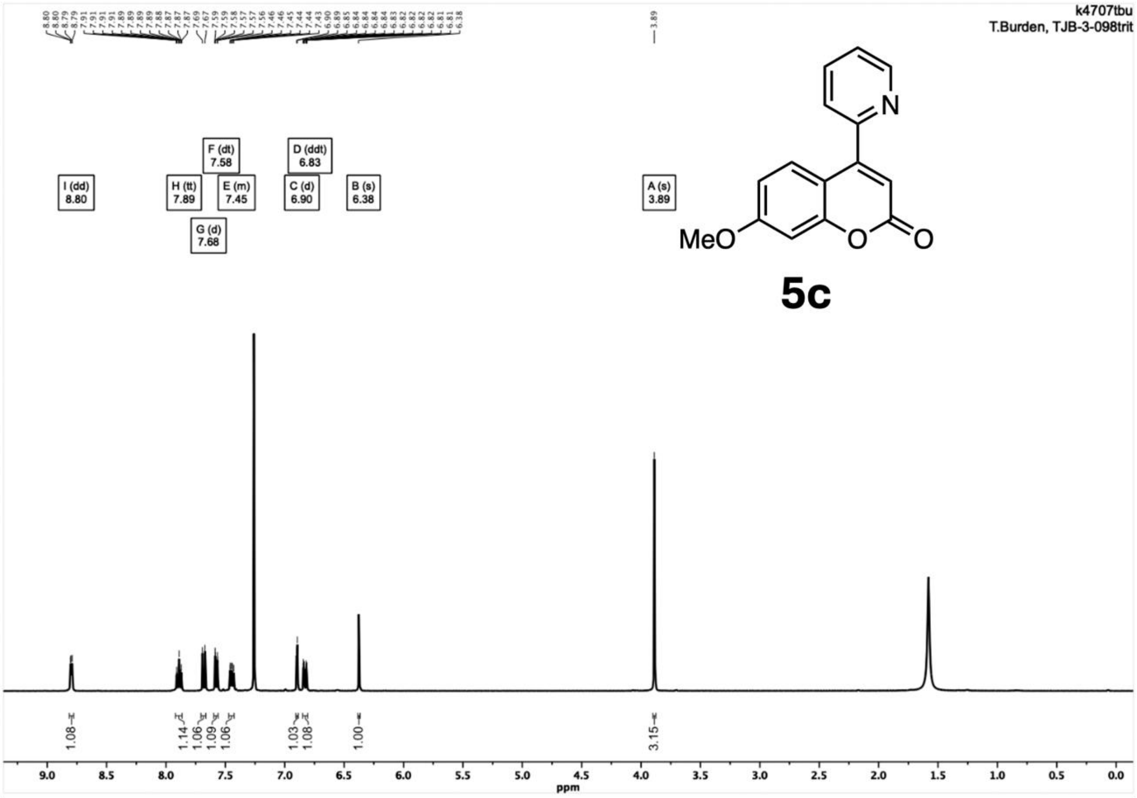

Figure S77 ^1H NMR Spectrum (400 MHz, $CDCl_3$) of **5c**.

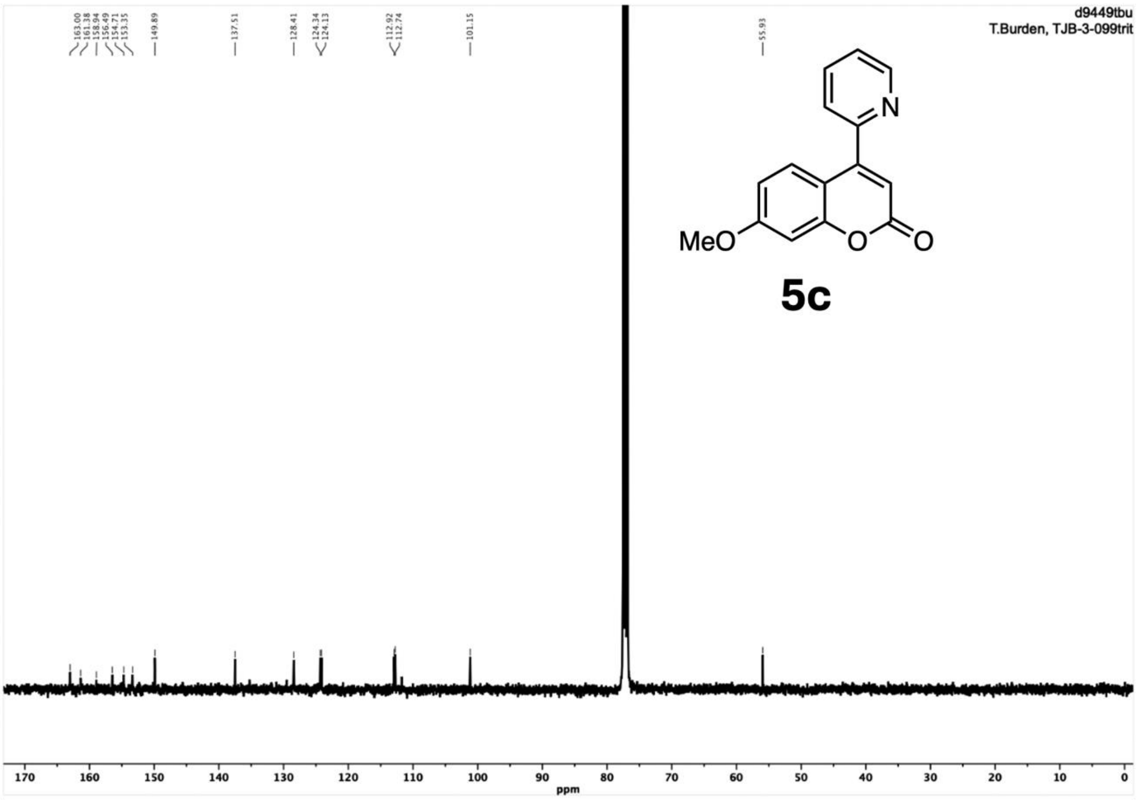

Figure S78 $^{13}C\{^{1}H\}$ NMR Spectrum (101 MHz, $CDCl_3$) of **5c**.

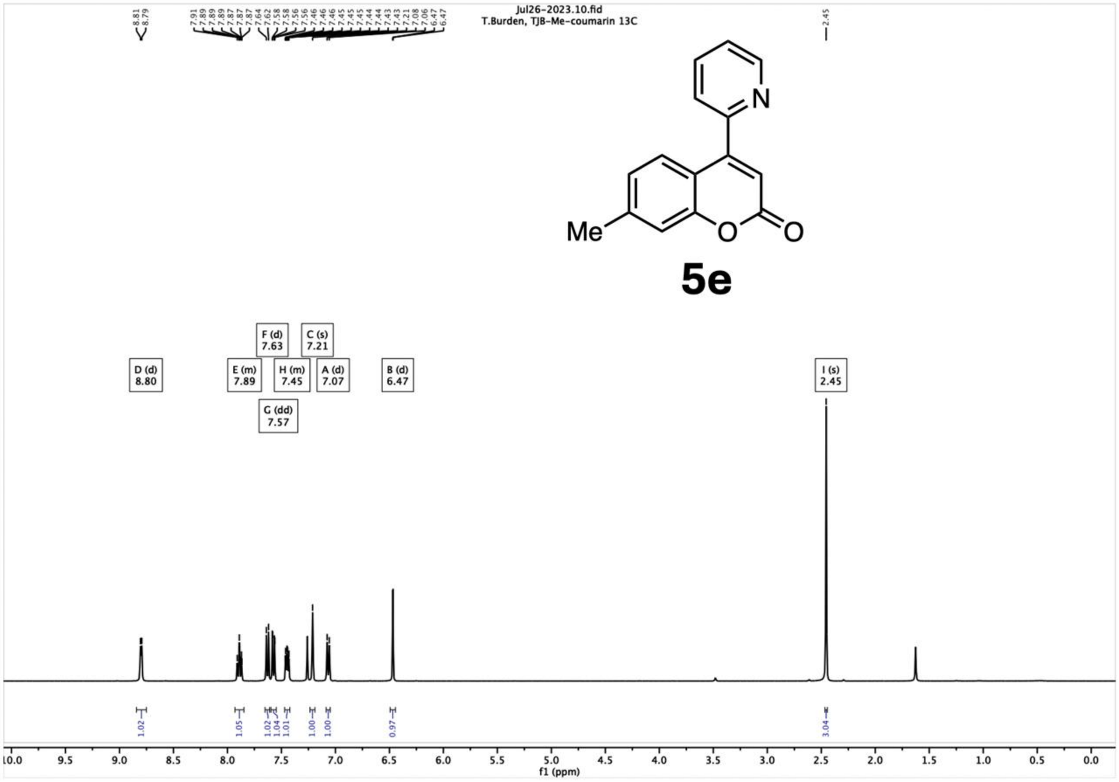

Figure S79 ^{1}H NMR Spectrum (400 MHz, $CDCl_3$) of **5e**.

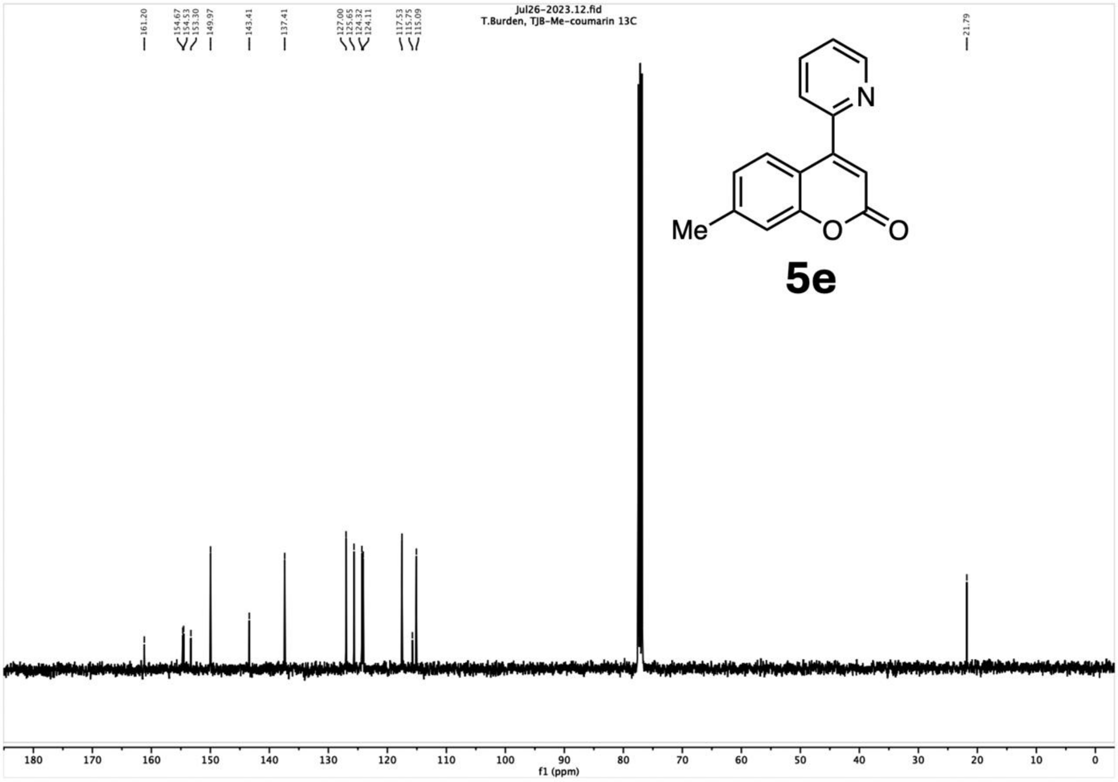

Figure S80 $^{13}C\{^{1}H\}$ NMR Spectrum (101 MHz, $CDCl_3$) of **5e**.

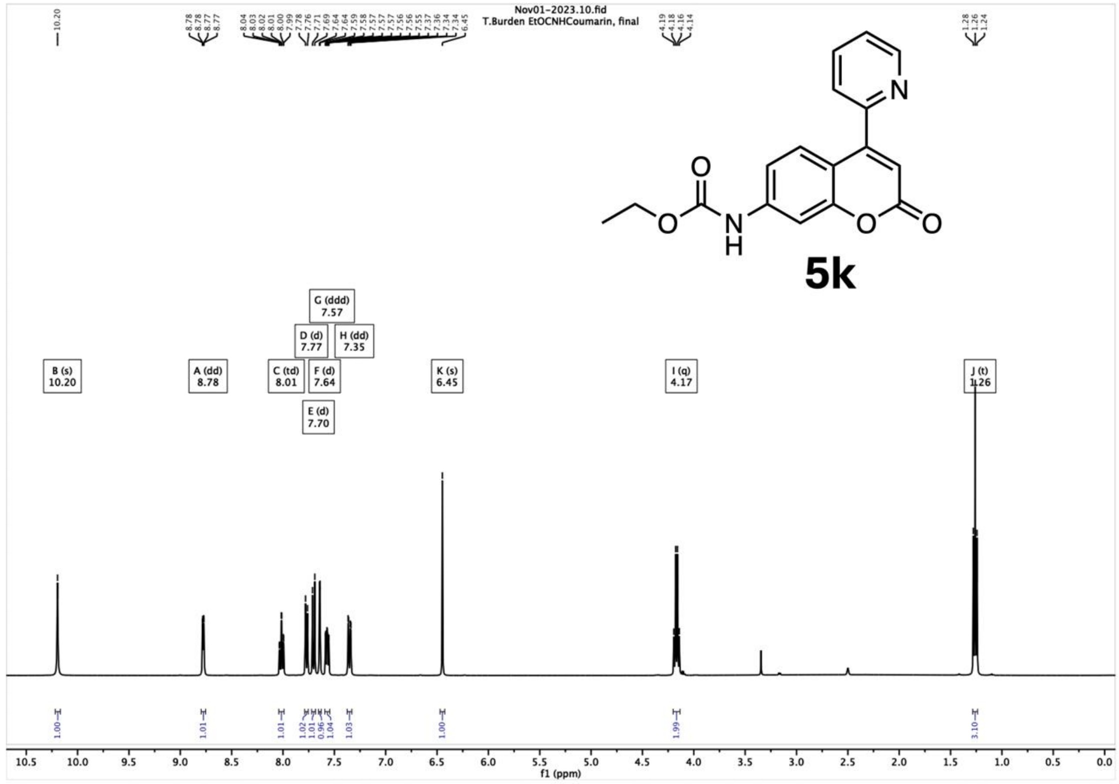

Figure S81 ^{1}H NMR Spectrum (400 MHz, DMSO-d_6) of **5k**.

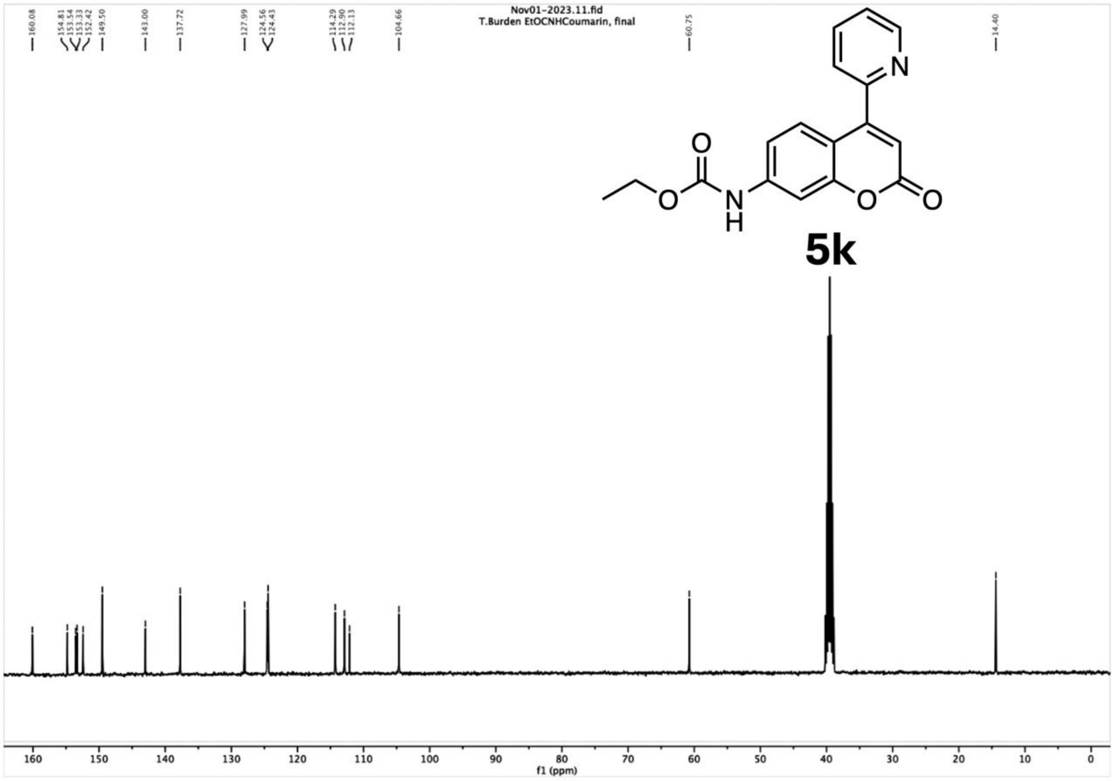

Figure S82 $^{13}C\{^{1}H\}$ NMR Spectrum (101 MHz, DMSO-d_6) of **5k**.

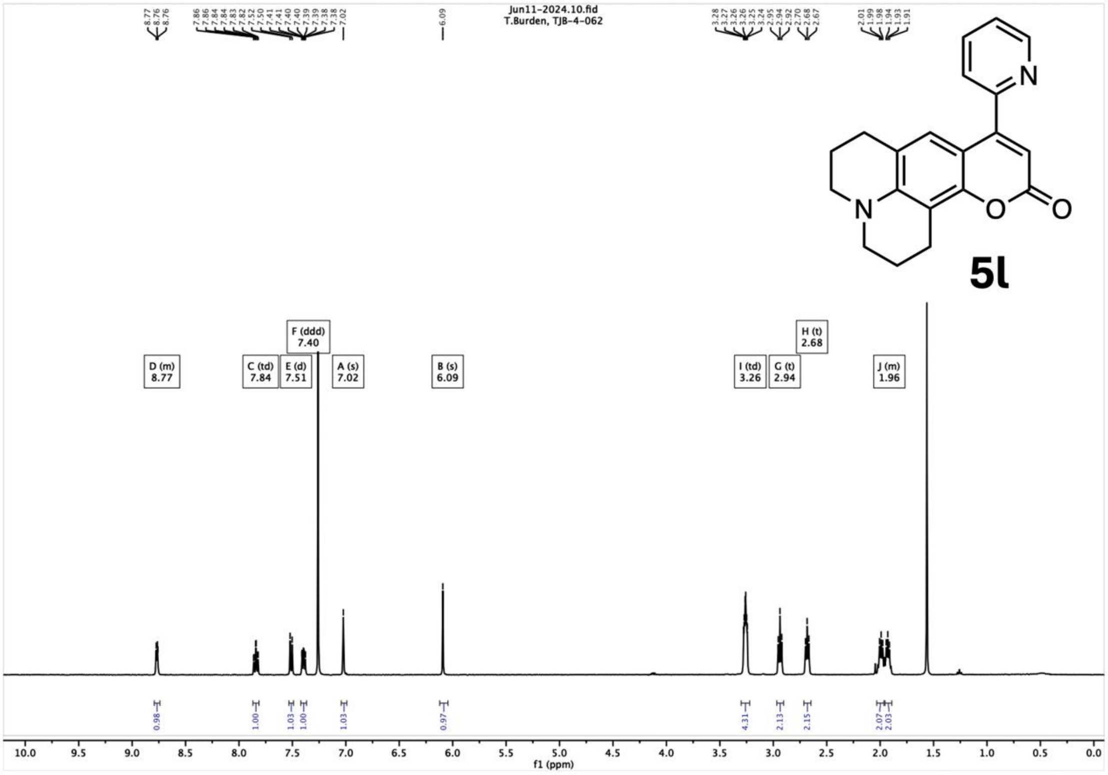

Figure S83 ^{1}H NMR Spectrum (400 MHz, $CDCl_3$) of **5l**.

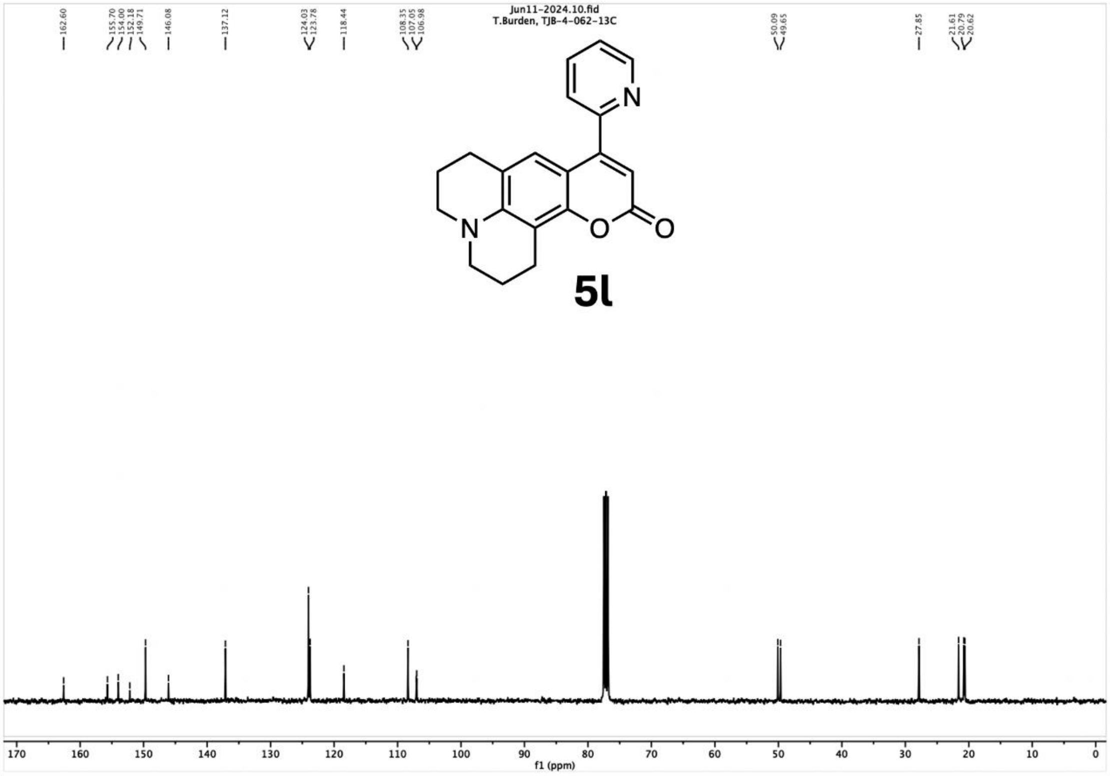

Figure S84 $^{13}C\{^{1}H\}$ NMR Spectrum (101 MHz, $CDCl_3$) of **5l**.

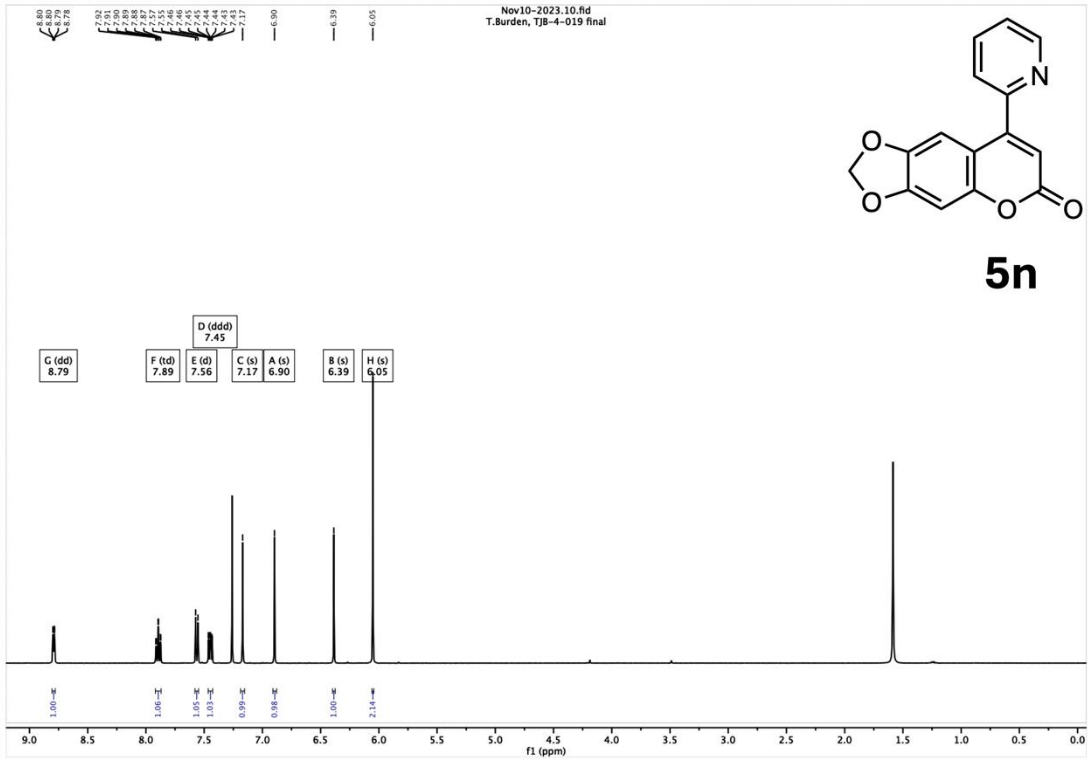

Figure S85 ^{1}H NMR Spectrum (400 MHz, $CDCl_3$) of **5n**.

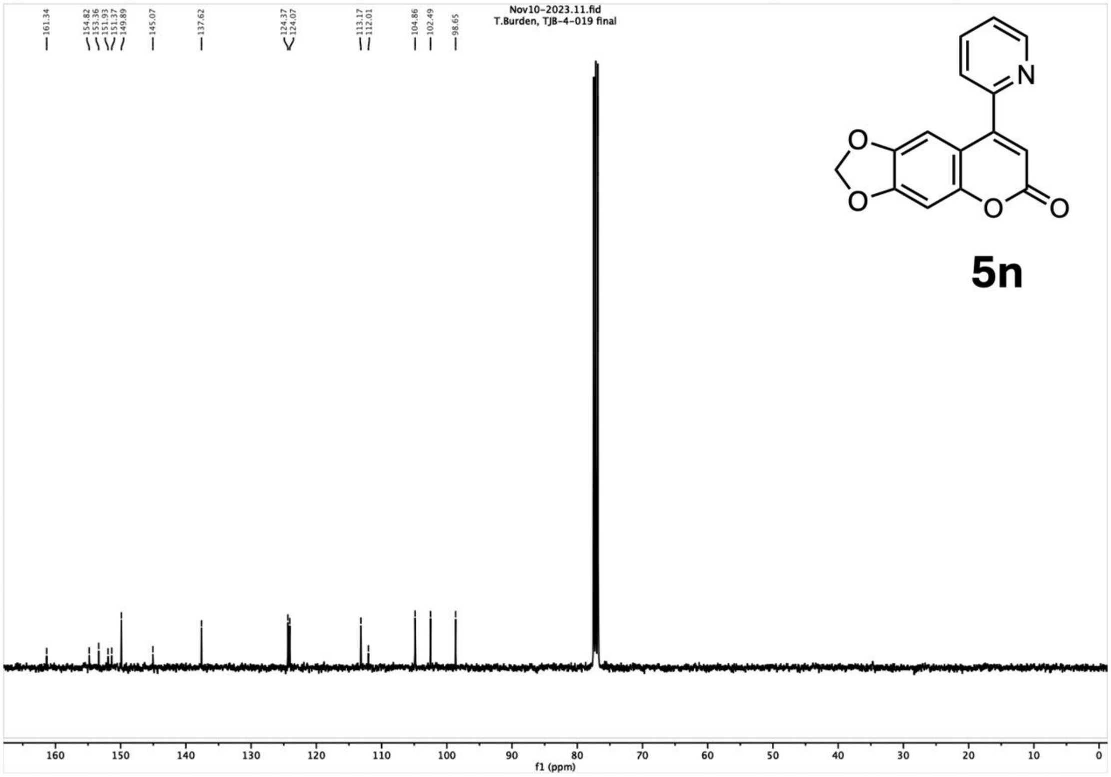

Figure S86 $^{13}C\{^{1}H\}$ NMR Spectrum (101 MHz, $CDCl_3$) of **5n**.

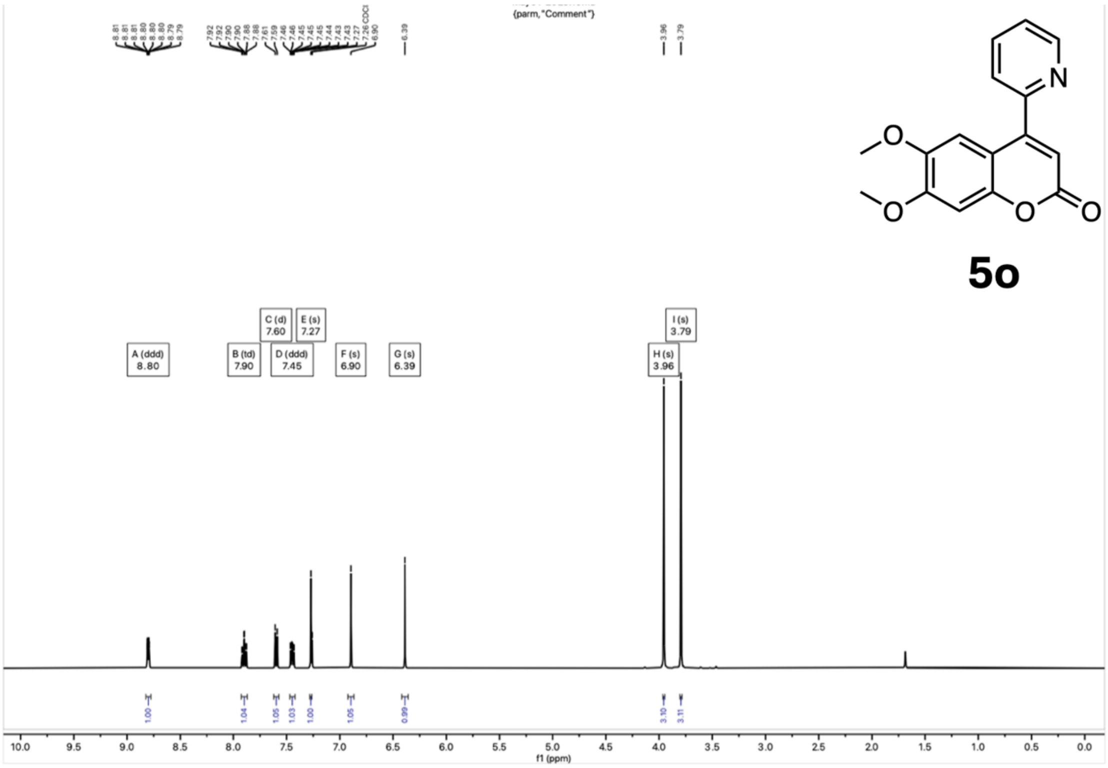

Figure S87 ^{1}H NMR Spectrum (400 MHz, $CDCl_3$) of **5o**.

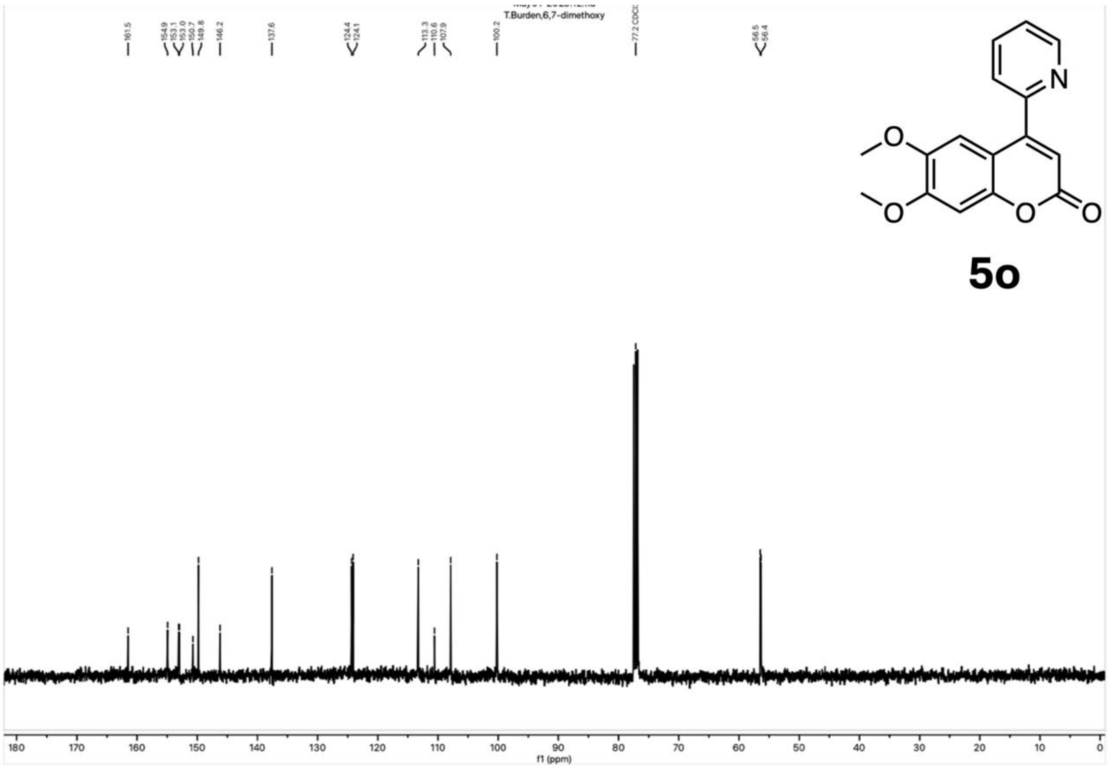

Figure S88 $^{13}C\{^{1}H\}$ NMR Spectrum (101 MHz, $CDCl_3$) of **5o**.

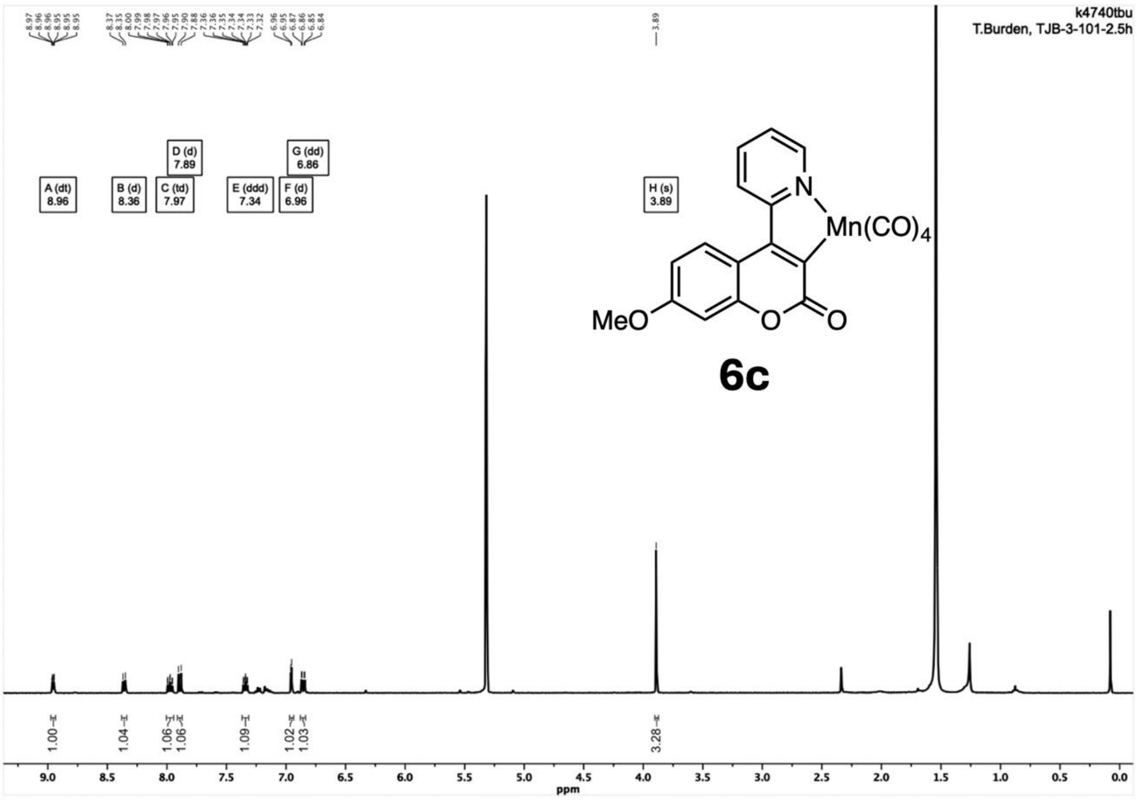

Figure S89 ^{1}H NMR Spectrum (400 MHz, CD_2Cl_2) of **6c**.

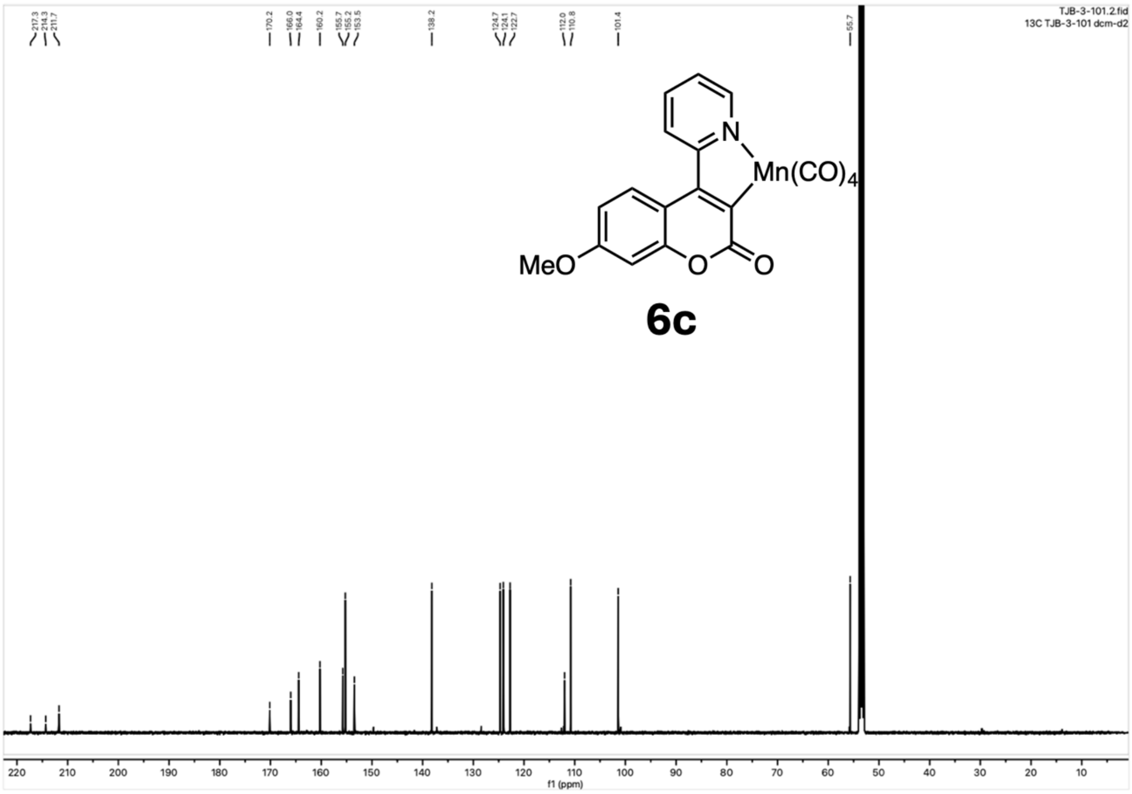

Figure S90 $^{13}C\{^{1}H\}$ NMR Spectrum (126 MHz, CD_2Cl_2) of **6c**.

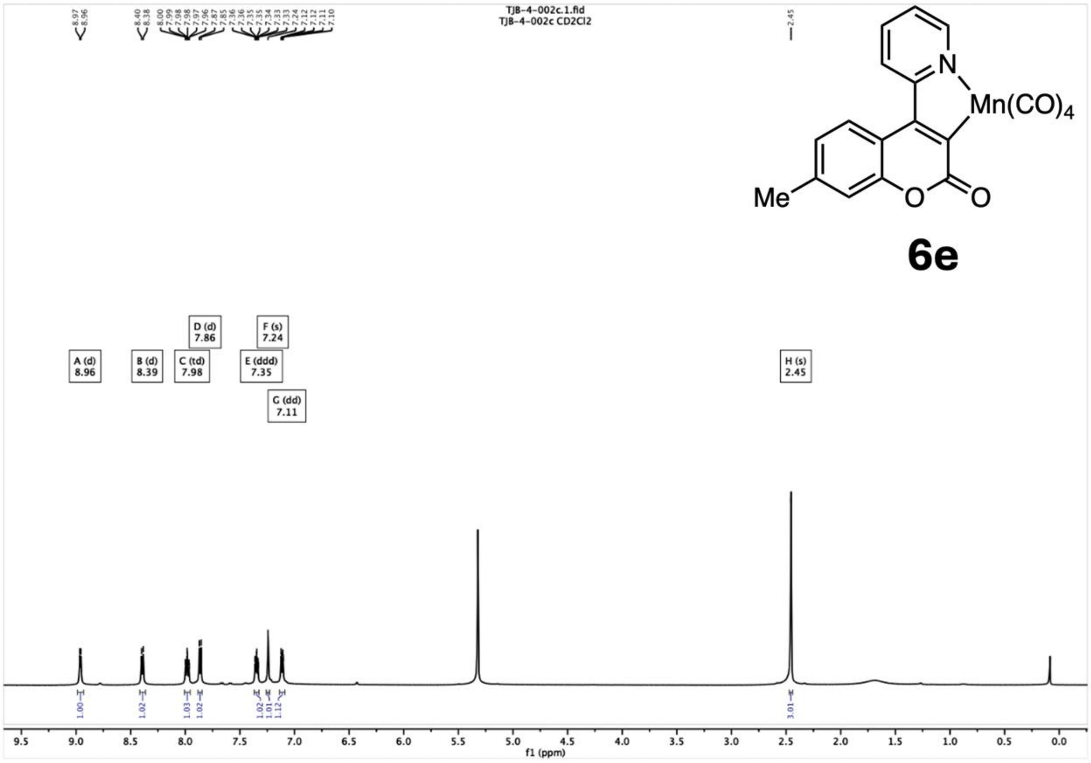

Figure S91 ^{1}H NMR Spectrum (500 MHz, CD_2Cl_2) of **6e**.

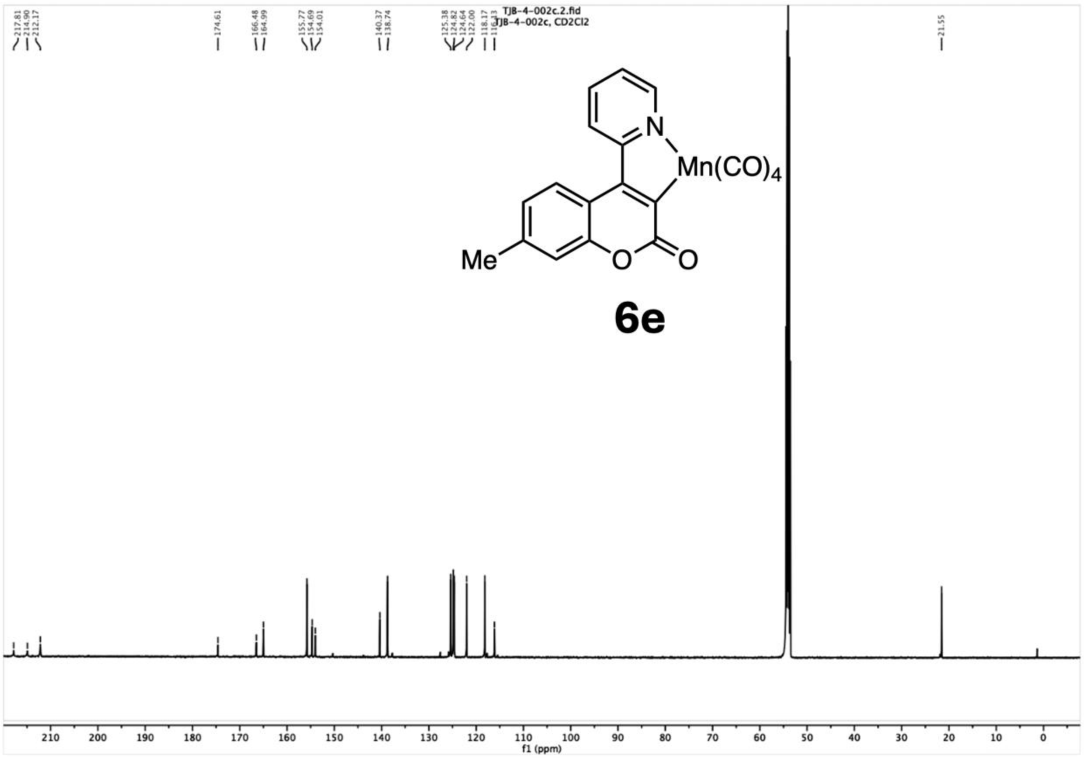

Figure S92 $^{13}C\{^{1}H\}$ NMR Spectrum (126 MHz, CD_2Cl_2) of **6e.**

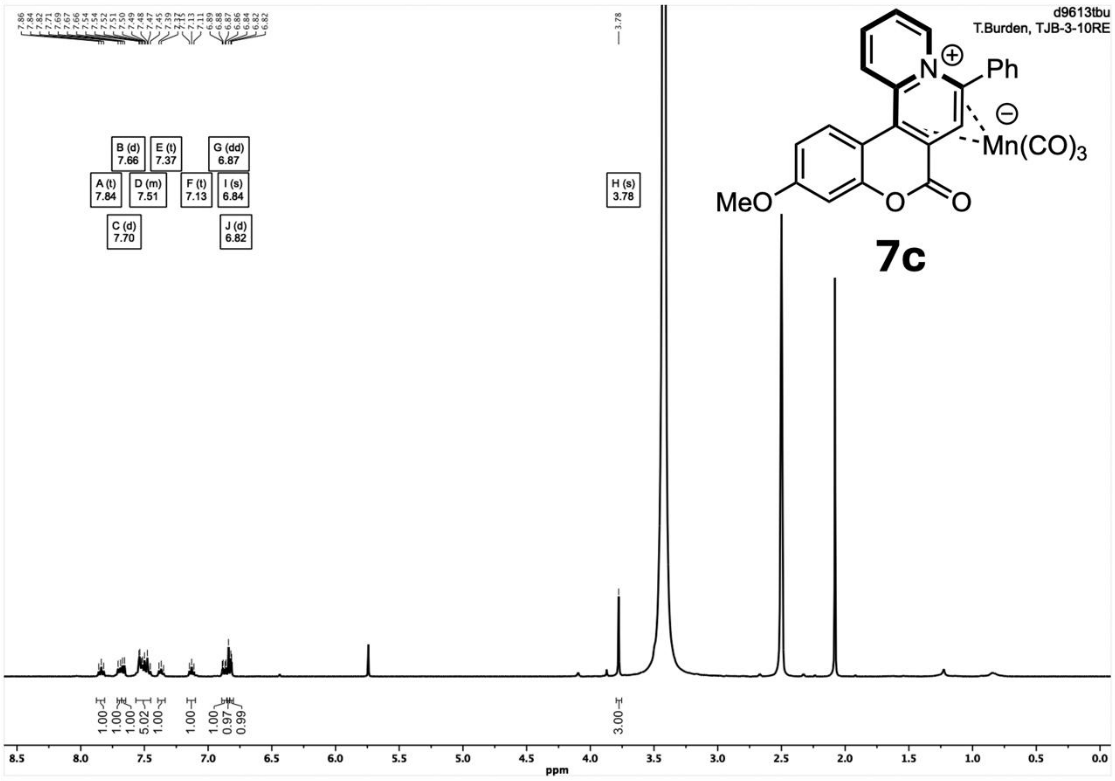

Figure S93 ^{1}H NMR Spectrum (400 MHz, DMSO-d_6) of **7c**.

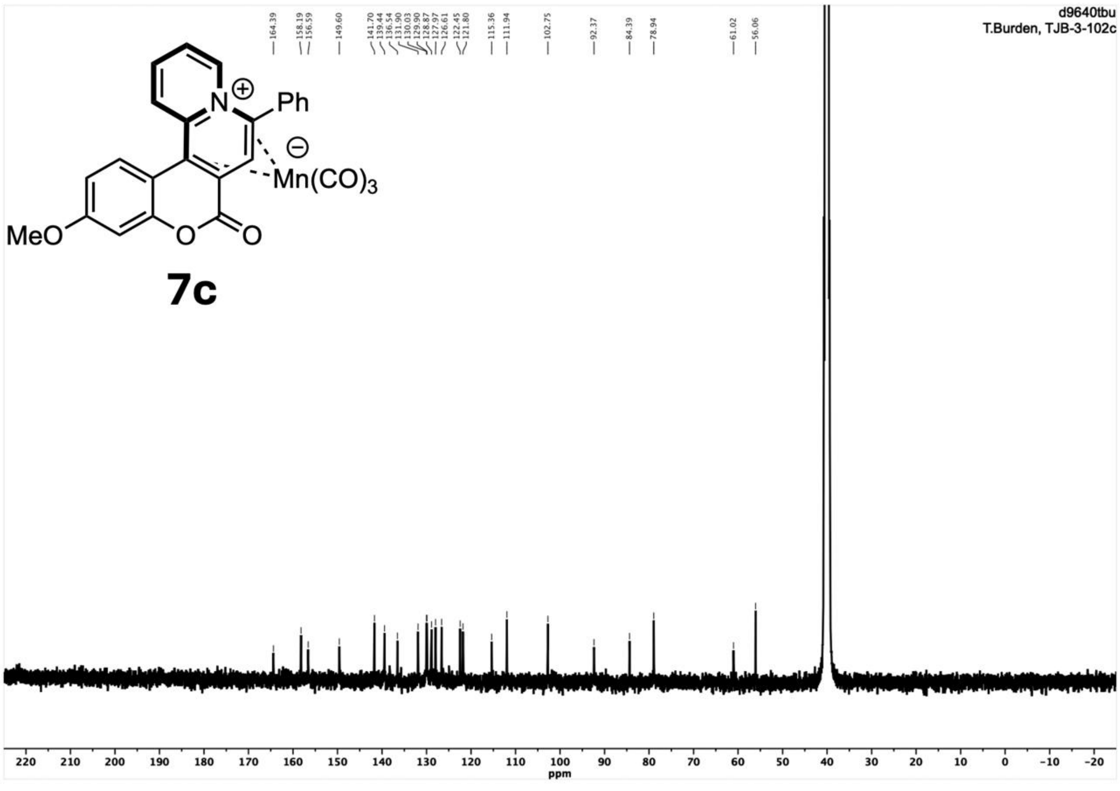

Figure S94 $^{13}C\{^{1}H\}$ NMR Spectrum (101 MHz, DMSO-d_6) of **7c**.

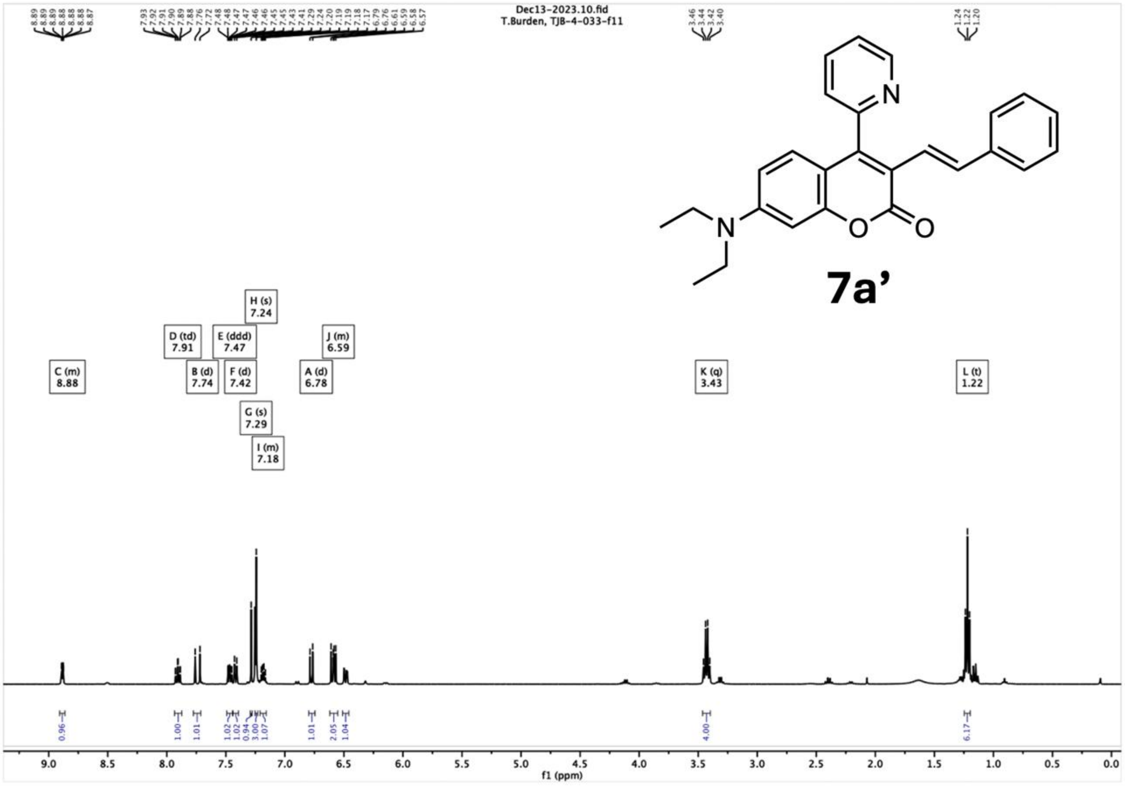

Figure S95 ^{1}H NMR Spectrum (400 MHz, $CDCl_3$) of **7a’**.

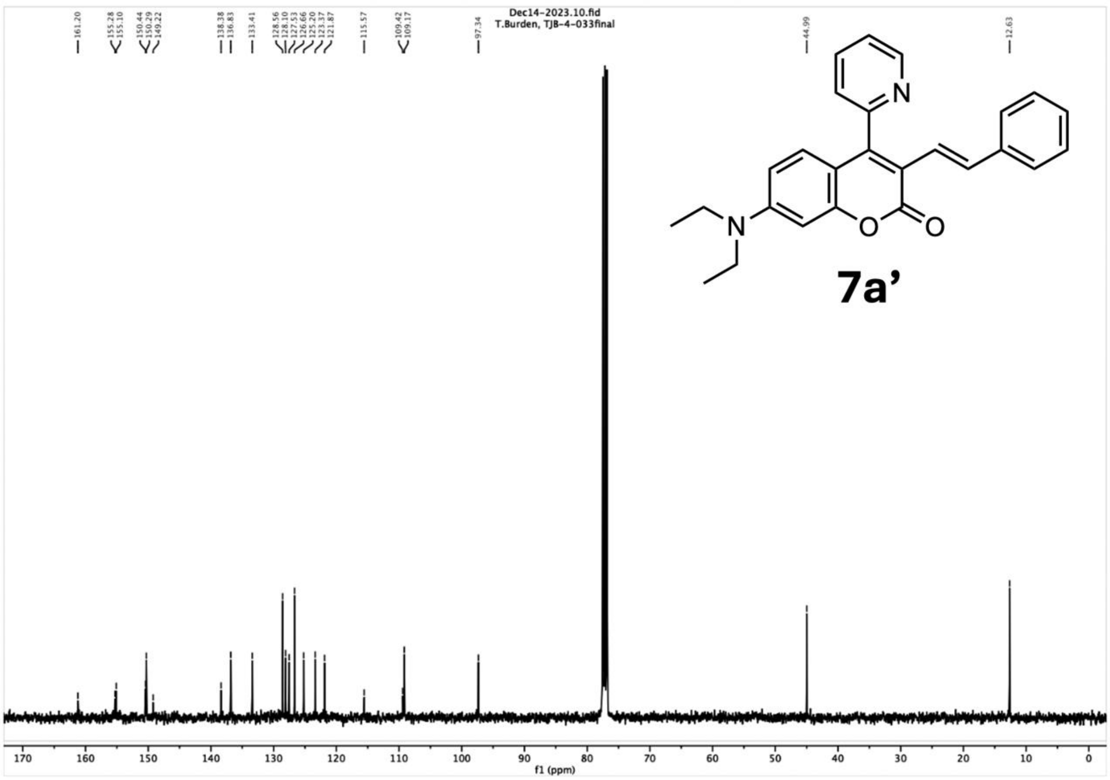

Figure S96 $^{13}C\{^{1}H\}$ NMR Spectrum (101 MHz, $CDCl_3$) of **7a’**.

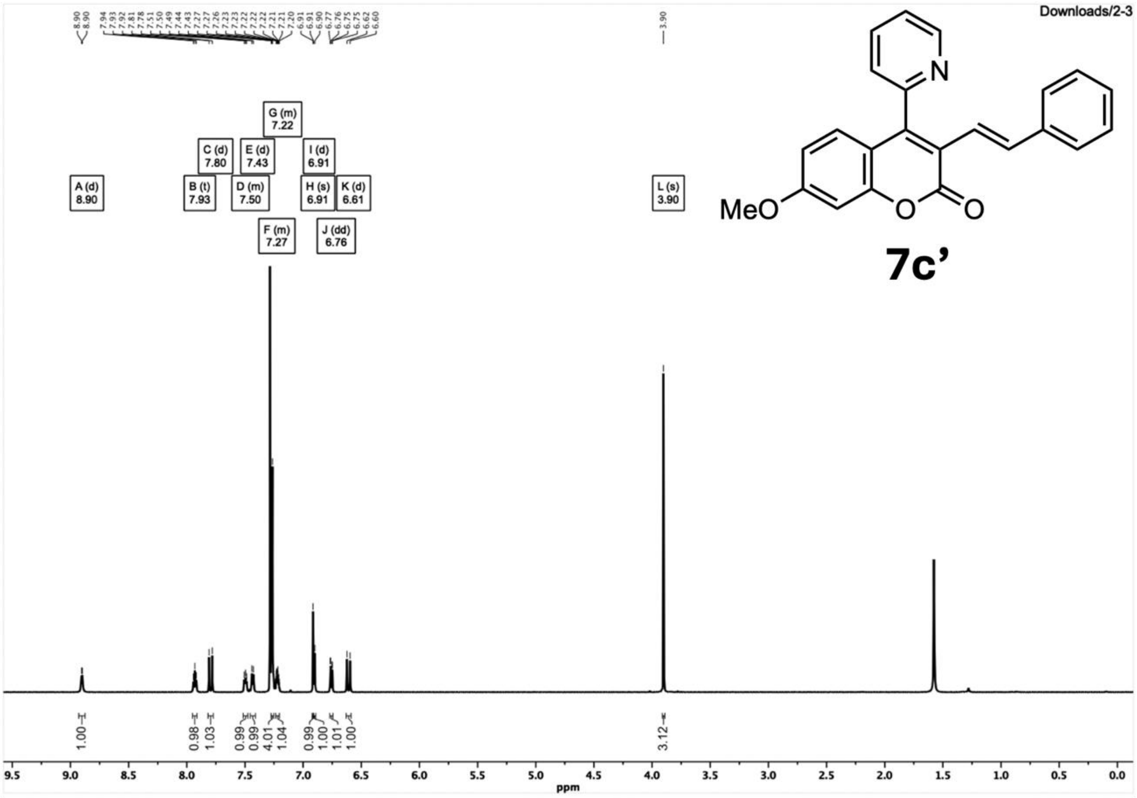

Figure S97 ^{1}H NMR Spectrum (600 MHz, $CDCl_3$) of **7c’**.

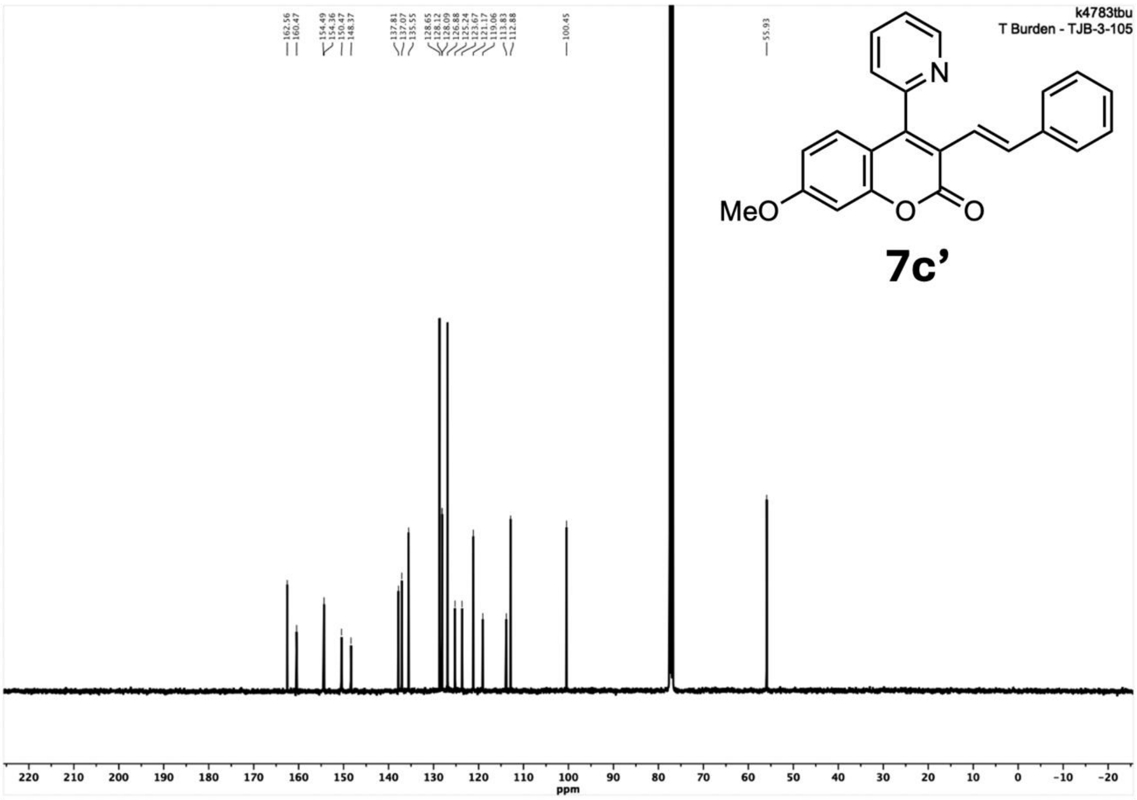

Figure S98 $^{13}C\{^{1}H\}$ NMR Spectrum (101 MHz, $CDCl_3$) of **7c’**.

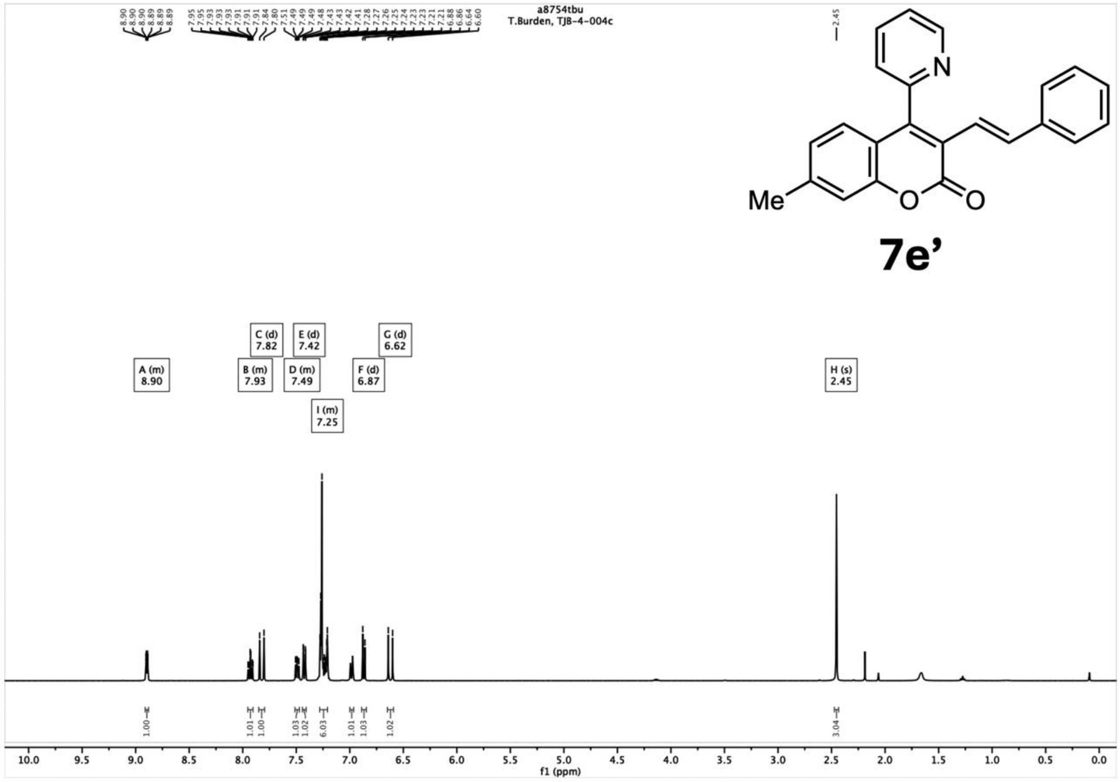

Figure S99 ^{1}H NMR Spectrum (400 MHz, $CDCl_3$) of **7e'**.

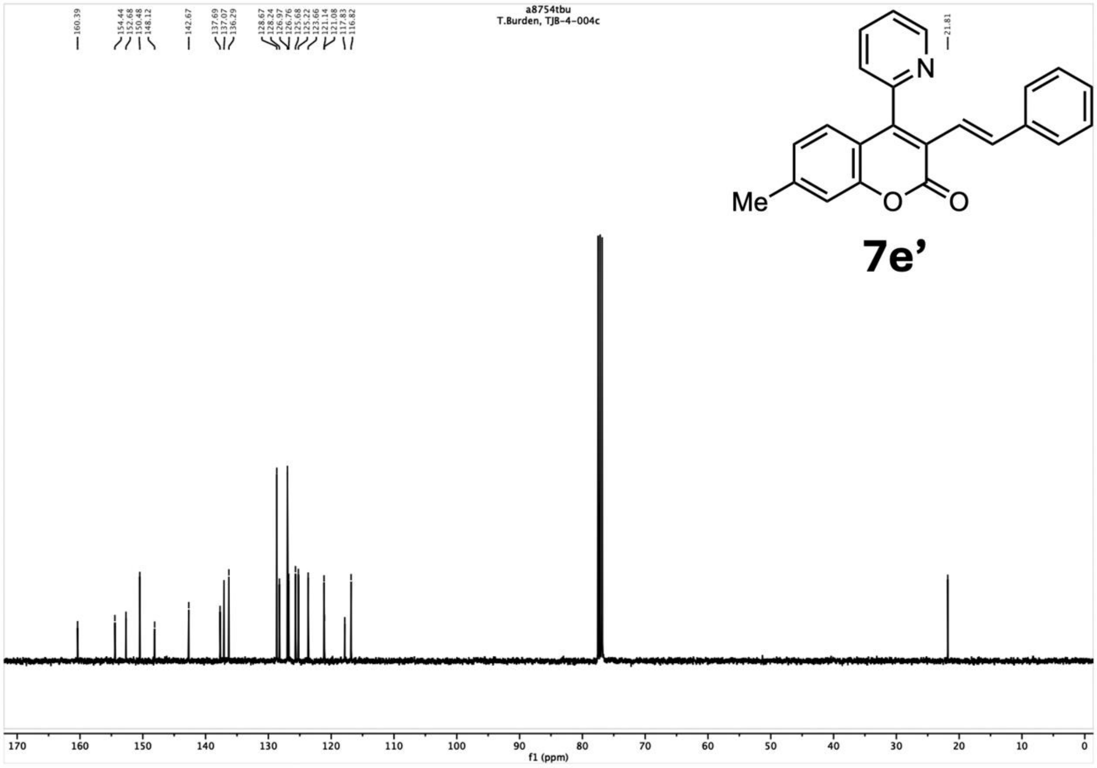

Figure S100 $^{13}C\{^{1}H\}$ NMR Spectrum (101 MHz, $CDCl_3$) of **7e'**.

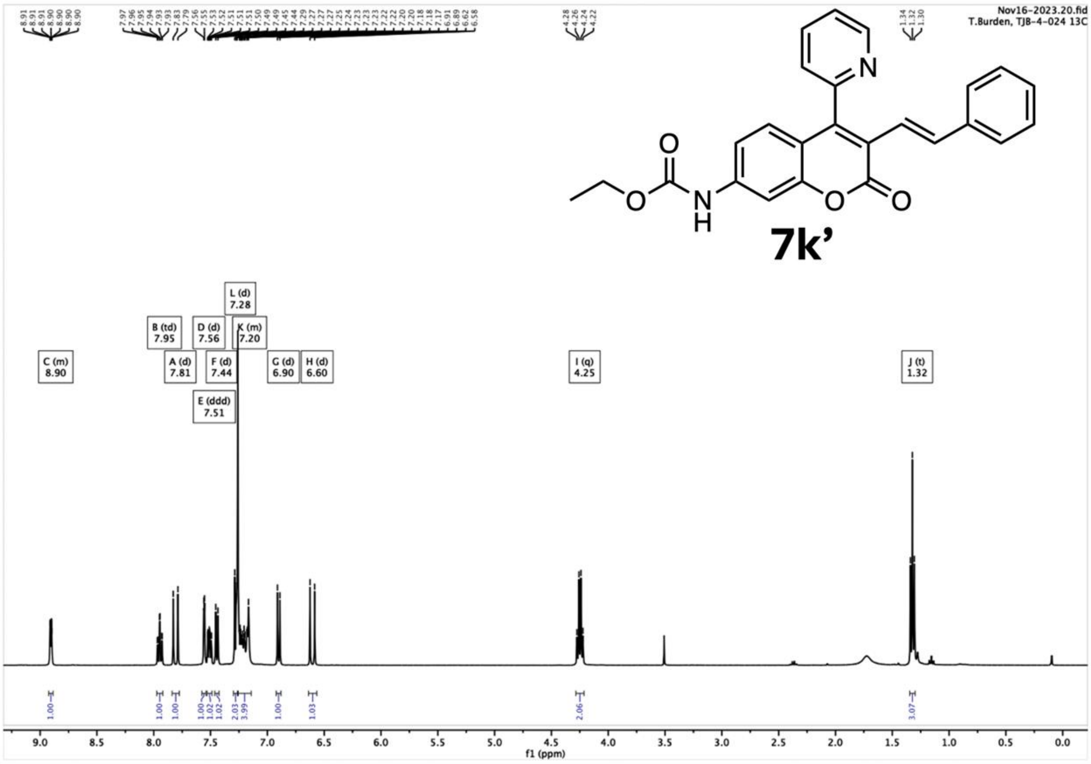

Figure S101 ^{1}H NMR Spectrum (400 MHz, $CDCl_3$) of **7k’**.

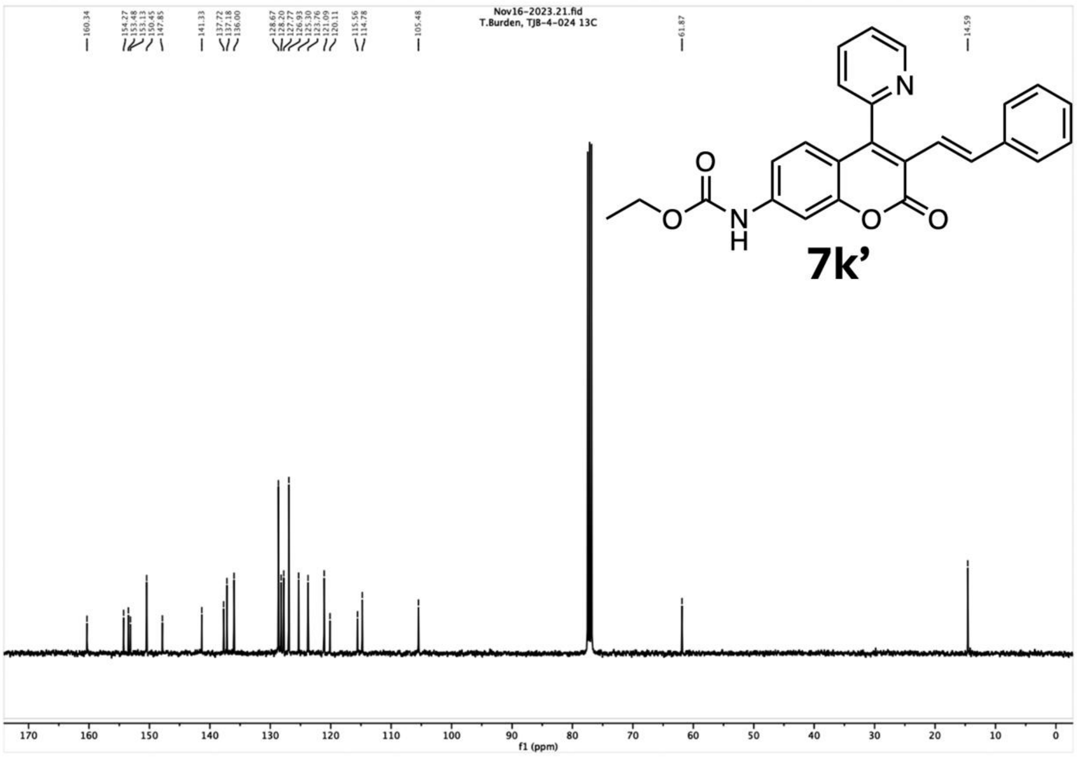

Figure S102 $^{13}C\{^{1}H\}$ NMR Spectrum (101 MHz, $CDCl_3$) of **7k’**.

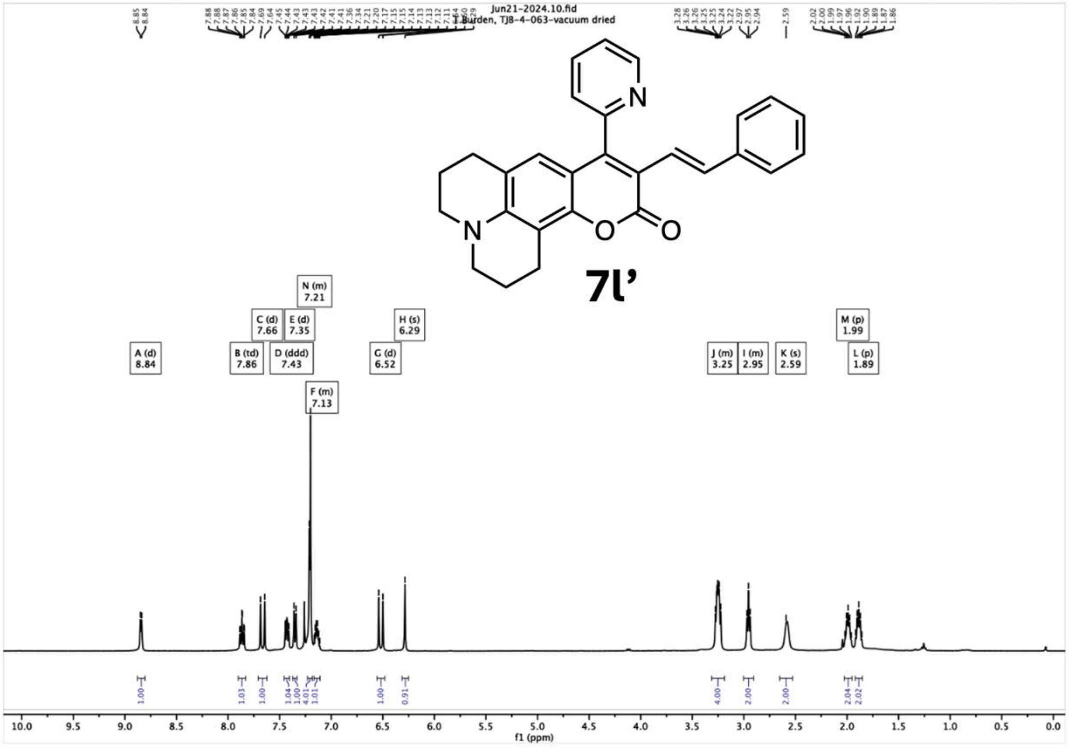

Figure S103 ^{1}H NMR Spectrum (400 MHz, $CDCl_3$) of **7l’**.

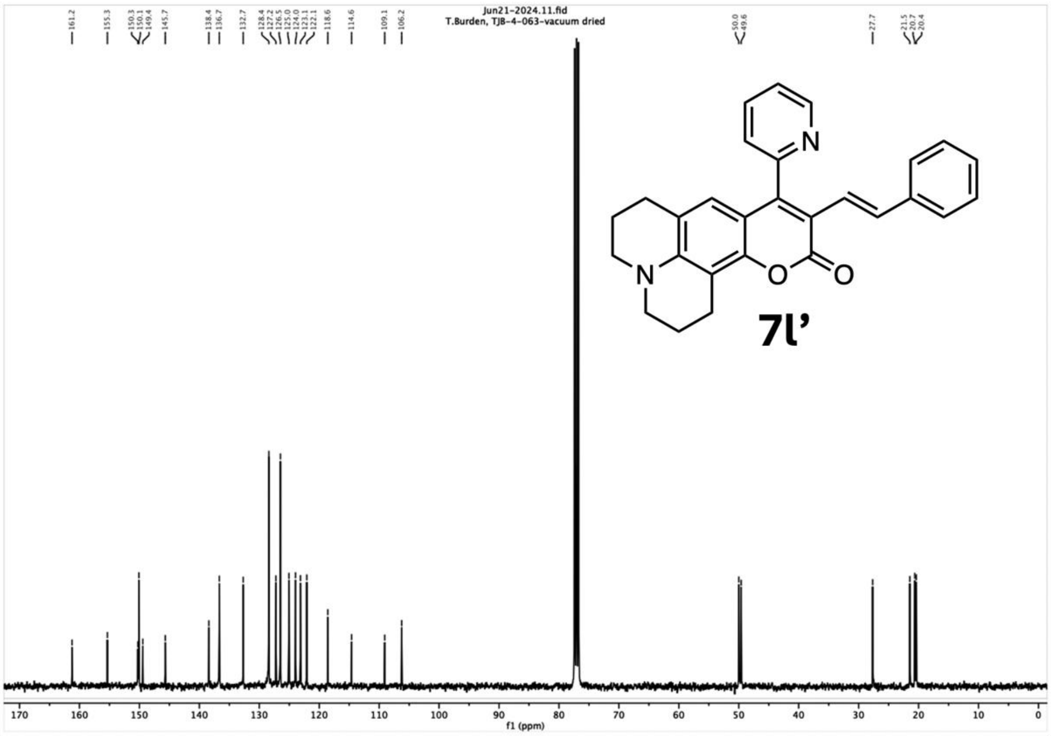

Figure S104 $^{13}C\{^{1}H\}$ NMR Spectrum (101 MHz, $CDCl_3$) of **7l’**.

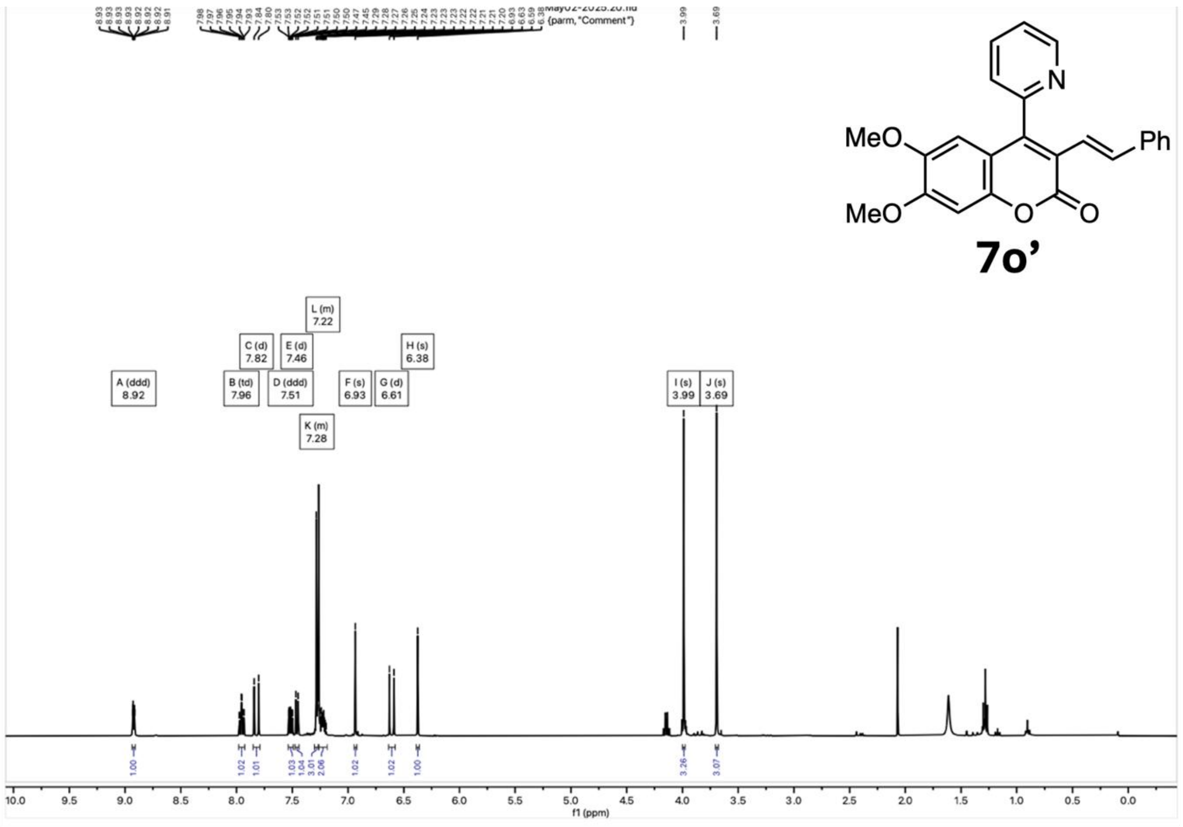

Figure S105 ^{1}H NMR Spectrum (400 MHz, $CDCl_3$) of **7o’**.

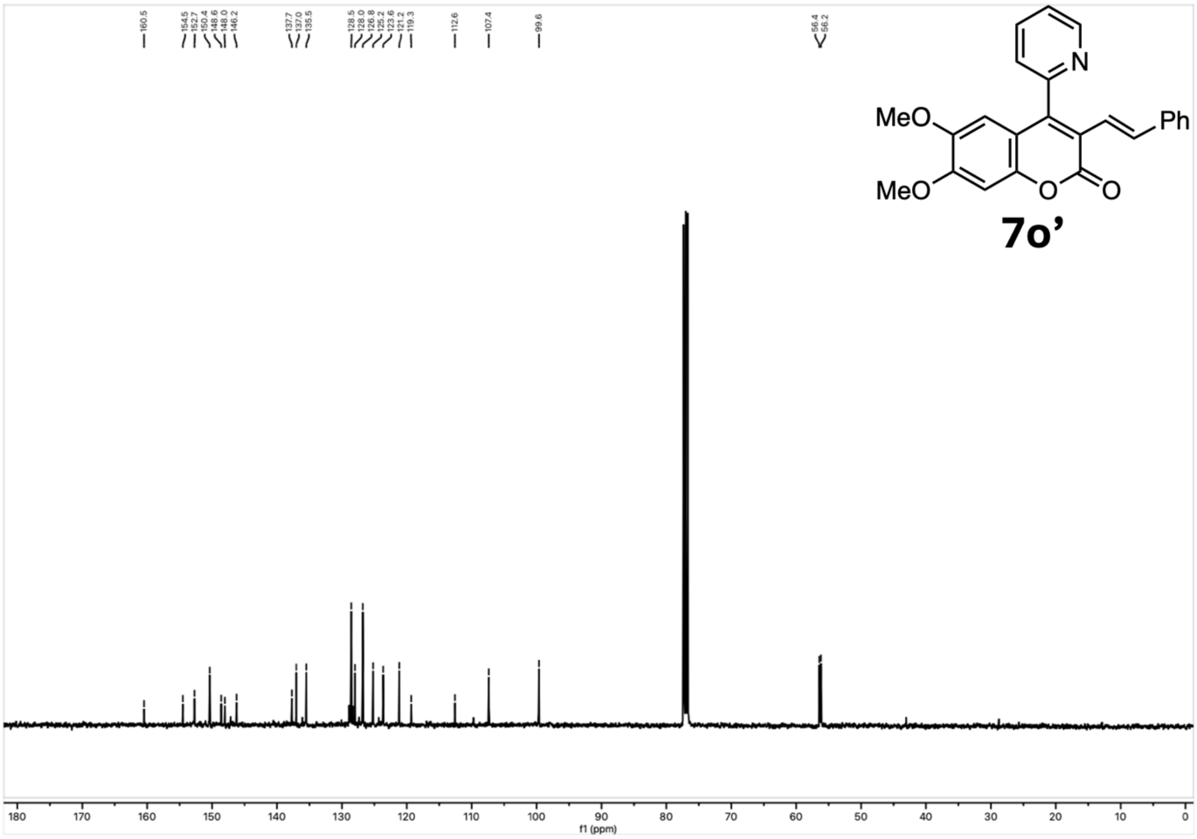

Figure S106 $^{13}C\{^{1}H\}$ NMR Spectrum (101 MHz, $CDCl_3$) of **7o’**.

21 References

(1) Greetham, G. M.; Pollard, M. R. ULTRA View Data Analysis. *ULTRA View Data Analysis, STFC* **2012**, No. 2.

(2) OriginPro 2019. *OriginPro Corporation* **2019**, Northampton, MA.

(3) Hammarback, L. A.; Robinson, A.; Lynam, J. M.; Fairlamb, I. J. S. Mechanistic Insight into Catalytic Redox-Neutral C-H Bond Activation Involving Manganese(I) Carbonyls: Catalyst Activation, Turnover, and Deactivation Pathways Reveal an Intricate Network of Steps. *J. Am. Chem. Soc.* **2019**, *141* (6), 2316–2328.

(4) (a) Burden, T. J.; Fernandez, K. P. R.; Kagoro, M.; Eastwood, J. B.; Tanner, T. F. N.; Whitwood, A. C.; Clark, I. P.; Towrie, M.; Krieger, J.; Lynam, J. M.; Fairlamb, I. J. S. Coumarin C–H Functionalization by Mn(I) Carbonyls: Mechanistic Insight by Ultra-Fast IR Spectroscopic Analysis. *Chem. Eur. J.* **2023**, *29*, e202203038. (b) Da, Y.-X.; Quan, Z.-Y. 3-(Pyridin-2-yl)coumarin. *Acta Crystallogr., Sect. E: Struct. Rep. Online*, **201 0**, *66*, o2872.

(5) Chuodhary, S.; Cannas, D. M.; Wheatley, M.; Larrosa, I. A Manganese(I)Tricarbonyl-Catalyst for near Room Temperature Alkene and Alkyne Hydroarylation. *Chem. Sci.* **2022**, *13* (44), 13225–13230.

(6) Pivetta, T.; Masuri, S.; Cabiddu, M. G.; Caltagirone, C.; Pintus, A.; Massa, M.; Isaia, F.; Cadoni, E. A Novel Ratiometric and Turn-on Fluorescent Coumarin-Based Probe for Fe(III). *New J. Chem.* **2019**, *43* (30), 12032–12041.

(7) Liu, J.; Zhang, X.; Shi, L.; Liu, M.; Yue, Y.; Li, F.; Zhuo, K. Base-Promoted Synthesis of Coumarins from Salicylaldehydes and Aryl-Substituted 1,1-Dibromo-1-Alkenes under Transition-Metal-Free Conditions. *Chem. Commun.* **2014**, *50* (69), 9887.

(8) Ahlrichs, R.; Bär, M.; Häser, M.; Horn, H.; Kölmel, C. Electronic Structure Calculations on Workstation Computers: The Program System Turbomole. *Chemical Physics Letters* **1989**, *162* (3), 165–169.

(9) Deglmann, P.; Furche, F.; Ahlrichs, R. An Efficient Implementation of Second Analytical Derivatives for Density Functional Methods. *Chemical Physics Letters* **2002**, *362* (5–6), 511–518.

(10) Deglmann, P.; May, K.; Furche, F.; Ahlrichs, R. Nuclear Second Analytical Derivative Calculations Using Auxiliary Basis Set Expansions. *Chemical Physics Letters* **2004**, *384* (1–3), 103–107.

(11) Eichkorn, K.; Treutler, O.; Öhm, H.; Häser, M.; Ahlrichs, R. Auxiliary Basis Sets to Approximate Coulomb Potentials. *Chemical Physics Letters* **1995**, *240* (4), 283–290.

(12) Eichkorn, K.; Weigend, F.; Treutler, O.; Ahlrichs, R. Auxiliary Basis Sets for Main Row Atoms and Transition Metals and Their Use to Approximate Coulomb Potentials. *Theoretical Chemistry Accounts: Theory, Computation, and Modeling (Theoretica Chimica Acta)* **1997**, *97* (1–4), 119–124.

(13) Von Arnim, M.; Ahlrichs, R. Geometry Optimization in Generalized Natural Internal Coordinates. *The Journal of Chemical Physics* **1999**, *111* (20), 9183–9190.

(14) Treutler, O.; Ahlrichs, R. Efficient Molecular Numerical Integration Schemes. *The Journal of Chemical Physics* **1995**, *102* (1), 346–354.

(15) Klamt, A.; Schüürmann, G. COSMO: A New Approach to Dielectric Screening in Solvents with Explicit Expressions for the Screening Energy and Its Gradient. *J. Chem. Soc., Perkin Trans. 2* **1993**, No. 5, 799–805.

(16) Grimme, S.; Antony, J.; Ehrlich, S.; Krieg, H. A Consistent and Accurate *Ab Initio* Parametrization of Density Functional Dispersion Correction (DFT-D) for the 94 Elements H-Pu. *The Journal of Chemical Physics* **2010**, *132* (15), 154104.
(17) Grimme, S.; Ehrlich, S.; Goerigk, L. Effect of the Damping Function in Dispersion Corrected Density Functional Theory. *J. Comput. Chem.* **2011**, *32* (7), 1456–1465.
(18) Glendening, E. D.; Badenhoop, J. K.; Reed, A. E.; Carpenter, J. E.; Bohmann, J. A.; Morales, C. M.; Karafiloglou, P.; Landis, C. R.; Weinhold, F. NBO 7.0., 2018.
(19) Frisch, M. J.; Trucks, G. W.; Schlegel, H. B.; Scuseria, G. E.; Robb, M. A.; Cheeseman, J. R.; Scalmani, G.; Barone, G. A.; Petersson, G. A.; Nakatsuji, H.; Li, X.; Caricato, M.; Marenich, A. V.; Bloino, J.; Janesko, B. G.; Gomberts, R.; Mennucci, B.; Hratchian, H. P.; Ortiz, J. V.; Izmaylov, A. F.; Sonnenberg, J. L.; Ding, F.; Lipparini, F.; Egidi, F.; Goings, J.; Peng, B.; Petrone, A.; Henderson, T.; Ranasinghe, D.; Zakrzewski, V. G.; Gao, J.; Rega, N.; Zheng, G.; Liang, W.; Hada, M.; Ehara, M.; Toyota, K.; Fukuda, R.; Hasegawa, J.; Ishida, M.; Nakajima, T.; Honda, Y.; Kitao, O.; Nakai, H.; Vreven, T.; Throssell, K.; Montgomery Jr, J. A.; Peralta, J. E.; Ogliaro, F.; Bearpark, M. J.; Heyd, J. J.; Brothers, E. N.; Kudin, K. N.; Staroverov, V. N.; Keith, T. A.; Kobayashi, R.; Normand, J.; Raghavachari, K.; Rendell, A. P.; Burant, J. C.; Lyengar, S. S.; Tomasi, J.; Cossi, M.; Millam, J. M.; Klene, M.; Adamo, C.; Cammi, R.; Ochterski, J. W.; Martin, R. L.; Morokuma, K.; Farkas, O.; Foresman, J. B.; Fox, D. J. Gaussian.
(20) Lu, T.; Chen, F. Multiwfn: A Multifunctional Wavefunction Analyzer. *J Comput Chem* **2012**, *33* (5), 580–592.
(21) Roy Dennington, T. A. K.; Millam, J. M. GaussView, 2016.
(22) Hansch, Corwin.; Leo, A.; Taft, R. W. A Survey of Hammett Substituent Constants and Resonance and Field Parameters. *Chem. Rev.* **1991**, *91* (2), 165–195.
(23) CrysAlisPro. CrysAlisPro. *Oxford Diffraction Ltd* **2009**, Version 1.171.34.41.
(24) Dolomanov, O. V.; Bourhis, L. J.; Gildea, R. J.; Howard, J. A. K.; Puschmann, H. *OLEX2* : A Complete Structure Solution, Refinement and Analysis Program. *J Appl Crystallogr* **2009**, *42* (2), 339–341.
(25) Sheldrick, G. M. *SHELXT* – Integrated Space-Group and Crystal-Structure Determination. *Acta Crysta.* **2015**, *71* (1), 3–8.
(26) Palatinus, L.; Chapuis, G. *SUPERFLIP* – a Computer Program for the Solution of Crystal Structures by Charge Flipping in Arbitrary Dimensions. *J Appl Crystallogr* **2007**, *40* (4), 786–790.
(27) Betteridge, P. W.; Carruthers, J. R.; Cooper, R. I.; Prout, K.; Watkin, D. J. CRYSTALS. *J. Appl. Cryst.* **2003**, No. 36, 1487.
(28) Winter, G.; Waterman, D. G.; Parkhurst, J. M.; Brewster, A. S.; Gildea, R. J.; Gerstel, M.; Fuentes-Montero, L.; Vollmar, M.; Michels-Clark, T.; Young, I. D.; Sauter, N. K.; Evans, G. *DIALS* : Implementation and Evaluation of a New Integration Package. *Acta Crystallogr D Struct Biol* **2018**, *74* (2), 85–97.
(29) Sheldrick, G. M. Crystal Structure Refinement with *SHELXL*. *Acta Crystallogr C Struct Chem* **2015**, *71* (1), 3–8.
(30) Peng, L.-M. Electron Atomic Scattering Factors and Scattering Potentials of Crystals. *Micron* **1999**, *30* (6), 625–648.
(31) Gruene, T.; Hahn, H. W.; Luebben, A. V.; Meilleur, F.; Sheldrick, G. M. Refinement of Macromolecular Structures against Neutron Data with *SHELXL2013*. *J. Appl. Cryst.* **2014**, *47* (1), 462–466.

(32) Thorn, A.; Dittrich, B.; Sheldrick, G. M. Enhanced Rigid-Bond Restraints. *Acta Cryst.* **2012**, *68* (4), 448–451.
